# Hydroxyl-rich nanocavities on perovskite enable nearly barrierless intramolecular hydrogen transfer for nitrate electroreduction to ammonia

**DOI:** 10.1038/s41467-026-74405-1

**Published:** 2026-06-29

**Authors:** Mingkai Xu, Ruizhao Wang, Zaixing Wang, Zheng Tang, Wei-Hsiang Huang, Wang Tan, Lingjie Yuan, Huanhuan Tao, Zhongliang Dong, Min-Hsin Yeh, Chih-Wen Pao, Jun Yin, Zhiwei Hu, Jie Dai, Yinlong Zhu

**Affiliations:** 1 State Key Laboratory of Mechanics and Control for Mechanical Structures, Key Laboratory for Intelligent Nano Materials and Devices of the Ministry of Education, Institute for Frontier Science, Nanjing University of Aeronautics and Astronautics, Nanjing, China; 2 Center of Hydrogen Science, School of Materials Science and Engineering, Shanghai Jiao Tong University, Shanghai, China; 3 Zhangjiang Institute for Advanced Study (ZIAS), Shanghai Jiao Tong University, Shanghai, China; 4 Department of Applied Physics, The Hong Kong Polytechnic University, Hong Kong, China; 5 National Synchrotron Radiation Research Center (NSRRC), Hsinchu, Taiwan; 6 Sustainable Electrochemical Energy Development (SEED) Center, National Taiwan University of Science and Technology, Taipei, Taiwan; 7 State Key Laboratory of Mechanics and Control of Mechanical Structures, Key Laboratory for Intelligent Nano Materials and Devices of the Ministry of Education, College of Aerospace Engineering, Nanjing University of Aeronautics and Astronautics, Nanjing, China; 8 Department of Chemical Engineering, National Taiwan University of Science and Technology, Taipei, Taiwan; 9 Max Planck Institute for Chemical Physics of Solids, Nothnitzer Strasse 40, Dresden, Germany

**Keywords:** Electrocatalysis, Structural properties, Energy, Catalytic mechanisms, Electrocatalysis

## Abstract

Electrocatalytic nitrate reduction to ammonia (NITRR) provides a sustainable avenue for simultaneous nitrate mitigation and ammonia synthesis, but the sluggish surface hydrogen migration during NITRR remains a major bottleneck. Here, we show a barrierless hydrogen transfer pathway along intramolecular hydrogen bonds between hydroxyls of hydroxyl-rich nanocavities for efficient nitrate electroreduction to ammonia. This nanocavity is constructed via electrochemical reduction-assisted selective Sr ions leaching on the La_0.4_Sr_0.6_FeO_3-δ_ perovskites. Combined experimental and theoretical investigations reveal that the nanocavity features nanocavity-like architecture with hydroxyl-enriched walls, boosting active hydrogen generating and hopping for NO_3_^-^ hydrogenation. Benefiting from such unusual intramolecular hydrogen transfer, the surface nanoconcaved La_0.4_Sr_0.6_FeO_3-δ_ achieves a Faradaic efficiency of 97.81 % and an ammonia yield rate of 51.37 mg h^−1^ cm^−2^ at −0.8 V versus reversible hydrogen electrode (RHE), surpassing nanocavity-free counterpart and ranking among superior NITRR catalysts. Ampere-level current density of nitrate-to-ammonia conversion are further realized in a renewable-energy-powered electrolyzer at a very low cell voltage of 2.23 V. Techno-economic analysis underscores dual benefits of this process including economic viability in ammonia synthesis and environmental impact in nitrate remediation.

## Abstract (web summary)

Electrocatalytic nitrate reduction offers a dual-effect solution for nitrate remediation and ammonia synthesis, yet slow hydrogen migration limits its efficiency. Here, the authors design hydroxyl-rich nanocavities on perovskites to enable rapid hydrogen transfer for efficient ammonia production.

## Introduction

Ammonia (NH_3_), a vital industrial chemical with annual consumption over 150 million tons, is mainly produced via the energy-intensive Haber-Bosch process (350–500 °C, 15–30 MPa), which accounts for 1.5% of global energy use and 1.3% of anthropogenic CO_2_ emissions, consequently hindering carbon neutrality^1,2^. Currently, nitrate (NO_3_^-^) pollution from industrial, agricultural and domestic sources causes eutrophication, hypoxic zones and groundwater contamination, often exceeding WHO safety limits (50 mg L^−1^) by orders of magnitude^3,4^. The electrocatalytic NO_3_^-^ reduction reaction into NH_3_ offers a solution for addressing both sustainable energy needs and environmental remediation challenges.

The electrocatalytic nitrate reduction reaction (NITRR) generally proceeds through the adsorption of NO_3_^-^ onto catalysts’ surfaces, followed by a series of deoxygenation and subsequent hydrogenation steps, ultimately yielding NH_3_. Inspired by the iron (Fe)-cofactor in the nitrate enzymatic reduction and Fe-catalyzed Haber-Bosch process, Fe-based materials^5,6^, especially for unusually coordinated Fe centers with highly occupied *d*-orbitals and open *d*-orbital shells, have emerged as a promising class of electrocatalysts for NITRR due to favorable NO_3_^-^ adsorption and activation ability^7–10^. However, these NO_3_^-^-preoccupied transition Fe centers usually prohibit the competitive water or hydrogen adsorption and activation, limiting the active hydrogen supply for sustaining effective hydrogenation of adsorbed NO_3_^-^ to NH_3_. Recent advances highlighted the potential of integrating a water-dissociation active phase into Fe-based NITRR electrocatalysts to balance nitrate and water adsorption through dual-site configuration^8^, self-corrosion reconstruction^11^, interfacial engineering^12^ and alloying methodologies^13^. Notably, nanoconfinement engineering that can modulate the interfacial water structure, has become promising strategies for reducing the energy barriers of water dissociation through reorganizing hydrogen-bond networks into low-coordination water configurations^14–19^, but this approach has been rarely explored in the field of NITRR. Besides, within nanoconfined environments, the migration of space-confined active hydrogen species (^*^H) is always restricted due to the spatial constraint and the local electric field concentration^20–22^. If ^*^H fails to timely spillover to hydrogenation-active sites, it is forced to either combine with adjacent H_2_O or ^*^H to form H_2_ or diffuse outward bulk water through the hydrogen-bonding network, leading to low ^*^H utilization efficiency and limited targeted NH_3_ generation^14^. Therefore, a precisely designed nanoconfined catalysis system with feasible ^*^H mobility pathways is urgently needed for advancing the NITRR electrocatalysts.

Selective ion exchange between proton and metal ions at the electrolyte/electrocatalyst interface is expected to create surface nanocavities for reconfiguring the interfacial water structure owing to significant differences in the ionic radii^19,23–25^. Fortunately, perovskite oxides with an ABO_3_ framework, in which alkali, alkaline-earth or lanthanide cations (A-site) with large ionic radii (0.90 Å−1.40 Å) reside in the interstitial cuboctahedral cavities of B-site corner-sharing transition metal-oxygen octahedra, offer an ideal platform for effective surface nanoconfinement engineering^26–28^. This judgment is largely attributed to the chemical dissolution of alkali and alkaline-earth ions (*e.g*., Sr^2+^ ions) with high solubility in aqueous media for perovskite oxides, which has been extensively revealed in the oxygen evolution reactions (OER)^23–25^. Nevertheless, unlike the dissolution/re-deposition mechanism of B-site during lattice-oxygen participated OER, the dissolution of transition metal ions (*e.g*., Fe ions) is intrinsically inhibited at a negative bias potential^29^, which thereby can facilitate selective cation leaching to provide a confined nanocavity-like space for reconstructing interfacial water and exposing undercoordinated B-site metal catalytic centers. More importantly, water dissociation on perovskite oxides under cathodic polarization can generate ^*^OH on metal sites and ^*^H on lattice oxygen sites, subsequently shaping the hydroxyl-rich nanocavity walls. Intramolecular hydrogen bond among abundant hydroxyls on the nanocavity walls could create active hydrogen transfer mediators through possible hydrogen hopping effect^30–34^, possibly overcoming the intrinsic ^*^H transport limitations within confined nanostructures.

**Fig. 1:**
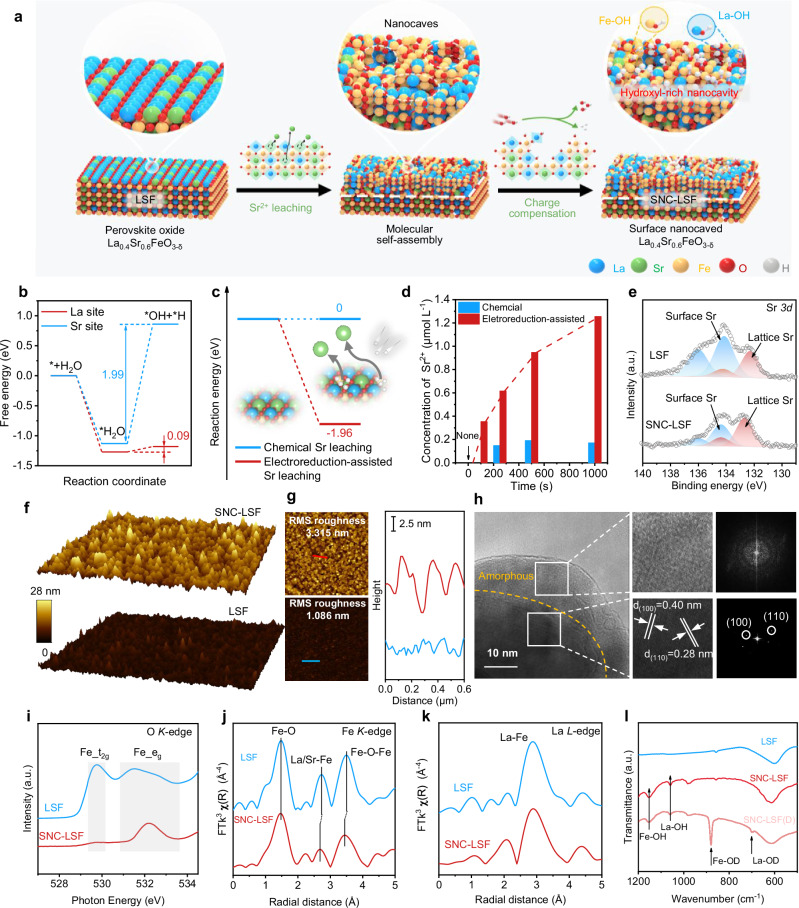
Preparation and characterization of SNC-LSF. **a** Schematic illustration of the preparation for the surface nano-concaved perovskite oxide La_0.4_Sr_0.6_FeO_3-δ_ catalyst (SNC-LSF) via a molecular self-assembly approach induced by the electrochemical reduction-assisted selective Sr^2+^ leaching. **b** Free energy of H_2_O dissociation on La or Sr site for LSF. **c** Reaction energy of Sr^2+^ leaching for LSF under different conditions. **d** Dissolution concentrations of Sr elements from LSF in 0.1 M KOH. **e** Sr 3 *d* XPS spectra of LSF and SNC-LSF. **f** AFM maps of LSF and SNC-LSF. **g** Root mean square (RMS) roughness and line scans of LSF and SNC-LSF. **h** HRTEM image of SNC-LSF and the corresponding FFT pattern. **i** O *K*-edge spectra of LSF and SNC-LSF in TEY mode. **j** FT-EXAFS spectra of LSF and SNC-LSF at the Fe *K*-edge. **k** FT-EXAFS spectra of LSF and SNC-LSF at the La *L*-edge. **l** FTIR spectra of LSF, SNC-LSF and SNC-LSF(D). Source data are provided as a Source Data file.

Motivated by above considerations, a surface nano-concaved perovskite oxide La_0.4_Sr_0.6_FeO_3-δ_ catalyst (denoted as SNC-LSF) was rationally designed via the electrochemical reduction-assisted selective Sr^2+^ leaching, as illustrated in Fig. 1a. The designed SNC-LSF catalyst exhibits a remarkable NH_3_ Faradaic efficiency of 97.81%, along with a production rate of 51.37 mg h^−1^ cm^−2^ at − 0.8 V vs. RHE, significantly outperforming pristine La_0.4_Sr_0.6_FeO_3-δ_ (LSF), counterpart LaFeO_3_ (LF) without surface nano-concavities and ranking among superior NITRR catalysts. Benefiting from surface reconstruction under cathodic polarization, the surface nanocavity-confined hydrogenation reactors over SNC-LSF were experimentally and theoretically demonstrated to not only offer more undercoordinated La/Fe-O moieties for initial NO_3_^-^ adsorption and confined spaces for reconfiguring interfacial water to accelerate H_2_O activation, but feature hydroxyl-enriched walls with intramolecular hydrogen bonds for a facile ^*^H transfer, synergistically contributing to its superior NITRR catalytic performance. More importantly, the general applicability of the electrochemical reduction-assisted selective alkaline-earth leaching in constructing nanocavity-confined hydrogenation reactors for boosting NITRR is confirmed in other perovskites with different compositions. The developed SNC-LSF catalyst was also integrated into a renewable-energy-powered membrane electrode assembly (MEA) electrolyzer with in situ ammonia stripping and absorption for industrial-level current density NO_3_^-^ reduction and high-purity NH_3_ recovery from NO_3_^-^-laden wastewaters. Comprehensive techno-economic analysis (TEA) further confirms the viability of this green NH_3_ production strategy as a promising complement to the conventional Haber-Bosch process.

## Results

### Preparation and characterization of SNC-LSF

As illustrated in Fig. 1a and Supplementary Fig. 1, soluble Sr^2+^ ions occupy the interstitial sites between the corner-sharing Fe-O octahedra in a robust three-dimensional lattice framework of La_0.4_Sr_0.6_FeO_3-δ_ (LSF) perovskites, which offers a platform to construct surface nanocages via selective Sr^2+^ leaching at the electrolyte/electrocatalyst interface^26,27^. The electrochemically-generated active hydrogen species through water dissociation under a reductive potential would prefer to bond with surface oxygen anions to compensate for charge imbalance during Sr^2+^ ions dissolution, thereby forming abundant hydroxyl species on the surface. Because the Sr^2+^ ion possesses a considerably large ionic radius, the electrochemical reduction-assisted selective ion exchange effect could further induce lattice distortion and give birth to surface nanoscale concavities^23,24^. Besides, such a rough surface on surface nano-concaved La_0.4_Sr_0.6_FeO_3-δ_ (SNC-LSF) can also expose more coordinatively unsaturated Fe sites for NO_3_^-^ adsorption. As expected, all La_1-x_Sr_x_FeO_3-δ_ (x = 0.2, 0.4, 0.6 and 0.8) with electrochemical reduction-assisted selective ion exchange (denoted as SNC-LSFx) exhibit evidently enhanced NITRR activity relative to LaFeO_3_ (denoted as LF) without surface nano-concavities (Supplementary Figs. 2–4). Considering that SNC-LSF0.6 has the highest NITRR activity among the SNC-LSFx, it is therefore selected as the model catalyst for subsequent studies, which is also referred as the representative SNC-LSF unless otherwise stated in this study.

Density functional theory (DFT) calculations were first performed to elucidate the mechanism of the electrochemical reduction-assisted selective ion exchange. Figure 1b reveals that both of the surface exposed La^3+^ and Sr^2+^ ions on LSF can easily adsorb H_2_O molecules (Supplementary data 1). La^3+^ sites dissociate H_2_O to generate ^*^H with a negligible free energy difference, suggesting its thermodynamically favorable water dissociation ability to supply ^*^H. In contrast, an intensively endothermic Volmer step over Sr sites suggests that Sr ions tend to firmly coordinate with H_2_O in solution, as previously reported^23,24,26^, facilitating the chemical leaching of surface Sr ions. As shown in Fig. 1c, with the assistance of ^*^H generated by electrochemical reduction, the required energy for Sr^2+^ leaching significantly drops to − 1.96 eV compared with the chemical leaching (Supplementary data 1). This result could be ascribed to a stronger H-O interaction relative to the Sr-O bond, which promotes surface element displacement during electrochemical reduction-assisted Sr^2+^ leaching^35^. Inductively coupled plasma optical emission spectroscopy (ICP-OES) analysis further validates that Sr^2+^ ion leaching could be substantially boosted by electrochemical reduction in comparison with negligible chemical Sr^2+^ ion leaching in solution without applying potentials. Meanwhile, no detectable La^3+^ or Fe^3+^ species were observed in the electrolyte (Fig. 1d and Supplementary Fig. 5), in agreement with prior reports, indicating the selective Sr^2+^ ions dissolution under cathodic bias^29^. In Sr 3 *d* X-ray photoelectron spectroscopy (XPS) (Fig. 1e), a notable intensity decrease of surface Sr species was observed in SNC-LSF compared with pristine LSF, further confirming significant Sr^2+^ leaching during the electrochemical process^36,37^. This electrochemically assisted ion exchange strategy is also applicable to the selective leaching of other alkaline earth metals (*e.g*., Ca^2+^ and Ba^2+^ ions) in the surface nano-concaved La_0.4_Ca_0.6_FeO_3-δ_ (SNC-LCF) and surface nano-concaved La_0.4_Ba_0.6_FeO_3-δ_ (SNC-LBF) catalysts (Supplementary Fig. 6), as revealed by the significant amount of Ba^2+^ and Ca^2+^ ions compared with undetectable La^3+^ or Fe^3+^ ions in electrolytes during electrochemical reduction activation in Supplementary Fig. 7. Analogous to SNC-LSF, SNC-LCF and SNC-LBF exhibit higher ammonia yield rates and Faradaic efficiencies than those of LF (Supplementary Fig. 8). The slight differences in NITRR activity among SNC-LCF, SNC-LSF, and SNC-LBF may be attributed to variations in the leaching rates and ionic radii of Ca^2+^, Sr^2+^ and Ba^2+^ ^38,39^.

We then investigated the surface topography change of LSF during electrochemical reduction-assisted Sr^2+^ leaching, and found that SNC-LSF exhibits a higher density of mesopores in the range of 2–10 nm and apparently increased BET surface area (22.018 m^2 ^g^−1^) relative to pristine LSF (8.823 m^2 ^g^−1^) (Supplementary Fig. 9). Atomic force microscopy (AFM) analysis (Fig. 1f and Supplementary Fig. 10) further indicates increased surface roughness of LSF during electrochemical reduction-assisted Sr^2+^ leaching^40^. Line-scan profiles (Fig. 1g) show that the root-mean-square (RMS) roughness rises from 1.086 nm for LSF to 3.315 nm for SNC-LSF, corroborating the formation of nanocavity-confined hydrogenation reactors. High-resolution AFM images provide close-up views of the nanocavities (Supplementary Fig. 11). Quantitative AFM analysis further reveals that, within a 0.5 × 0.5 μm region, approximately three nanocavities with sizes larger than 5 nm were observed along each edge on average (Supplementary Fig. 12). As expected, we observed the surface roughness enhancement in Supplementary Figs. 13, 14 for SNC-LCF and SNC-LBF as well, suggesting the generality of this strategy in constructing nanocavity-confined reactors. In addition, high-resolution transmission electron microscopy (HRTEM) images and corresponding fast Fourier transform (FFT) patterns (Fig. 1h and Supplementary Figs. 15, 16) uncover an amorphous reconstructed surface layer with approximately 10 nm thickness in SNC-LSF, demonstrating long-range disorder in this reconstructed region. The surface amorphization was also confirmed by the decrease in peak intensity of powder X-ray diffraction (XRD) pattern in for SNC-LSF compared to LSF (Supplementary Fig. 17), with similar peak attenuation also observed for SNC-LCF and SNC-LBF relative to their corresponding pristine phases (Supplementary Fig. 18).

Subsequently, we carried out soft X-ray absorption spectroscopy (sXAS) and X-ray near-edge absorption fine structure (XANES) to study the evolution of surface electronic structure in SNC-LSF. In Fig. 1i, a weakened *t*_2g_ peak (~ 528 eV) and a blue-shifted *e*_g_ peak (~ 530.5 eV) in O *K*-edge spectra of SNC-LSF compared with pristine LSF reflect the valence reduction and ligand field distortion of Fe, which are likely resulted from the changes in O 2*p*-Fe 3 *d* hybridization during electrochemical reduction-assisted Sr^2+^ leaching^41,42^. A negative shift of the absorption edge in Fe *K*-edge XANES spectra was observed for SNC-LSF relative to LSF (Supplementary Fig. 19), confirming the valence reduction of Fe as well^43–45^. Besides, the local structures of surface remained Fe and La ions after electrochemical reduction-assisted selective Sr^2+^ leaching were investigated by extended X-ray absorption fine structure spectroscopy (EXAFS). The reduction in the intensity of the Fe-O scattering peak at ~ 1.50 Å in the *k*^3^-weighted Fourier-transformed EXAFS spectra of Fe *K*-edge (Fig. 1j) and the corresponding fitting results of Fe-O coordination number in SNC-LSF indicate the increase of undercoordinated Fe sites (Supplementary Fig. 20 and Supplementary Table 1). Moreover, both Fe-La/Sr (2.68 Å) and Fe-O-Fe (3.44 Å) distances in the *k*^3^-weighted Fourier-transformed EXAFS spectra of Fe *K*-edge were shorter in SNC-LSF than LSF, assigned to the lattice-disorder-induced formation of edge-sharing FeO_6_ octahedra^46^. A weakened La-Fe scattering peaks in the *k*^3^-weighted Fourier-transformed EXAFS spectra of La *L*-edge also evidence the local disorder of SNC-LSF induced by selective Sr²^+^ dissolution (Fig. 1k).

Raman spectroscopy and Fourier-transform infrared spectroscopy (FTIR) were further carried out to confirm the enrichment of hydroxyl groups after electrochemical reduction-assisted selective Sr^2+^ leaching (Fig. 1l and Supplementary Fig. 21). Compared with pristine LSF with a centrosymmetric structure, SNC-LSF displays emerging vibrational bands of Fe-O and Fe-OH stretching modes at ~ 211.3, ~273.0, ~389.9 and ~600.9 cm^−1^ in Raman spectra, suggesting local structure disorder and hydroxyl enrichment^47^. In FTIR spectra, distinct absorption peaks of La-OH and Fe-OH vibrations at 1058 and 1124 cm^−1^ were observed as well^47,48^. To firmly verify the formation of La-OH and Fe-OH species on SNC-LSF, FTIR analysis was conducted on SNC-LSF(D) that is electrochemically activated in D_2_O-containing electrolyte. We obviously detected new peaks at 703 cm^−1^ and 880 cm^−1^, which are attributed to La-OD and Fe-OD stretching modes, respectively. Such a distinct vibrational shift between them aligns with the expected isotopic shift ratio (ν(O-H)/ν(O-D) = 1/√2)^49,50^. Meanwhile, characteristic peaks of surface OH^-^ species with a well-resolved resonance at − 1.7 ppm were observed on SNC-LSF in the ^1^H magic-angle spinning nuclear magnetic resonance (MAS-NMR) spectra (Supplementary Fig. 22), also supporting the appearance of surface OH species^51^. Collectively, on the basis of the above results, hydroxyl-rich nano-concavities with undercoordinated Fe catalytic sites on the reconstructed surface of SNC-LSF through electrochemical reduction-assisted selective Sr^2+^ leaching were successfully constructed.

### Nitrate adsorption and hydrogen transfer behaviors on SNC-LSF

In situ Raman spectroscopy was initially employed to investigate the NITRR process on the surface of SNC-LSF (Supplementary Fig. 23). As a comparison, pristine LSF and LF without surface nanocavities were tested under identical conditions.

**Fig. 2:**
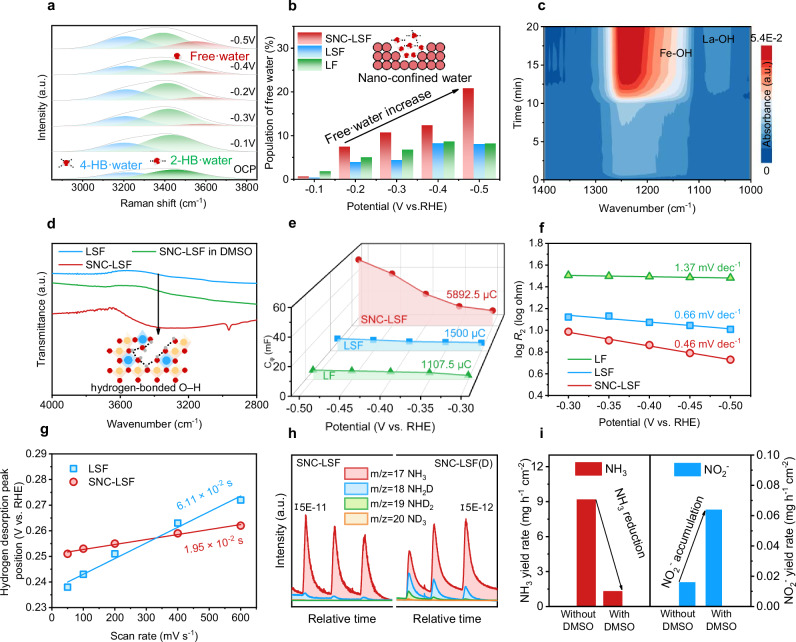
Interfacial nitrate adsorption and hydrogen transfer behaviors on SNC-LSF (non iR-corrected). **a** In situ Raman spectra of SNC-LSF. **b** Potential-dependent relative proportions of interfacial water over LSF and SNC-LSF. **c** Time-resolved in situ ATR-SEIRAS spectra collected on LSF at − 0.6 V vs. RHE. **d** FTIR spectra of LSF, SNC-LSF and SNC-LSF treated by DMSO. **e** Hydrogen adsorption charge (Q_H_) and (**f**) Tafel plots of the LF, LSF and SNC-LSF. **g** Plots of hydrogen desorption peak position vs. scan rates of the LF and SNC-LSF. **h** DEMS measurements of NITRR over SNC-LSF and SNC-LSF(D). **i** NH_3_ and NO_2_^-^ yield rate of SNC-LSF in 1 M KOH + 0.1 M KNO_3_ with and without DMSO at − 0.4 V vs. RHE. Source data are provided as a Source Data file.

The most prominent peak of adsorbed NO_3_^-^ (^*^NO_3_) at 1003 cm^−1^ for SNC-LSF was observed among all catalysts, indicating the strengthened adsorption ability of NO_3_^-^ on SNC-LSF relative to LSF and LF^52,53^. Such a result could be attributed to fully exposed Fe and La sites with unsaturated coordination in the amorphous surface nanocavity-confined hydrogenation reactor of SNC-LSF. For NITRR, beyond nitrate adsorption, sufficient hydrogen supply for subsequent ^*^NO_3_ hydrogenation to NH_3_ is equally critical^54^. In alkaline electrolyte, the interfacial water adsorbed on the catalyst surface serves as a hydrogen source for ^*^NO_3_ hydrogenation, which plays a crucial role in producing ^*^H and modulating the selectivity of NH_3_. To further clarify the effect of confined interfacial water on the NITRR performance of SNC-LSF, we performed Gaussian fitting on the broad Raman band of the O-H stretching mode (ν_O-H_) of interface water (Fig. 2a and Supplementary Fig. 24). Among three types of interfacial water, free ion hydrated water (free-H_2_O) without hydrogen bond, has been widely recognized as the most dissociated water on the catalyst surface for supplying ^*^H^55^. As the potential decreases from -0.1 to -0.5 V vs. RHE during NITRR, the proportion of free-H_2_O on SNC-LSF increases the most from 0.62 to 20.85% among all three catalysts, revealing the strongest water dissociation and ^*^H generation ability of SNC-LSF (Fig. 2b). In situ attenuated total reflectance surface-enhanced infrared absorption spectroscopy (ATR-SEIRAS) was also conducted to disclose the possible hydrogen transfer path on the surface of SNC-LSF during NITRR. As shown in Fig. 2c, a new peak of the Fe-OH vibrational frequency at 1124 cm^−1^ immediately appears for LSF once cathodic potential was applied, possibly resulting from the hydroxylation of coordinatively unsaturated Fe on LSF in alkaline conditions. With the increase of activation time, the progressive enhancement of its peak intensity was observed, which could be understood that the electrochemically generated nanocavity-confined hydrogenation reactor with local structure disorder on the rough surface further expose more coordinatively unsaturated Fe to form Fe-OH species. Meanwhile, a new peak at 1058 cm^−1^ corresponding to the La-OH vibrational frequency emerges after 10 min NITRR. It is speculated that, different from Fe-OH, the formation of La-OH in SNC-LSF only occurs when sufficient ion exchange between proton and Sr ions has taken place during the electrochemical activation process. In contrast, no obvious Fe-OH or La-OH species was detected in LF because of the absence of both coordinatively unsaturated Fe sites and Sr^2+^-leaching-induced surface nanocavity-confined hydrogenation reactor (Supplementary Fig. 25). In the nanocavity-confined hydrogenation reactor with such enriched hydroxyls on SNC-LSF, adjacent hydroxyls readily establish intramolecular hydrogen bonds, and the self-assembled hydrogen bond network is desired for favorable hydrogen transport during NITRR^30–34^. As expected, we observed a broad O-H stretching band in the range of 3000–3700 cm^-1^ in the FTIR spectrum on SNC-LSF (Fig. 2d), especially presenting the characteristic absorption centered at ~ 3411 cm^−1^ assigned to hydrogen-bonded O-H^56^, which verifies the intramolecular hydrogen-bond networks formed on SNC-LSF. Such unusual intramolecular hydrogen-bond networks also appear on the walls of nanocavity-confined reactors formed by the selective dissolution of other alkaline earth metals in SNC-LCF and SNC-LBF (Supplementary Fig. 26), verifying the universality of this strategy in constructing hydroxyl-functionalized hydrogenation nanoreactors.

Electrochemical impedance spectroscopy (EIS) and cyclic voltammetry (CV) were further performed to study the impact of the constructed intramolecular hydrogen-bond network on ^*^H transfer. It was found that the hydrogen adsorption charge (Q_H_) reflecting the catalysts’ adsorption capacity of ^*^H derived from EIS measurements (Supplementary Fig. 27 and Supplementary Table 2), follows the order of LF (1107.5 μC) < LSF (1500 μC) < SNC-LSF (5892.5 μC) (Fig. 2e), suggesting that SNC-LSF possesses the highest ^*^H adsorption amount. Lower Tafel slope of SNC-LSF (0.46 mV dec^-1^) relative to LSF (0.66 mV dec^-1^) and LF (1.37 mV dec^-1^) also confirms the improved ^*^H adsorption and transfer ability of SNC-LSF (Fig. 2f). Furthermore, we explored the ^*^H desorption kinetics via conducting the CV tests at varied scan rates and found that SNC-LSF possess smaller slope (1.95 × 10^−2 ^s) of the ^*^H desorption peak shift than pristine LSF (6.11 × 10^−2 ^s). LF displays no ^*^H desorption peak because of its insufficient adsorbed ^*^H content (Fig. 2g and Supplementary Fig. 28). In addition, temperature-dependent EIS measurement was performed to investigate the hydrogen transfer capacity of the intramolecular hydrogen bonds on SNC-LSF (Supplementary Fig. 29). Fitted Arrhenius plots indicate that the hydrogen transfer activation energy (E_a_) of 0.22 eV on SNC-LSF is below the empirical threshold of 0.4 eV, suggesting that hydrogen transport on SNC-LSF is predominantly governed by the Grotthuss-like mechanism^57^. These analyses jointly demonstrate that the ^*^H adsorption and desorption process during ^*^H transfer are significantly boosted along the intramolecular hydrogen-bond network on the nanocavity-confined hydrogenation reactor of SNC-LSF.

To solidly prove the hydrogen transfer mechanism through the intramolecular hydrogen-bond network on SNC-LSF, we used isotopic labeling combined with on-line differential electrochemical mass spectrometry (DEMS) as well and monitored the real-time NITRR products over SNC-LSF and SNC-LSF(D) in 1 M KOH + 0.1 M KNO_3_ (Fig. 2h). For SNC-LSF that was pre-hydroxylated in KOH/H_2_O through electrochemical reduction at − 0.6 V vs. RHE for 10 minutes, the main product corresponds to m/z = 17 (NH_3_) with only trace m/z = 18 (NH_2_D) due to the residual deuterium in H_2_O^58^. In contrast, for SNC-LSF(D) that was pre-hydroxylated in NaOD/D_2_O, the first cycle produces simultaneous signals of m/z = 17 (NH_3_), 18 (NH_2_D), 19 (NHD_2_), and 20 (ND_3_), confirming that deuterons of Fe-OD or La-OD in SNC-LSF(D) are incorporated into hydrogenation intermediates. In subsequent cycles, NH_3_ yields increase, but deuterated ammonia amounts decrease. Such an interesting phenomenon affirms the hydrogen migration along the intramolecular hydrogen-bond network on SNC-LSF, where free H_2_O dissociates to generate ^*^H and form H-O on the lattice oxygen of surface hydroxyls, as well as hydrogen existed initially in surface hydroxyls dissociates and transfers stepwise along the hydrogen-bonded network to the NO_3_^-^-adsorbed active sites for NO_3_^-^ hydrogenation. This process resembles the Grotthuss mechanism^57,59^, in which the O-H bond cleavage and formation along the intramolecular hydrogen-bond network on the nanocavity-confined hydrogenation reactor of SNC-LSF mediate hydrogen transport. Additionally, the H/D kinetic isotope effect (KIE) was re-evaluated using the NH_3_ yield rate and Faradaic efficiency. Upon deuteration, the NH_3_ yield rate and Faradaic efficiency decrease while NO_2_^-^ production increase, with this trend becoming more pronounced for pre-deuterated SNC-LSF(D). These results indicate that hydrogen transfer is involved in the rate-determining steps and support a hydrogen-bond-mediated hydrogen transfer pathway during NITRR. (Supplementary Fig. 30)^60,61^. We further introduced dimethyl sulfoxide (DMSO) to selectively disrupt the intramolecular hydrogen bonds on SNC-LSF, which is confirmed by the disappearance of the characteristic broad band of intramolecular hydrogen bonds from 3000 cm^−1^ to 3700 cm^−1^ in FTIR spectra (Fig. 2d)^62^. Importantly, complementary FTIR and ESR analyses demonstrate that DMSO does not form stable coordination bonds with the metal centers nor quench hydrogen radical (^*^H) species under our experimental conditions (Supplementary Figs. 31, 32). It was found the selectively break of the intramolecular hydrogen-bond network by DMSO or selectively replacing surface hydroxyl groups with F^-^ using NaF treatment (denoted as SNC-LSF(F))^62,63^, significantly reduces NH_3_ yields and accumulated NO_2_^-^ intermediates (Fig. 2i and Supplementary Fig. 33), which is due to the inability of the impaired hydrogen-bond network to transport hydrogen for NO_3_^-^ hydrogenation that consequently results in the reaction stagnation at NO_2_^-^ intermediates. Therefore, benefiting from the intramolecular hydrogen-bond network on the hydroxyl-rich nanocavity of SNC-LSF, the produced ^*^H could be timely transferred and utilized during NITRR, as confirmed by the sharpest DMPO-H signal attenuation among all catalysts in electron spin resonance (ESR) measurements using DMPO as a spin-trapping probe (Supplementary Fig. 34).

### Insights into the NITRR mechanism on SNC-LSF

**Fig. 3:**
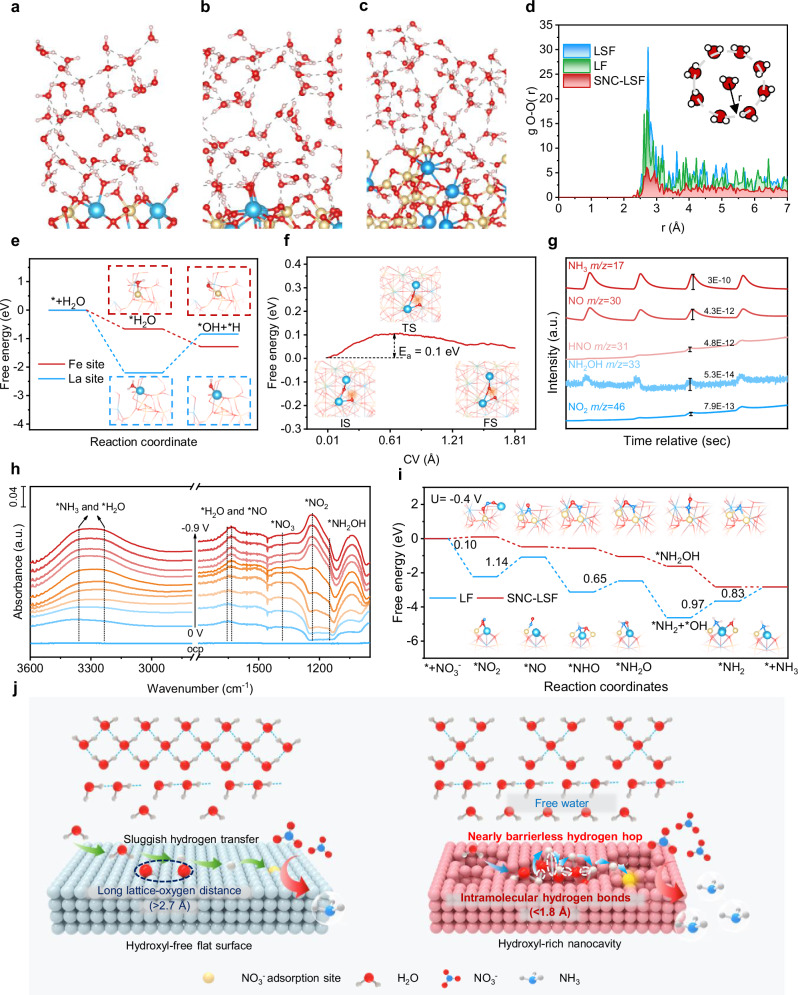
Insights into the NITRR mechanism on SNC-LSF. Representative snapshots of the interfacial H_2_O structures for (**a**) LF, (**b**) LSF and (**c**) SNC-LSF. The La, Sr, Fe, O and H atoms are colored in blue, green, yellow, red, and pink, respectively. The gray dashed lines represent the H bonds. **d** RDF of H_2_O molecules on the LF, LSF and SNC-LSF. **e** Free energy of H_2_O dissociation at different active sites of SNC-LSF. **f** Energy profiles of hydrogen transfer through the intramolecular hydrogen-bond network with the change of initial, transition and final state structures. **g** DEMS measurements of NITRR over SNC-LSF. **h** In situ ATR-SEIRAS spectra collected on SNC-LSF under the potentials from OCP to − 0.9 V vs. RHE (**non iR-corrected)**. **i** Calculated free energy diagrams for NITRR on LF and SNC-LSF at U = − 0.4 V vs. RHE. **j** Schematic illustrations of a hydroxyl-free flat surface and Nanocavity-confined hydrogenation reactor for NITRR. Source data are provided as a Source Data file.

To experimentally identify the catalytically active sites of SNC-LSF, selective poisoning experiments were carried out using KSCN as a probe molecule for Fe centers^64^. As shown in Supplementary Fig. 35, the introduction of SCN^-^ causes a pronounced suppression of the catalytic current, decreasing it to values even lower than those associated with the hydrogen evolution reaction, indicative of effective deactivation of Fe sites. Consistent with this observation, electron spin resonance (ESR) measurements using DMPO as a spin-trapping agent reveal a substantial attenuation of the DMPO-H signal upon SCN^-^ addition, confirming that the formation of ^*^H on Fe sites is strongly inhibited. Correspondingly, subsequent NITRR measurements exhibit a marked decline in both ammonia production rate and Faradaic efficiency. Collectively, these results provide compelling experimental evidence that *Fe centers serve as the primary active sites for H generation and nitrate-to-ammonia conversion on SNC-LSF. In addition, ab initio molecular dynamics (AIMD) simulations in combination with density functional theory (DFT) calculations were further adopted to elucidate the molecular-level interfacial microenvironment and dynamic hydrogen transfer pathways on SNC-LSF. To begin with, AIMD simulations were performed to examine how nanocavity-confined hydrogenation reactor on SNC-LSF influences the interfacial water structure. As illustrated in Fig. 3a–c, the spatial distribution of H_2_O molecules in representative snapshots of the interfacial H_2_O structures on the surface reveals that the nanocavity-confined hydrogenation reactor on SNC-LSF significantly reduces the gap between water molecules and the surface of SNC-LSF as compared to both LF and LSF with flat surfaces (Supplementary Data 2). Moreover, the oxygen radial distribution function (RDF) analysis shows that SNC-LSF possesses the lowest peak probability density of water molecules at 0.276 nm (Fig. 3d), indicating a reduced density of hydrogen-bonded water in the first hydration layer^65^. This observation is in good agreement with the in situ Raman spectra analysis as discussed above. Subsequently, DFT calculations were conducted to investigate the ^*^H generation via H_2_O dissociation (Supplementary Data 1). Notably, interfacial H_2_O adsorption and dissociation steps became energetically downhill on Fe sites on SNC-LSF compared with LF (Fig. 3e and Supplementary Fig. 36), implying that the readily water cleavage on SNC-LSF is favored to produce ^*^H. Following water dissociation, the generated ^*^H species should be rapidly delivered to ^*^NO_3_ adsorption sites for high-efficiency NITRR electrocatalysts to drive hydrogenation and suppress the competing hydrogen evolution reaction (HER). This preference originates from the confined hydrogen-bond network within the hydroxyl-rich nanocavities, which enables directional hydrogen transfer toward nitrate intermediates, together with the non-ideal hydrogen adsorption energies that deviate from thermoneutral conditions (ΔG_H*_ ≈ 0 eV) for HER, thereby disfavoring H_2_ evolution (Supplementary Fig. 37). Thus, slow-growth AIMD simulations were then carried out to probe the hydrogen transfer pathway mediated by intramolecular hydrogen bond over nanocavity-confined hydrogenation reactor of SNC-LSF (Supplementary data 3)^57^. In this pathway, hydrogen hops along the hydrogen-bond network formed between hydroxyl groups within the SNC-LSF nanocavity-confined hydrogenation reactor. Specifically, free H_2_O molecules dissociate to produce ^*^H, which subsequently bonds to lattice oxygen atoms of surface hydroxyls on SNC-LSF. The hydrogen atoms in these surface hydroxyls then dissociate and migrate stepwise through the intramolecular hydrogen-bond network toward ^*^NO_3_ adsorbed active sites on SNC-LSF for selective hydrogenation of ^*^NO_3_ to NH_3_. This hydrogen transfer pathway closely resembles the Grotthuss mechanism, wherein O-H bonds are continuously cleaved and reformed along the hydrogen-bond network to realize hydrogen transport^32^. Notably, the calculated energy barrier for this confined hydrogen transfer process is only 0.10 eV (Fig. 3f), in line with the experimentally observed low activation energy from temperature-dependent EIS measurements, significantly lower than that (0.40 eV) of the conventional Grotthuss mechanism in bulk water^57^. In contrast, on the surface of hydroxyl-free LF, the hydrogen transfer pathway is impeded by the relatively large distance between lattice oxygen sites (> 2.70 Å), underscoring the pivotal role of intramolecular hydrogen bonds between adjacent surface hydroxyls with a distance of 1.53 ~ 1.88 Å in enabling efficient hydrogen migration on SNC-LSF.

To gain mechanistic insights into NO_3_^-^ reduction, we also investigated the NITRR pathways on LF and SNC-LSF. We first used electrochemical differential electrochemical mass spectrometry (DEMS) to detect and identify key intermediates and products. As shown in Fig. 3g, signals at m/z = 46 (NO_2_^-^), 30 (NO), 31 (HNO), 33 (NH_2_OH), and 17 (NH_3_) were detected for SNC-LSF, whereas signals except for m/z = 33 (NH_2_OH) was observed on LF (Supplementary Fig. 38). Besides DEMS, In situ attenuated total reflectance surface-enhanced infrared absorption spectroscopy (ATR-SEIRAS) measurements were conducted to reveal the evolution of these surface-bound intermediates. Upon applying increasingly negative potentials, distinct absorptionbands appear for both LF and SNC-LSF (Fig. 3h and Supplementary Fig. 39). For instance, the evolving peak at 1382 cm^-1^, corresponding to the symmetric stretching of ^*^NO_3_, initially intensifies then diminishes, reflecting nitrate adsorption followed by its reduction^65,66^. Notably, two bands at 1236and 1656 cm^−1^, related to the antisymmetric stretching of ^*^NO_2_ and ^*^NO, respectively, show more pronounced peak intensity enhancement relative to LF, suggesting more favorable NO_3_^-^ and NO_2_^-^ deoxygenation ability on the adequately exposed catalytic sites of SNC-LSF. Moreover, a more enhanced peak of the bending mode of adsorbed ^*^H_2_O at 1638 cm^−1^ on SNC-LSF indicates the H_2_O enrichment of due to the superiority of space-confined nanocavity-confined hydrogenation reactor in reconstructing interfacial H_2_O and hydrogen bond networks on SNC-LSF^52^. In consistent with DEMS results, we also observed the characteristic band of the N-O bond of hydroxylamine (NH_2_OH) at ~ 1145 cm^−1^ only on SNC-LSF. Benefiting from the excellent NO_3_^-^ adsorption, activation and hydrogenation ability of SNC-LSF, a significantly stronger positive band at ~ 3200–3380 cm^−1^, associated with the NH stretching of solution-phase NH_3_, was found for SNC-LSF than LF^52,67^. Based on combined DEMS and ATR-SEIRAS analysis, we constructed the free energy profiles of respective intermediates on LF and SNC-LSF (Supplementary data 1) at − 0.4 V vs. RHE (Fig. 3i). Before calculating the energy path, the optimal NO_3_^-^ adsorption site is determined by calculating the adsorption energies of ^*^NO_3_ on five possible sites (Supplementary Fig. 40). The results imply that the ^*^NO_3_ adsorption and activation are favored on site 2 with the side-on adsorption configuration on the oxygen-undercoordinated La and Fe sites. For LF, the reaction steps involve several energetically demanding transitions, including ^*^NO_2_ → ^*^NO, ^*^NHO → ^*^NH_2_O, ^*^NH_2_ + ^*^OH → ^*^NH_2_ and ^*^NH_2_ → ^*^+NH_3_, with the highest energy barrier reaching 1.14 eV. Besides, the strong binding of ^*^NH_3_ on LF impedes its desorption, further limiting catalytic efficiency. In contrast, the introduction of the nanocavity-confined hydrogenation environment reactors in SNC-LSF could significantly modulate the reaction landscape. The calculated free energy profile indicates that the initial step (^*^+ NO_3_^-^ → ^*^NO_2_) is the only mildly endothermic process, with a low energy barrier of approximately 0.10 eV, suggesting that nitrate adsorption and activation is favorable. Subsequent hydrogenation steps are energetically downhill, reflecting the facile hydrogenation of reaction intermediates within the hydroxyl-rich nanocavity. Notably, the final ^*^NH_3_ desorption step is predicted to be thermodynamically barrierless (0 eV), which facilitates efficient product release and rapid regeneration of active sites. Overall, combined experimental results and theoretical calculations confirm that, compared with hydroxyl-free flat surfaces, the nanocavity-confined hydrogenation reactors significantly promote the generation, transfer, and utilization of ^*^H through a unique hydrogen hopping effect. A schematic illustrated in Fig. 3j, surface nanocavity-confined hydrogenation reactors disrupt the rigidity of interfacial water, thereby facilitating water dissociation to produce ^*^H. The generated ^*^H is then rapidly delivered to ^*^NO_3_^-^ adsorption sites via an intramolecular hydrogen-bonding network formed on the hydroxyl-rich walls of nanoreactors, subsequently boosting NO_3_^-^ hydrogenation steps.

### Electrocatalytic NITRR performance

**Fig. 4:**
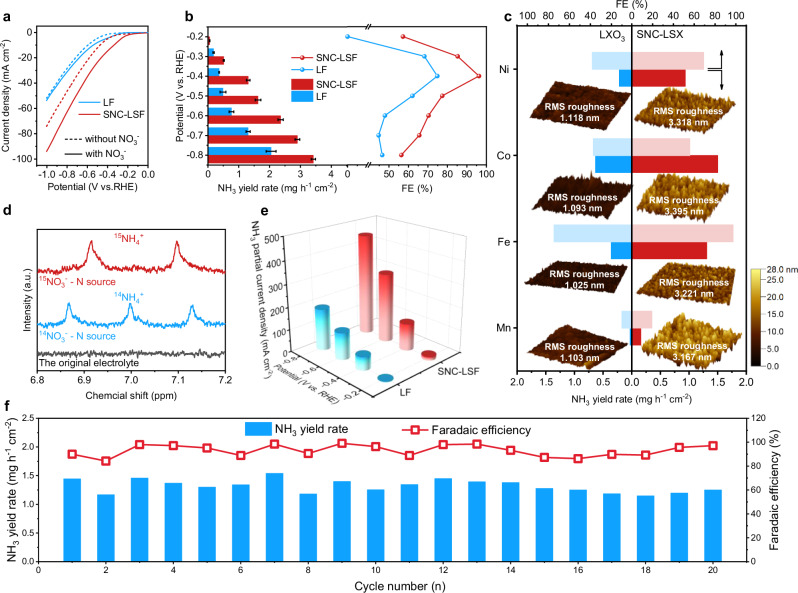
Electrocatalytic NITRR performances (non iR-corrected). **a** LSV polarization curves of LF and SNC-LSF in 0.1 M KOH with and without 8 mM KNO_3_. **b** NH_3_ yield rate and FE_NH3_ of LF and SNC-LSF in 0.1 M KOH with 8 mM KNO_3_ at various potentials. **c** NH_3_ yield rate and FE_NH3_ of LXO_3_ and SNC-LSX (X = Mn, Fe, Co or Ni) in 0.1 M KOH with 8 mM KNO_3_ at − 0.4 V vs. RHE (inset are AFM maps and root mean square roughness of LXO_3_ and SNC-LSX). **d** NMR spectrum of the products generated during the electrocatalytic NITRR on the SNC-LSF at  −0.4 V vs. RHE. **e** Partial NH_3_ current densities of LF and SNC-LSF normalized to the geometric area in 1 M KOH with 0.1 M KNO_3_ at various potentials. **f** NH_3_ yield rate and Faradaic efficiency of SNC-LSF in 0.1 M KOH with 8 mM KNO_3_ under the applied potential of at − 0.4 V vs. RHE during 20 consecutive electrolysis cycles. Source data are provided as a Source Data file.

The aforementioned experimental and theoretical results have fully validated the potential of the SNC-LSF electrode for selective ammonia production via nitrate reduction. Therefore, we systematically evaluated its performance toward NITRR, including activity, selectivity, and stability. The catalytic performance of SNC-LSF electrocatalysts toward NITRR was evaluated using a standard three-electrode configuration in an H-type cell. As a comparison, the counterpart LF without surface nano-concavities was tested under the same conditions. As displayed in linear sweep voltammetry (LSV) curves in Fig. 4a and Supplementary Fig. 41, SNC-LSF exhibits considerably increased current density in the presence of NO_3_^-^ compared to the NO_3_^-^-free electrolyte among all catalysts, implying a higher nitrate reduction activity of SNC-LSF. To further quantify the Faradaic efficiency (FE_NH3_) and ammonia yield rate, ultraviolet-visible (UV-Vis) spectrophotometry was performed after 1-hour electrolysis at selected potentials (Supplementary Fig. 42). As can been seen, among all catalysts, SNC-LSF exhibits the highest FE_NH3_ and ammonia yield rate (Fig. 4b and Supplementary Fig. 4) within the selected potential window. To rule out the potential background contributions of carbon black, its NITRR activity was measured, which exhibits a negligible NITRR activity (Supplementary Fig. 43). Remarkably, SNC-LSF achieves an impressive NH_3_ yield of 1.31 mg h^−1^ cm^−2^ and a Faradaic efficiency of 96.19 % at − 0.4 V vs. RHE, which were also verified by the results obtained from ^1^H nuclear magnetic resonance (NMR) measurements (Supplementary Figs. 44, 45). Besides, it should be noted that the pristine Sr-containing LSF displays a gradual increase in ammonia production during NITRR, suggesting the enhancement effect of the nanocavity-confined hydrogenation reactor on the NITRR due to in situ electrochemical-reduction-induced selective Sr^2+^ leaching. However, Sr-free LF exhibits nearly unchanged activity throughout the 1-hour testing (Supplementary Fig. 46). At the same time, similar enhancements in activity were also observed in other activated Sr-containing perovskites with B-sites occupied by Co, Ni and Mn transition metals, namely surface nano-caved La_0.4_Sr_0.6_CoO_3-δ_ (SNC-LSC), surface nano-caved La_0.4_Sr_0.6_NiO_3-δ_ (SNC-LSN) and surface nano-caved La_0.4_Sr_0.6_MnO_3-δ_ (SNC-LSM), further confirming the broad applicability of this method (Fig. 4c and Supplementary Figs. 47–51). Notably, AFM analyses reveal that the surface roughness of all these samples increases significantly after Sr leaching, with RMS roughness increasing from 1.025-1.118 nm for the pristine samples to 3.167–3.395 nm after activation, confirming the formation of nanocavity features. More importantly, SNC-LSF shows a remarkable superiority in electron utilization efficiency over SNC-LSC, SNC-LSN and SNC-LSM. In light of variable nitrate concentrations in practical wastewater sources, the NITRR performance of SNC-LSF was further evaluated under a range of NO_3_^-^ concentrations, including 8, 16, 32, 100 and 1000 mM. As show in Supplementary Figs. 52, 53, SNC-LSF delivers a FE_NH3_ above 90% under all range of NO_3_^-^ concentrations. In particular, a remarkable FE_NH3_ of 97.81% and a higher production rate of 51.37 mg h^−1^ cm^−2^ were obtained on SNC-LSF at a higher NO_3_^-^ concentration of 1000 mM. Such activities of SNC-LSF ranking among superior NITRR electrocatalysts under both low and high NO_3_^-^ concentrations as reported (Supplementary Fig. 54 and Supplementary Table 3). We further evaluate the NITRR activity of the SNC-LSF and nanocavity-free LF at the different pH electrolyte. As expected in Supplementary Fig. 55, SNC-LSF consistently exhibits higher ammonia production rates and Faradaic efficiencies than LF, demonstrating its superior catalytic performance over a broad pH range. These results indicate that the intramolecular hydrogen transfer in the hydroxyl-rich nanocavity mechanism remains effective under neutral, mildly alkaline, and strongly alkaline conditions.

The contributions of other possible products to the faradaic current were also evaluated using gas chromatography equipped with a thermal conductivity detector and UV-vis spectrometry (Supplementary Figs. 56, 57). Only H_2_ and NO_2_^-^ were detected, and their FEs at − 0.4 V vs. RHE were calculated to be 2.08 % and 0.88 %, respectively. After taking the side-reactions into consideration, the total FE was close to 100 % as expected (Supplementary Fig. 58). Moreover, the NH_3_ selectivity of the SNC-LSF electrode is determined to be 99.10 % at − 0.4 V vs. RHE, further confirming its high selectivity towards NH_3_ production from NITRR. To further verify the nitrogen source of the generated NH_3_, isotope-labeling experiments were carried out using ^15^N-labeled NO_3_^-^ as the nitrogen precursor. Unlike ^14^NH_4_^+^, which displays three peaks in the ^1^H NMR spectrum, only two characteristic peaks of ^15^NH_4_^+^ were observed when ^15^NO_3_^-^ was used (Fig. 4d). This result undoubtedly confirms that the ammonia production is originated from the electroreduction of nitrate rather than from contaminants. Also, no ^15^NH_4_^+^ signals were detected in the ^1^H NMR spectra of the original electrolyte ^15^NO_3_^-^, ruling out the background contamination as the ammonia source as well. In addition, SNC-LSF exhibits a higher NH_3_ partial current density of 448.72 mA cm^−2^ at a more negative potential of − 0.8 V vs. RHE compared to LF (Fig. 4e), suggesting its superior performance and practical potential. We also assessed the intrinsic catalytic activity by calculating the specific activity (SA), which is defined as the current density normalized to the electrochemical surface area^68^ (ECSA, Supplementary Fig. 59). As seen from Supplementary Fig. 60, SNC-LSF shows a significantly higher SA than LF without surface nanocavities at − 0.4 V vs. RHE, highlighting the inherently high NITRR activity of SNC-LSF and the substantial contribution of nanocavity-confined hydrogenation reactor.

More importantly, both the FE_NH3_ and NH_3_ yield rate of SNC-LSF remains highly stable over 20 consecutive electrolysis cycles in the H-cell at − 0.4 V vs. RHE (Fig. 4f and Supplementary Fig. 61), demonstrating excellent durability and high potential for long-term practical applications. As shown in Supplementary Fig. 62–66, we further conducted ICP, XRD, AFM, XPS, and FTIR measurements to provide evidence to support the durability of the SNC-LSF electrode after stability tests. Notably, during the cathodic one-hour nitrate reduction electrolysis, the Sr concentration in the electrolyte increases only within the initial stage and becomes nearly constant after ~ 20 min. Compared to the 1000-s electrocatalyst activation in Fig. 1d, Sr dissolution during nitrate reduction electrocatalysis decreases by an order of magnitude, indicating that the formation of SNC-LSF is essentially completed prior to catalysis and the reconstructed structure remains stable thereafter. The post-reaction XRD pattern preserves the characteristic perovskite diffraction peaks with no detectable new crystalline phases upon air exposure. AFM images reveal a surface morphology similar to that of the pristine catalyst, with only a slight increase in roughness (4.079 nm). Moreover, FT-IR spectra confirm the persistence of La-OH/Fe-OH and intramolecular hydrogen-bond features, and XPS analyses show similar elemental compositions/oxidation states with only a minor enhancement of the hydroxyl-related O *1 s* component. Collectively, these results demonstrate that the electrochemically induced amorphous nanocavity structure is not a transient metastable phase but rather a stable reconstructed surface under both operating and post-exposure conditions, further substantiating the excellent structural and chemical stability of SNC-LSF during NITRR operation and the durability of the proposed intramolecular hydrogen-bond reaction mechanism.

### MEA industrial application of SNC-LSF

**Fig. 5:**
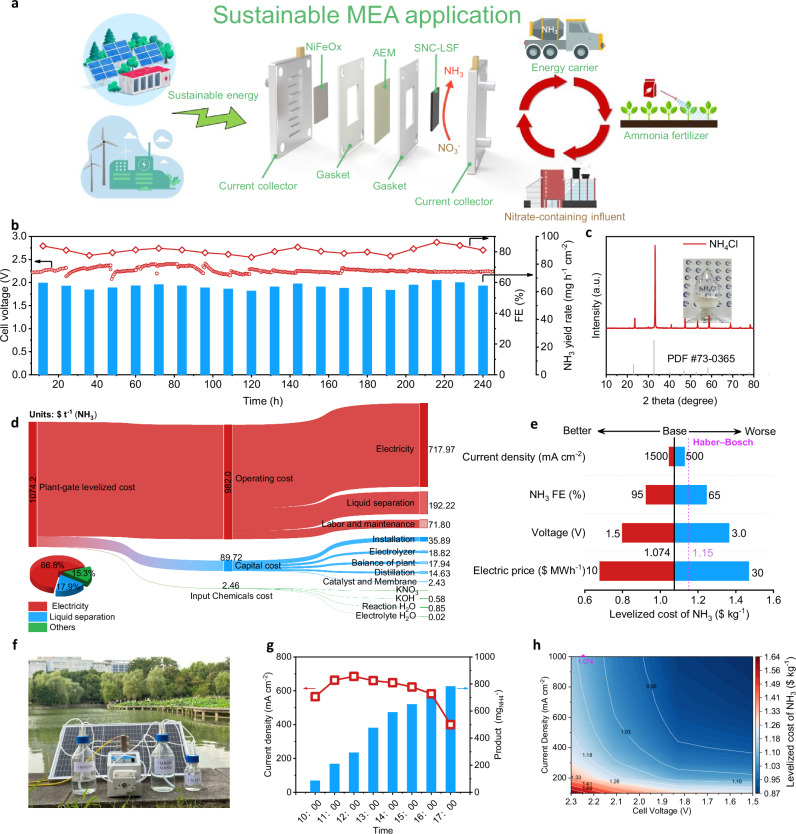
Practical NH_3_ product synthesis and analysis (non iR-corrected). **a** Schematic of an MEA cell for the NITRR. **b** Long-term stability test of the MEA system at 1 A cm^−2^, tracking NH_3_ FEs, yield rates, and the cell voltage using a SNC-LSF/CP cathode and a NiFeO_x_/Ni anode. **c** XRD pattern of the synthesized NH_4_Cl product (the inset is the photograph of the synthesized NH_4_Cl product). **d** Sankey diagram illustrating the breakdown of levelized NH_3_ production cost under benchmark conditions (2.23 V, 1000 mA cm^−2^). **e** Sensitivity analysis of levelized NH_3_ cost for operational and economic parameters. **f** Optical image of the solar-driven MEA electrolyzer. **g** The evolution of current density and recovered NH_4_^+^ under real sunlight at different times. **h** Contour plot of NH_3_ production cost as a function of cell voltage and current density. All other parameters are set to the baseline values in (**e**). Source data are provided as a Source Data file.

To demonstrate the industrial potential for the NITRR under real conditions, we assembled an NITRR-OER membrane electrode assembly (MEA) electrolyzer with SNC-LSF on carbon paper (SNC-LSF/CP) (2 × 2 cm^2^) as the cathode and commercial NiFeO_x_ on Nickel felt (NiFeO_x_/Ni) (2 × 2 cm^2^) as the anode (Fig. 5a). The SNC-LSF/CP || NiFeOx/Ni MEA electrolyze achieves an ampere-level current density at a low cell voltage of 2.23 V, which is competitive with most reported home-made devices (Supplementary Table 4). Using the MEA electrolyzer with SNC-LSF/CP as the cathode, NO_3_^-^ was converted to NH_3_ with a high NH_3_ FE up to 81.52 % and a yield rate of 32.32 mg h^−1^ cm^−2^ at 500 mA cm^−2^, demonstrating excellent device performance for industrial NH_3_ production (Supplementary Fig. 67). Besides, the durability was also explored and it was found that this MEA electrolyzer can maintain an average NH_3_ FE of ~ 80.69 %, an NH_3_ yield rate of ~ 63.92 mg h^−1^ cm^−2^ and a low voltage of 2.23 V at an industrial ampere-level current density for over 240 h (Fig. 5b). Minor voltage fluctuations in the durability tests could be the result of the electrolyte replenishment for compensating for nitrate depletion and maintaining consistent reaction conditions.

In addition, HRTEM images after the 240 hr operation (Supplementary Fig. 68) confirm that the amorphous surface layer is well-preserved with no observable recrystallization or structural collapse, demonstrating the robustness of the nanocavity-confined amorphous layer under prolonged industrial-level conditions. To further assess the potential impact of by-product accumulation and possible re-adsorption of leached Sr^2+^ ions, additional analyses were conducted. ICP monitoring of the electrolyte during long-term operation (Supplementary Fig. 69) shows that the Sr concentration increases initially (within 4 h) and then stabilizes, indicating that dissolved Sr^2+^ remains in solution without re-adsorption onto the catalyst surface, consistent with its high solubility in alkaline media and thermodynamic instability for re-deposition under cathodic conditions. Furthermore, the concentration of the primary intermediate NO_2_^-^ was monitored during the first 6 hours of electrolysis (Supplementary Fig. 70), and no noticeable accumulation was observed, confirming that intermediate species are continuously consumed. The absence of performance decay over 240 h further corroborates that neither Sr^2+^ re-adsorption nor by-product accumulation adversely affects the catalytic performance or stability. To confirm the practical potential of this process for NH_3_ production, a high-purity NH_3_ product was collected after NO_3_^-^ electroreduction using an acid trap method. Successful production of NH_4_Cl is confirmed by the XRD pattern (Fig. 5c).

Building on this promising performance, we assessed the commercial feasibility of nitrate-to-ammonia electrochemical synthesis through a detailed techno-economic analysis (TEA) based on experimentally validated parameters. As depicted in the Sankey diagram for the cost analysis of this process in Fig. 5d, the levelized NH_3_ production cost was demonstrated to be approximately US$1.074 kg^−1^ under standard operational conditions of 2.23 V and 1000 mA cm^−2^, dominated by the operating expenditures. We also found that especially electricity alone accounts for 66.8 % of total expenses. Consequently, if the electricity cost reduces from US$1.0 to US$0.1 per kWh in the future, the total cost of this NH_3_ production process is expected to dramatically decreases from US$4.23 to US$0.68 kg^−1^ (Supplementary Fig. 71), indicating application prospects of this process. Additional operational costs from the gas-liquid stripping process in the upstreaming NH_3_ recovery makes up 17.9 % of the total cost. Moreover, capital expenditures in stack materials and balance-of-plant infrastructure contribute minimally to the total cost due to the amortization of high product yield. We then compared the electrochemical approach with the conventional Haber-Bosch process in Fig. 5e and found that under the present experimentally validated baseline conditions, our method achieves a lower NH_3_ production cost (US$1.074 kg^−1^) than that of the Haber-Bosch benchmark process (US$1.15 kg^−1^), indicating the advantage in cost^69^. Furthermore, by lowing electricity prices (*e.g*., US$0.1 kWh^−1^) as discussed before or optimizing the operating conditions (*e.g*., 1.5 V), substantial economic advantages in the reduced levelized cost could be obtained, reflected by sensitivity analyses in Fig. 5e. Therefore, on one hand, we further adopted a solar cell to drive the MEA electrolyzer (Fig. 5f), employing SNC-LSF as the cathode, to access the feasibility of operating this process using affordable and sustainable electrical power. Notably, during prolonged solar illumination from 9:00 to 17:00 in the day, the yield of recovered NH_4_^+^ continued to increase, reaching as high as 800 mg. Throughout the 8-hour operation, the average Faradaic efficiency for NH_4_^+^ production remains at approximately 80 % (Fig. 5g). These findings demonstrate that the cost-effective MEA electrolysis powered by solar energy exhibits the technical feasibility. On the other hand, the detailed NH_3_ production cost sensitivity to cell voltage and current density was further illustrated in Fig. 5h. We found that, within an operating window at lower voltages (below 2.0 V) and moderate current densities (up to 500 mA cm^−2^), the process cost can be effectively maintained below that of the conventional Haber-Bosch process. Beyond this threshold, the efficiency gains of this process will diminish. Besides, converting NH_3_ into ammonium chloride (NH_4_Cl) via neutralization provides an alternative product route with enhanced market and transport advantages. As displayed in Supplementary Fig. 72, the simplicity of downstream processing potentially lowers the product cost to around US$0.368 per kg of NH_4_Cl, further enhancing its economic appeal. Therefore, this nitrate-to-ammonia electrochemical system in our work have been demonstrated both technical feasibility and economic viability under current operational conditions. When coupled with its environmental co-benefits in nitrate pollutant control, its compatibility with low-carbon electricity sources, and its flexibility in downstream product formats (NH_3_ or NH_4_Cl), this approach offers a compelling and modular pathway for decentralized ammonia production and sustainable nitrogen management.

Finally, industrial NO_3_^-^-wastewater was employed to evaluate the practical NO_3_^-^-to-NH_3_ performance of SNC-LSF. Despite the complex composition of the wastewater, including the presence of coexisting ions (*e.g*., Ca^2+^ and Mg^2+^), SNC-LSF exhibits efficient nitrate reduction under electrochemical operation (Supplementary Table 8). To further assess its robustness under realistic conditions, additional electrochemical measurements were conducted. As shown in Supplementary Fig. 73, LSV curves collected in industrial wastewater indicate a maintained catalytic activity in the presence of impurities. Moreover, repeated electrolysis tests (five consecutive cycles) were performed in the MEA system, demonstrating stable ammonia production with negligible decay in activity and selectivity. Notably, the total nitrogen concentration substantially reduces from 113.35 mg N L^−1^ to 2.56 mg N L^−1^, reaching the regulation levels in WTO (10 mg N L^−1^ for total nitrogen), while no detectable accumulation of the toxic intermediate NO_2_^-^ was observed within the detection limit. Collectively, these results confirm the strong tolerance of SNC-LSF toward coexisting ions and impurities, highlighting its robustness under realistic conditions and demonstrating its strong potential for the treatment and resource utilization of NO_3_^-^-containing wastewater.

## Discussion

In summary, we have developed a surface nano-concaved perovskite oxide SNC-LSF via electrochemical reduction-assisted selective Sr^2+^ leaching, which exhibits effective NITRR catalytic activity and durability. When evaluated under ambient conditions, SNC-LSF delivers a Faradaic efficiency of 97.81 % and an ammonia yield rate of 51.37 mg h^−1^ cm^−2^ at − 0.8 V vs. RHE, significantly outperforming its counterpart LF without surface nanocavities, pristine LSF and ranking among superior NITRR catalysts. Mechanistic investigations reveal that the excellent catalytic performance of SNC-LSF roots in the superiority of surface nanocavity-confined hydrogenation reactor; namely, undercoordinated La/Fe-O sites facilitate NO_3_^-^ adsorption and activation, space-confined architecture functions favorably for interfacial H_2_O reconstruction and activation, and hydroxyl-enriched walls with intramolecular hydrogen-bonding networks enable nearly-barrierless ^*^H transfer for NO_3_^-^ hydrogenation. The universal promotion effect of such nanocavity-confined hydrogenation reactors for NITRR is also found in other perovskites with varied compositions. Moreover, a renewable-energy-powered membrane electrode assembly electrolyzer using SNC-LSF as cathode achieves industrial-level nitrate conversion at a low voltage of 2.23 V during long-term operation and high-purity NH_3_ recovery, which provides a promising complement approach to the Haber-Bosch process by techno-economic analysis. This work not only establishes a highly efficient catalyst system for green ammonia production but elucidates a facile confined-hydroxyl-mediated intermolecular hydrogen transfer mechanism for electrocatalytic hydrogenation.

## Methods

### Chemicals

Lanthanum nitrate hexahydrate (Sinopharm Chemical Reagent Co., Ltd, La(NO_3_)_3_·6H_2_O, analytical reagent), strontium nitrate (Sinopharm Chemical Reagent Co., Ltd, Sr(NO_3_)_2_, analytical reagent), iron(III) nitrate nonahydrate (Aladdin Reagent (Shanghai) Co., Ltd, Fe(NO_3_)_3_·9H_2_O, analytical reagent), calcium nitrate (Sinopharm Chemical Reagent Co., Ltd, Ca(NO_3_)_2_, analytical reagent), barium nitrate (Sinopharm Chemical Reagent Co., Ltd, Ba(NO_3_)_2_, analytical reagent), ammonium molybdate (Sinopharm Chemical Reagent Co., Ltd, (NH_4_)Mo_2_O_7_, analytical reagent), ethylenediaminetetraacetic acid (Sinopharm Chemical Reagent Co., Ltd, C_1_₀H_16_N_2_O_8_, analytical reagent), citric acid monohydrate (Sinopharm Chemical Reagent Co., Ltd, C_6_H_8_O_7_·H_2_O, 99.5%), ammonia solution (Sinopharm Chemical Reagent Co., Ltd, NH_3_·H_2_O, 25.0-28.0%), potassium hydroxide (Aladdin Reagent (Shanghai) Co., Ltd, KOH, analytical reagent), sodium sulfate anhydrous (Sinopharm Chemical Reagent Co., Ltd, Na_2_SO_4_, analytical reagent), potassium nitrate (Sinopharm Chemical Reagent Co., Ltd, KNO_3_, analytical reagent), potassium sodium tartrate tetrahydrate (Sinopharm Chemical Reagent Co., Ltd, C_4_H_4_KNaO_6_·4H_2_O, 99%), Nessler reagent (Aladdin Reagent (Shanghai) Co., Ltd, Nessler Reagent, analytical reagent), ammonium chloride (Sinopharm Chemical Reagent Co., Ltd, NH_4_Cl, analytical reagent), sulfanilamide (Sinopharm Chemical Reagent Co., Ltd, C_6_H_8_N_2_O_2_S, 99.8%), N-(1-naphthyl)ethylenediamine dihydrochloride (Sinopharm Chemical Reagent Co., Ltd, C_12_H_14_N_2_·2HCl, 97%), phosphoric acid (Sinopharm Chemical Reagent Co., Ltd, H_3_PO_4_, 85%), argon (Nanjing Special Gas Factory Co., Ltd, Ar, 99.999%), ammonium fluoride (Aladdin Reagent (Shanghai) Co., Ltd, NH_4_F, analytical reagent), dimethyl sulfoxide (Sinopharm Chemical Reagent Co., Ltd, DMSO, analytical reagent), potassium nitrate (Sinopharm Chemical Reagent Co., Ltd, K^15^NO_3_, 98 atom% N) Nafion solution (Shanghai Geli New Energy Technology Co., Ltd, 5 wt%), hydrogen peroxide solution (Sinopharm Chemical Reagent Co., Ltd, H_2_O_2_, 30 wt%), 5,5-dimethyl-1-pyrroline N-oxide (Aladdin Reagent (Shanghai) Co., Ltd, DMPO, analytical reagent), hydrochloric acid (Sinopharm Chemical Reagent Co., Ltd, HCl, 36.0-38.0%), sulfuric acid (Sinopharm Chemical Reagent Co., Ltd, H_2_SO_4_, 98 wt%). potassium deuteroxide/deuterium oxide solution (Aladdin Reagent (Shanghai) Co., Ltd, KOD/D_2_O, 99.9 atom% D). All chemicals were used without further purification.

### Material synthesis

La_1−x_Sr_x_FeO_3-δ_ (LSF-x, where x = 0, 0.2, 0.4, 0.6 and 0.8) powders were prepared by following a synthetic procedure reported before^23^. In brief, 80 mL of ammonia solution dissolved with 0.1 mol of ethylenediaminetetraacetic acid was well-mixed under vigorous stirring into a solution containing a mixture of La(NO_3_)_3_· 6H_2_O (0.05 − 0.05 × x mol), Sr(NO_3_)_2_ (0.05 × x mol) and Fe(NO_3_)_3_· 9H_2_O (0.05 mol). Subsequently, citric acid (0.2 mol) was added into the above mixture. The resulting homogeneous mixture was heated at 90 °C with continuous stirring until a transparent gel was obtained, which was prefired at 250 °C to form a foamlike solid precursor. Finally, the solid precursors of LSF-x were further heated to 800 °C at a rate of 5 °C min^-1^ and maintained at 800 °C for 5 h.

### Preparation of SNC-LSF

The synthesized SNC-LSF was immersed in 50 mL of electrolyte (0.1 M KOH) for 3 ~ 5 min, and then activated at the applied negative potential of − 0.6 V vs. RHE for 1000 s.

### Cathode preparation

Approximately 6 mg of catalyst and 60 μL of a 5 wt% Nafion solution were dispersed in 940 μL of absolute ethyl alcohol. The mixture was sonicated to achieve a homogeneous ink. Subsequently, 90 μL of the prepared ink was dropped on a carbon paper electrode (CPE) with dimensions of 0.5 cm × 0.5 cm.

### Electrochemical measurements

Before the NITRR tests, the Nafion 117 membrane was pretreated as follows: it was first oxidized in 5% H_2_O_2_ solution at 80 °C for 1 h to remove organic impurities, then boiled in deionized (DI) water for 1 h to eliminate residual H_2_O_2_ and allow for expansion. Subsequently, the membrane was treated in 0.5 M H_2_SO_4_ at 80 °C for 1 h to remove metallic impurities and residual ammonia contaminants and to protonate the membrane. Finally, it was rinsed with DI water to remove excess acid and further expand the Nafion 117. This pretreatment procedure was repeated at least every three days to ensure membrane reusability.

The NITRR performance was evaluated on an electrochemical workstation (DH 7002 A) using a conventional three-electrode H-type cell. The system consists of the as-prepared catalyst electrode as the working electrode, a platinum electrode as the counter electrode and a Hg/HgO electrode as the reference electrode. The Nafion 117 membrane was fixed between the cathodic and anodic chambers, each containing 50 mL of 0.1 M KOH electrolyte with different concentrations of NO_3_^-^. All potentials were converted to the reversible hydrogen electrode (RHE) scale according to the all reported potentials were referenced to RHE. Prior to each measurement, the electrolyte was purged with Ar (99.999%) for 30 min. Linear sweep voltammetry (LSV) was performed at a scan rate of 10 mV s^−1^ under stirring at 500 rpm. Potentiostatic electrolysis was conducted at selected potentials within the NITRR window for 1 h.

### Determination of nitrate (NO_3_^−^)

Firstly, a certain amount of electrolyte was taken out from the electrolytic cell and diluted to 5 mL to the detection range. Then, 0.1 mL 1 M HCl and 0.01 mL 0.8 wt% sulfamic acid solution were added into the aforementioned solution. The absorption spectrum was measured using an ultraviolet-visible spectrophotometer, and the absorption intensities at a wavelength of 220 nm and 275 nm were recorded. The final absorbance value was calculated by this equation: A = A220 nm-2A275 nm. The concentration-absorbance curve was calibrated using a series of standard potassium nitrate solutions, and the potassium nitrate crystal was dried at 105–110 ^o^C for 2 h in advance.

### Determination of nitrite (NO_2_^-^)

The Griess test was used to determine the NO_2_^-^ concentration in electrolytes after chronoamperometry tests. Generally, N-1-naphthyl)ethylenediamine dihydrochloride (0.04 g), p-aminobenzenesulfonamide (0.8 g), and H_3_PO_4_ (2 mL, 85%) were dissolved in Dl water (10 mL) using the Griess reagent. The diluted electrolyte (20 times, 2 mL) was mixed with the Griess agent (40 μL) and rested for 10 min at room temperature (25 °C) to conduct UV-vis tests. NO_2_^-^ concentrations of the electrolyte were determined by the absorbance at 540 nm on the recorded UV-vis curves (400–650 nm). In the same operation, various NaNO_2_ aqueous solutions were used as the standard samples to obtain the calibration curve. The FE of byproduct i (FEi) was calculated via the following relation:

$${{{\rm{FE}}}}_{{{\rm{i}}}}=({{{\rm{n}}}}_{{{\rm{i}}}}\times {{\rm{F}}}\times {{{\rm{c}}}}_{{{\rm{i}}}}\times {{\rm{v}}})/(17\times {{\rm{Q}}})$$

Where n_i_ is the number of electrons transferred to by product i, c_i_ is the measured by product i concentration, V is the volume of the electrolyte or the upper space, F is the Faraday constant (96485 C mol^−1^), and Q is the total charge.

### Determination of ammonia (NH_3_)

Nessler’s reagent method was employed to quantify the generated NH_3_ in the electrolyte to further determine the Faradaic efficiency (FE_NH3_) and NH_3_ yield rate. In particular, a certain amount of electrolyte was taken out from the cell and diluted to the detection range. Then, an aqueous solution of potassium sodium tartrate (KNaC_4_H_6_O_6_, 100 μL, 500 g L^−1^) and Nessler’s reagent (100 μL) were added into the diluted electrolyte (5 mL) to obtain the uniform mixture. Let stand for 10 min. The UV-Vis absorption intensity at the absorbance peak at 420 nm was collected to determine the NH_3_ concentration of a series of standard ammonium chloride solutions and the mixed solution. The concentration-absorbance curve was plotted for the calculation of the NH_3_ concentration of the mixed solution.

Calculation of the NH_3_ yield rate, the Faradaic efficiency (FE):

NH_3_ yield rate was calculated using the following equation:

$${{\rm{NH}}}_3\,{{\rm{yield}}}\; {{\rm{rate}}}=[{{{\rm{NH}}}_{4}}^{+}]\times {{\rm{V}}}/({{\rm{S}}}.\,\times {{\rm{t}}})$$

FE was calculated according to the following equation:

$${{\rm{FE}}}=8\times {{\rm{F}}}\times [{{{\rm{NH}}}_{4}}^{+}]\times {{\rm{V}}}/(17\times {{\rm{Q}}})$$

Where [NH_4_^+^] is the measured NH_4_^+^ concentration, V is the volume of the cathodic reaction electrolyte, t is the potential applied time, S is the electrode area, F is the Faraday constant, and Q is the quantity of applied electricity.

### Isotope labeling experiments

K^15^NO_3_ was used as the feeding N-source to perform the isotopic labeling nitrate reduction experiments to clarify the source of ammonia. 0.1 M KOH was used as electrolyte, and 8 mM K^15^NO_3_ was added into the cathode compartment as the reactant. After electroreduction, the electrolyte with the obtained ^15^NH_4_^+^ was taken out, and the pH value was adjusted to be weak acid for further analysis by ^1^H NMR (300 MHz) with external standards of DMSO-d^6^.

### ^1^H nuclear magnetic resonance (^1^H NMR)

The produced ^14^NH_4_^+^ was quantified by ^1^H nuclear magnetic resonance spectroscopy. In a typical procedure, the standard ^14^NH_4_Cl solution with defined concentrations was first prepared, and the solution pH was adjusted to be weak acid. Next, 1 mL of the solution was mixed with 200 μL of DMSO-d^6^ as the internal standard. Finally, the resultant solution was tested on a Bruker 300 MHz AVANCE spectrometer. Noticeably, a standard curve was obtained by plotting the peak area ratio of ^14^NH_4_^+^ to DMSO-d^6^ with the series ^14^NH_4_^+^ concentrations, considering that the ^14^NH_4_^+^ concentration and the area ratio were positively correlated.

### Hydrogen adsorption based on EIS measurements

The Nyquist plots were acquired in the frequency range from 100 kHz to 1 Hz under different potentials (− 0.3 ~ −0.5 V vs RHE). The applied potential range was selected based on NITRR potential, as displayed in the liner cyclic voltammetry (LCV) curves (Fig. 3a). The Nyquist plots were simulated with the double-parallel equivalent circuit model (Supplementary Fig. 18) and fitting parameters were listed in Supplementary Table 2. Then, the hydrogen adsorption charge (Q_H_) was calculated with the following formula:

$${{\mbox{Q}}}_{{\mbox{H}}}={\int _{{{\mbox{U}}}_{1}}^{{{\mbox{U}}}_{2}}} {{\mbox{C}}}_{{\upphi}}$$

In which, C_φ_ refers to the hydrogen adsorption pseudo-capacitance; U_1_ and U_2_ refer to the initial and ultimate applied potentials (U_1_ = − 0.3 V, U_2_ = − 0.5 V). Besides, the EIS-derived Tafel slope was calculated by plotting hydrogen adsorption resistance (R_2_) as a function of applied potentials (U).

### Hydrogen desorption based on CV measurements

CV measurements were conducted in Ar-saturated KOH electrolyte solution under a scan rate range from 50 to 600 mV s^−1^. The hydrogen desorption peak positions were plotted with scan rates, and the slopes of the curves were calculated to study the hydrogen desorption kinetics.

### Temperature-dependent electrochemical impedance spectroscopy (EIS) measurements

Temperature-dependent EIS measurements of SNC-LSF were conducted using a Solartron 1260 coupled with Solartron 1287 via the quasi-four-probe method. A 0.4 g sample was pressed into uniform pellets with a diameter of 12 mm and a thickness of 1-2 mm. The pellets were placed in an anhydrous chamber or under humidified conditions for 24 h, after which conductivity measurements were performed under various conditions.

### Electrochemical in situ Raman spectroscopy

In situ Raman measurements were conducted on a Horiba JY Labram EVO micro-Raman system equipped with a 50 × objective lens, using carbon paper (0.5 cm × 0.5 cm) loaded with catalyst, an Ag/AgCl reference electrode, and a platinum counter electrode. Experiments were performed in Ar-saturated 0.1 M KOH containing 8 mM KNO_3_ under varying applied potentials, and Raman spectra were collected after 5 min of potential application.

### Electrochemical in situ ATR-SEIRAS spectroscopy

In situ ATR-SEIRAS measurements were carried out on a Nicolet iS50 spectrometer equipped with an MCT detector cooled by liquid nitrogen. A custom-made three-electrode cell was used, with Hg/HgO as the reference electrode, Pt wire as the counter electrode, and Au-coated Si prisms (20 mm diameter) loaded with catalyst ink as the working electrode. Au-coated Si substrates were prepared by polishing, piranha treatment, and gold plating of semi-cylindrical Si prisms. Spectra were recorded in 0.1 M KOH with 8 mM NO_3_^-^ under potentials from 0 to − 0.9 V vs. RHE, using the spectrum at OCP as the background.

### Electrochemical online DEMS tests

Online DEMS experiments were performed in a custom-made reactor using Ar-saturated 0.1 M KOH with 8 mM NO_3_^-^ as electrolyte. Catalysts coated on a breathable gold-coated PTFE membrane (pore size ≤ 20 nm, thickness 40 μm, uniform pore distribution, brand Linglu), Pt wire, and a saturated Hg/HgO electrode served as the working, counter, and reference electrodes, respectively. Potentials of OCP and − 0.4 V vs. RHE were alternately applied at 1 min intervals for five cycles, and differential mass signals were recorded to track gaseous products. Prior to isotope-labeling DEMS measurements, the catalyst-coated electrodes were thoroughly cleaned to remove any physically adsorbed species. Specifically, the electrodes were rinsed with ultrapure water and subjected to repeated sonication, followed by drying under an inert atmosphere. This procedure ensures the effective removal of weakly bound adsorbates, minimizing interference from physically adsorbed deuterium species during subsequent measurements.

### Electron spin resonance (ESR) spectroscopy for radical determination

Hydrogen radicals were detected by ESR spectroscopy (Bruker A300) using 5,5-dimethyl−1-pyrroline N-oxide (DMPO, 100 mM) as the spin-trapping reagent. Prior to electrolysis, the electrolyte was purged with Ar for 30 min, followed by the addition of DMPO. After 5 min electrolysis, the electrolyte was collected and analyzed at room temperature.

### NITRR in MEA system and NH_3_ recovery

The home-made MEA electrolyzer comprises an anode (electrode size of 4.0 cm^2^), a cathode (electrode size of 4.0 cm^2^), an anion exchange membrane, gaskets, flow channels, current collectors and end plates. The membrane was immersed in 1 M KOH solution for at least 24 h before use. The catalyst coated substrate technology was adopted to prepare the electrodes. The cathode ink solution was prepared by mixing 200 mg catalysts with 40 mg conductive carbon black and 600 mg 5 wt.% ionomer solution in 16 g solvent (DI water: isopropyl alcohol = 1: 10 at mass ratio) and ultrasonicated at room temperature for 1 h. The anode ink solution was prepared by mixing 200 mg NiFeOx, 150 mg 5 wt.% ionomer solution and 16 g solvent (DI water: isopropyl alcohol = 1: 10 at mass ratio), followed by the same ultrasonic dispersion for 1 h. The as-prepared cathode and anode catalyst inks were then spray-deposited onto the Carbon paper and Ni felt using an ultrasonic spraying instrument, with the mass loading of cathodes and anodes being 2 mg cm^−2^ and 1 mg cm^−2^, respectively. A solution of 1 M KOH containing 1 M NO_3_^-^ was circulated from the electrolyte reservoir through the cathode side, while another 1 M KOH solution was circulated on the anode side. A fully sealed circulation system was used to prevent NH_3_ volatilization. To convert the produced NH_3_ into NH_4_Cl, the reaction electrolyte was transferred to a sealed bottle connected to another containing an HCl solution (1 M, 200 mL). Inert Ar gas (100 sccm) was supplied for 12 h, enabling selective NH_3_ extraction through air stripping and trapping in the HCl solution to form NH_4_Cl. Water was removed from the product using a rotary evaporator, yielding NH_4_Cl as a white powder. Residual moisture was removed in a box oven, ensuring complete conversion of NO_3_^-^ to NH_4_Cl powder.

### Computational simulations

The computational simulations were carried out using the CP2K software package^70^. We employed the Perdew-Burke-Ernzerhof (PBE) exchange-correlation functional augmented with Grimme’s DFT-D3 dispersion correction for the electronic structure description^71^. Calculations utilized optimized triple-ζ Gaussian basis sets and scalar relativistic norm-conserving pseudopotentials. The valence electrons were explicitly treated as follows: La (11), Sr (10), Fe (16), O (6), N (5), and H (1)^72^. A plane-wave cutoff energy of 400 Ry and a relative cutoff of 50 Ry were used within the dual-plane-wave approach. Integration over the Brillouin zone was performed solely at the Γ-point. All calculations adopted the spin-unrestricted formalism to accommodate potential open-shell interactions. We also utilized the Multiwfn software package during the computational process^73,74^.

The computational hydrogen electrode (CHE) model developed by Nørskov et al. was applied. This model is expressed as ref. ^75^:

$${\mbox{ΔG}}={\mbox{ΔE}}+{\mbox{Δ}}{{\mbox{G}}}_{{\mbox{U}}}$$

Here, ΔE denotes the quantum-chemical reaction energy derived directly from DFT calculations; Δ*G*_U_ quantifies the electrochemical free energy contribution and is defined as Δ*G*_U_ = qU, where q represents the number of transferred electrons, and U is the applied electrode potential. Within this framework, the step exhibiting the largest free energy increase (ΔG) was identified as the potential-determining step (PDS), which serves to evaluate catalytic activity. We also performed Born-Oppenheimer molecular dynamics (BOMD) simulations to construct an amorphous surface of SNC-LSF and characterize the dynamic properties of the liquid system, with a double-ζ Gaussian basis set. For the construction of the SNC-LSF surface, the Sr atoms on the LSF surface were replaced with H atoms at twice the atomic ratio. An NVT ensemble simulation was then performed at 1800 K for 5 ps to obtain a randomized, followed by slowly cooling to 0 K. After removing the unbound H_2_O molecules generated, geometry optimization was conducted to obtain the SNC-LSF structure. Simulations employed an NVT ensemble maintained at 298 K with a canonical sampling velocity rescaling thermostat, using a time step of 1.0 fs for the dynamic properties of the liquid-solid interface. To evaluate kinetic barriers and sample the free energy profile associated with hydrogen atom transfer, we used constrained molecular dynamics (cMD) simulations combined with the thermodynamic integration method, using a time step of 0.5 fs^76^. This approach enables the study of rare reaction events by imposing a holonomic constraint on a predefined reaction coordinate (ξ). By performing thermodynamic integration over the solvent average force of constraint 〈*f*s(*ξ*′)〉*ξ*′ (with respect to the specified reaction coordinate *ξ*′), the free energy Δ*A*(*ξ*) relative to a chosen reference state *ξ*0 can be computed according to eq2:

$${\Delta} {{\rm{A}}} ({\xi})=- {\int _{{\xi}_{0}}^{\xi}} {{\rm{d}}} {\xi}^{\prime} {\left\langle {{\rm{f}}}_{{\rm{s}}}({\xi}^{\prime})\right\rangle}_{{\xi}^{\prime}}$$

The solvent-averaged constraint force 〈*f*s(*ξ*′)〉*ξ*′ corresponds to the offset of the induced bias in the bare constraint force λ (the Lagrange multiplier enforcing the constraint). In this work, the SHAKE algorithm was implemented to integrate the Newtonian equations of motion under the specified constraints^77^. The reaction coordination for H migration on the SNC-LSF surface was described using a collective variable defined as the difference between the O1-H distance and the O2-H distance.

To avoid using the charged NO_3_^-^ species as a reference, the neutral HNO_3_ was alternatively used to calculate the Gibbs free energy of NO3 on the basis of literatureis^78–80^, and the ΔG(*NO3) can be calculated as

$${\mbox{Δ}}{{\mbox{G}}}_{*{{\mbox{NO}}}_{3}}={{\mbox{G}}}_{*{{\mbox{NO}}}_{3}}-{{\mbox{G}}}_{*}-{{\mbox{G}}}_{{{\mbox{HNO}}}_{3}({\mbox{g}})}+0.5{{\mbox{G}}}_{{{\mbox{H}}}_{2}({\mbox{g}})}+{\mbox{Δ}}{{\mbox{G}}}_{{\mbox{correct}}}$$

$${\mbox{Δ}}{{\mbox{G}}}_{{\mbox{correct}}}=\Delta {{\mbox{G}}}_{{\mbox{S}}1}+{\mbox{Δ}}{{\mbox{G}}}_{{\mbox{S}}2}$$

Where ${{\mbox{G}}}_{*{{\mbox{NO}}}_{3}}$, ${{\mbox{G}}}_{*}$, ${{\mbox{G}}}_{{{\mbox{HNO}}}_{3}({\mbox{g}})}$ and ${{\mbox{G}}}_{{{\mbox{H}}}_{2}({\mbox{g}})}$ are the Gibbs free energies of adsorbed NO_3_, clean substrate, HNO_3_ and H_2_ molecules in the gas phase, respectively. ${\mbox{Δ}}{{\mbox{G}}}_{{\mbox{correct}}}$ denotes the correction of the adsorption energy. ${\mbox{Δ}}{{\mbox{G}}}_{{\mbox{S}}1}$ and ${\mbox{Δ}}{{\mbox{G}}}_{{\mbox{S}}2}$ are the Gibbs free energy of formation of HNO_3_(l) from NO_3_^-^(aq) (0.317 eV) and the Gibbs free energy of vaporization of HNO_3_(l) (0.075 eV). Both values can be obtained from the CRC handbook, and thesame approach has been done in literatureis^78–80^.

## Supplementary information

Supplementary Information

Description of Additional Supplementary Files

Supplementary Data 1

Supplementary Data 2

Supplementary Data 3

Transparent Peer Review file

## Source data

Source Data

## Data Availability

The data supporting the conclusions of this study are presented in the paper and its supplementary information. Source data are provided in this paper.

## References

[1] Ashida, Y., Arashiba, K., Nakajima, K. & Nishibayashi, Y. Molybdenum-catalysed ammonia production with samarium diiodide and alcohols or water. *Nature* **568**, 536–540 (2019). 31019315 10.1038/s41586-019-1134-2

[2] Suryanto, B. H. R. et al. Challenges and prospects in the catalysis of electroreduction of nitrogen to ammonia. *Nat. Catal.* **2**, 290–296 (2019).

[3] Tang, C. & Qiao, S.-Z. How to explore ambient electrocatalytic nitrogen reduction reliably and insightfully. *Chem. Soc. Rev.* **48**, 3166–3180 (2019). 31107485 10.1039/c9cs00280d

[4] Dai, J. et al. Spin polarized Fe_1_-Ti pairs for highly efficient electroreduction nitrate to ammonia. *Nat. Commun.* **15**, 88 (2024). 38167739 10.1038/s41467-023-44469-4 PMC10762114

[5] Ford, C. L., Park, Y. J., Matson, E. M., Gordon, Z. & Fout, A. R. A bioinspired iron catalyst for nitrate and perchlorate reduction. *Science* **354**, 741–743 (2016). 27846604 10.1126/science.aah6886

[6] Anderson, J. S., Rittle, J. & Peters, J. C. Catalytic conversion of nitrogen to ammonia by an iron model complex. *Nature* **501**, 84–87 (2013). 24005414 10.1038/nature12435 PMC3882122

[7] Murphy, E. et al. Elucidating electrochemical nitrate and nitrite reduction over atomically-dispersed transition metal sites. *Nat. Commun.* **14**, 4554 (2023). 37507382 10.1038/s41467-023-40174-4 PMC10382506

[8] Li, P., Jin, Z., Fang, Z. & Yu, G. A single-site iron catalyst with preoccupied active centers that achieves selective ammonia electrosynthesis from nitrate. *Energy Environ. Sci.* **14**, 3522–3531 (2021).

[9] Du, H. et al. Durable electrocatalytic reduction of nitrate to ammonia over defective pseudobrookite Fe_2_TiO_5_ nanofibers with abundant oxygen vacancies. *Angew. Chem. Int. Ed.* **62**, e202215782 (2023). 10.1002/anie.202215782 36468550

[10] Xu, H., Ma, Y., Chen, J., Zhang, W. & Yang, J. Electrocatalytic reduction of nitrate-a step towards a sustainable nitrogen cycle. *Chem. Soc. Rev.* **51**, 2710–2758 (2022). 35274646 10.1039/d1cs00857a

[11] Chandra Majhi, K. et al. In-tandem electrochemical reduction of nitrate to ammonia on ultrathin-sheet-assembled iron-nickel alloy nanoflowers. *Angew. Chem. Int. Ed.* **64**, e202500167 (2025). 10.1002/anie.202500167 39904929

[12] Li, P. et al. Pulsed nitrate-to-ammonia electroreduction facilitated by tandem catalysis of nitrite intermediates. *J. Am. Chem. Soc.* **145**, 6471–6479 (2023). 36897656 10.1021/jacs.3c00334

[13] Wang, Y. et al. Atomic coordination environment engineering of bimetallic alloy nanostructures for efficient ammonia electrosynthesis from nitrate. *Proc. Natl. Acad. Sci. USA* **120**, e2306461120 (2023). 37523530 10.1073/pnas.2306461120 PMC10410719

[14] Hu, Q. et al. Subnanometric nickel phosphide heteroclusters with highly active Ni^δ+^-P^δ-^ pairs for nitrate reduction toward ammonia. *J. Am. Chem. Soc.* **147**, 12228–12238 (2025). 40138702 10.1021/jacs.5c01455

[15] Liao, W. et al. Sustainable conversion of alkaline nitrate to ammonia at activities greater than 2 A cm^-2^. *Nat. Commun.* **15**, 1264 (2024). 38341446 10.1038/s41467-024-45534-2 PMC10858923

[16] Wang, Y.-H. et al. In situ Raman spectroscopy reveals the structure and dissociation of interfacial water. *Nature* **600**, 81–85 (2021). 34853456 10.1038/s41586-021-04068-z

[17] Musat, R. et al. Finite size effects on hydrogen bonds in confined water. *Angew. Chem. Int. Ed.* **47**, 8033–8035 (2008). 10.1002/anie.200802630 18792051

[18] Li, S. et al. Uncovering the dominant role of an extended asymmetric four-coordinated water network in the hydrogen evolution reaction. *J. Am. Chem. Soc.* **145**, 26711–26719 (2023). 38031299 10.1021/jacs.3c08333

[19] Muñoz-Santiburcio, D. & Marx, D. Nanoconfinement in slit pores enhances water self-dissociation. *Phys. Rev. Lett.* **119**, 056002 (2017). 28949727 10.1103/PhysRevLett.119.056002

[20] Shifa, T. A. & Vomiero, A. Confined catalysis: progress and prospects in energy conversion. *Adv. Energy Mater.* **9**, 1970158 (2019).

[21] Liu, M. et al. Enhanced electrocatalytic CO_2_ reduction via field-induced reagent concentration. *Nature* **537**, 382–386 (2016). 27487220 10.1038/nature19060

[22] Li, X. et al. Mechanism of cations suppressing proton diffusion kinetics for electrocatalysis. *Angew. Chem. Int. Ed.* **62**, e202218669 (2023). 10.1002/anie.202218669 36762956

[23] Chen, Y. et al. Exceptionally active iridium evolved from a pseudo-cubic perovskite for oxygen evolution in acid. *Nat. Commun.* **10**, 572 (2019). 30718514 10.1038/s41467-019-08532-3 PMC6362036

[24] Chen, Y. et al. Support-free iridium hydroxide for high-efficiency proton-exchange membrane water electrolysis. *Nat. Commun.* **16**, 2730 (2025). 40108156 10.1038/s41467-025-58019-7 PMC11923266

[25] Fabbri, E. et al. Dynamic surface self-reconstruction is the key of highly active perovskite nano-electrocatalysts for water splitting. *Nat. Mater.* **16**, 925–931 (2017). 28714982 10.1038/nmat4938

[26] Zhu, Y. et al. Beyond conventional structures: emerging complex metal oxides for efficient oxygen and hydrogen electrocatalysis. *Chem. Soc. Rev.* **54**, 1027–1092 (2025). 39661069 10.1039/d3cs01020a

[27] Yuan, L., Dong, Z., Tang, Z., Tao, H. & Zhu, Y. Low-iridium/ruthenium perovskite oxides: An emerging family of material platforms for oxygen evolution reaction in acid. *J. Energy Chem.* **109**, 186–209 (2025).

[28] Dai, J. et al. A complex oxide containing inherent peroxide ions for catalyzing oxygen evolution reactions in acid. *J. Am. Chem. Soc.* **146**, 33663–33674 (2024). 39585747 10.1021/jacs.4c11477

[29] Speck, F. D., Zagalskaya, A., Alexandrov, V. & Cherevko, S. Periodicity in the electrochemical dissolution of transition metals. *Angew. Chem. Int. Ed.* **60**, 13343–13349 (2021). 10.1002/anie.202100337 PMC8252536 33687762

[30] Zhang, B. et al. Atomically dispersed chromium coordinated with hydroxyl clusters enabling efficient hydrogen oxidation on ruthenium. *Nat. Commun.* **13**, 5894 (2022). 36202856 10.1038/s41467-022-33625-x PMC9537559

[31] Kumagai, T. et al. Tunneling dynamics of a hydroxyl group adsorbed on Cu (110). *Phys. Rev. B* **79**, 035423 (2009).

[32] Merte, L. R. et al. Water-mediated proton hopping on an iron oxide surface. *Science* **336**, 889–893 (2012). 22605771 10.1126/science.1219468

[33] Liu, Y. et al. Direct observation of accelerating hydrogen spillover via surface-lattice-confinement effect. *Nat. Commun.* **14**, 613 (2023). 36739275 10.1038/s41467-023-36044-8 PMC9899253

[34] Mahdavi-Shakib, A. et al. The role of surface hydroxyls in the entropy-driven adsorption and spillover of H_2_ on Au/TiO_2_ catalysts. *Nat. Catal.* **6**, 710–719 (2023).

[35] Zhang, W. et al. Strontium doped IrO_x_ triggers direct O−O coupling to boost acid water oxidation electrocatalysis. *Angew. Chem. Int. Ed.* **64**, e202418456 (2025). 10.1002/anie.202418456 39387682

[36] Mutoro, E., Crumlin, E. J., Biegalski, M. D., Christen, H. M. & Shao-Horn, Y. Enhanced oxygen reduction activity on surface-decorated perovskite thin films for solid oxide fuel cells. *Energy Environ. Sci.* **4**, 3689 (2011).

[37] Crumlin, E. J. et al. Surface strontium enrichment on highly active perovskites for oxygen electrocatalysis in solid oxide fuel cells. *Energy Environ. Sci.* **5**, 6081 (2012).

[38] Xu, W. et al. A-site cation leaching induced surface reconstruction in perovskite oxides for enhanced electrocatalytic nitrate reduction. *ACS Nano* **19**, 39480–39490 (2025).41187303

[39] Li, L. et al. Exceptional oxygen evolution reactivities on CaCoO_3_ and SrCoO_3_. *Sci. Adv.* **5**, eaav6262 (2019). 31448324 10.1126/sciadv.aav6262 PMC6688868

[40] Seitz, L. C. et al. A highly active and stable IrO_*x*_/SrIrO_3_ catalyst for the oxygen evolution reaction. *Science* **353**, 1011–1014 (2016). 27701108 10.1126/science.aaf5050

[41] Wan, G. et al. Amorphization mechanism of SrIrO_3_ electrocatalyst: How oxygen redox initiates ionic diffusion and structural reorganization. *Sci. Adv.* **7**, eabc7323 (2021). 33523986 10.1126/sciadv.abc7323 PMC7793586

[42] Zhang, J. et al. Large orbital polarization in a metallic square-planar nickelate. *Nat. Phys.* **13**, 864–869 (2017).

[43] Lopes, P. P. et al. Dynamically stable active sites from surface evolution of perovskite materials during the oxygen evolution reaction. *J. Am. Chem. Soc.* **143**, 2741–2750 (2021). 33399469 10.1021/jacs.0c08959

[44] Lian, W. et al. Planar asymmetric surface Fe^IV^=O synthesis with pyrite and chlorite for efficient oxygen atom transfer reactions. *Nat. Commun.* **16**, 5989 (2025). 40595548 10.1038/s41467-025-60919-7 PMC12219332

[45] Lazemi, M. et al. Ultrafast light-driven electronic and structural changes in LaFeO_3_ perovskites probed by femtosecond X-ray absorption spectroscopy. *Adv. Mater.* **37**, 2502932 (2025). 40370187 10.1002/adma.202502932 PMC12288815

[46] Blasco, J. et al. Charge disproportionation in La_1-x_Sr_x_FeO_3_ probed by diffraction and spectroscopic experiments. *Phys. Rev. B* **77**, 054107 (2008).

[47] Qu, K. et al. Enhancing nitrate reduction to ammonia through crystal phase engineering: Unveiling the hydrogen bonding effect in δ-FeOOH electrocatalysis. *Small* **20**, 2401327 (2024). 10.1002/smll.202401327 38429245

[48] Fang, L. et al. Removal mechanisms of phosphate by lanthanum hydroxide nanorods: Investigations using EXAFS, ATR-FTIR, DFT, and surface complexation modeling approaches. *Environ. Sci. Technol.* **51**, 12377–12384 (2017). 29035555 10.1021/acs.est.7b03803

[49] Dong, H. et al. Amine-functionalized quasi-MOF for direct air capture of CO_2_. *ACS Mater. Lett.* **5**, 2656–2664 (2023).

[50] Li, Y. et al. Reversible hydrogen acceptor-donor enables relay mechanism for nitrate-to-ammonia electrocatalysis. *Angew*. *Chem. Int. Ed.* **64**, e202417631 (2025). 10.1002/anie.202417631 39431499

[51] Cui, J., Olmsted, D. L., Mehta, A. K., Asta, M. & Hayes, S. E. NMR crystallography: Evaluation of hydrogen positions in hydromagnesite by^13^ C{^1^ H} REDOR solid-state NMR and density functional theory calculation of chemical shielding tensors. *Angew*. *Chem. Int. Ed.* **58**, 4210 (2019). 10.1002/anie.201813306 30672073

[52] Xiao, Y. et al. Strained au skin on mesoporous intermetallic AuCu_3_ nanocoral for electrocatalytic conversion of nitrate to ammonia across a wide concentration range. *Angew. Chem. Int. Ed.* **63**, e202408758 (2024). 10.1002/anie.202408758 38899532

[53] Gao, W. et al. Alloying of cu with ru enabling the relay catalysis for reduction of nitrate to ammonia. *Adv. Mater.* **35**, 2202952 (2023). 10.1002/adma.202202952 36871207

[54] Li, J. et al. Lattice hydrogen transfer in titanium hydride enhances electrocatalytic nitrate to ammonia conversion. *Nat. Commun.* **15**, 9499 (2024). 39489774 10.1038/s41467-024-53833-x PMC11532501

[55] Chen, Y. et al. A-site cation deficiency in antiperovskites for precisely accelerating the volmer step of alkaline hydrogen evolution. *Adv. Energy Mater*. **15**, e03319 (2025).

[56] Fang, Q. & Chen, B. Self-assembly of graphene oxide aerogels by layered double hydroxides cross-linking and their application in water purification. *J. Mater. Chem. A* **2**, 8941–8951 (2014).

[57] Zhao, R. et al. Pd single atoms guided proton transfer along an interfacial hydrogen bond network for efficient electrochemical hydrogenation. *Sci. Adv.* **11**, eadu1602 (2025). 40779631 10.1126/sciadv.adu1602 PMC12333693

[58] Lei, X. et al. Unraveling the oxygen vacancy site mechanism of a self-assembly hybrid catalyst for efficient alkaline water oxidation. *ACS Catal.* **14**, 4523–4535 (2024).

[59] Chen, L., Xu, Q. & Boettcher, S. W. Kinetics and mechanism of heterogeneous voltage-driven water-dissociation catalysis. *Joule* **7**, 1867–1886 (2023).

[60] Deng, Y. et al. Mechanical and covalent tailoring of copper catenanes for selective aqueous nitrate-to-ammonia electrocatalysis. *J. Am. Chem. Soc.* **147**, 14316–14325 (2025). 40260598 10.1021/jacs.4c18547 PMC12046556

[61] Meng, L. et al. Steering the nitrate electroreduction pathway via nanoconfinement-induced hydrogen-bond network regulation. *Nat. Water* **4**, 481–492 (2026).

[62] Tao, Y. et al. Tuning the hydrogen evolution reaction activity and mechanism on pt via nano-confinement of interfacial water. *J. Am. Chem. Soc.* **147**, 29977–29982 (2025). 40782074 10.1021/jacs.5c07325

[63] Ma, Z. & Cheng, H. Insights into the photochemical mechanism of goethite: roles of different types of surface hydroxyl groups in reactive oxygen species generation and Fe(III) reduction. *Environ. Sci. Technol.* **58**, 14812–14822 (2024). 39118219 10.1021/acs.est.4c03352

[64] Mao, Y. et al. Catalytic cascade depolymerization for sustainable recycling of waste polyvinyl chloride. *Nat. Sustain.* **8**, 1513–1523 (2025).

[65] Wang, Y. et al. Strong hydrogen-bonded interfacial water inhibiting hydrogen evolution kinetics to promote electrochemical CO_2_ reduction to C_2+_. *ACS Catal.* **14**, 3457–3465 (2024).

[66] Lin, H. et al. Bi_1_-CuCo_2_O_4_ hollow carbon nanofibers boosts NH_3_ production from electrocatalytic nitrate reduction. *Adv. Funct. Mater.* **34**, 2409696 (2024).

[67] Zhang, B. et al. Defect-induced triple synergistic modulation in copper for superior electrochemical ammonia production across broad nitrate concentrations. *Nat. Commun.* **15**, 2816 (2024). 38561364 10.1038/s41467-024-47025-w PMC10984973

[68] Wang, R. et al. Compressive-strained rutile TiO_2_ enables O_2_ mono-hydrogenation for singlet oxygen electrosynthesis. *Nat. Synth.* **4**, 754–764 (2025).

[69] Schnitkey, G., Paulson, N., Zulauf, C. & Baltz, J. *Fertilizer Prices and Company Profits Going into Spring 2023.* (2023).

[70] Vande Vondele, J. et al. Quickstep: Fast and accurate density functional calculations using a mixed gaussian and plane waves approach. *Comput. Phys. Commun.* **167**, 103–128 (2005).

[71] Grimme, S., Antony, J., Ehrlich, S. & Krieg, H. A consistent and accurate ab initio parametrization of density functional dispersion correction (DFT-D) for the 94 elements H-pu. *J. Chem. Phys.* **132**, 154104 (2010). 20423165 10.1063/1.3382344

[72] VandeVondele, J. & Hutter, J. Gaussian basis sets for accurate calculations on molecular systems in gas and condensed phases. *J. Chem. Phys.* **127**, 114105 (2007). 17887826 10.1063/1.2770708

[73] Lu, T. & Chen, F. Multiwfn: A multifunctional wavefunction analyzer. *J. Comput. Chem.* **33**, 580–592 (2012). 22162017 10.1002/jcc.22885

[74] Lu, T. A comprehensive electron wavefunction analysis toolbox for chemists, multiwfn. *J. Chem. Phys.* **161**, 082503 (2024). 39189657 10.1063/5.0216272

[75] Nørskov, J. K. et al. Origin of the overpotential for oxygen reduction at a fuel-cell cathode. *J. Phys. Chem. B* **108**, 17886–17892 (2004). 39682080 10.1021/jp047349j

[76] Sprik, M. & Ciccotti, G. Free energy from constrained molecular dynamics. *J. Chem. Phys.* **109**, 7737–7744 (1998).

[77] Ryckaert, J.-P., Ciccotti, G. & Berendsen, H. J. C. Numerical integration of the cartesian equations of motion of a system with constraints: Molecular dynamics of n-alkanes. *J. Comput. Phys.* **23**, 327–341 (1977).

[78] Guo, S. et al. Insights into Nitrate reduction over indium-decorated palladium nanoparticle catalysts. *ACS Catal.* **8**, 503–515 (2018).

[79] Liu, J.-X., Richards, D., Singh, N. & Goldsmith, B. R. Activity and selectivity trends in electrocatalytic nitrate reduction on transition metals. *ACS Catal.* **9**, 7052–7064 (2019).

[80] Niu, H. et al. Theoretical insights into the mechanism of selective nitrate-to-ammonia electroreduction on single-atom catalysts. *Adv. Funct. Mater.* **31**, 2008533 (2021).

