# Supplementary material for: Hydroxyl-rich nanocavities on perovskite enable nearly barrierless intramolecular hydrogen transfer for nitrate electroreduction to ammonia
Source: Nat Commun. 2026 Jun 29;17:8062. doi: 10.1038/s41467-026-74405-1 (PMC13454618; doi:10.1038/s41467-026-74405-1)
Supplement: Supplementary file 1 [file 41467_2026_74405_MOESM1_ESM.pdf]

Supplementary Information

Hydroxyl-rich nanocavities on perovskite enable nearly barrierless intramolecular hydrogen transfer for nitrate electroreduction to ammonia

Supplementary Figures

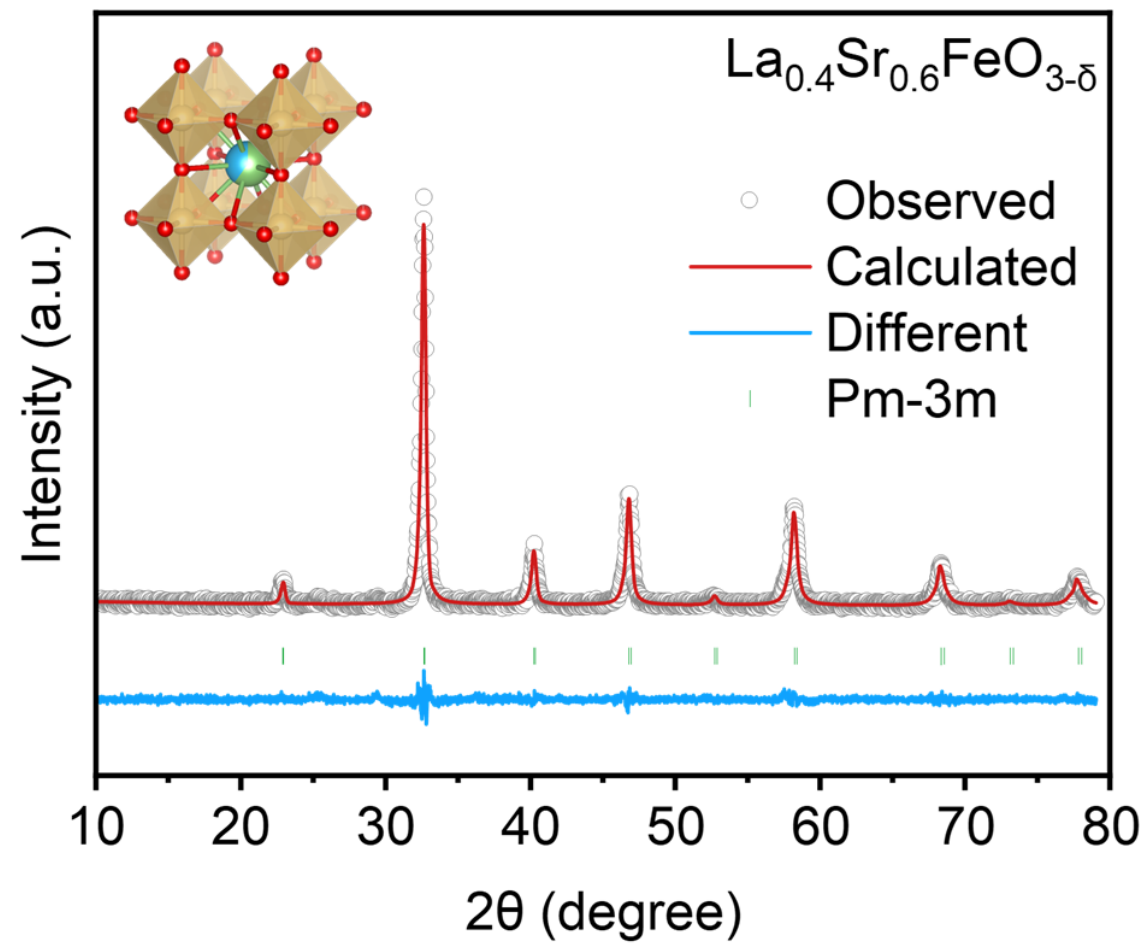

Supplementary Figure 1. Refined XRD profile of $La_{0.4}Sr_{0.6}FeO_{3-\delta}$. Insert shows the local crystal structure of $La_{0.4}Sr_{0.6}FeO_{3-\delta}$. Color code: La (blue), Sr (green), Fe (yellow), and O (red).

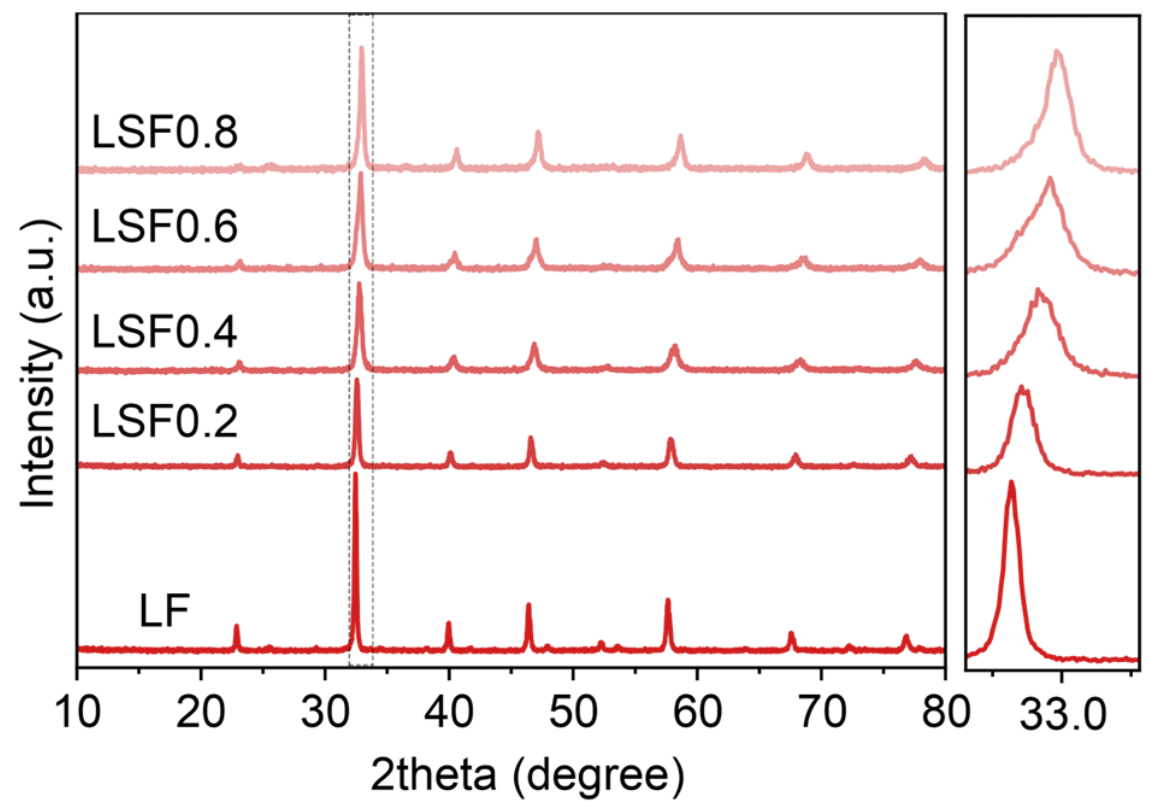

Supplementary Figure 2. XRD patterns of $La_{1-x}Sr_xFeO_{3-\delta}$ (x=0, 0.2, 0.4, 0.6, 0.8), which are denoted as LF, LSF0.2, LSF0.4, LSF0.6 and LSF0.8. The positions of the characteristic diffraction peaks shift systematically toward higher angles with increasing strontium content (x), indicating a reduction in lattice size, this may originate from the B-site charge compensation phenomenon upon substitution of La^{3+} by Sr^{2+}.

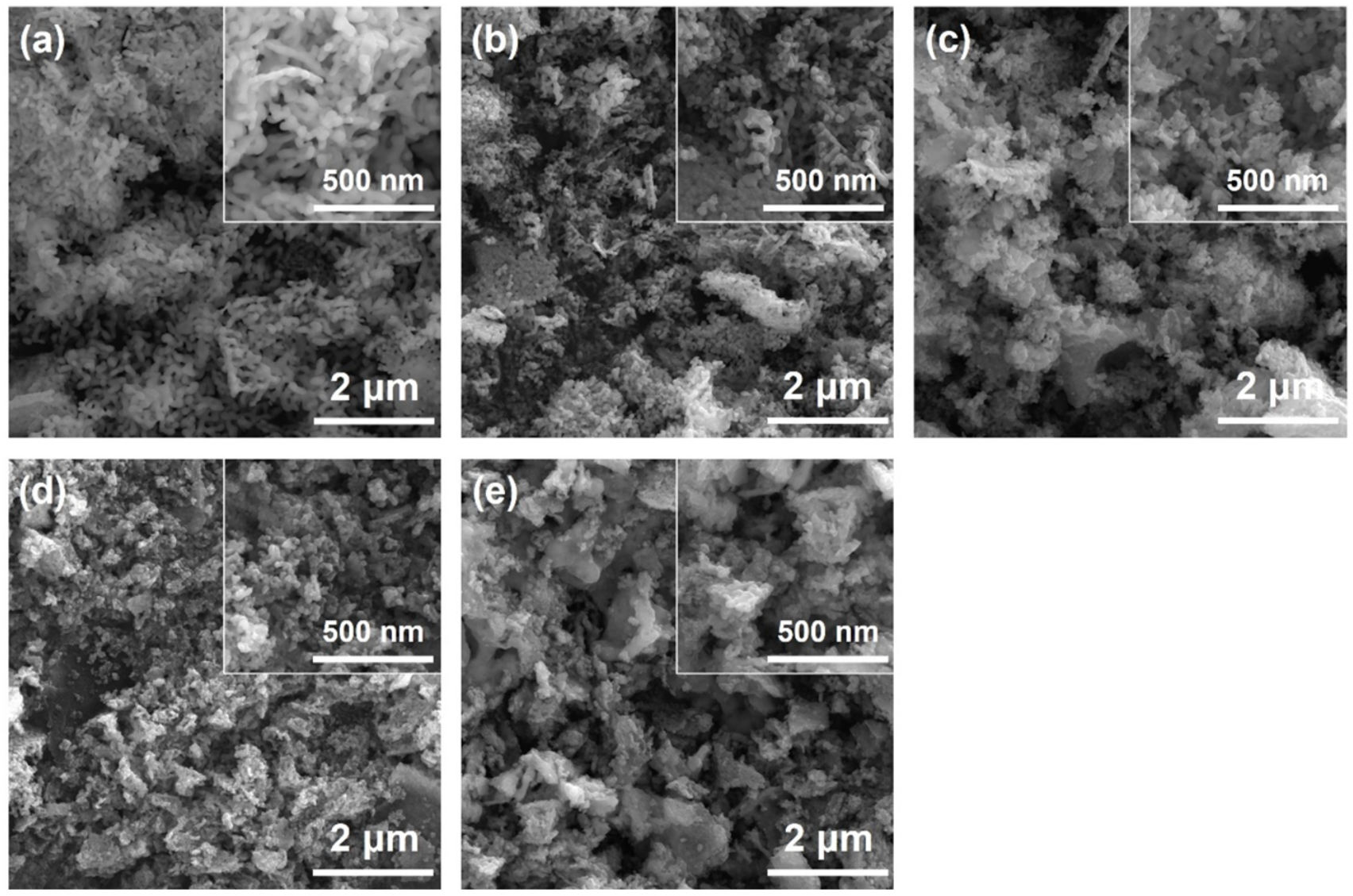

Supplementary Figure 3. SEM images of (a) LF, (b) LSF0.2, (c) LSF0.4, (d) LSF0.6 and (e) LSF0.8.

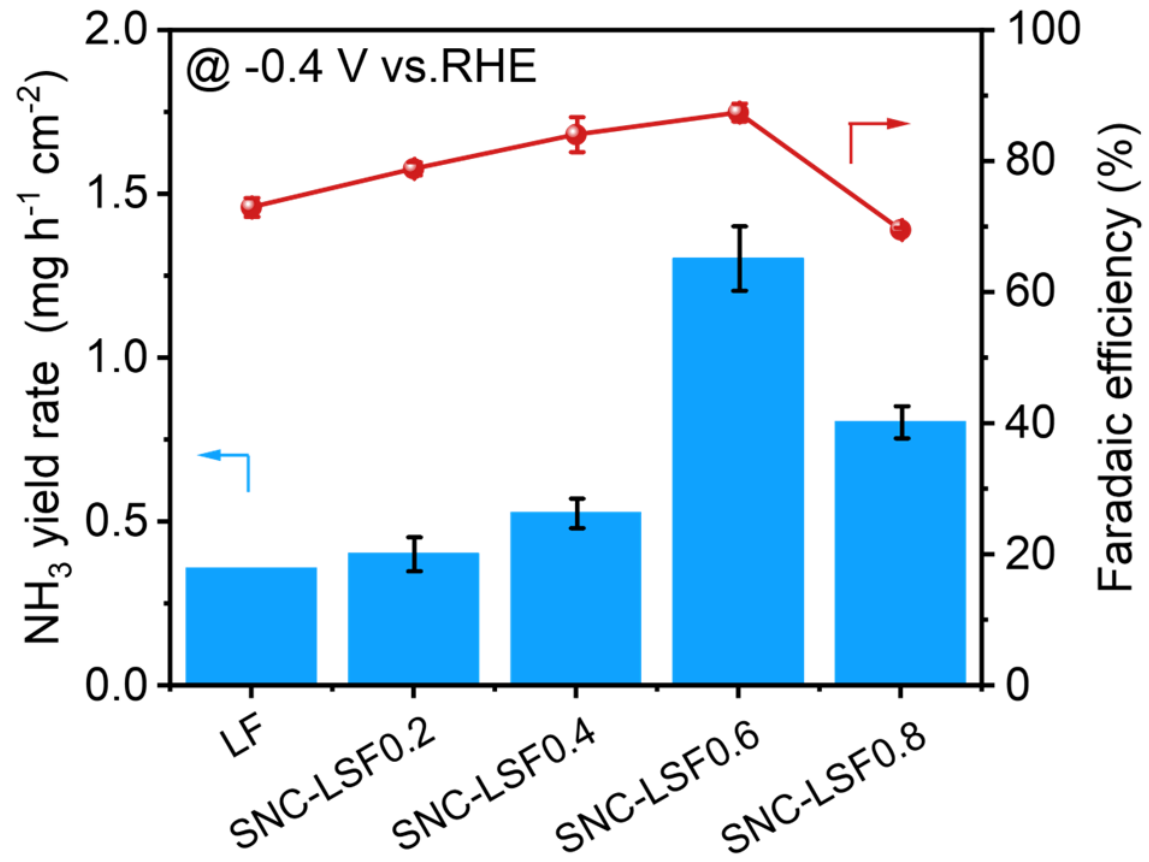

Supplementary Figure 4. NH_3 yield rate and FE_{NH3} of LF and SNC-LSFx (x=0.2, 0.4, 0.6 and 0.8) in 0.1 M KOH and 8 mM KNO_3 at -0.4 V vs. RHE.

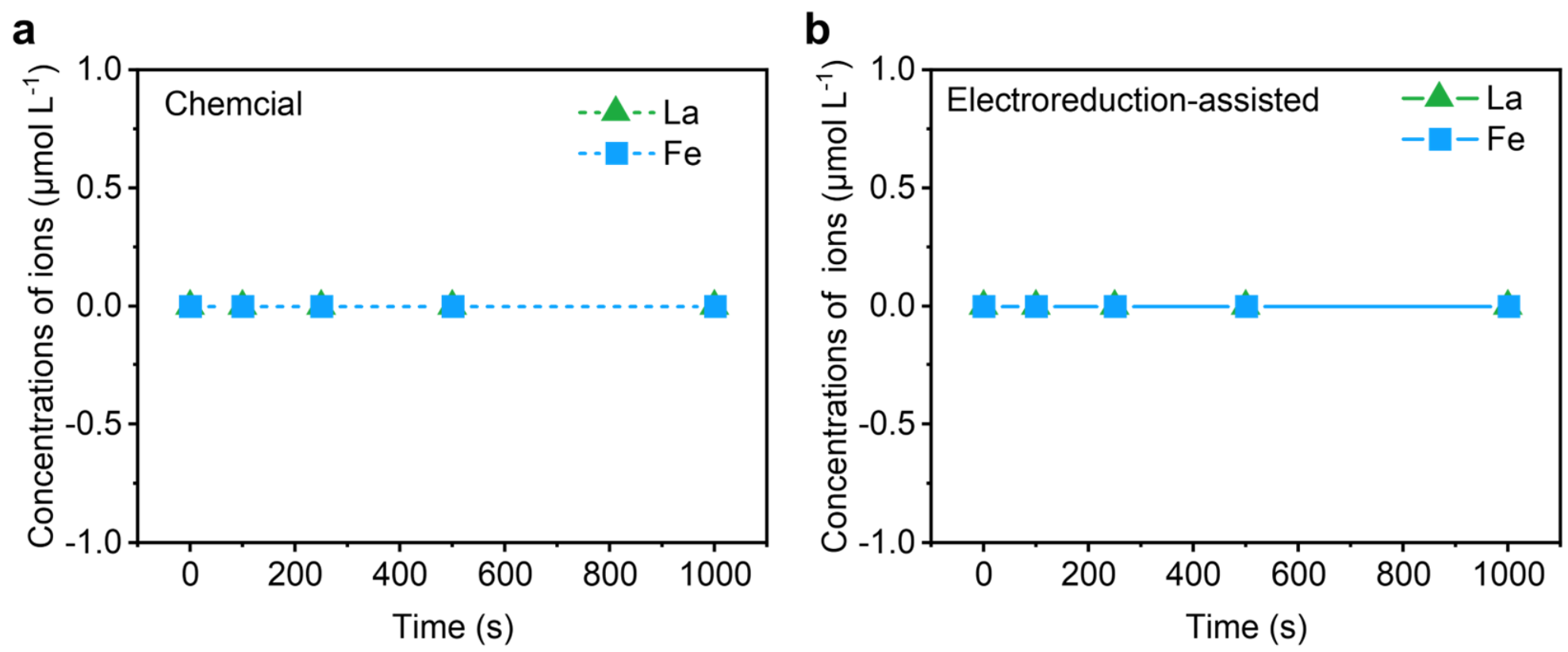

Supplementary Figure 5. (a) Chemical dissolution and (b) electroreduction-assisted dissolution concentrations of La and Fe elements from LSF in 0.1 M KOH.

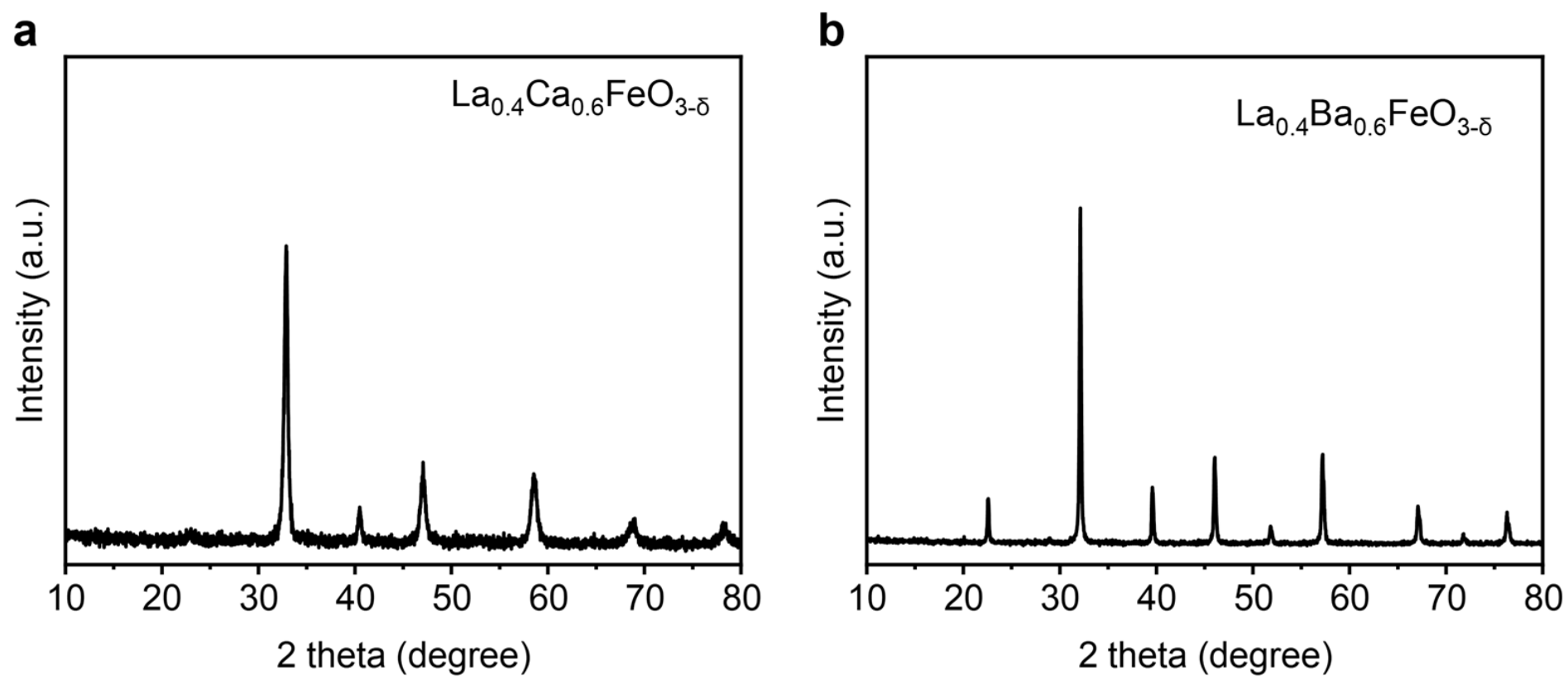

Supplementary Figure 6. XRD patterns of (a) $La_{0.4}Ca_{0.6}FeO_{3-\delta}$, (b) $La_{0.4}Ba_{0.6}FeO_{3-\delta}$.

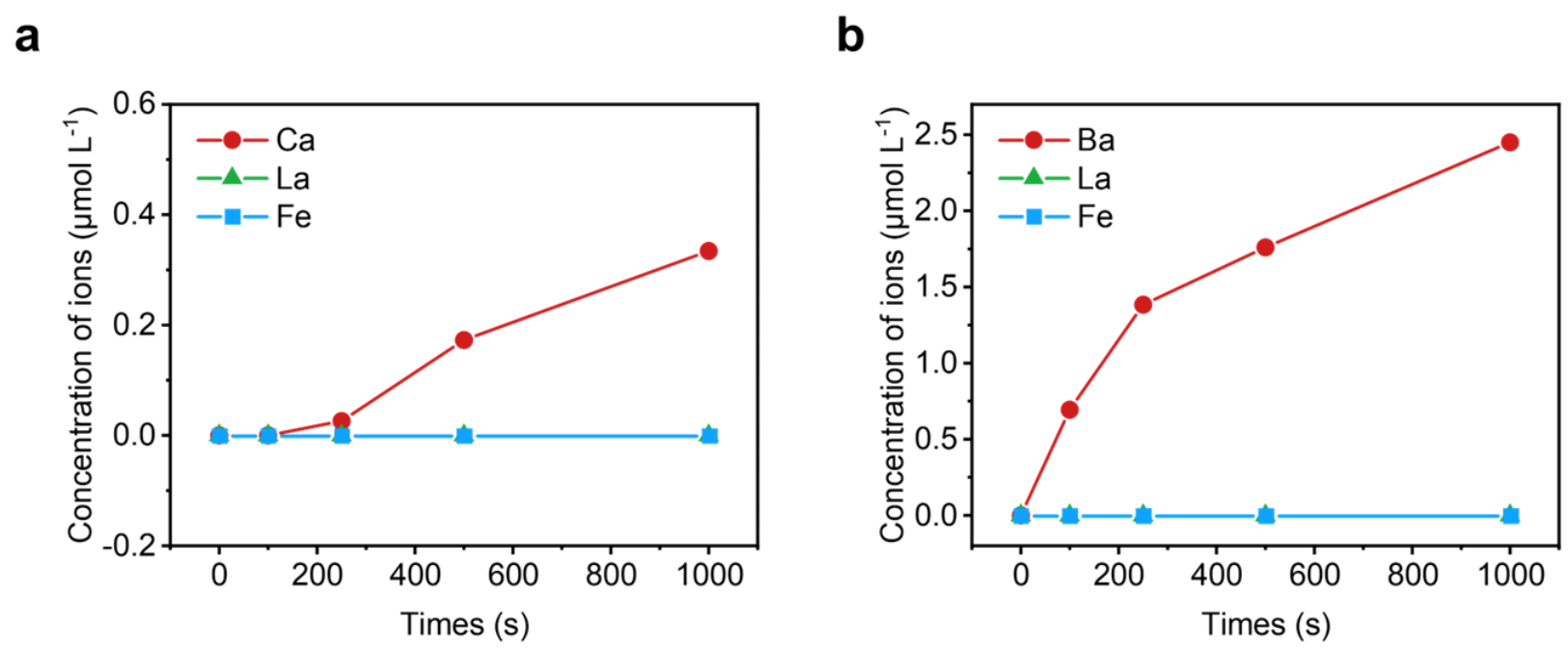

Supplementary Figure 7. Chemical dissolution concentrations of (a) Ca, La and Fe elements from LCF and (b) Ba, La and Fe elements from LBF in 0.1 M KOH.

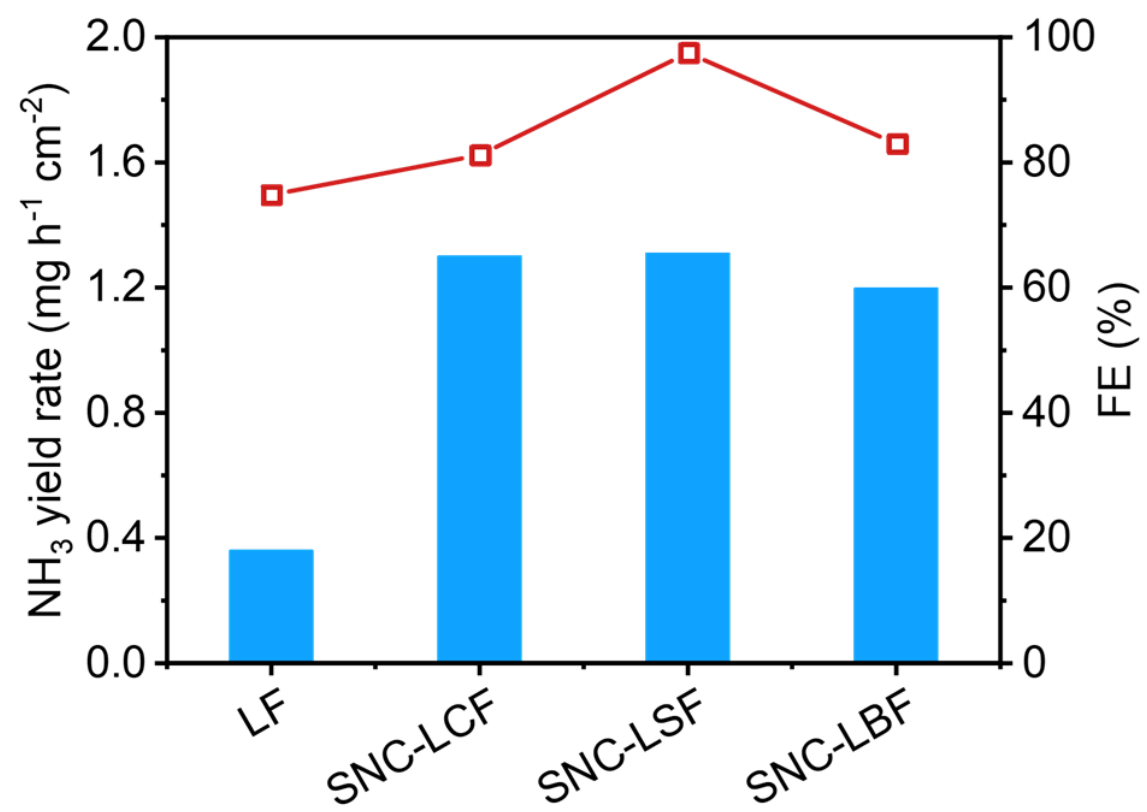

Supplementary Figure 8. NH_3 yield rate and FE_{NH3} of LF, SNC-LCF, SNC-LSF and SNC-LBF in 0.1 M KOH and 8 mM KNO_3 at -0.4 V vs. RHE.

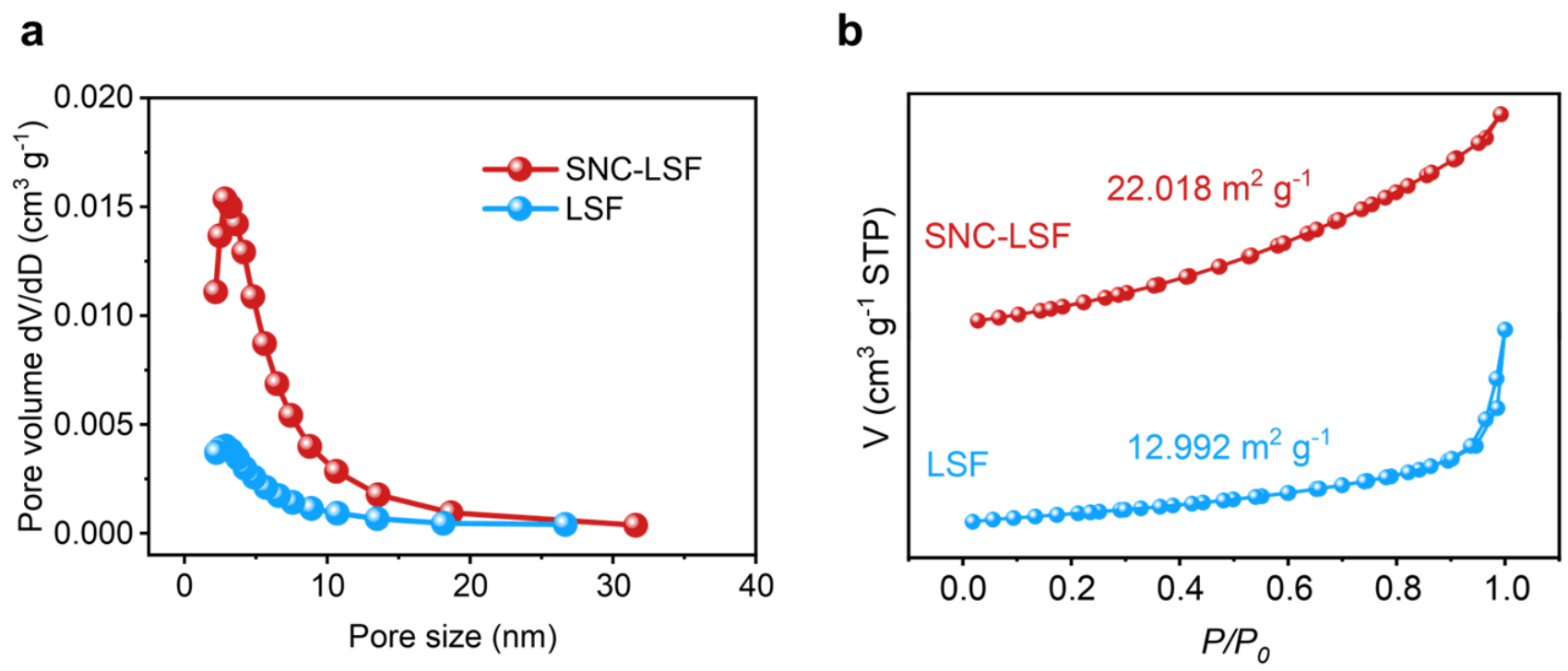

Supplementary Figure 9. (a) Pore size distribution and (b) N_2 sorption isotherm curve of LSF and SNC-LSF.

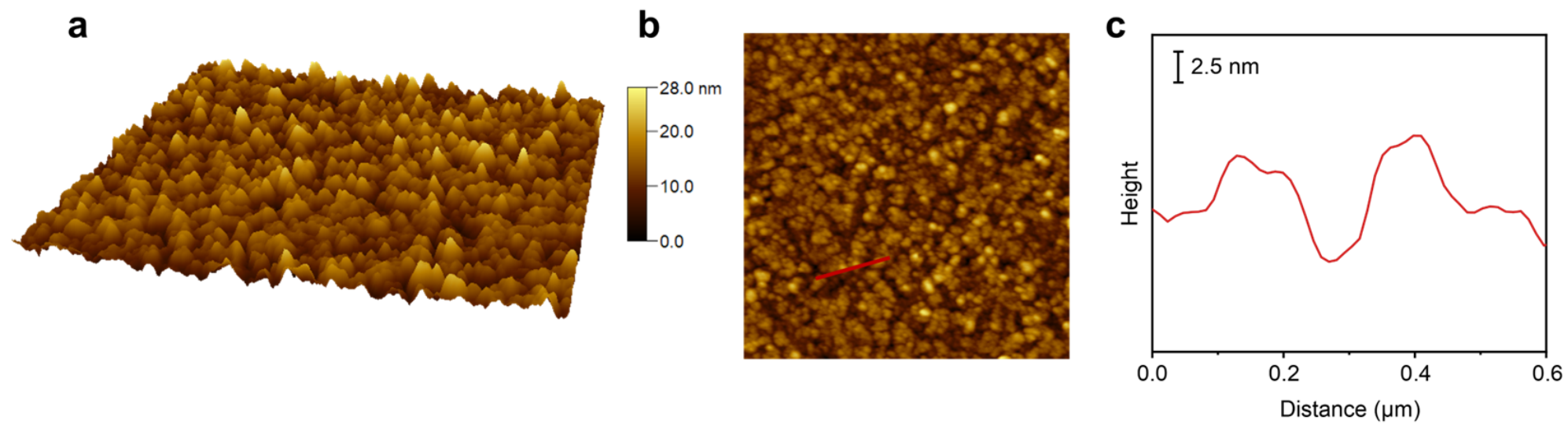

Supplementary Figure 10. (a, b) AFM maps of SNC-LSF. (c) The root mean square (RMS) roughness and line scans of SNC-LSF.

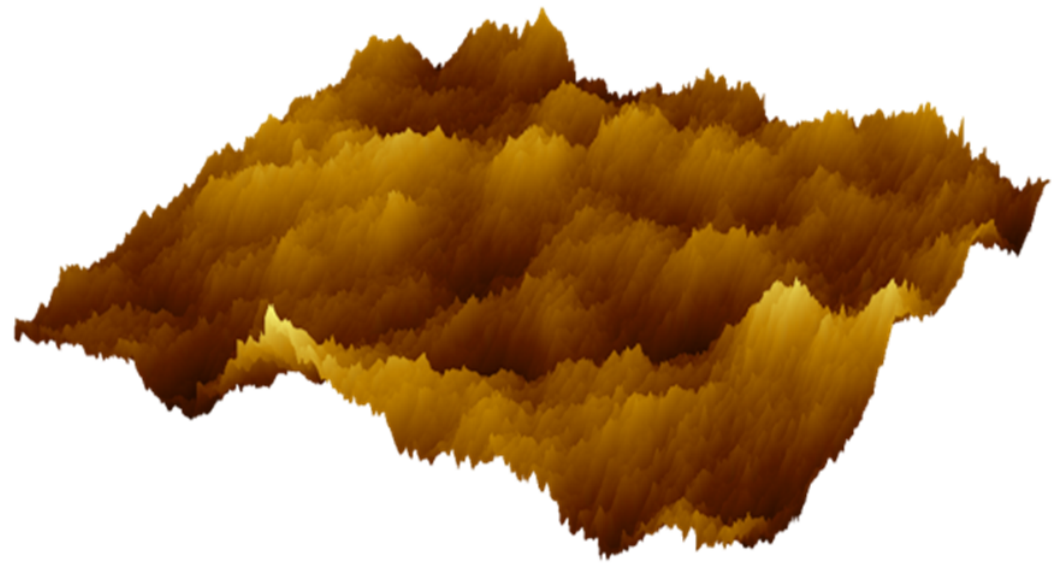

Supplementary Figure 11. AFM image of SNC-LSF with a scan size of 150 × 150 nm.

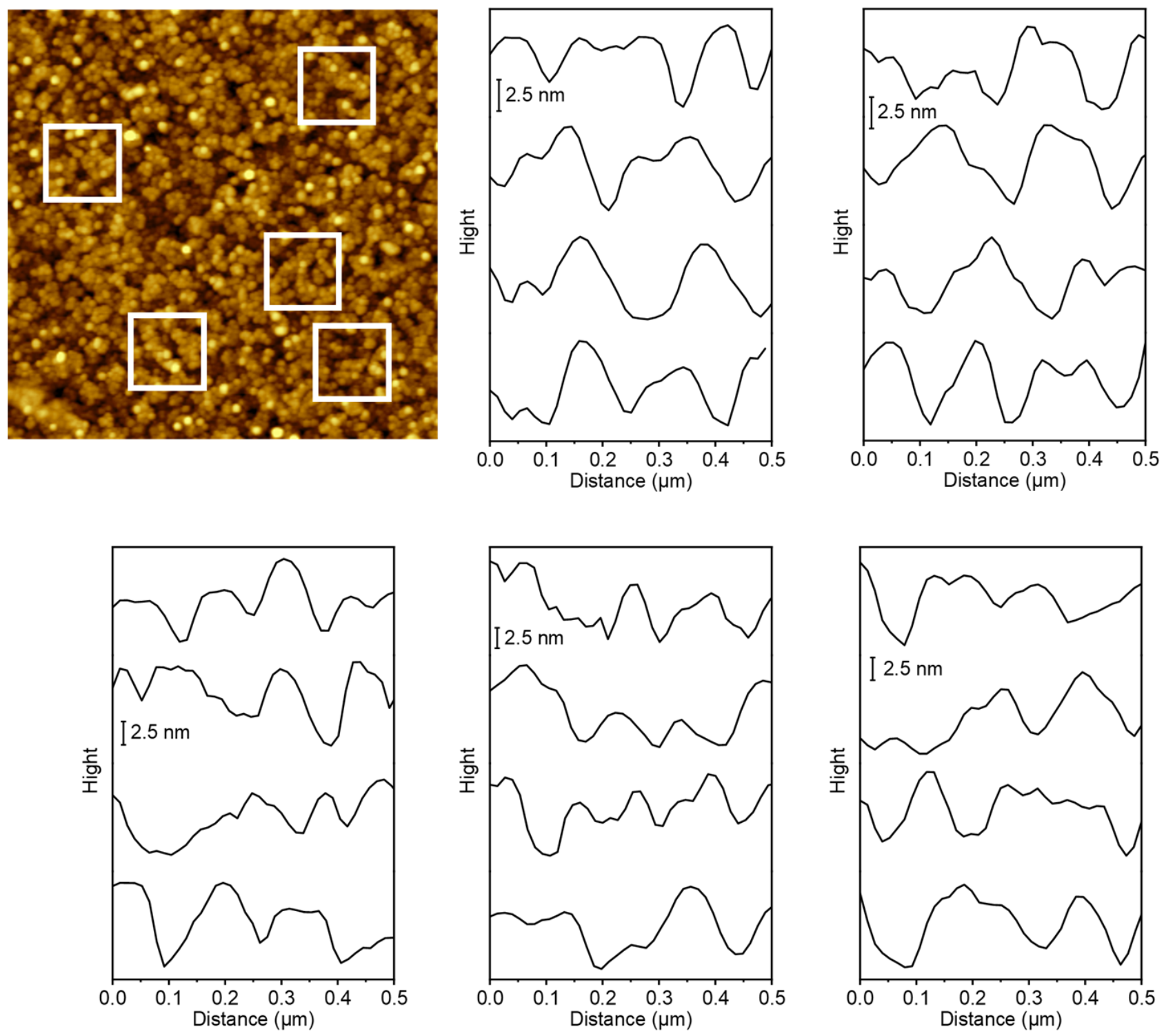

Supplementary Figure 12. Quantitative statistics of the nanopore density in SNC-LSF.

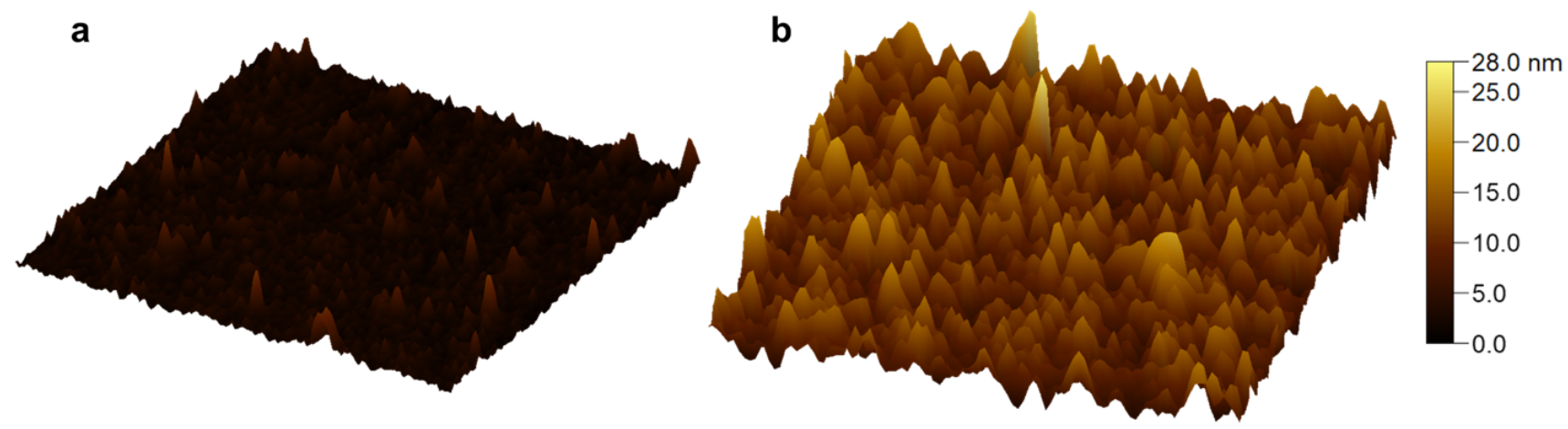

Supplementary Figure 13. AFM maps of (a) LCF and (b) SNC-LCF.

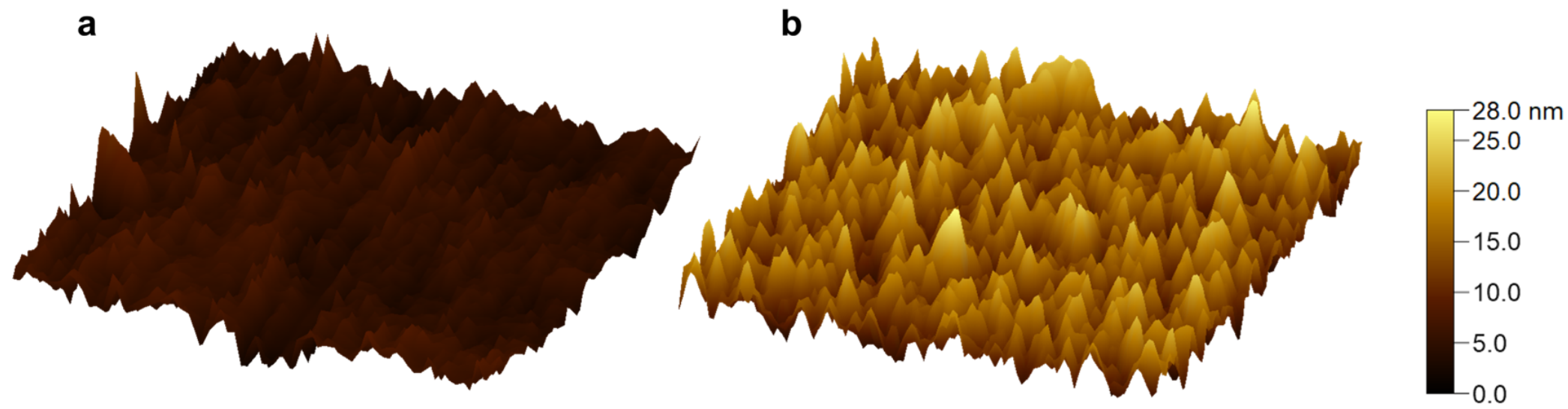

Supplementary Figure 14. AFM maps of (a) LBF and (b) SNC-LBF.

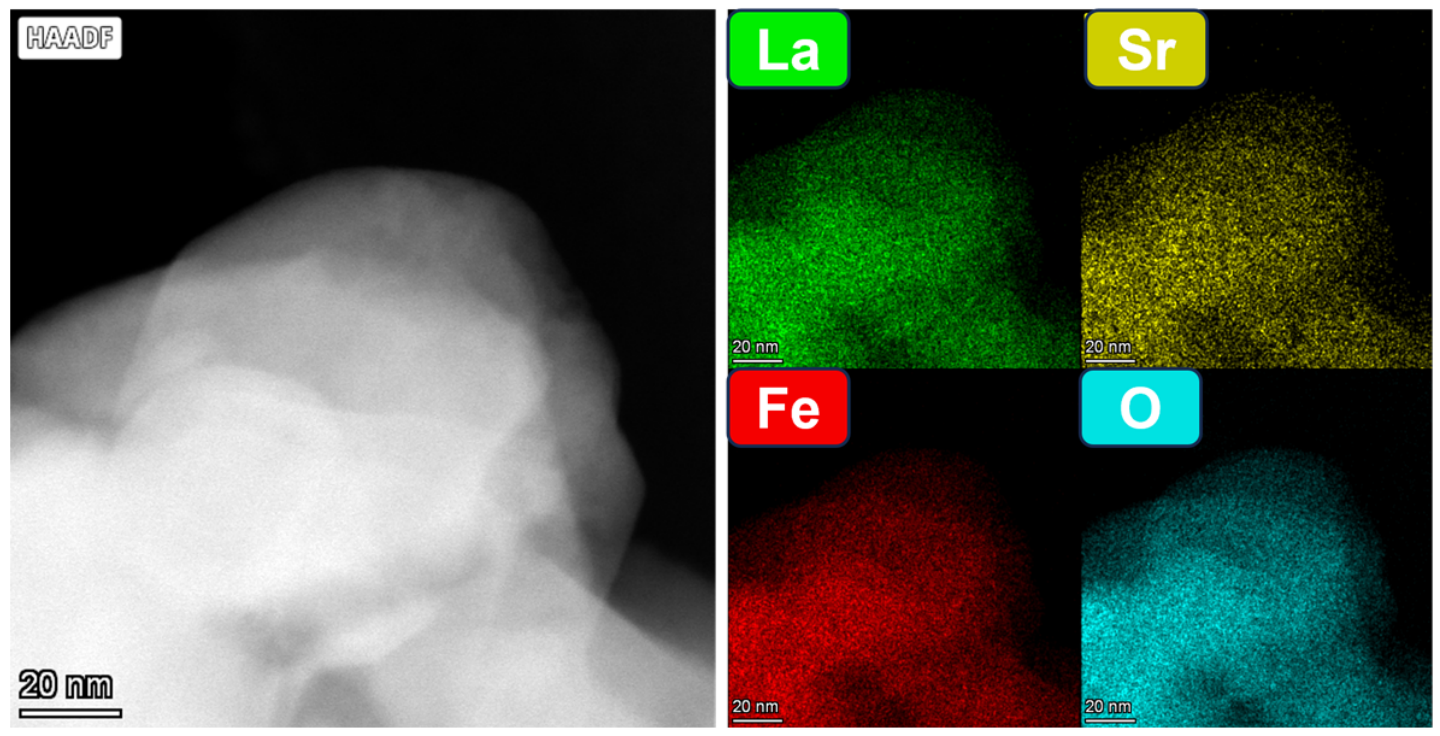

Supplementary Figure 15. HAADF-HRTEM image and STEM-EDS mapping of SNC-LSF.

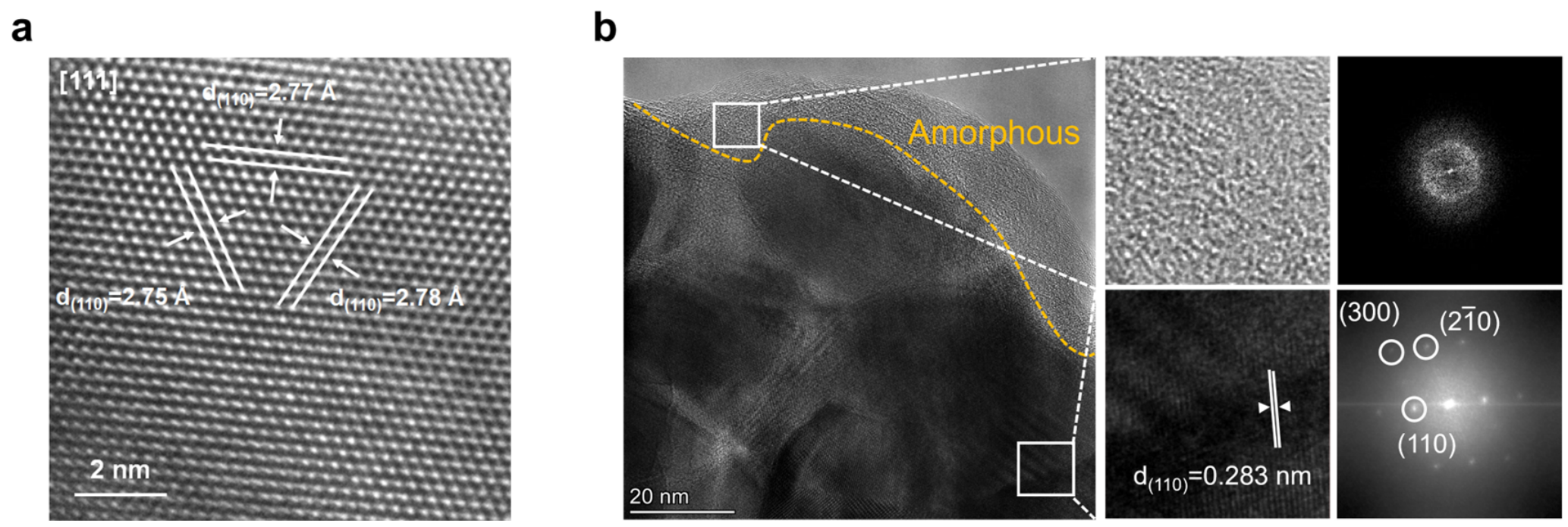

Supplementary Figure 16. (a) HRTEM image of LSF0.6. (b) HRTEM image of SNC-LSF and the corresponding FFT patterns.

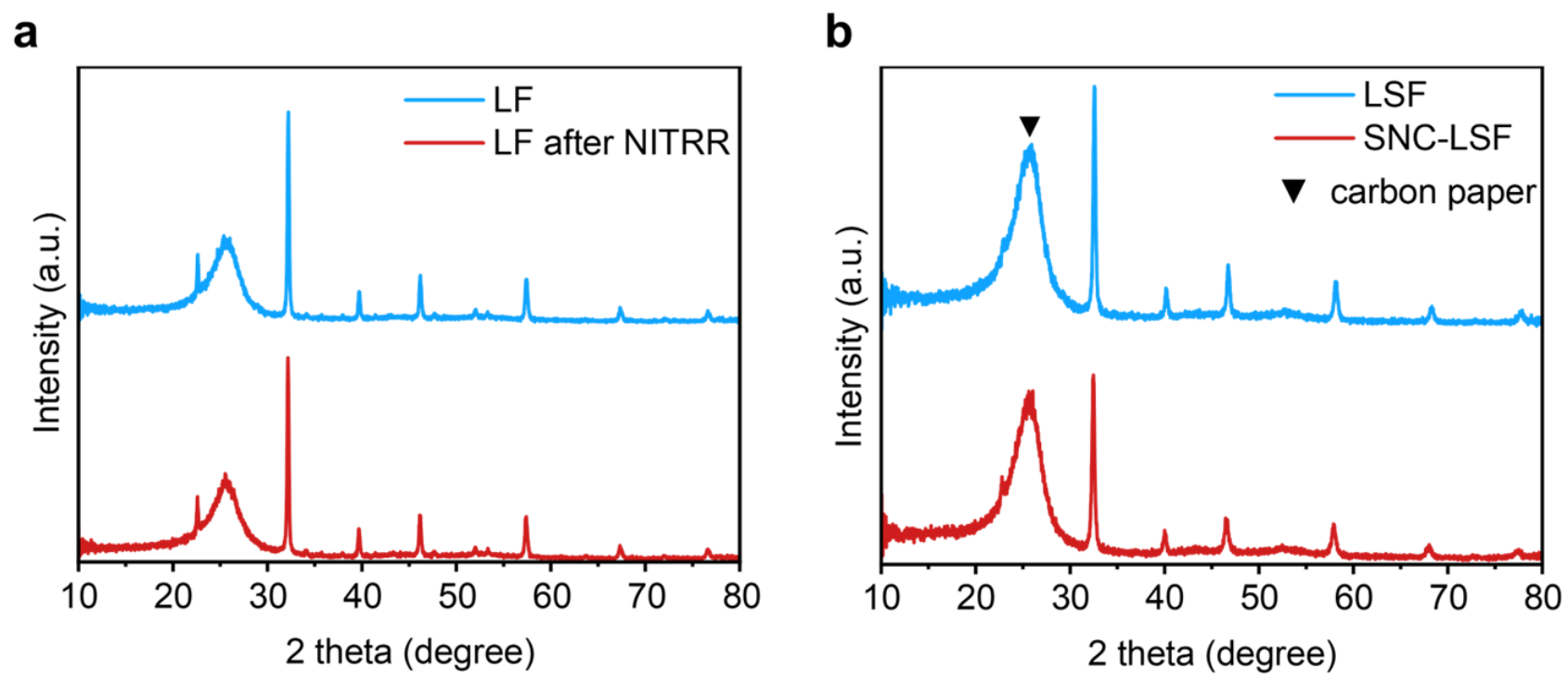

Supplementary Figure 17. (a) XRD patterns of LF before and after NITRR. XRD patterns show that there is no apparent variation in the peak pattern and position of LF before and after NITRR, confirming that the crystal structure of LF does not change during NITRR. (b) XRD patterns of LSF and SNC-LSF.

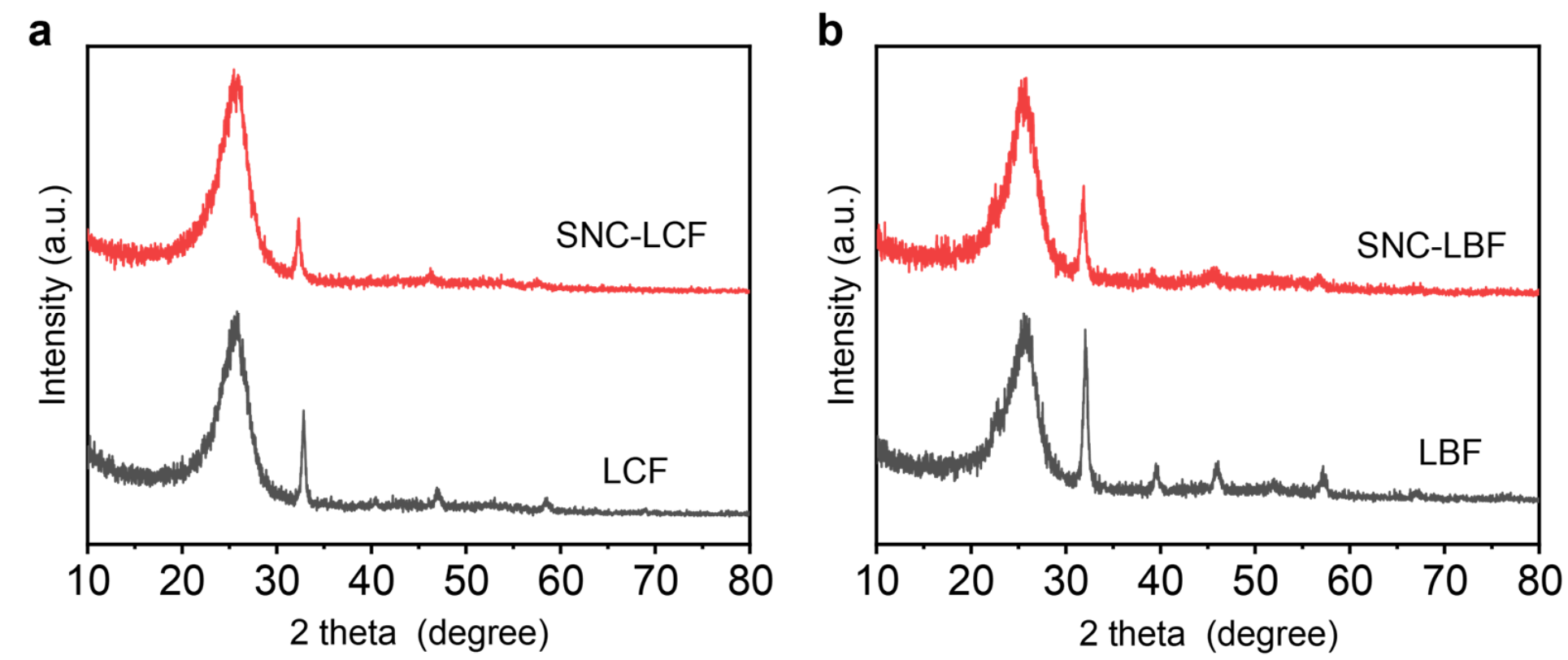

Supplementary Figure 18. XRD patterns of (a) LCF and SNC-LCF, (b) LBF and SNC-LBF.

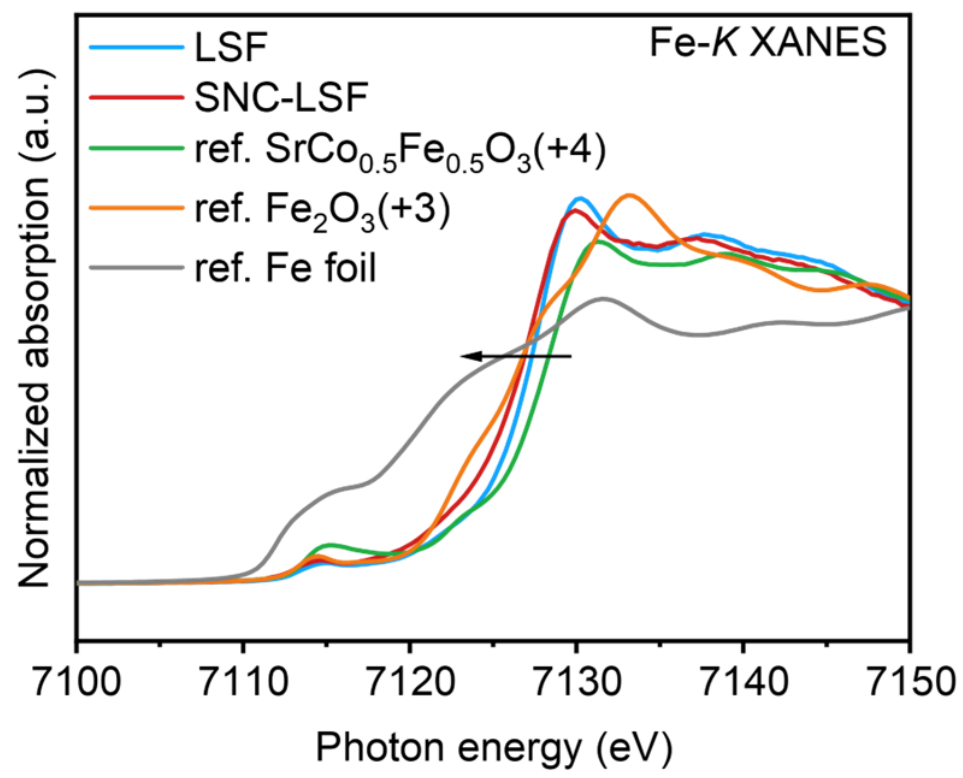

Supplementary Figure 19. Fe *K*-edge XANES spectra of LSF, SNC-LSF and several references.

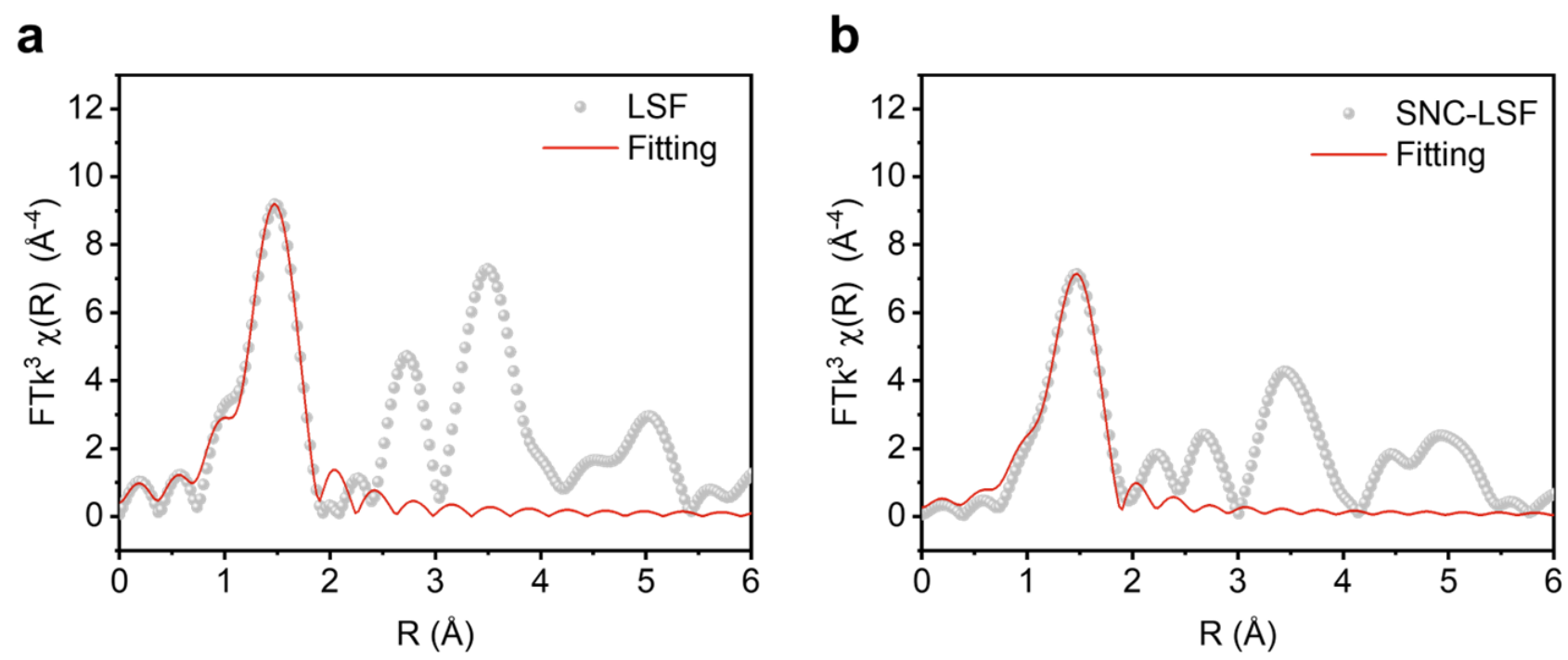

Supplementary Figure 20. R space fitting curves at Fe *K*-edge of (a) LSF and (b) SNC-LSF. Least square EXAFS fitting was performed to quantificational extract the local atomic structure parameters of LSF and SNC-LSF. It was found that the fitting curves match quite well with the experiment spectra. The fitting structural parameters are displayed in **Supplementary Table 1**.

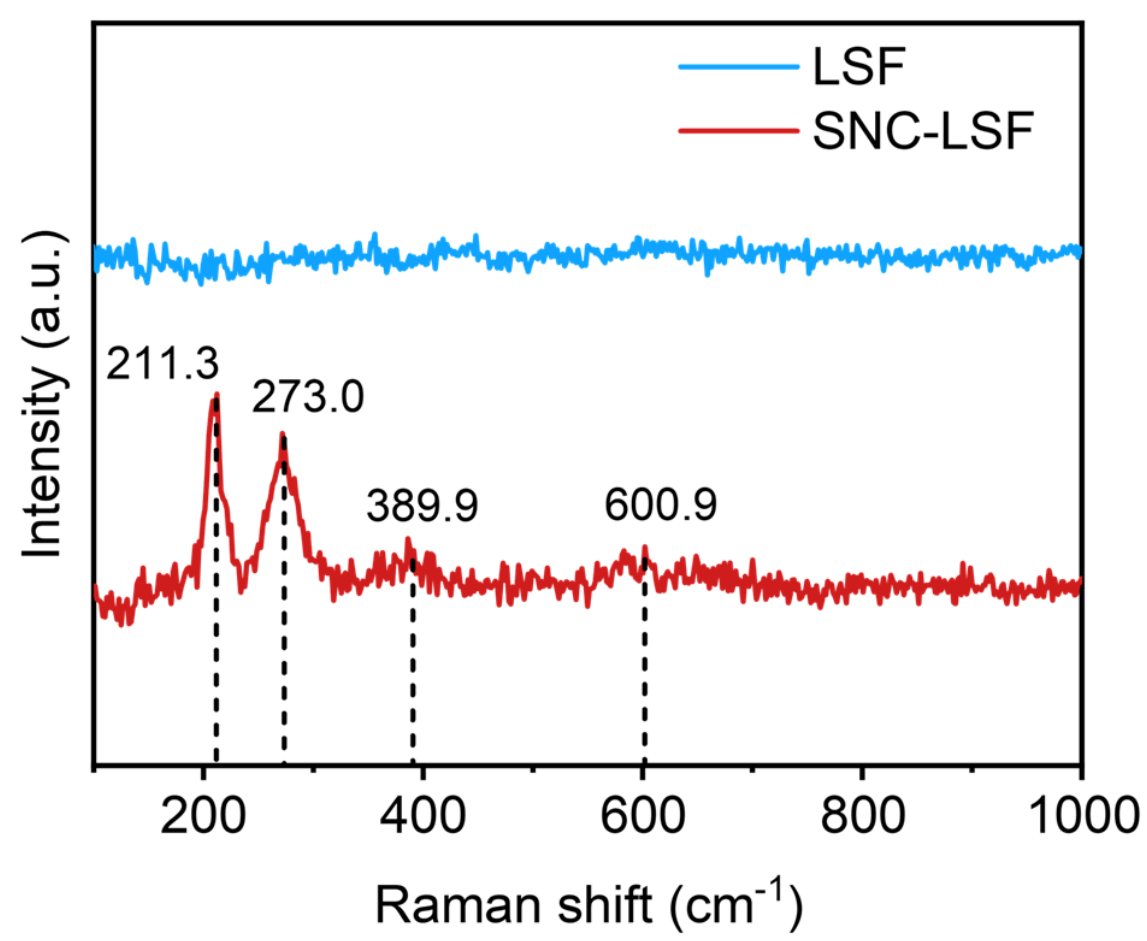

Supplementary Figure 21. Raman spectra of LSF and SNC-LSF.

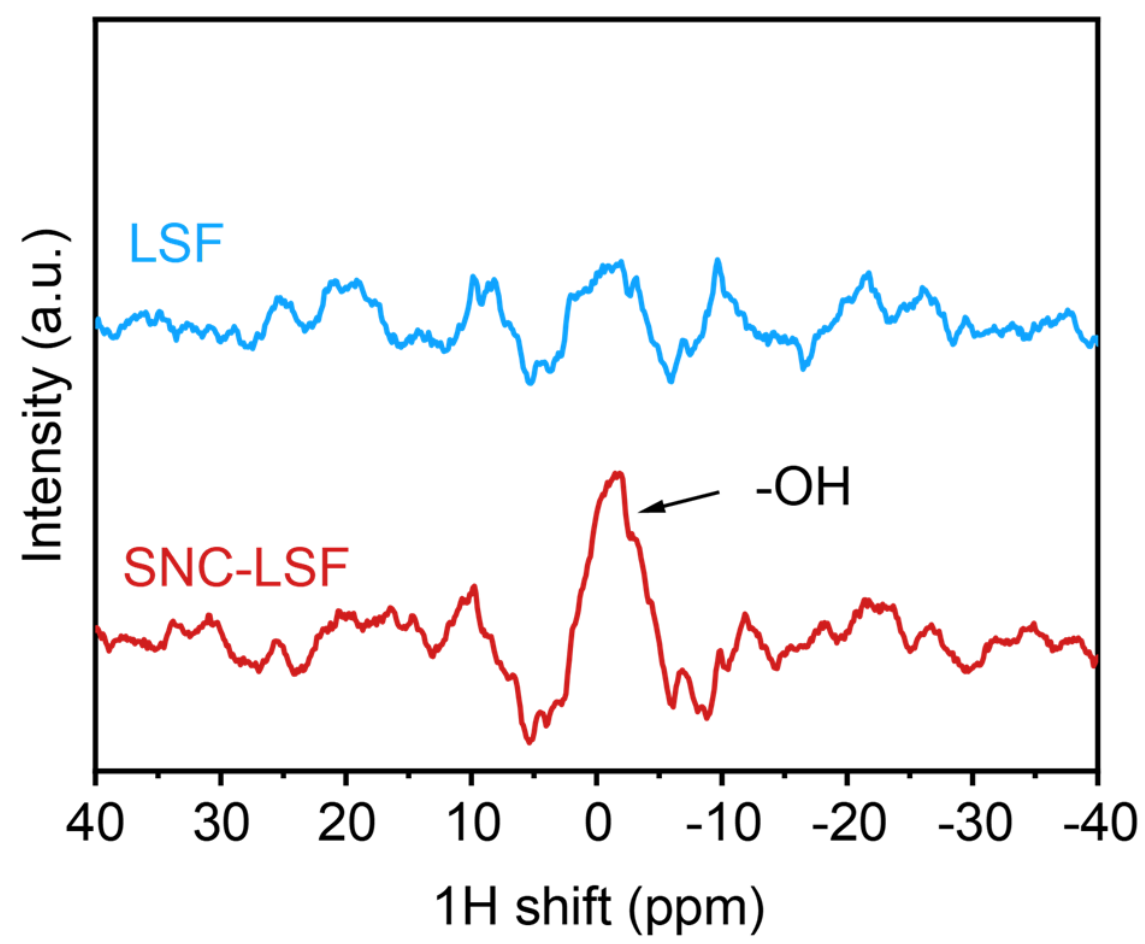

Supplementary Figure 22. Solid-state ^{1}H-NMR of LSF and SNC-LSF.

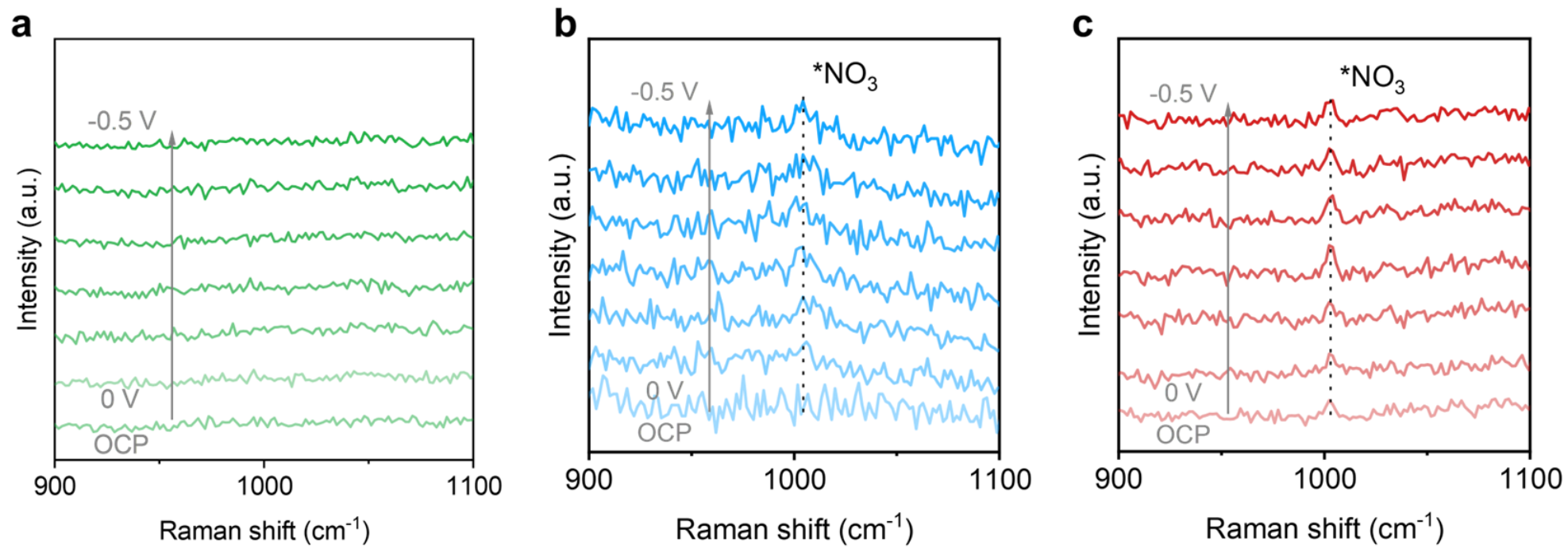

Supplementary Figure 23. *In-situ* Raman spectra at the range of 900-1100 cm^{-1} for (a) LF, (b) LSF and (c) SNC-LSF.

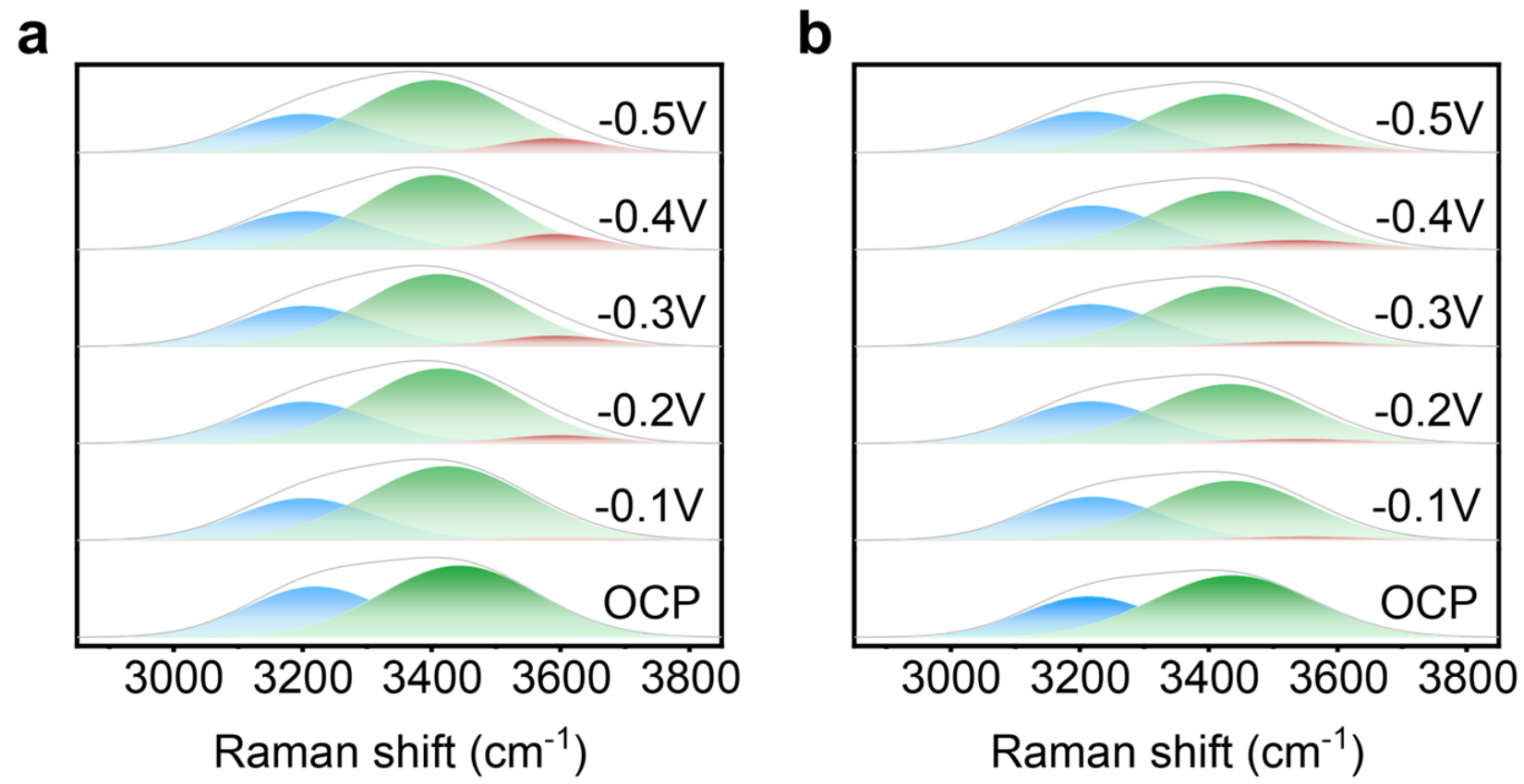

Supplementary Figure 24. *In-situ* Raman spectra at the range of 2800-3800 cm^{-1} for (a) LF and (b) LSF.

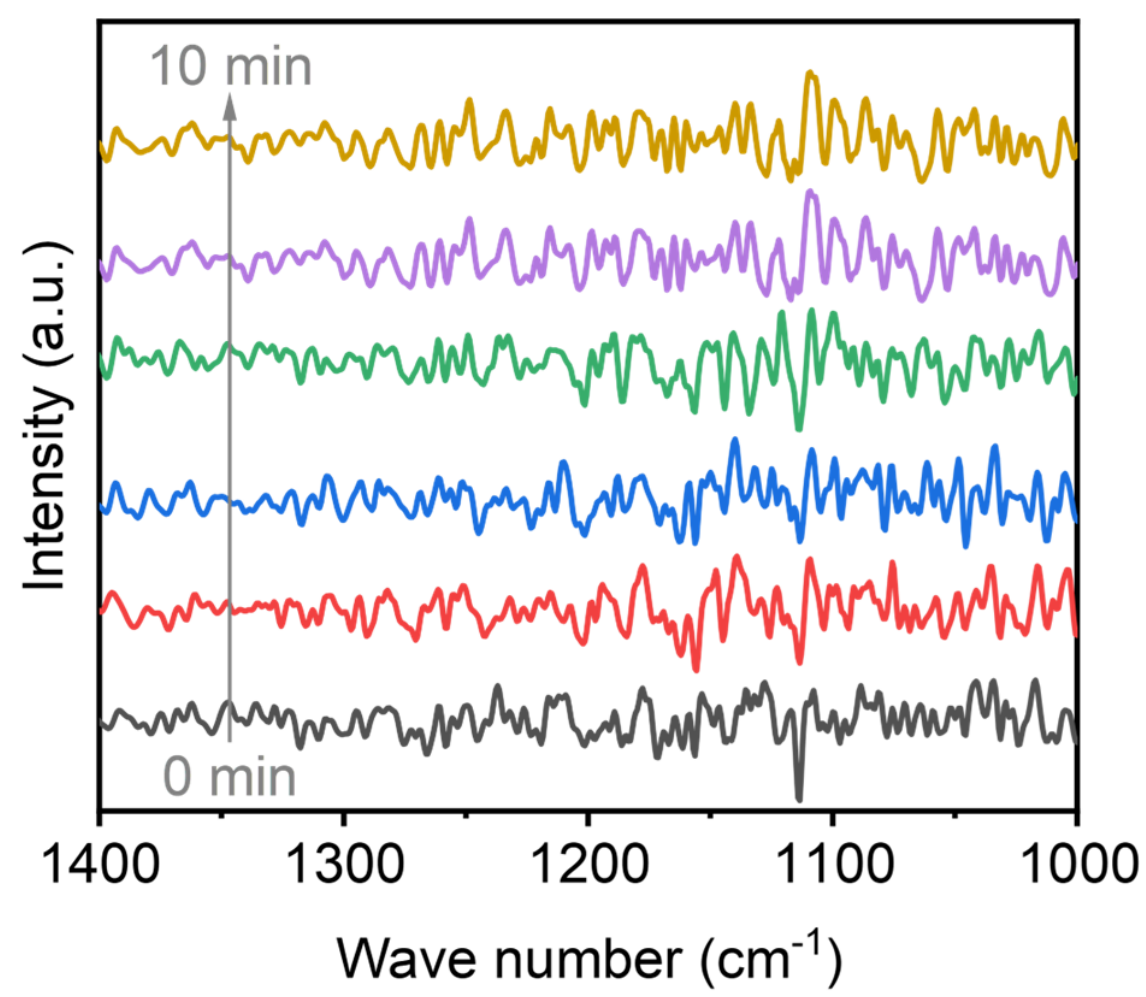

Supplementary Figure 25. Time-resolved *in-situ* ATR-SEIRAS spectra collected on LF at -0.6 V vs. RHE.

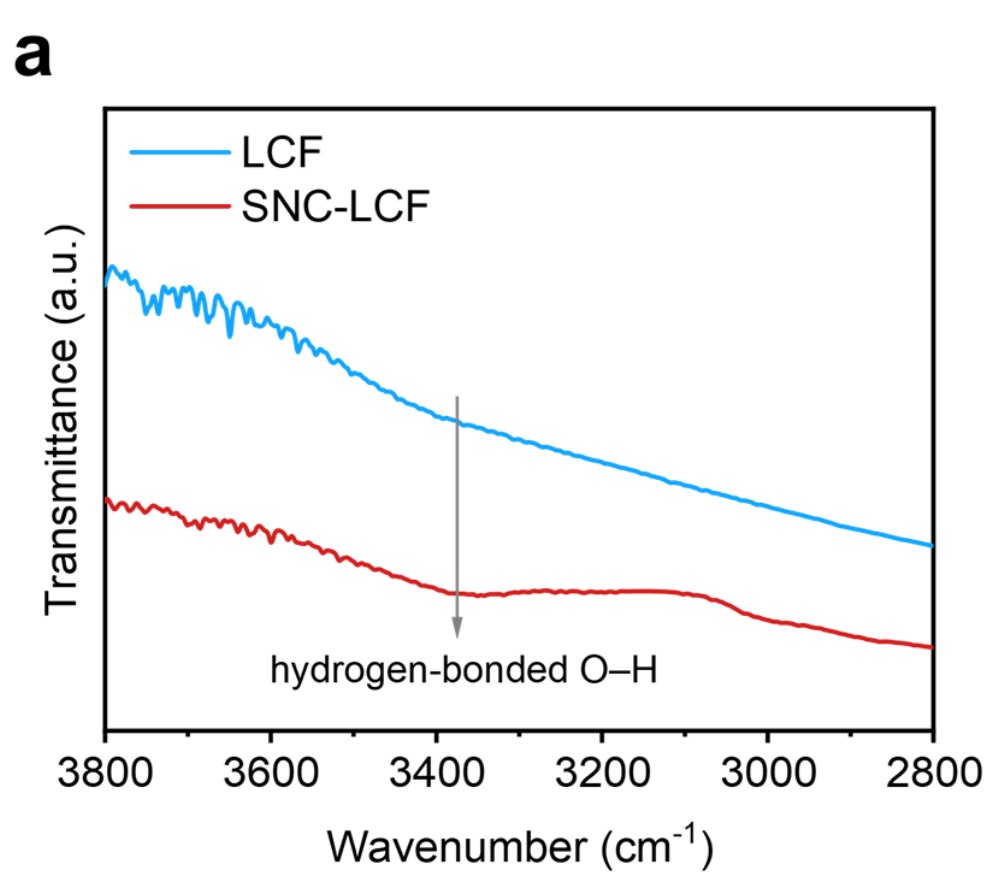

Supplementary Figure 26. FTIR spectra of (a) LCF and SNC-LCF, (b) LBF and SNC-LBF.

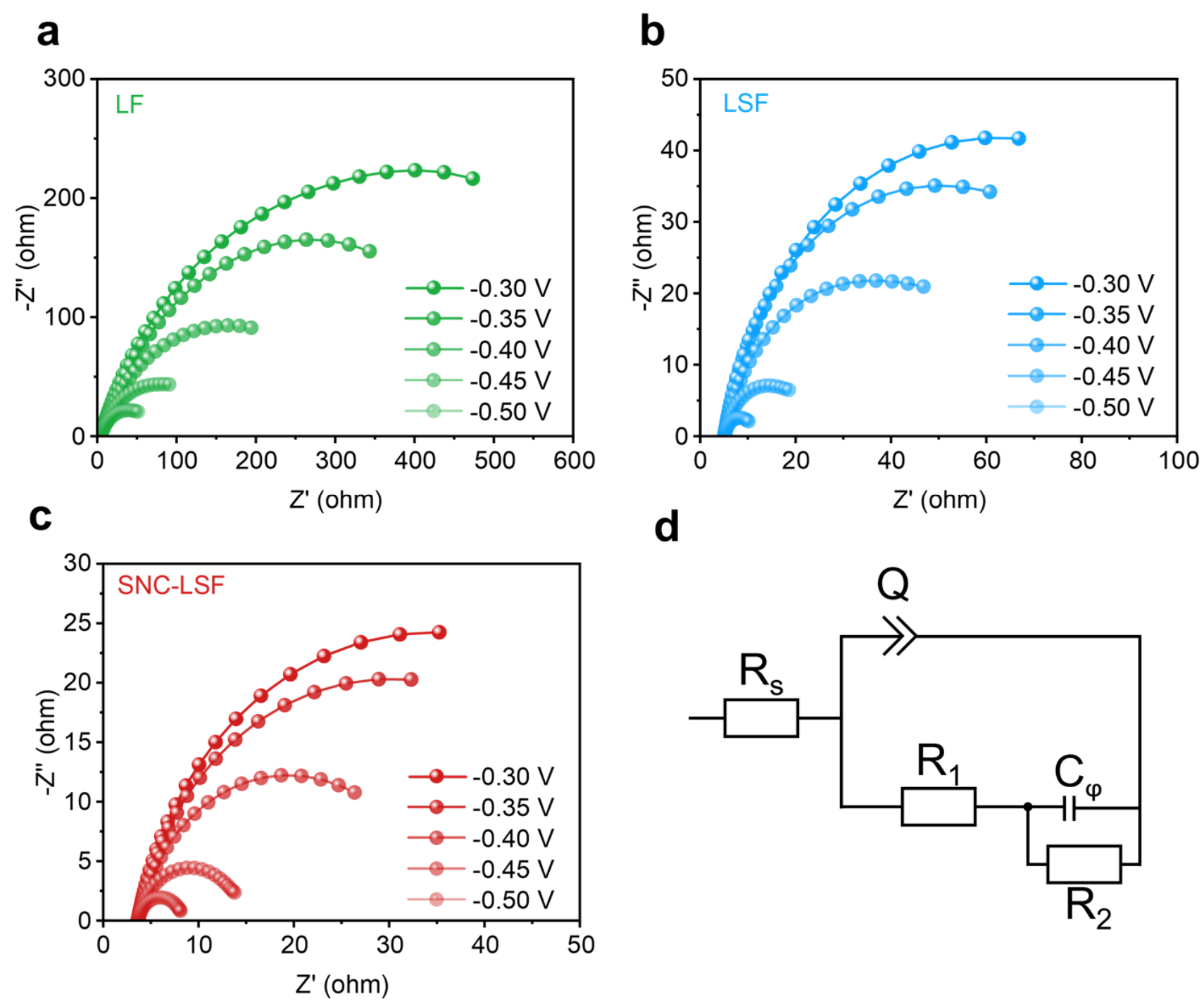

Supplementary Figure 27. Nyquist plots for the (a) LF, (b) LSF and (c) SNC-LSF catalysts at various potentials in 1.0 M KOH solution without NO_3^- addition. (d) The equivalent circuit for the simulation.

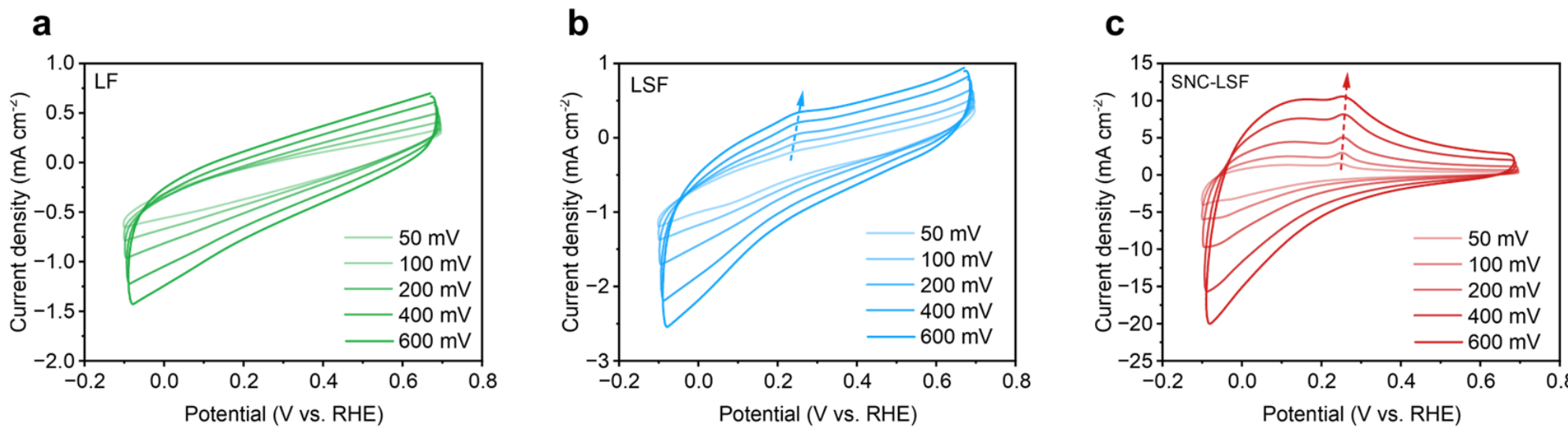

Supplementary Figure 28. CV curves of (a) LF, (b) LSF and (c) SNC-LSF under different scan rates.

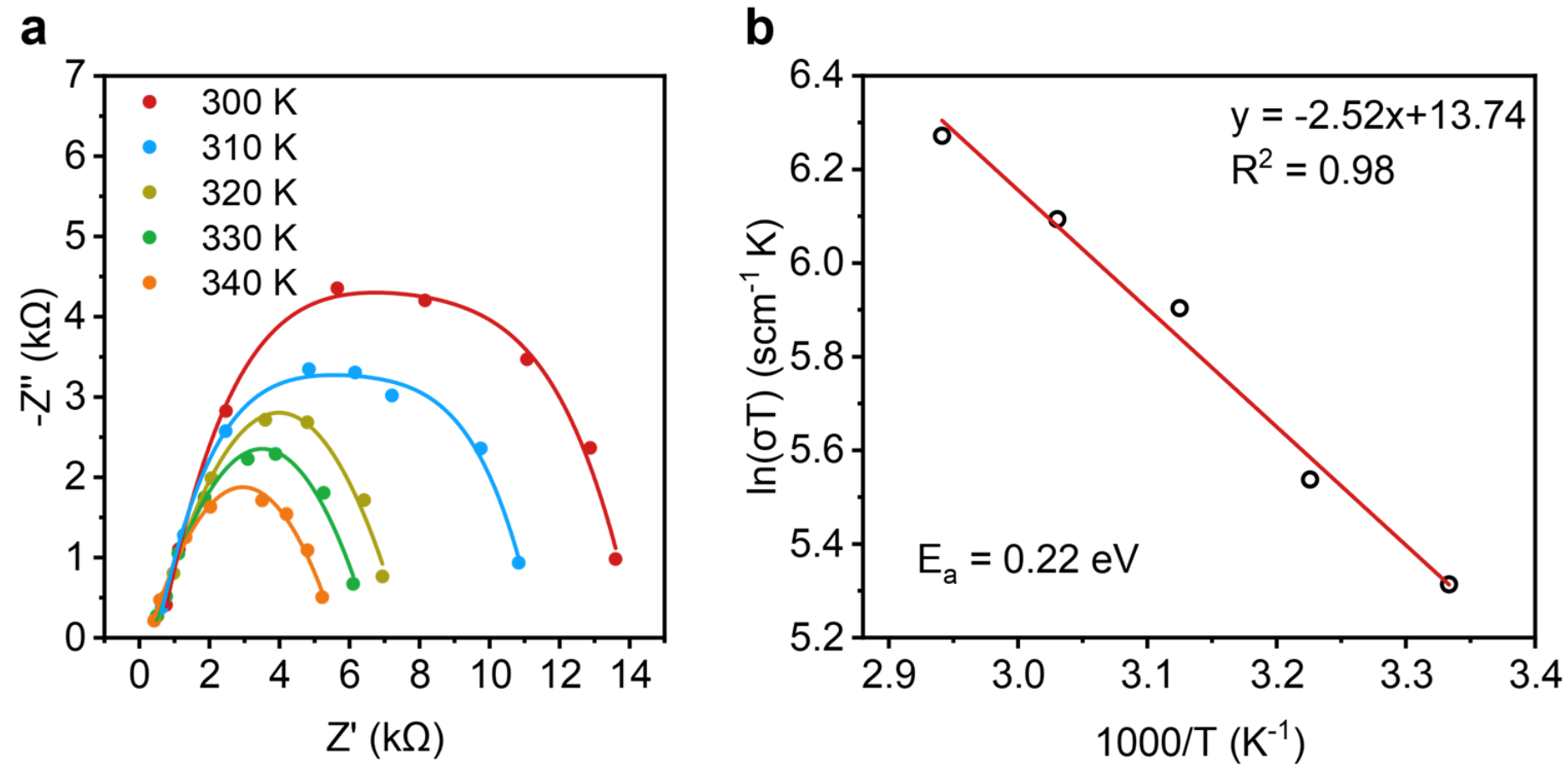

Supplementary Figure 29. Temperature-dependent EIS measurements. (a) Nyquist plots of SNC-LSF measured under 100% RH at different temperatures. (b) The fitted Arrhenius plots based on temperature-dependent EIS measurements on SNC-LSF.

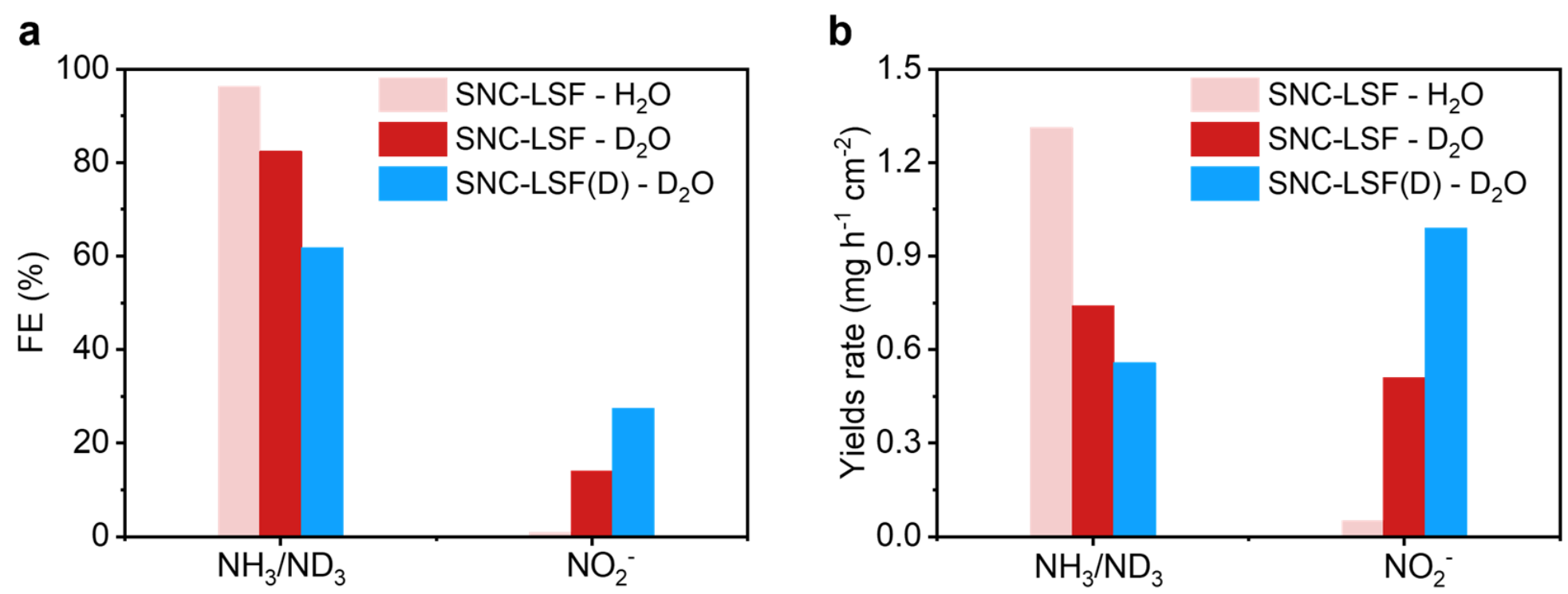

Supplementary Figure 30. Kinetic isotope effect. NITRR performance of SNC-LSF and SNC-LSF(D) recorded in both H_2O and D_2O electrolytes with the addition of 8 mM KNO_3 containing 0.1 M KOH or KOD. (a) Faradic efficiency (b) yields rate of NH_3/ND_3 and NO_2^- of SNC-LSF and SNC-LSF(D) at -0.4 V vs. RHE.

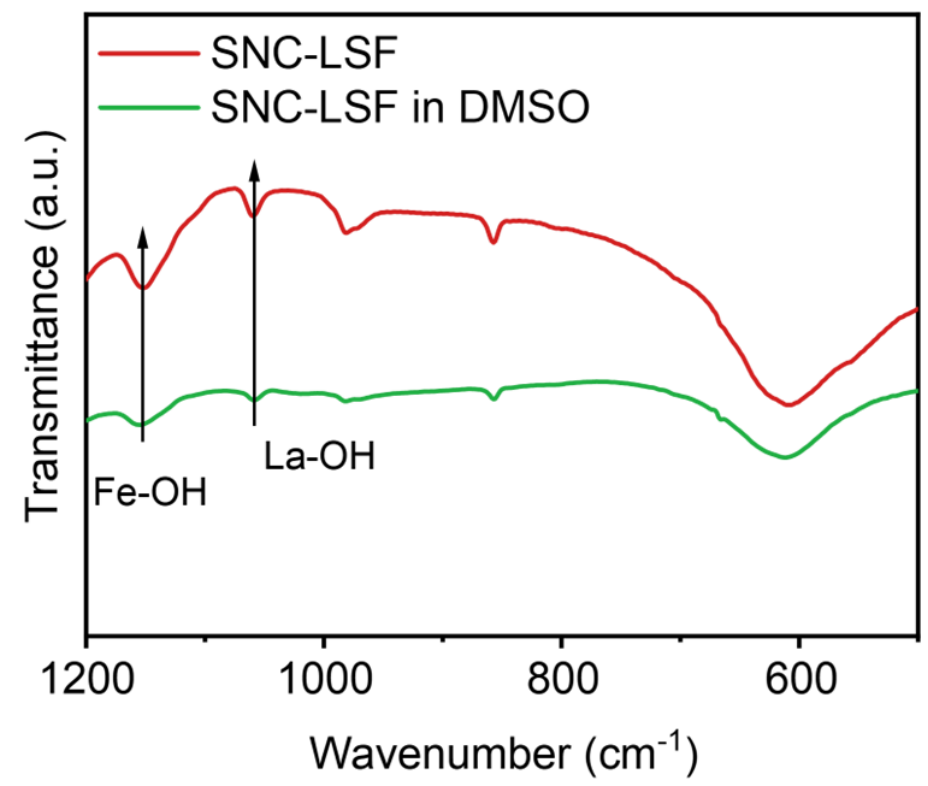

Supplementary Figure 31. FTIR spectra of SNC-LSF and SNC-LSF in DMSO.

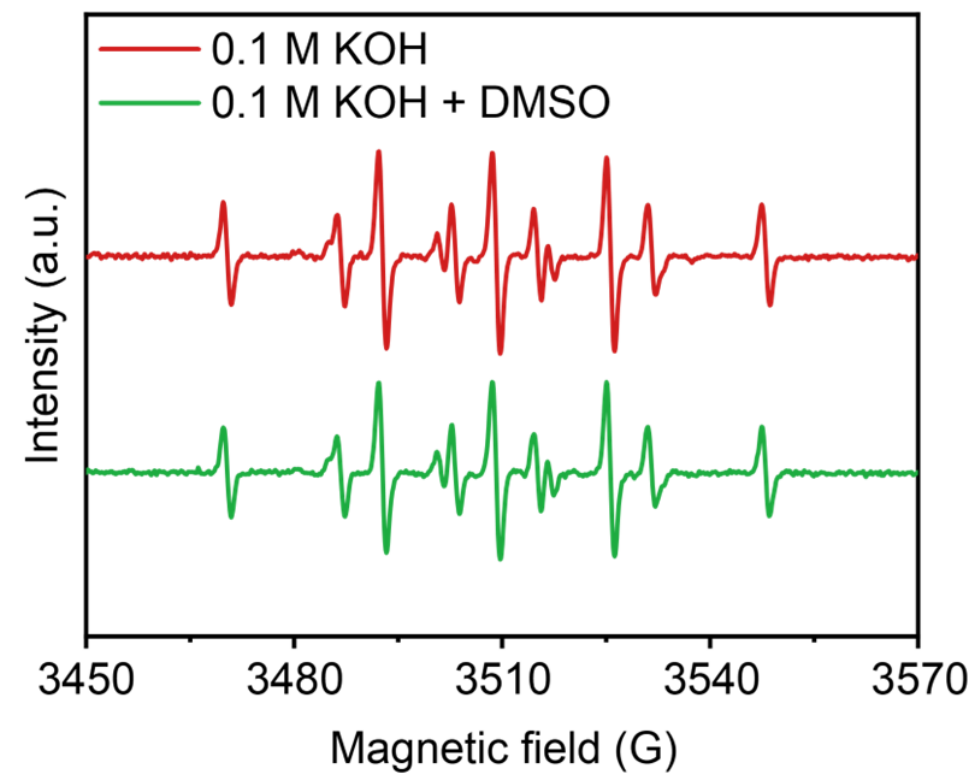

Supplementary Figure 32. ESR spectra of the pristine electrolyte, the electrolyte obtained after 10-min electrocatalysis SNC-LSF in 0.1 M KOH with and without DMSO.

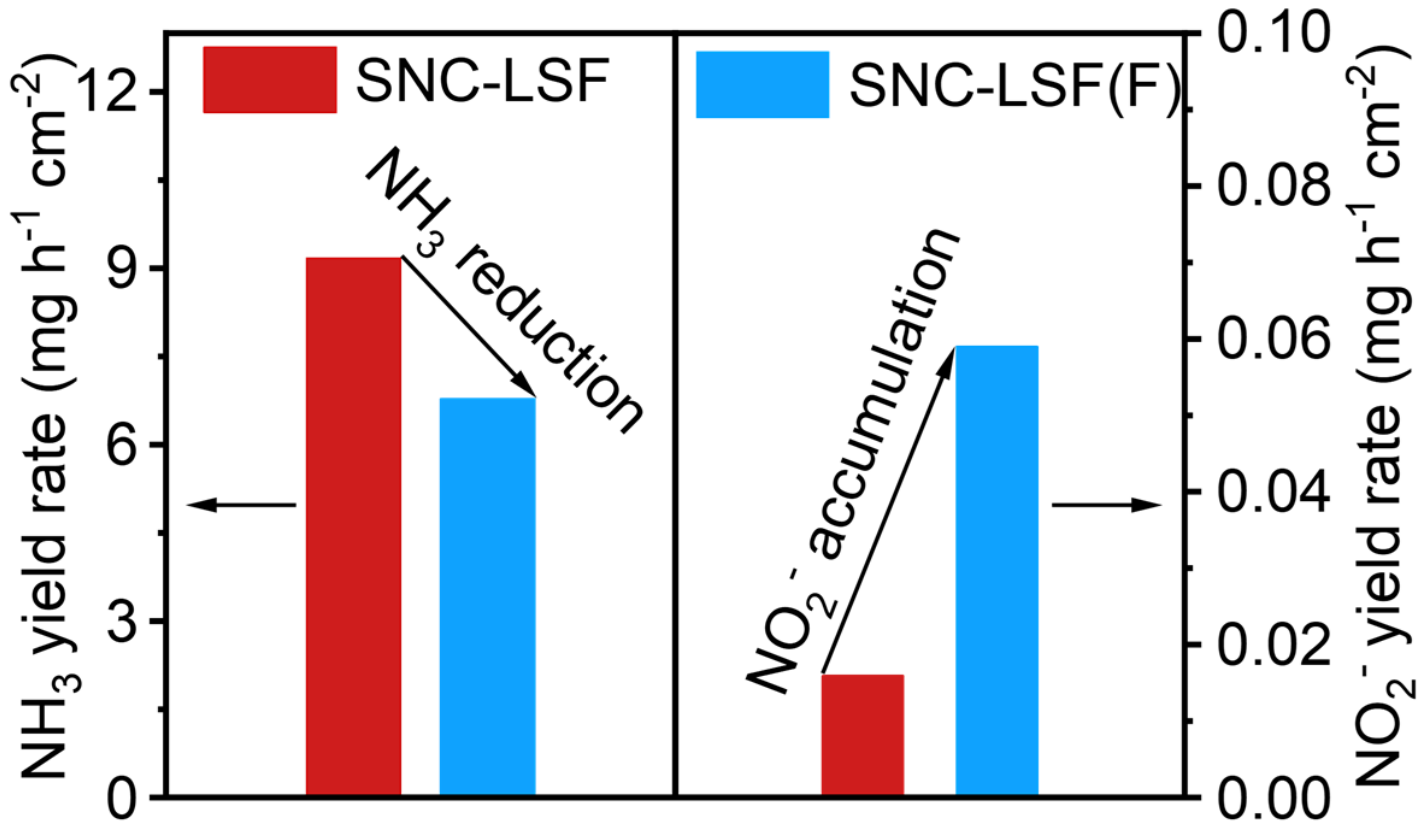

Supplementary Figure 33. NH_3 and NO_2^- yield rate of SNC-LSF and SNC-LSF(F) under -0.4 V vs. RHE in 1 M KOH + 0.1 M KNO_3.

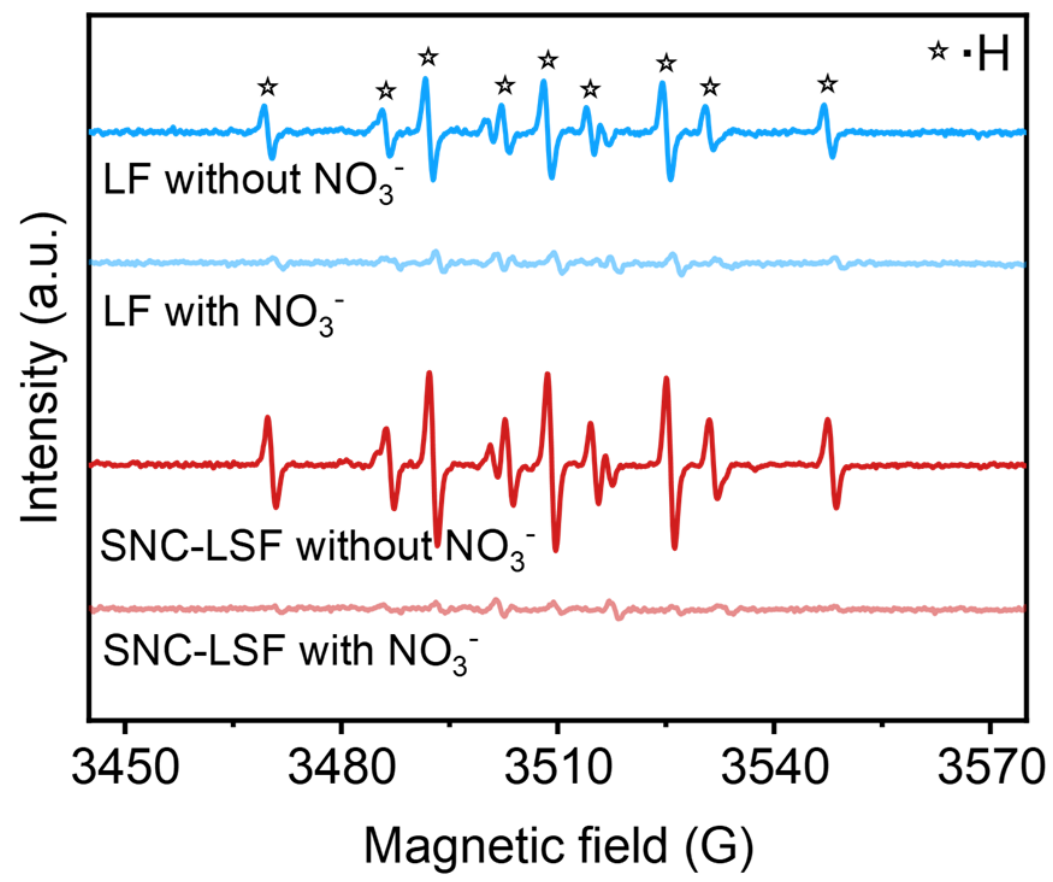

Supplementary Figure 34. ESR spectra for the investigation of hydrogen radicals. ESR spectra of the pristine electrolyte, the electrolyte obtained after 10-min electrocatalysis on LF and SNC-LSF in 0.1 M KOH without NO_3^-, and the electrolyte obtained after 10-min electrocatalysis on LF and SNC-LSF in 0.1 M KOH with 8 mM NO_3^- under argon using DMPO as the ·H-trapping reagent.

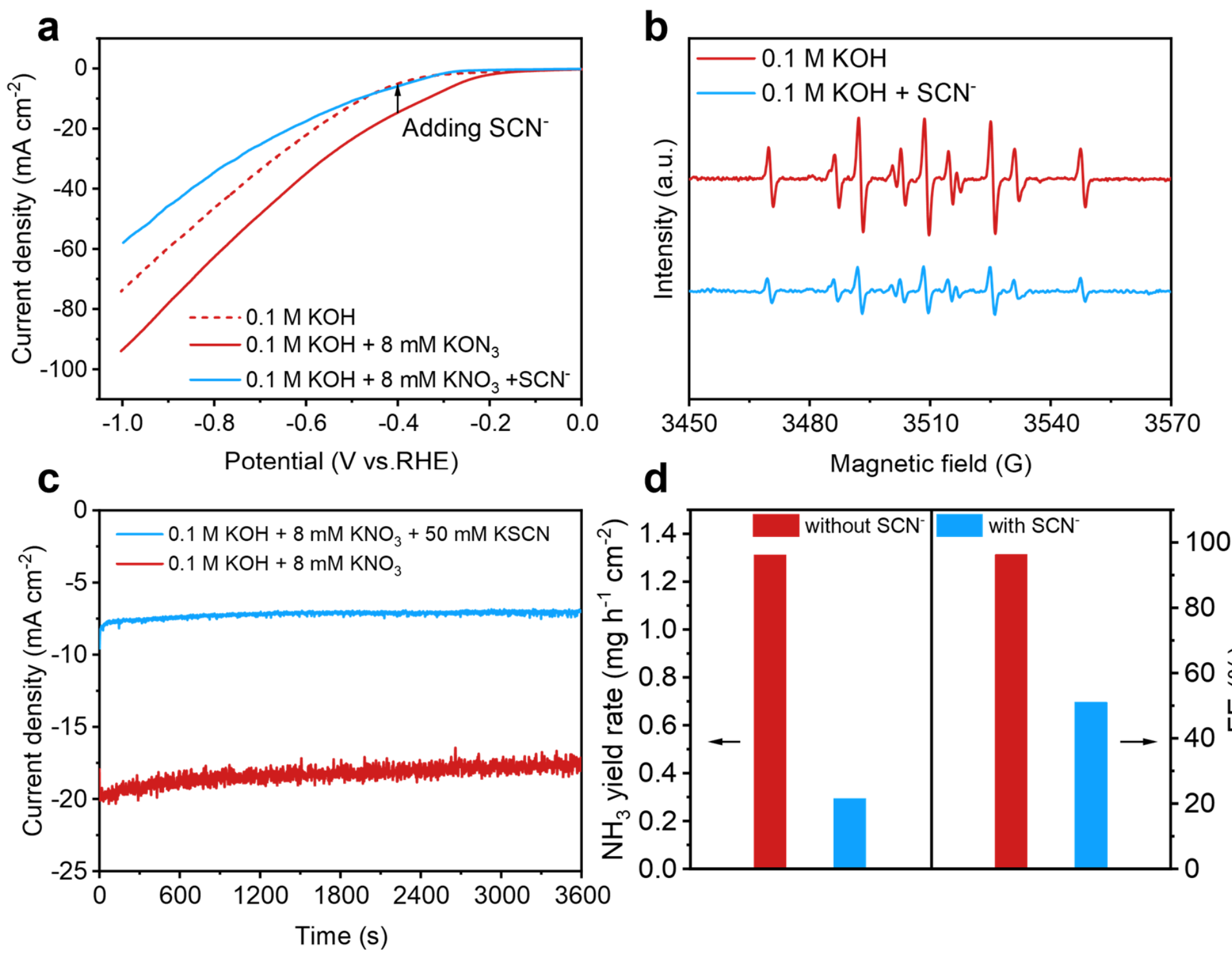

Supplementary Figure 35. (a) LSV curves for poisoning experiment on SNC-LSF. (b) ESR spectra of the pristine electrolyte, the electrolyte obtained after 10-min electrocatalysis SNC-LSF in 0.1 M KOH with and without SCN^-. (c) I-t curves of SNC-LSF under -0.4 V vs. RHE with and without SCN^-. (d) NH_3 yield rate and FE_{NH3} of SNC under -0.4 V vs. RHE with and without SCN^-.

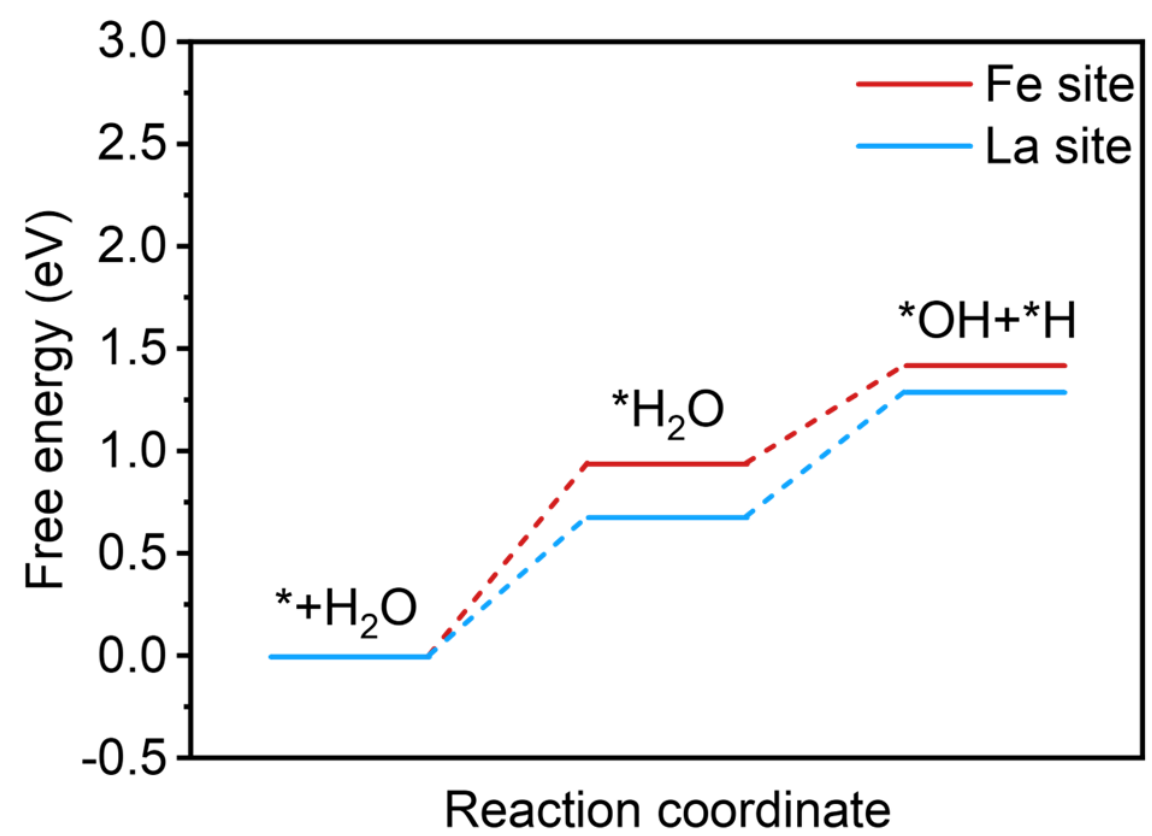

Supplementary Figure 36. Free energy of H_2O dissociation on different active sites of LF.

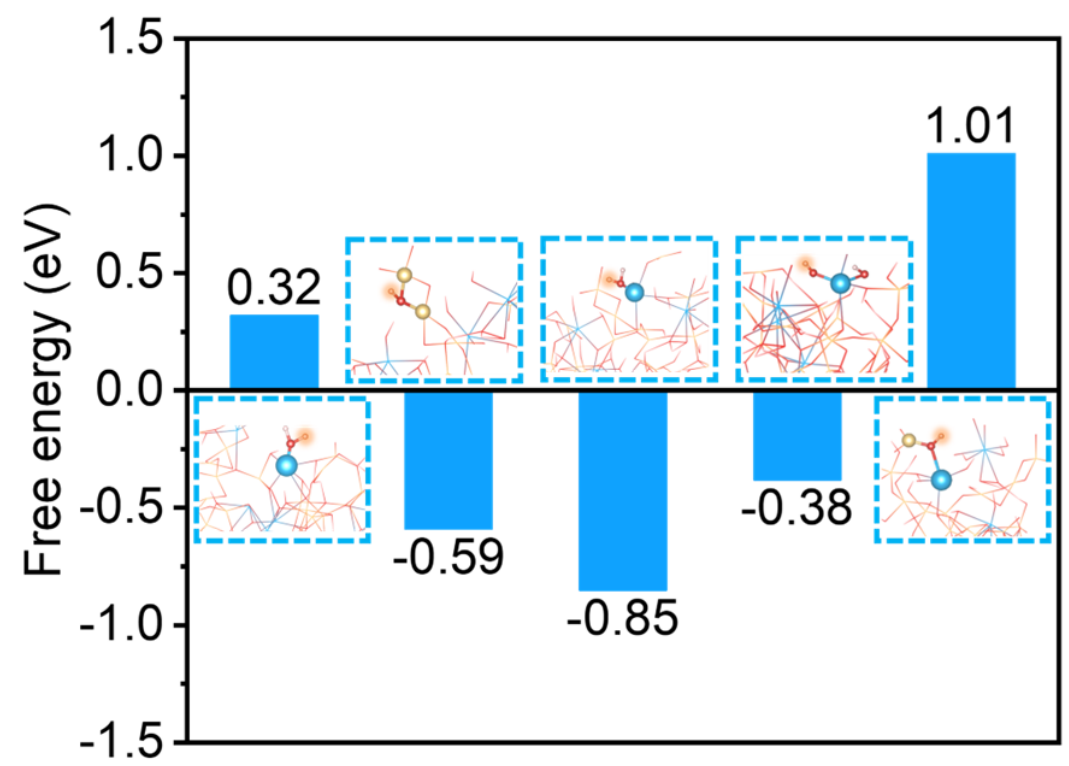

Supplementary Figure 37. Gibbs free energy diagram for hydrogen adsorption at different catalytic sites on SNC-LSF.

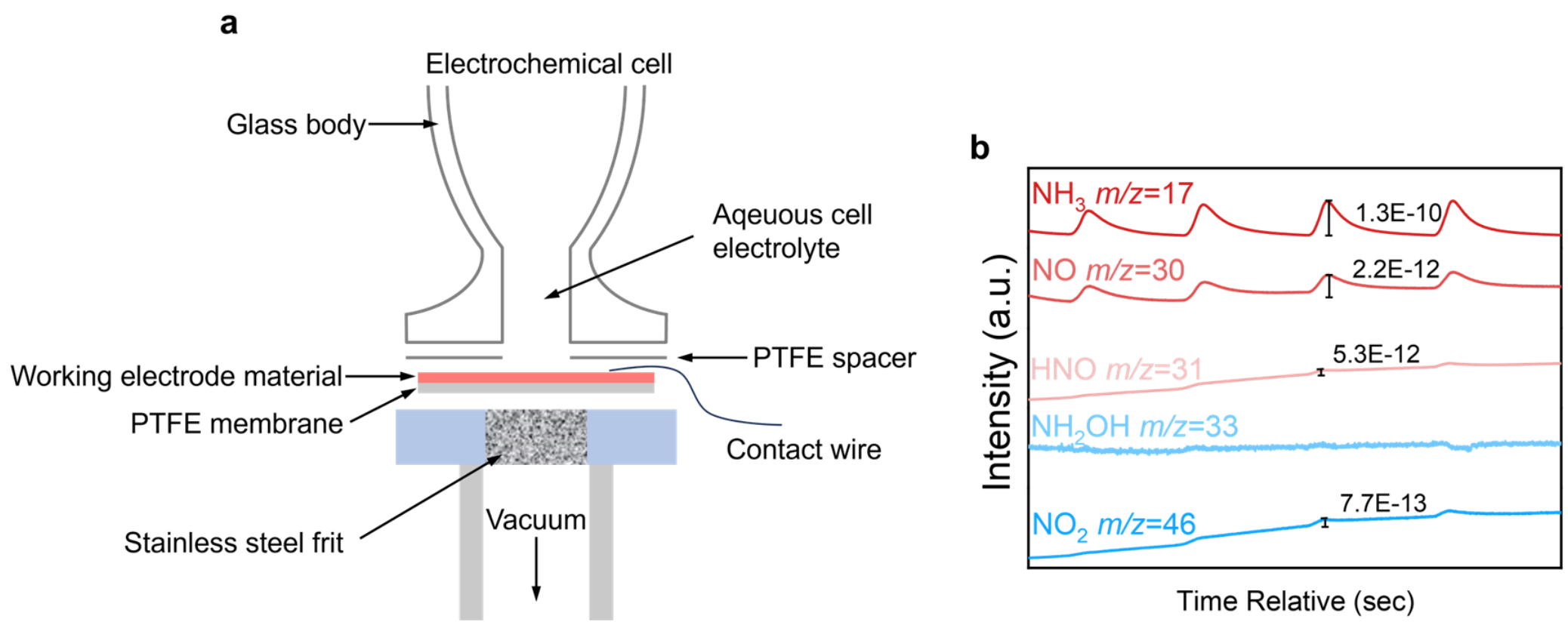

Supplementary Figure 38. (a) schematic of the DEMS electrochemical cell. (b) DEMS measurements of NITRR over LF.

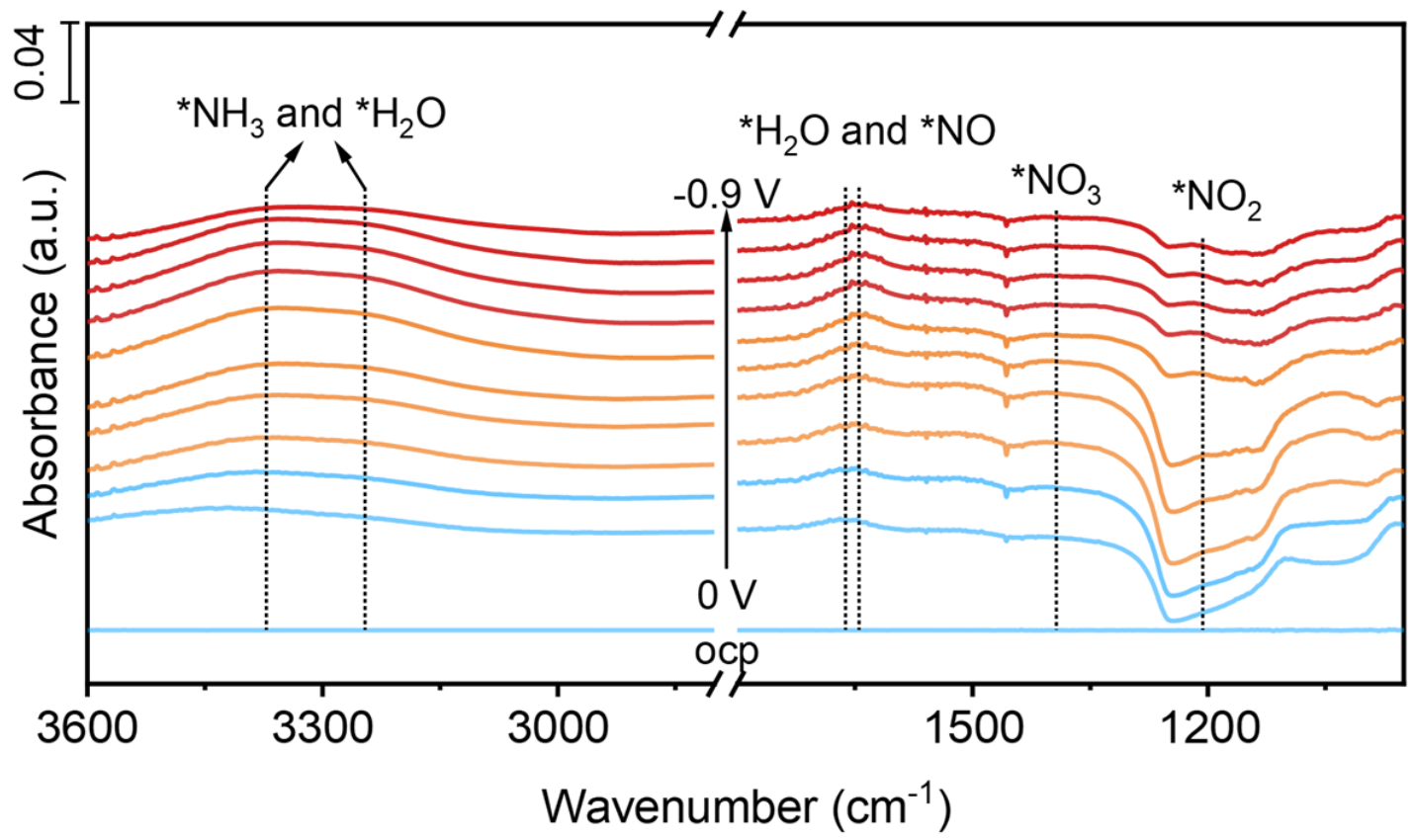

Supplementary Figure 39. *In-situ* ATR-SEIRAS spectra collected on LF under the potential from OCP to -0.9 V vs. RHE.

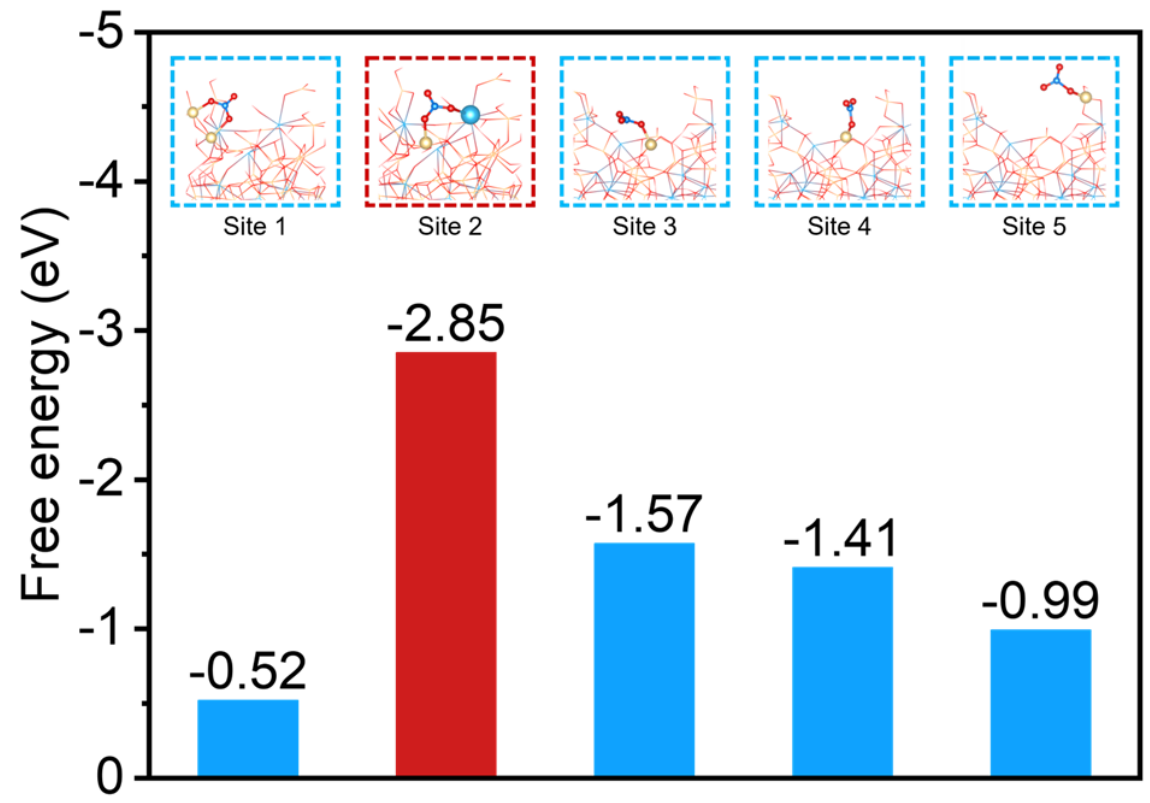

Supplementary Figure 40. The adsorption energy of $*NO_3$ on different sites calculated by DFT.

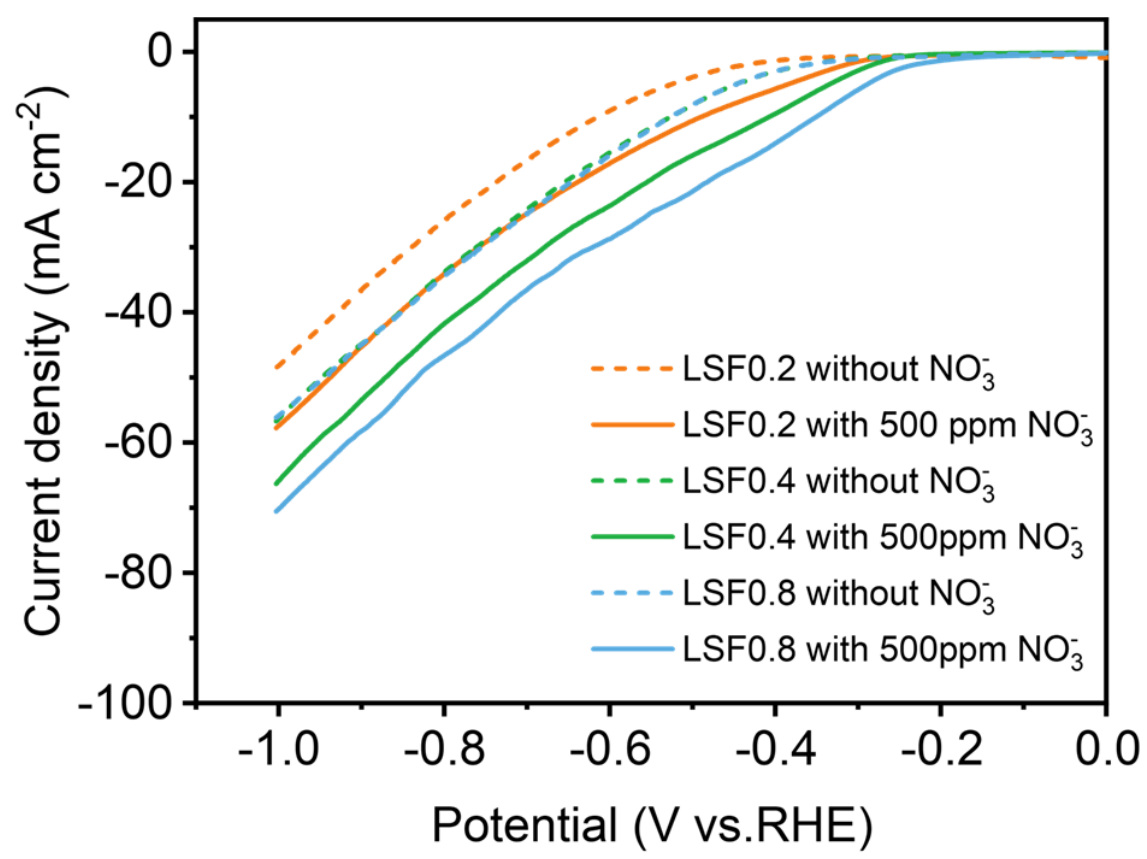

Supplementary Figure 41. LSV polarization curves of LSF0.2, LSF0.4 and LSF0.8 in 0.1 M KOH with and without 8 mM KNO_3.

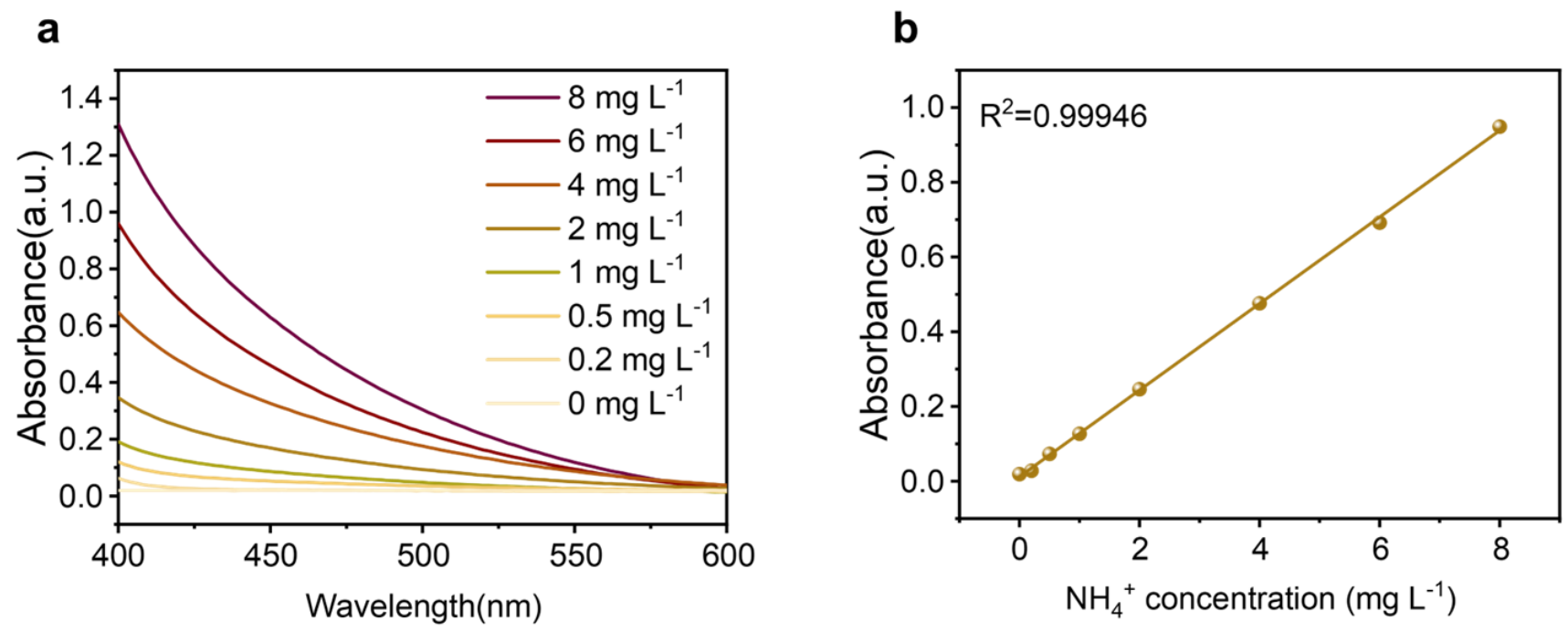

Supplementary Figure 42. (a) UV-Vis curves and (b) corresponding calibration curve of Nessler assays with known NH_3 concentrations (the absorbance was measured at a wavelength of 420 nm).

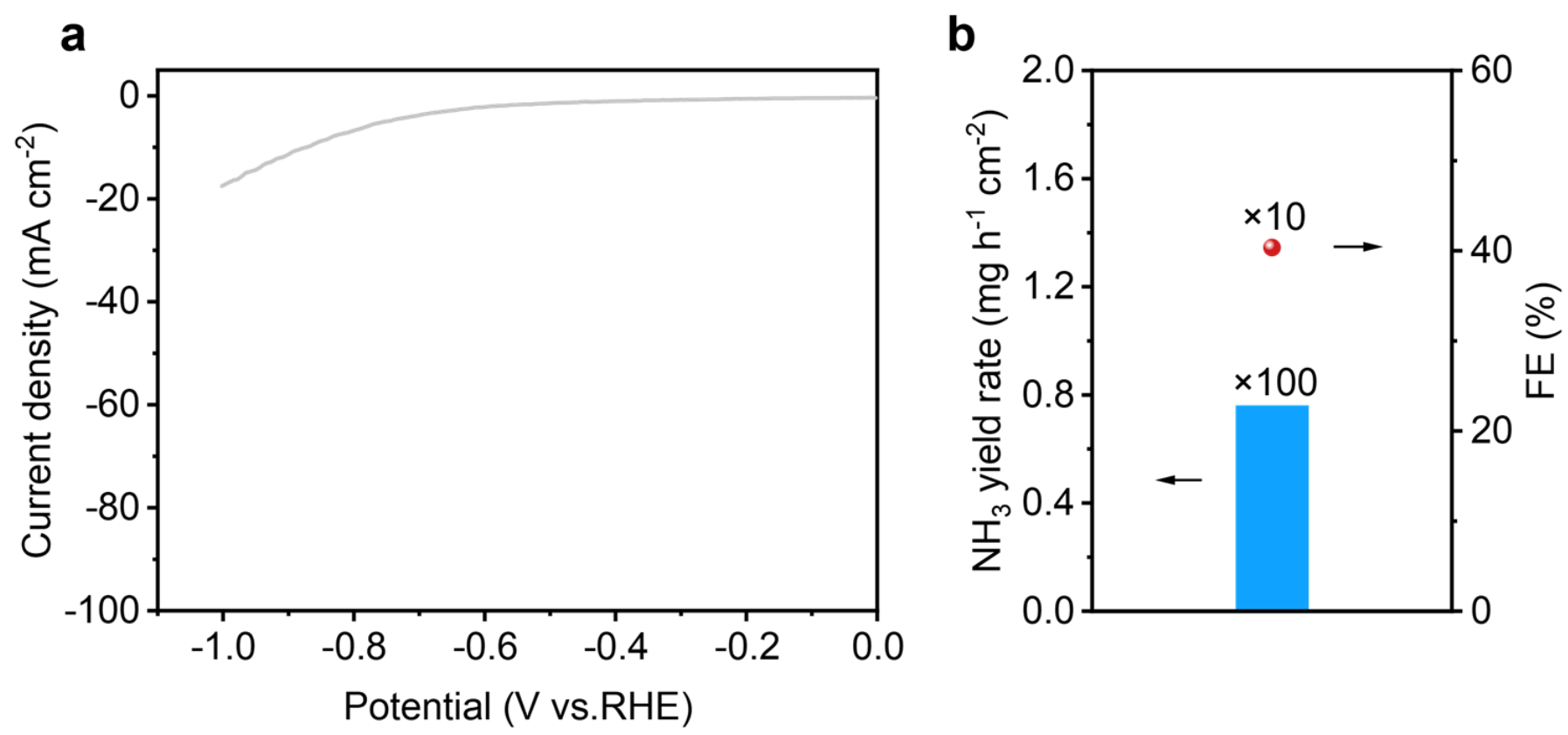

Supplementary Figure 43. NITRR performance of carbon black. (a) LSV polarization curves of carbon black, (b) NH_3 yield rate and FE_{NH3} of carbon black in 0.1 M KOH with 8 mM KNO_3 at -0.4 V vs. RHE.

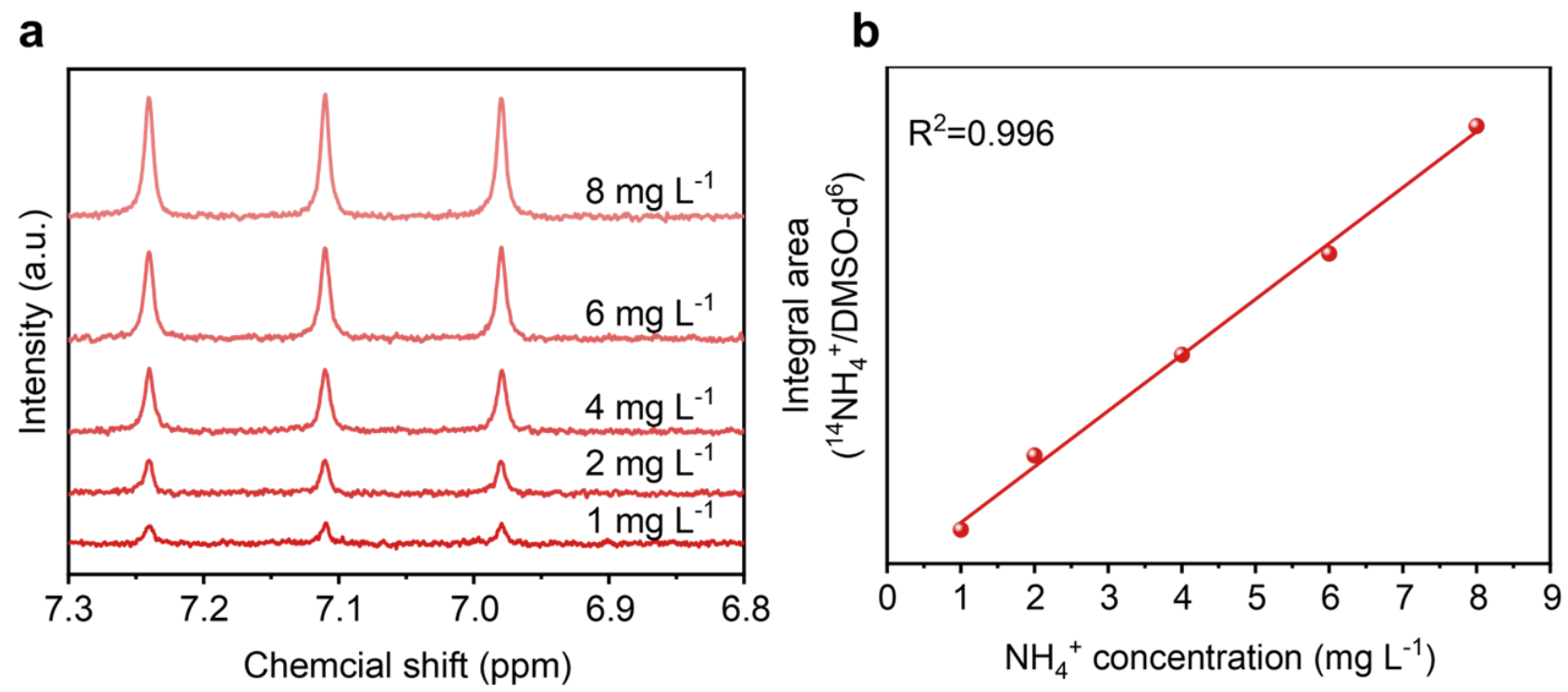

Supplementary Figure 44. Detection and quantification of $^{14}NH_4^+$ by ^{1}H NMR spectra. (a) ^{1}H NMR spectra of $^{14}NH_4^+$ with different concentrations. (b) Standard curve of integral area ($^{14}NH_4^+$/DMSO-d^6) against $^{14}NH_4^+$ concentration.

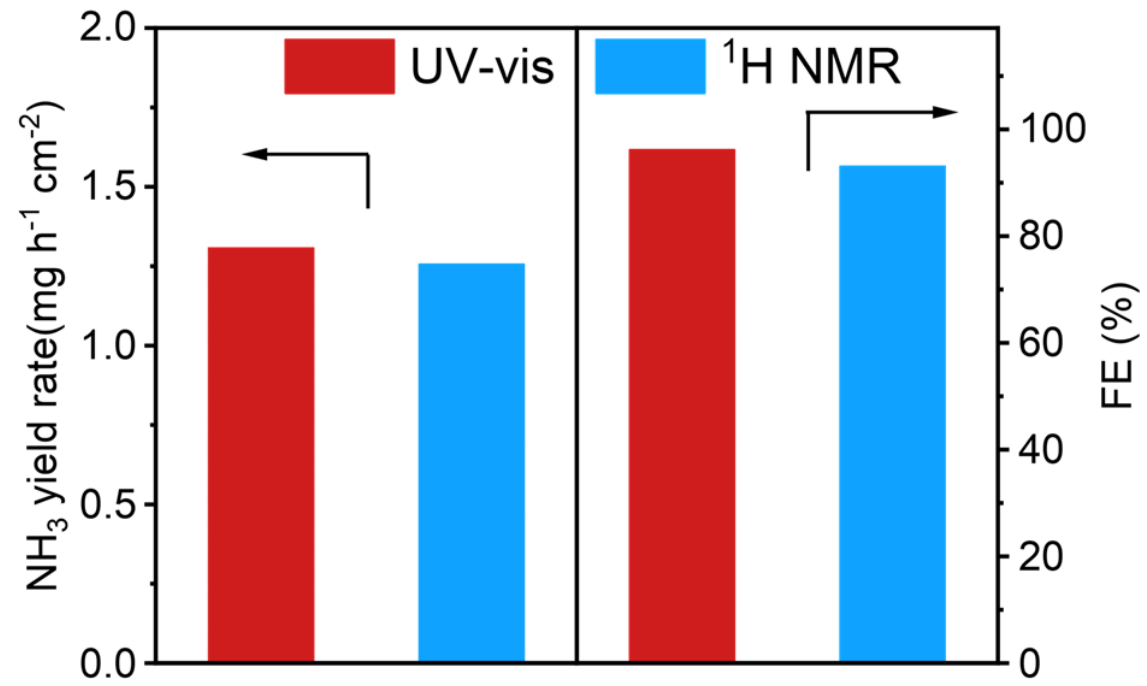

Supplementary Figure 45. NH_3 yield rate and FE_{NH3} determined by UV-vis and ^{1}H NMR methods.

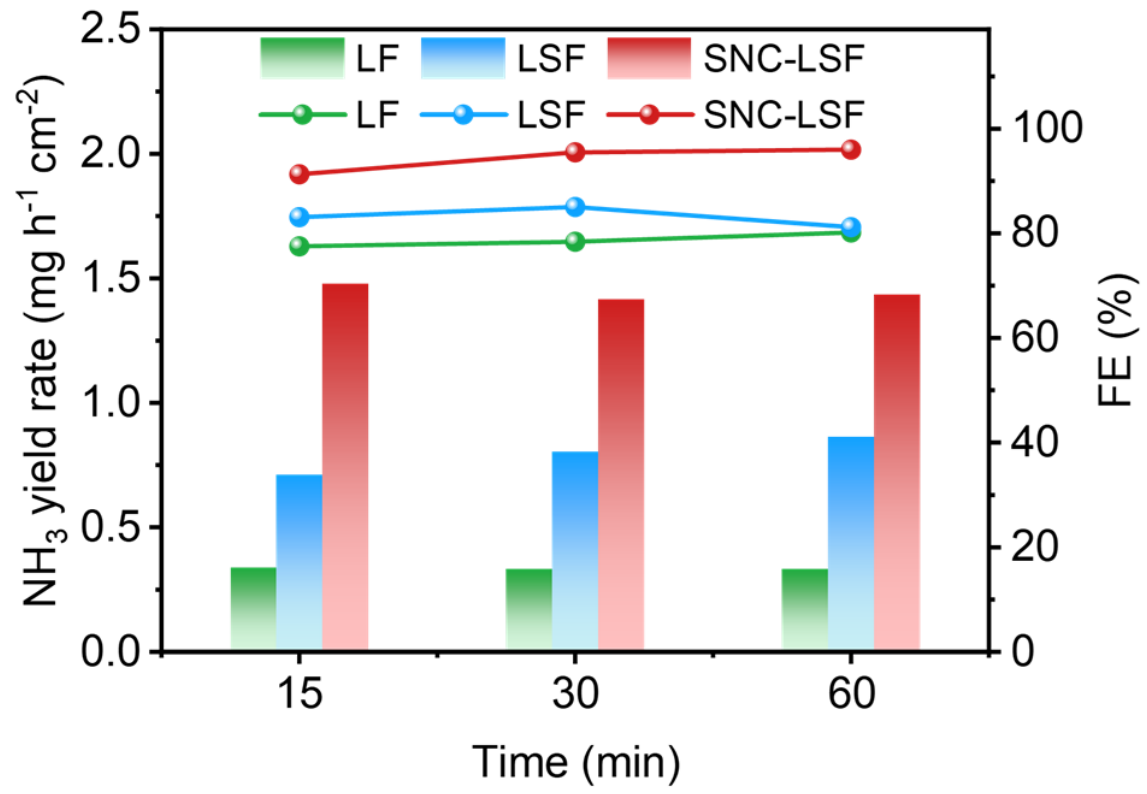

Supplementary Figure 46. NH_3 yield rate and FE_{NH3} of LF, LSF and SNC-LSF in 0.1 M KOH with 8 mM KNO_3 at -0.4 V vs. RHE.

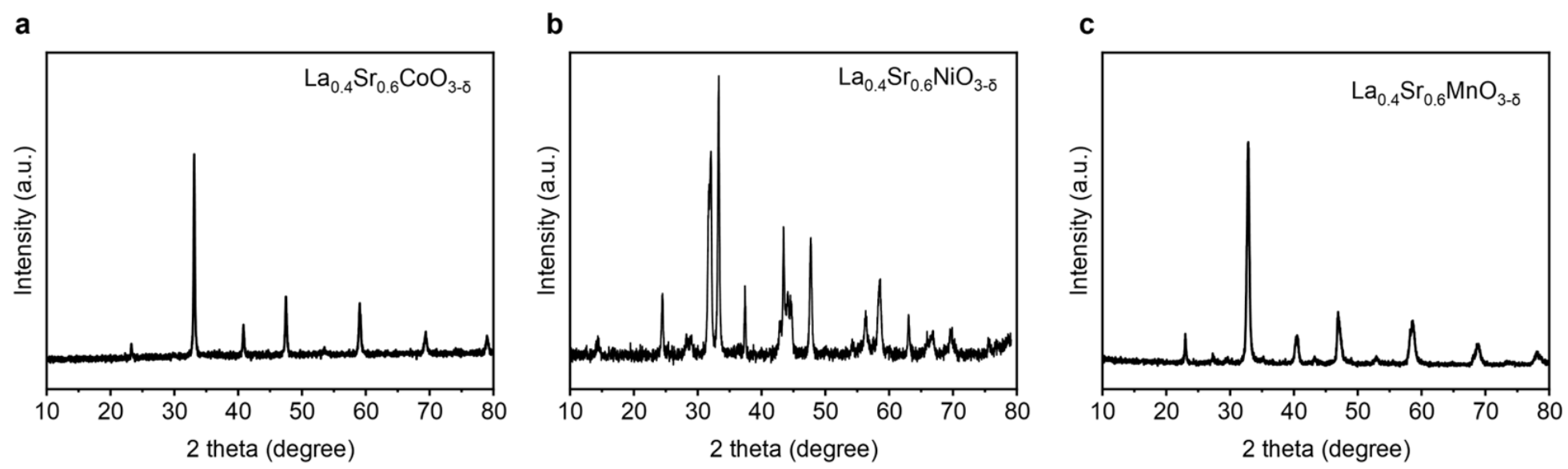

Supplementary Figure 47. XRD patterns of (a) $La_{0.4}Sr_{0.6}CoO_{3-\delta}$, (b) $La_{0.4}Sr_{0.6}NiO_{3-\delta}$, and (c) $La_{0.4}Sr_{0.6}MnO_{3-\delta}$.

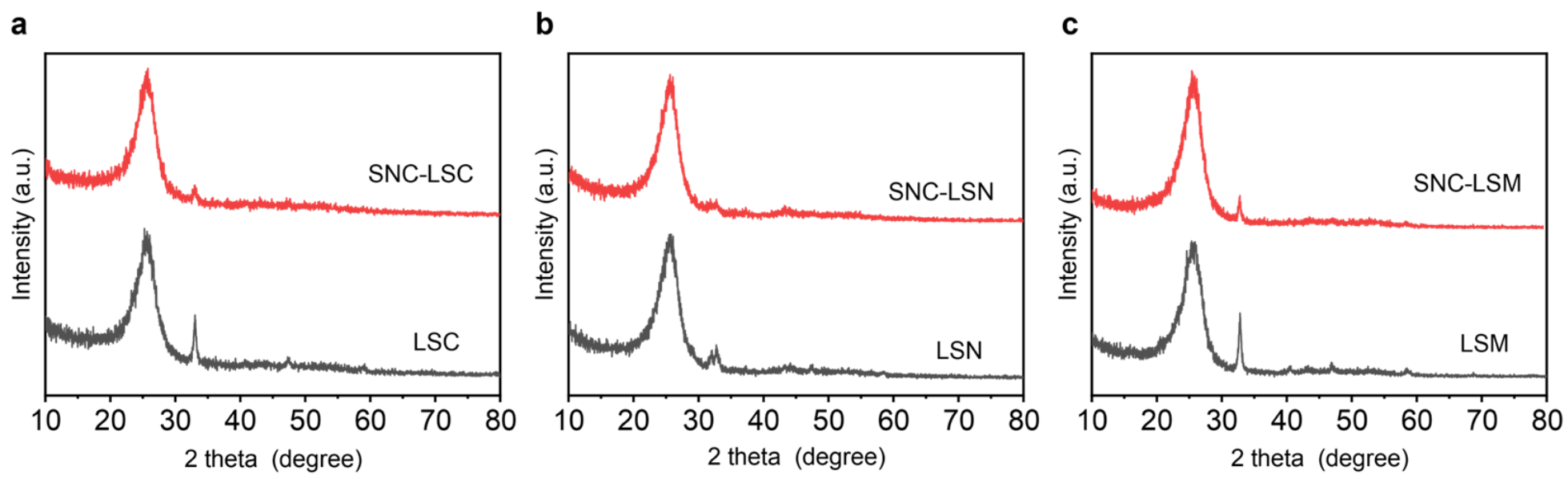

Supplementary Figure 48. XRD patterns of (a) LSC and SNC-LSC, (b) LSN and SNC-LSN, and (c) LSM and SNC-LSM.

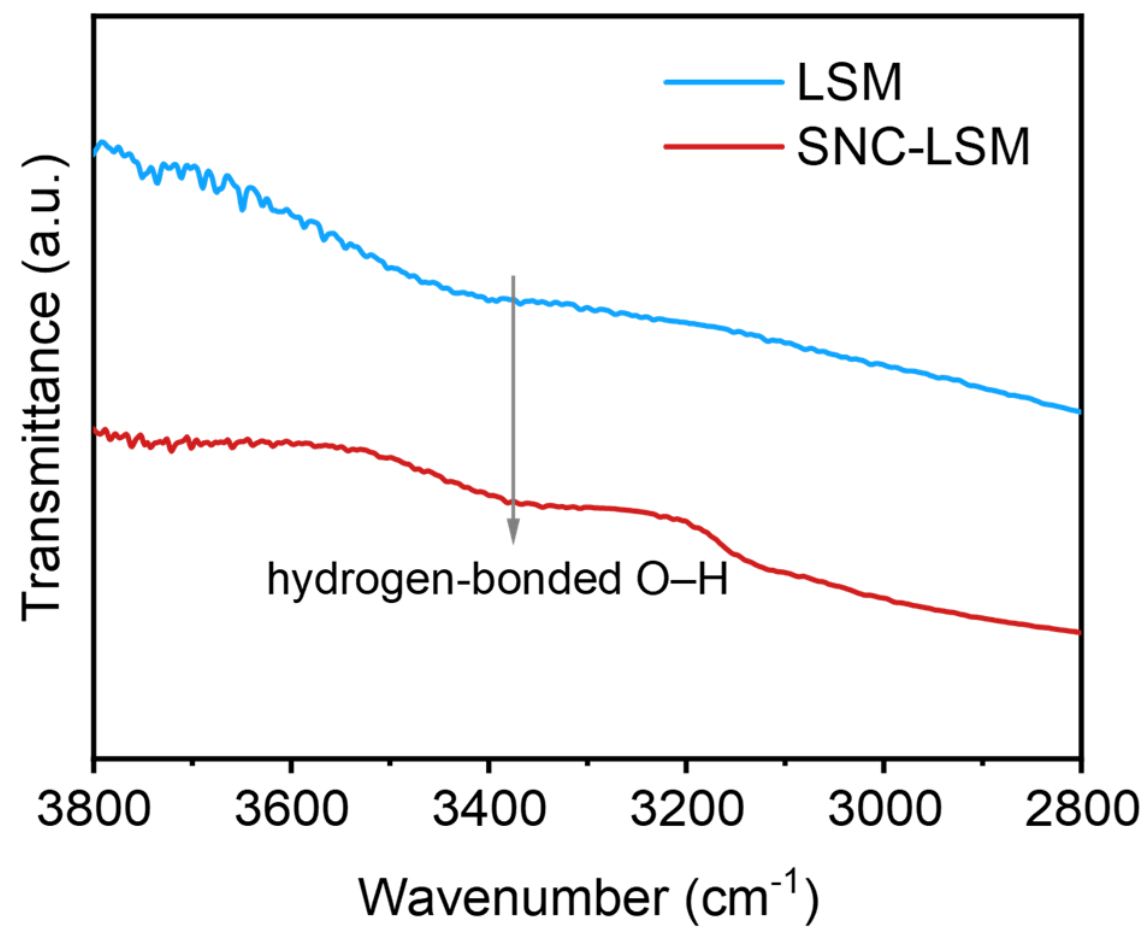

Supplementary Figure 49. FTIR spectra of LSM and SNC-LSM.

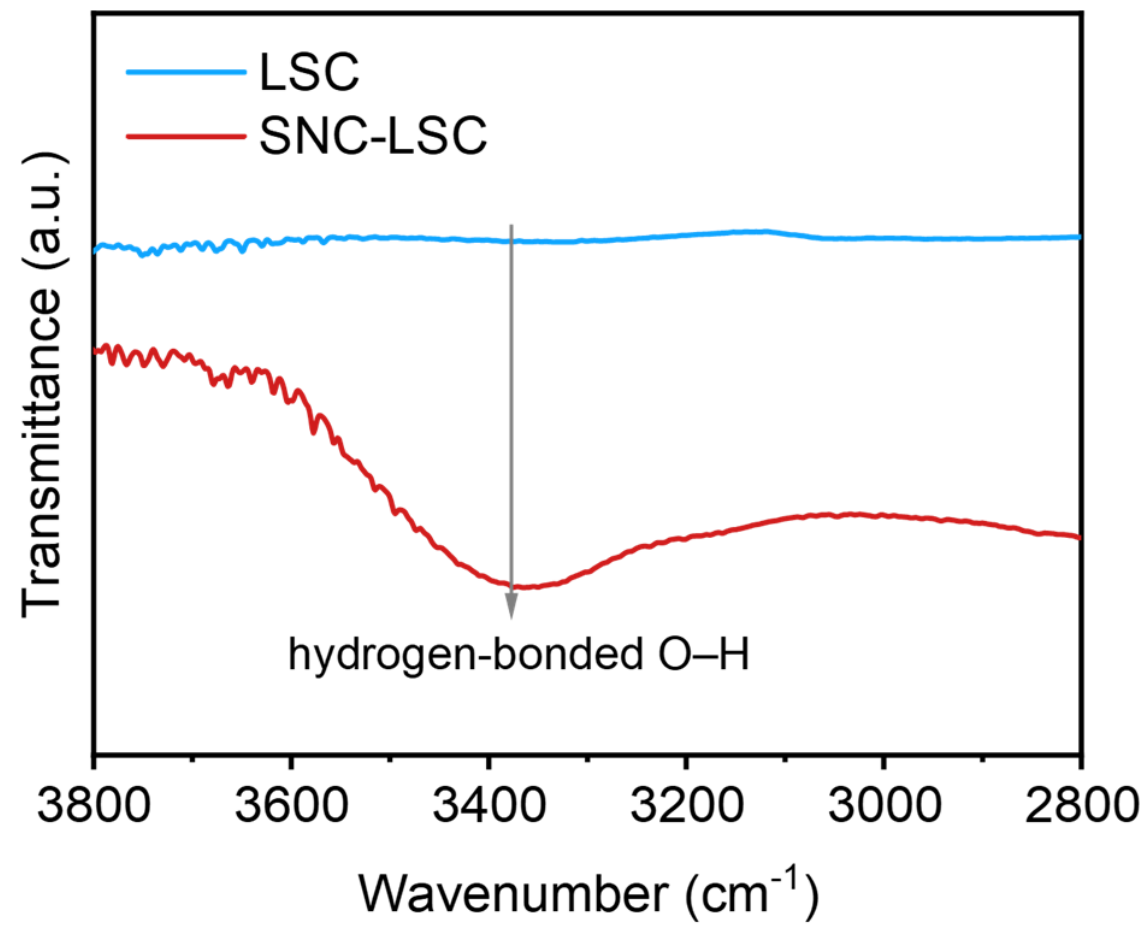

Supplementary Figure 50. FTIR spectra of LSC and SNC-LSC.

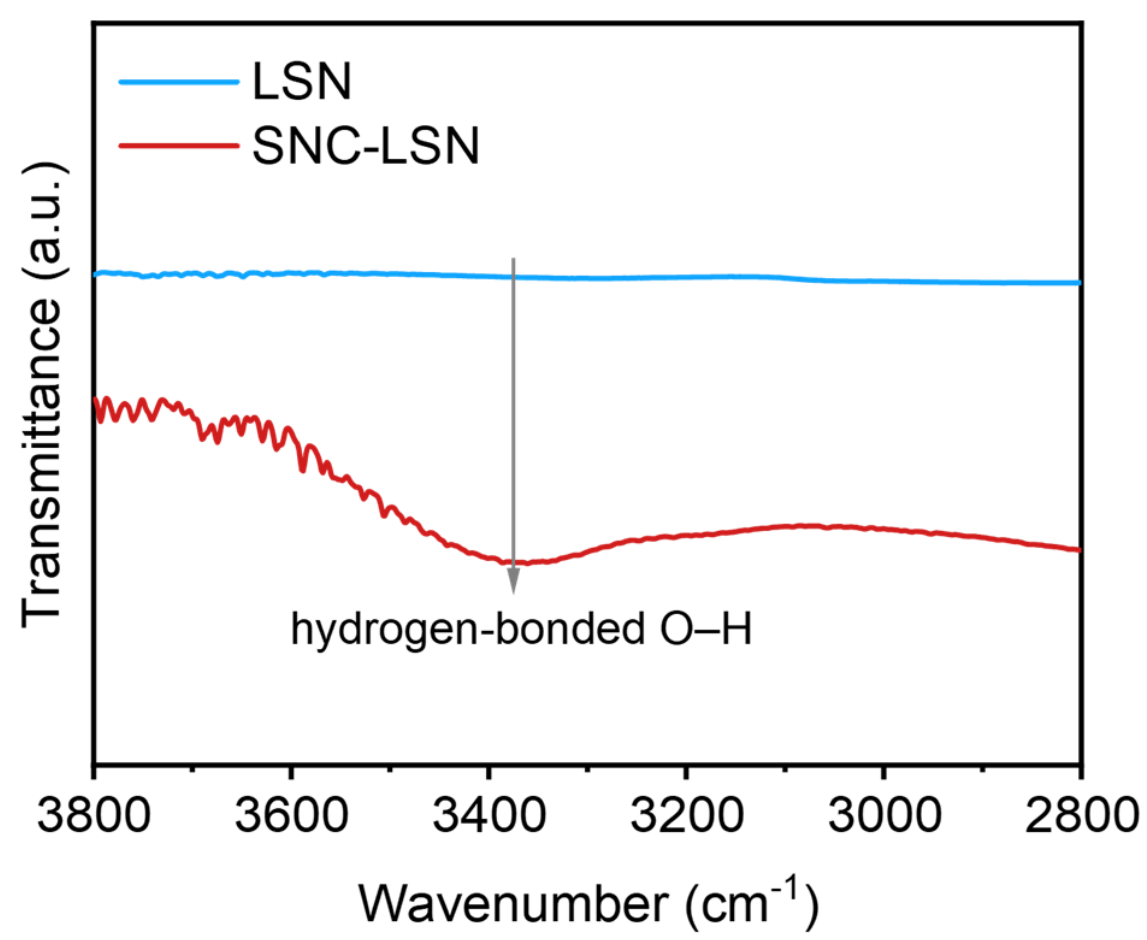

Supplementary Figure 51. FTIR spectra of LSN and SNC-LSN.

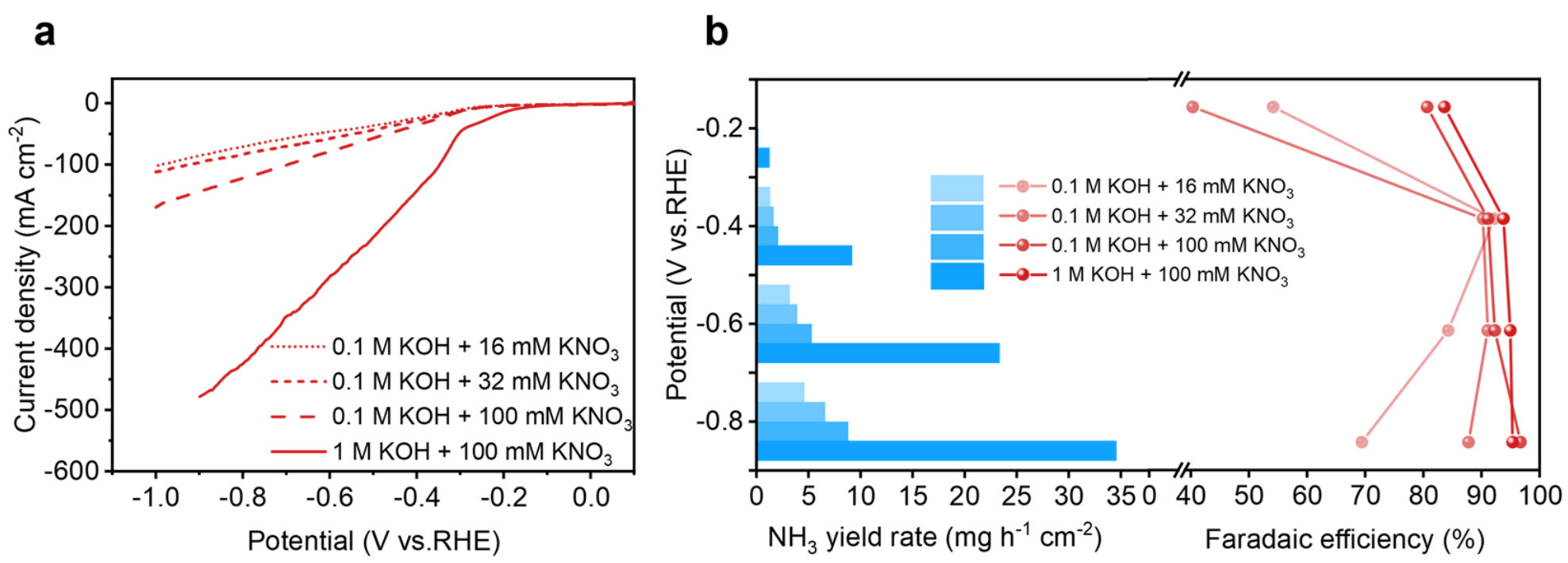

Supplementary Figure 52. (a) LSV curves and (b) NH_3 yield rate and FE_{NH3} of SNC-LSF in different electrolytes.

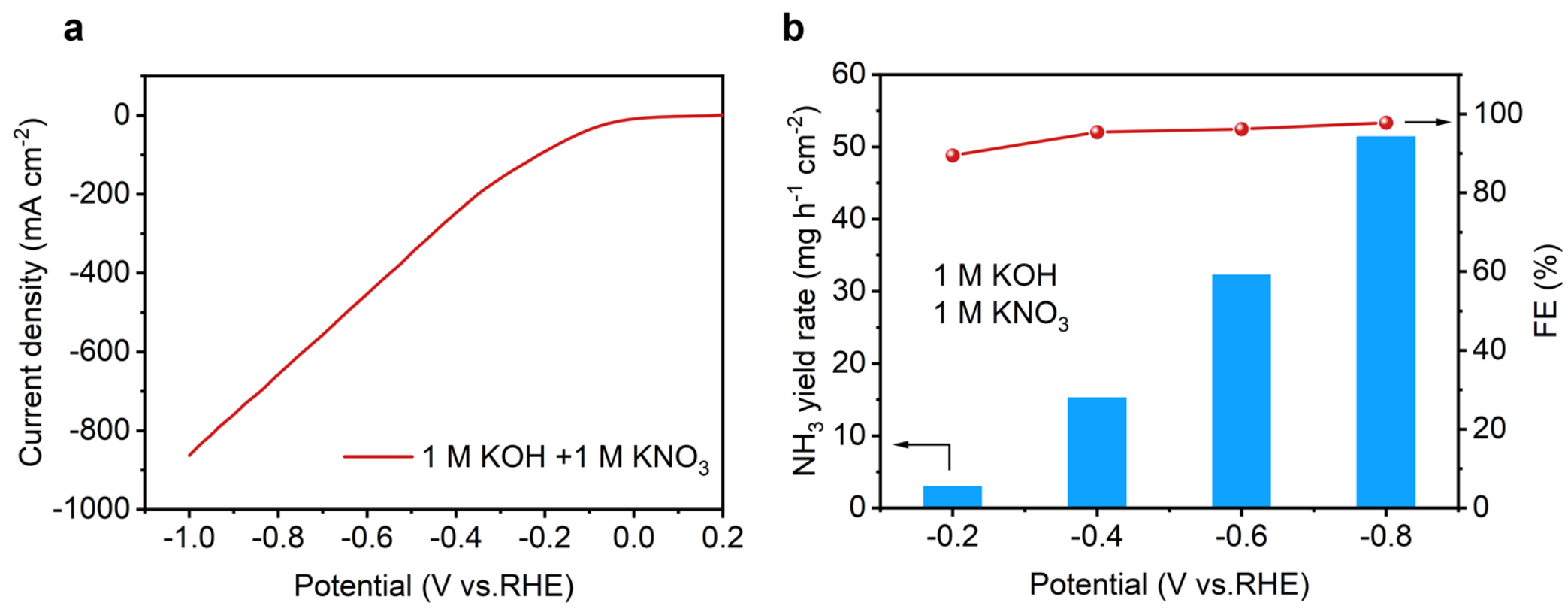

Supplementary Figure 53. (a) LSV curve and (b) NH_3 yield rate and FE_{NH3} of SNC-LSF in 1 M KOH with 1 M KNO_3.

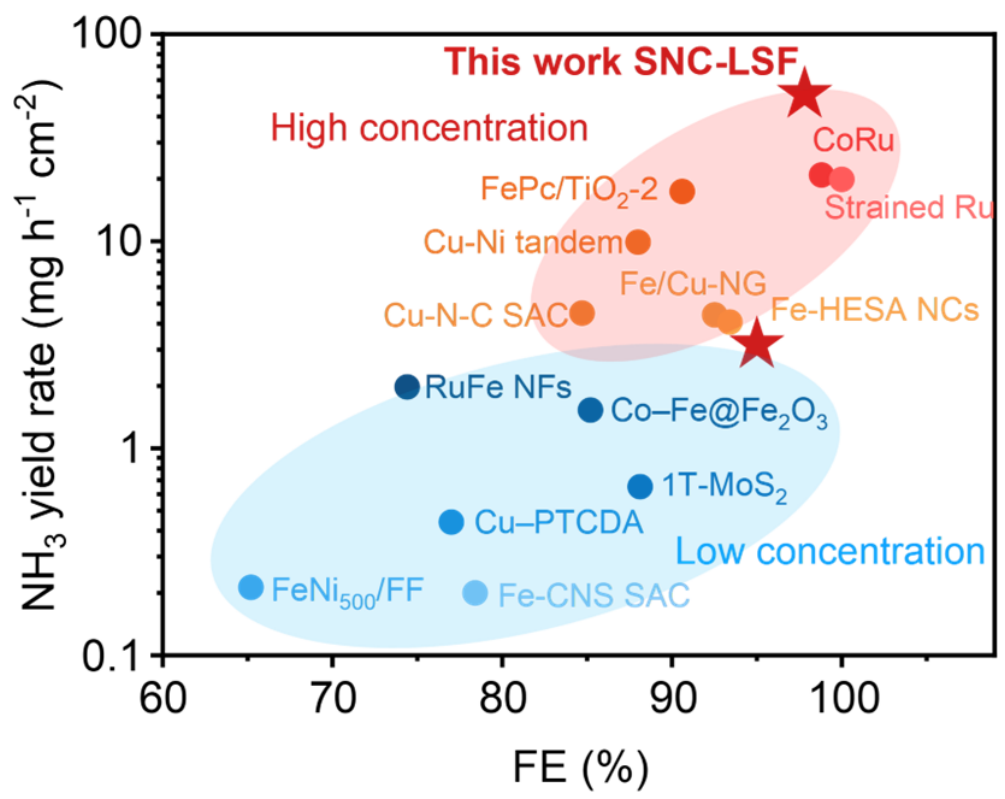

Supplementary Figure 54. Comparison of the electrocatalytic NITRR performance of SNC-LSF at high and low concentrations of nitrate with other extensively reported electrocatalysts.

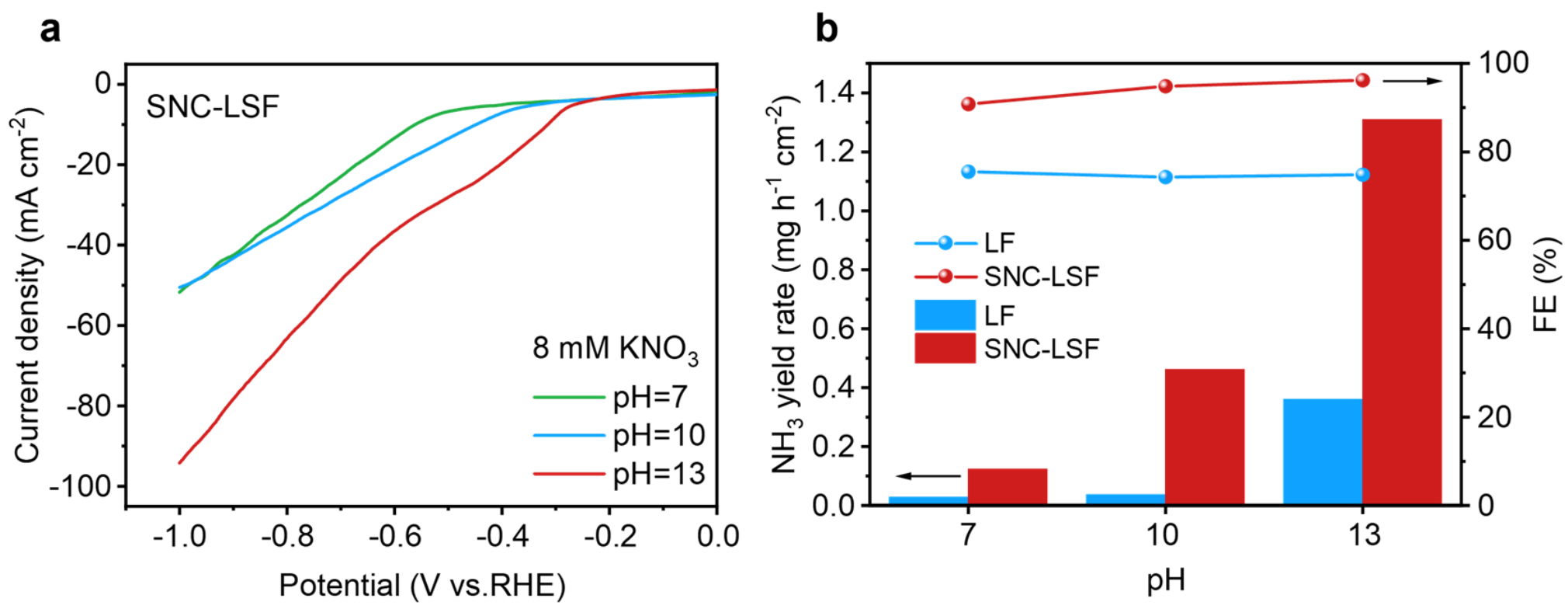

Supplementary Figure 55. (a) LSV curves of SNC-LSF in different electrolyte. (b) NH_3 yield rate and FE_{NH3} of LF and SNC-LSF in different electrolyte at -0.4 V vs. RHE.

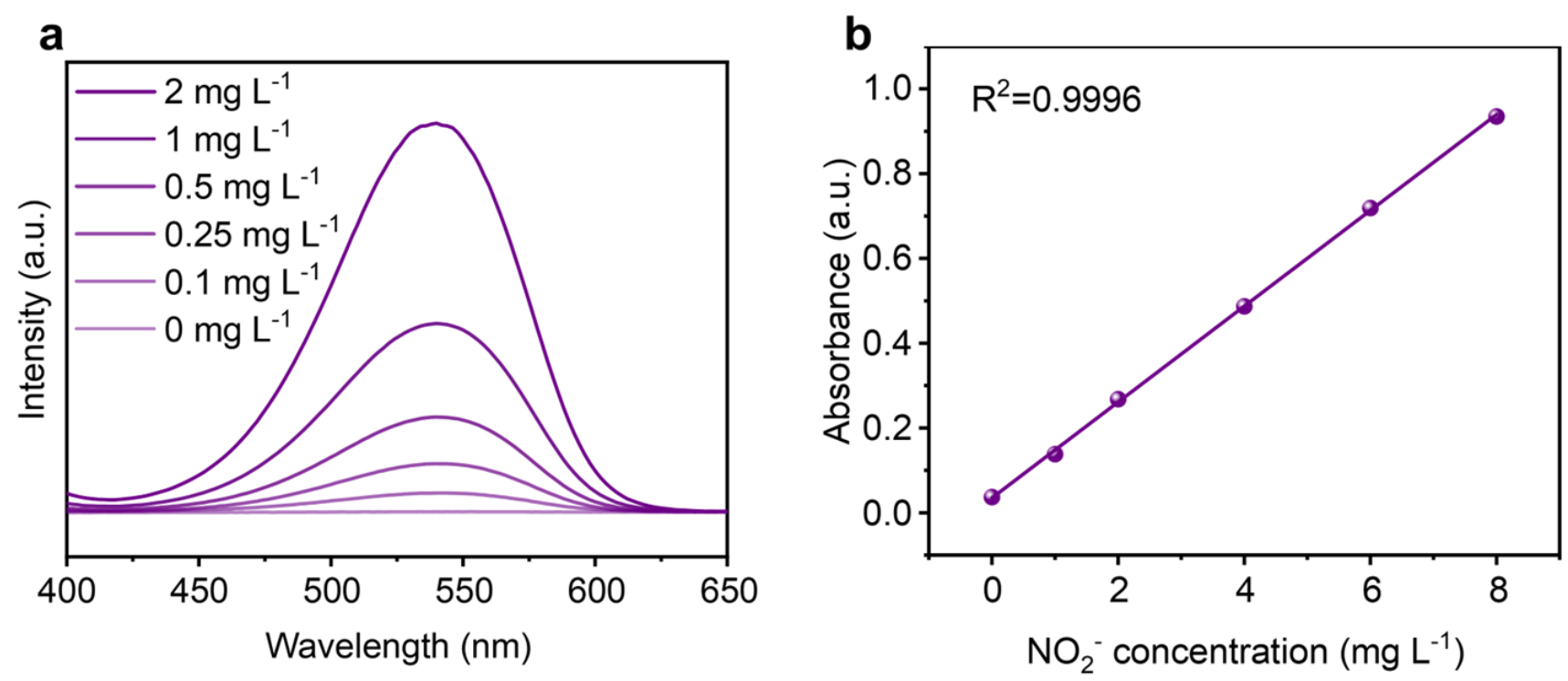

Supplementary Figure 56. (a) UV-vis curves and (b) corresponding calibration curve with known NO_2^- concentrations (the absorbance was measured at a wavelength of 540 nm).

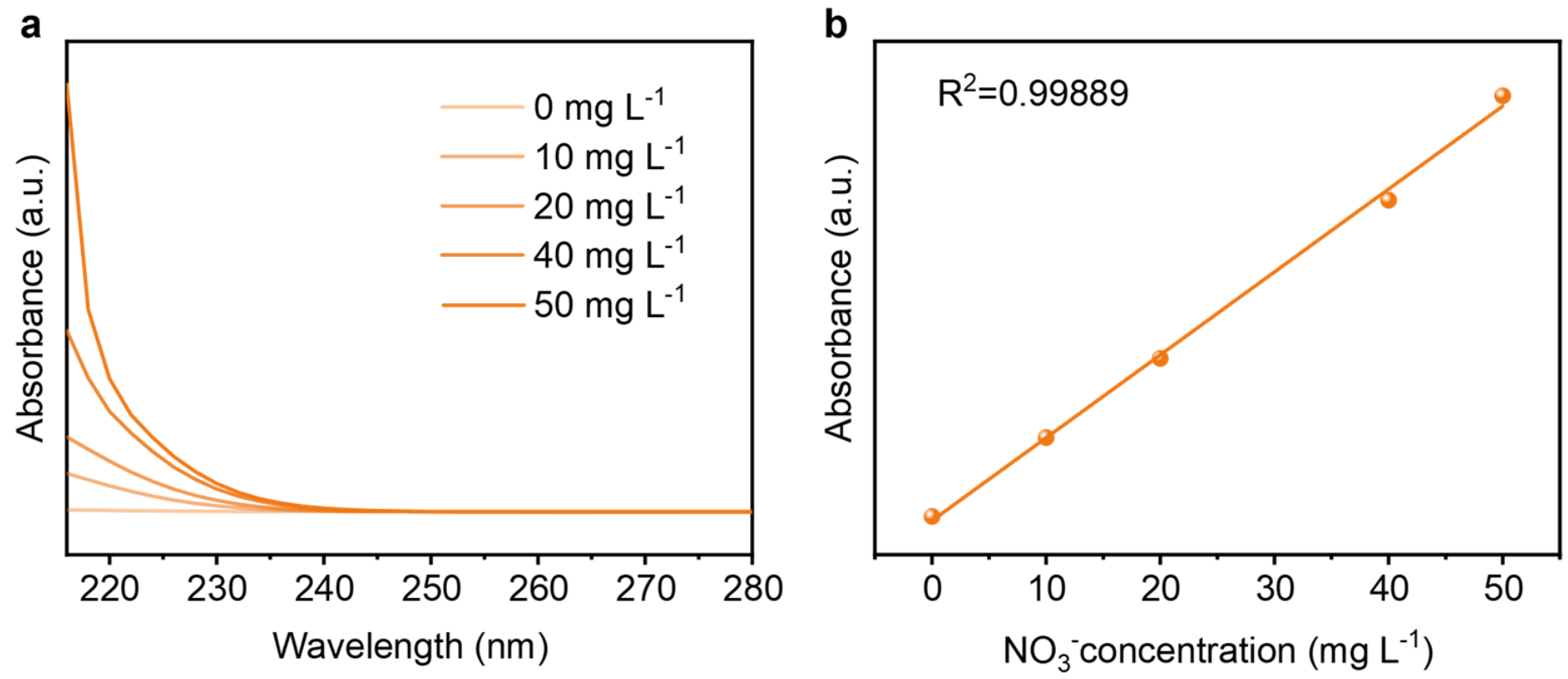

Supplementary Figure 57. (a) UV-vis curves and (b) corresponding calibration curve with known NO_3^- concentrations.

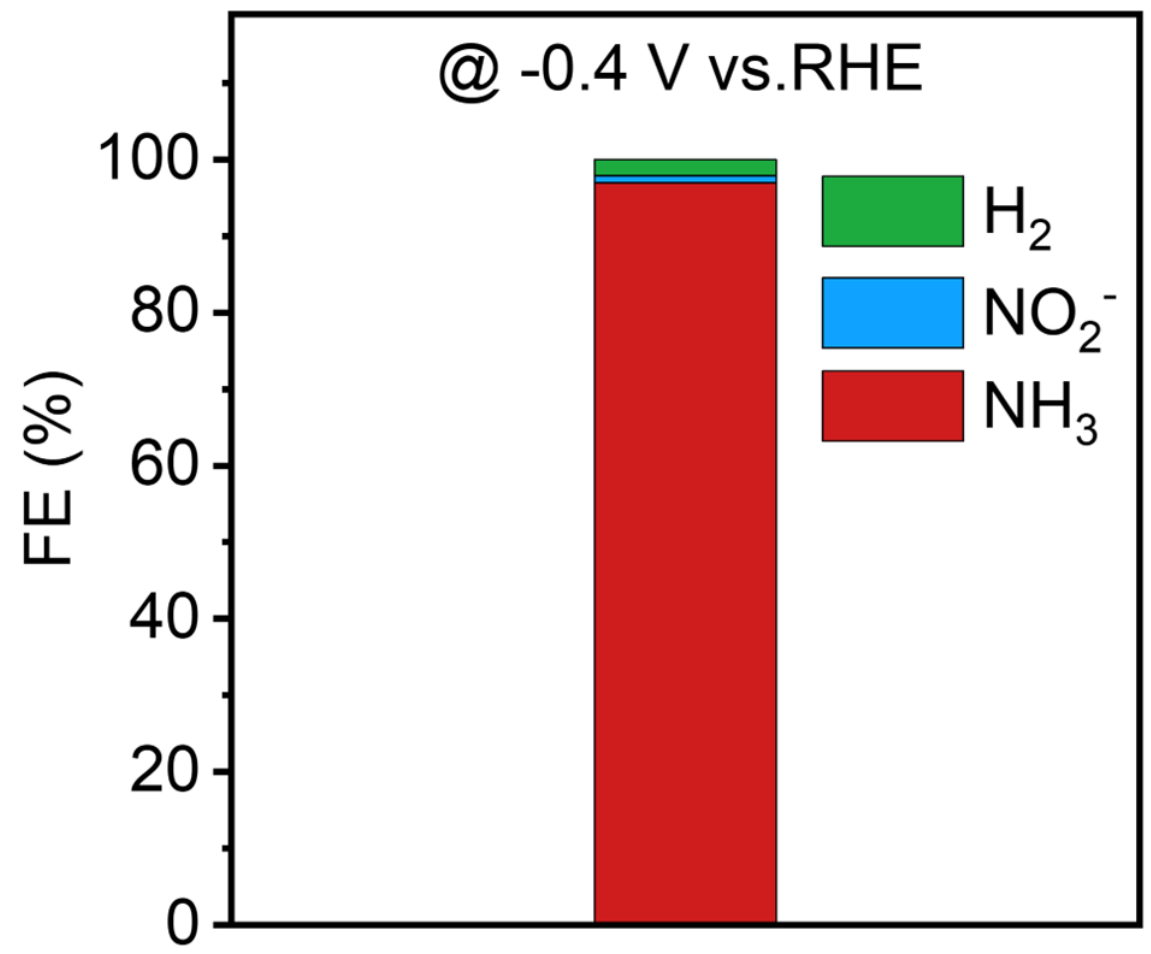

Supplementary Figure 58. Product distribution during NITRR on SNC-LSF. The FE of H_2, NO_2^- and NH_3 during NITRR on SNC-LSF at -0.4 V vs. RHE.

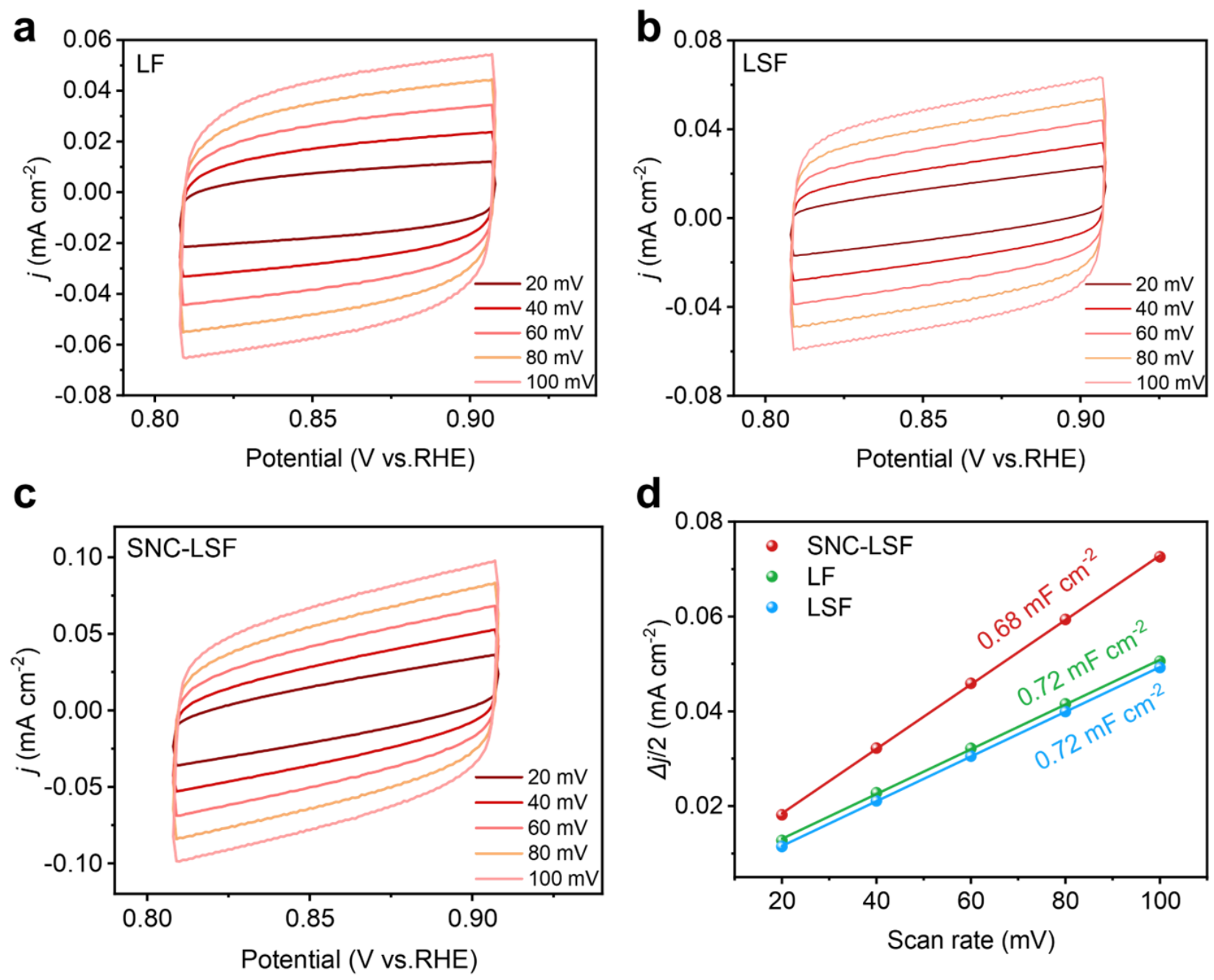

Supplementary Figure 59. Cyclic voltammetry curves of (a) LF, (b) LSF and (c) SNC-LSF at the scan rates of 20 to 100 mV s^{-1}. (d) Plots of half of the current density difference ($\Delta j/2$) at the centered potential against the scan rate. The slope of fitted straight line is the C_{dl} value.

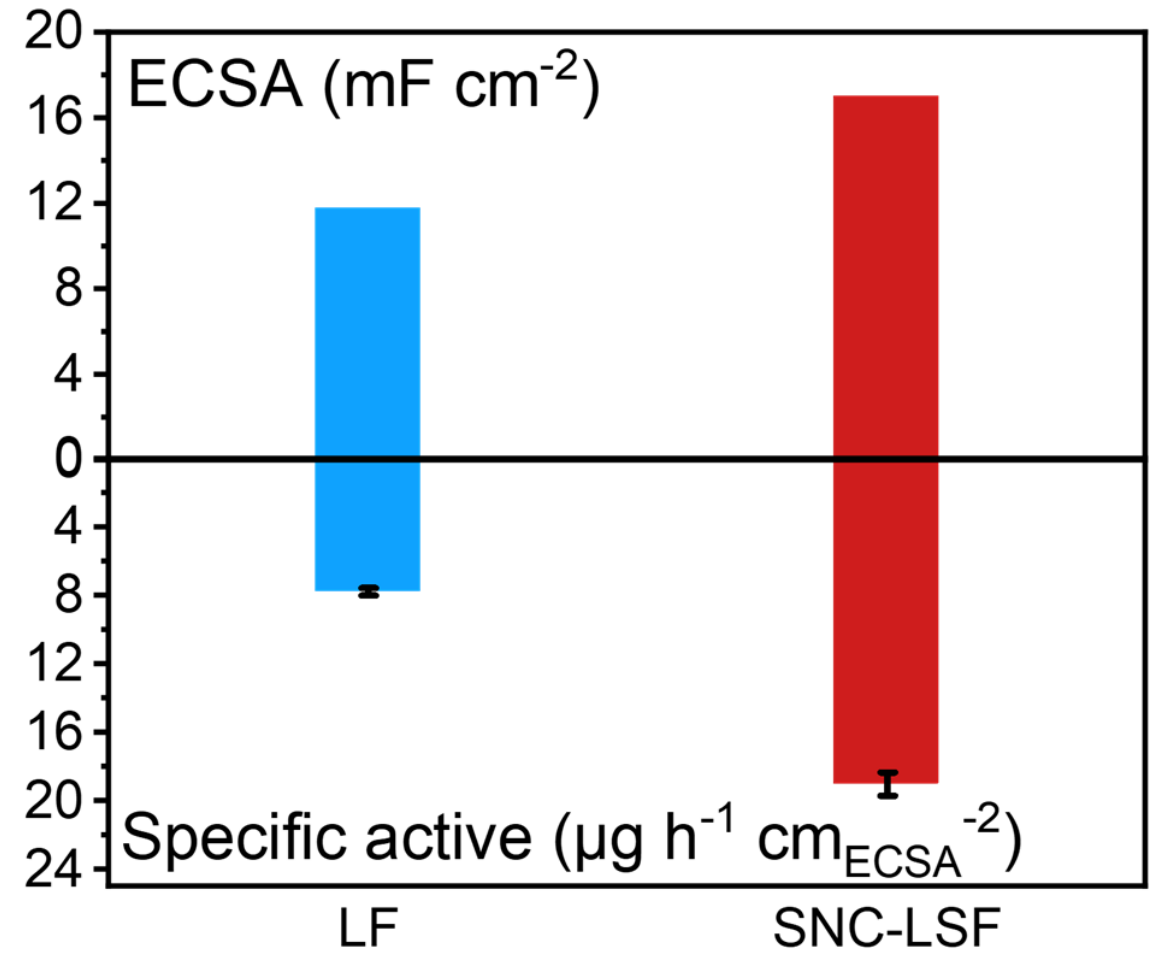

Supplementary Figure 60. Specific activity normalized to ECSA of LF and SNC-LSF in 0.1 M KOH with 8 mM KNO_3 at -0.4 V vs. RHE.

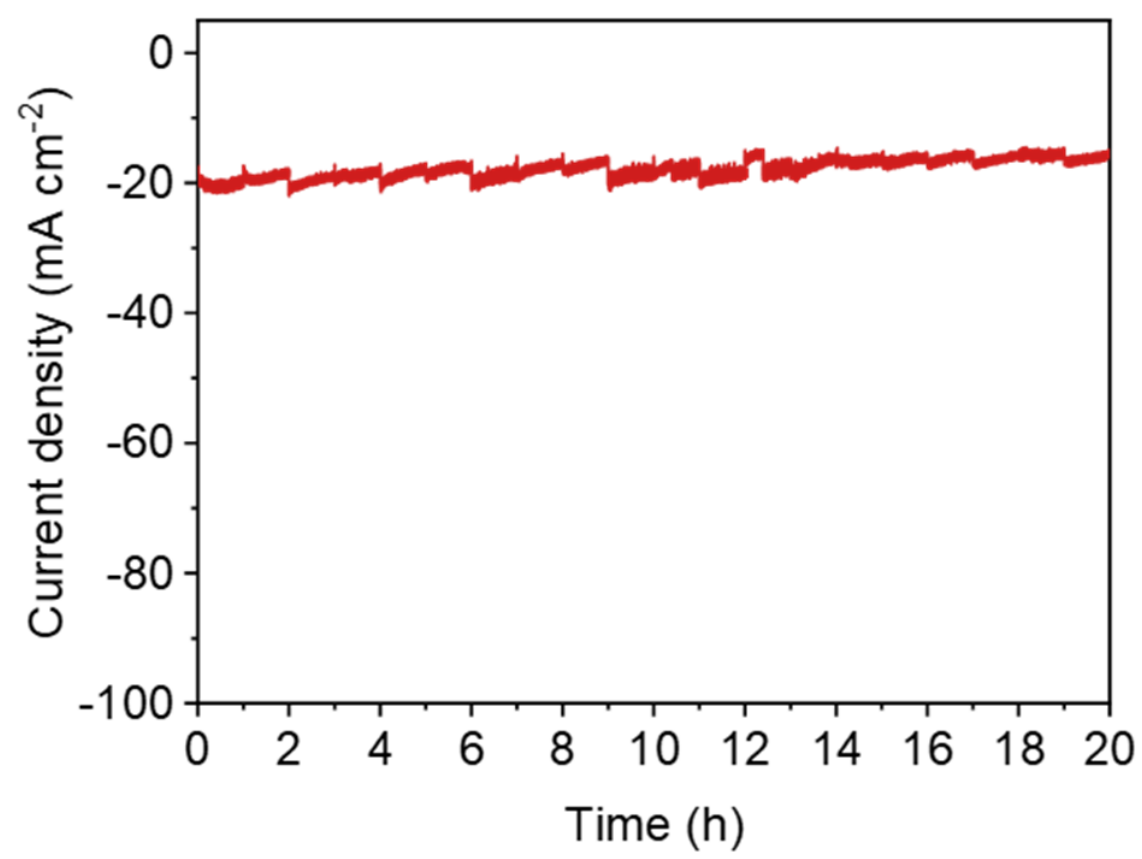

Supplementary Figure 61. Time-dependent current density curves of the SNC-LSF at -0.4 V vs. RHE.

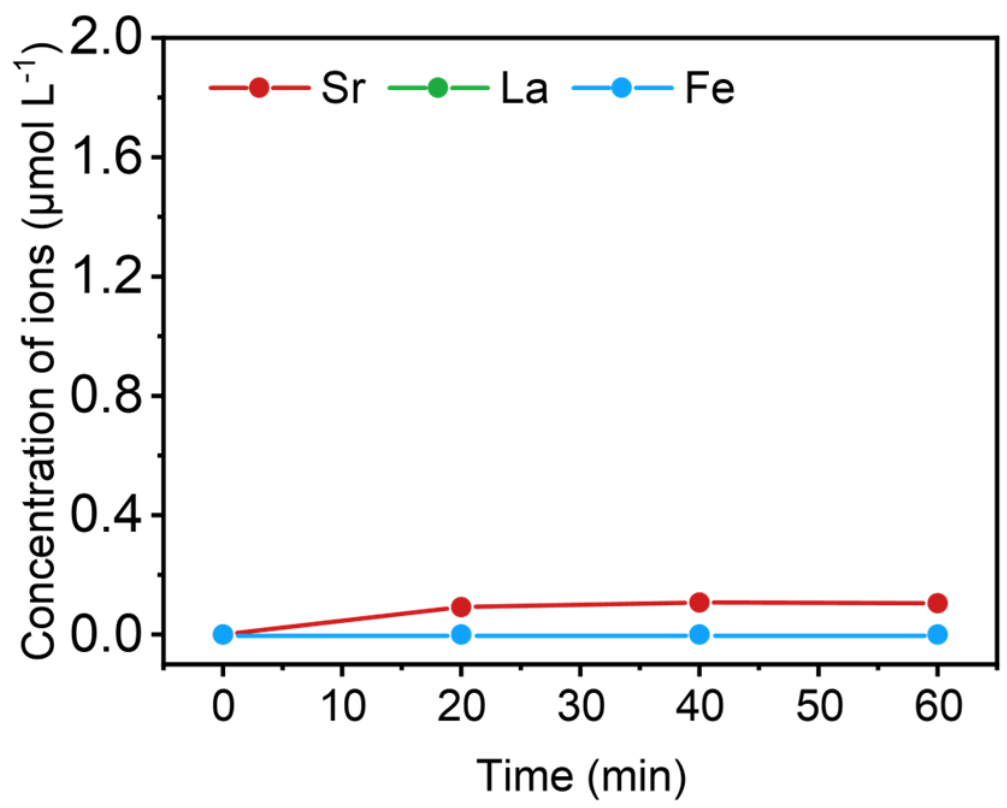

Supplementary Figure 62. Dissolution concentrations of La, Sr and Fe elements from SNC-LSF in 0.1 M KOH with 8 mM KNO_3 at -0.4 V vs. RHE.

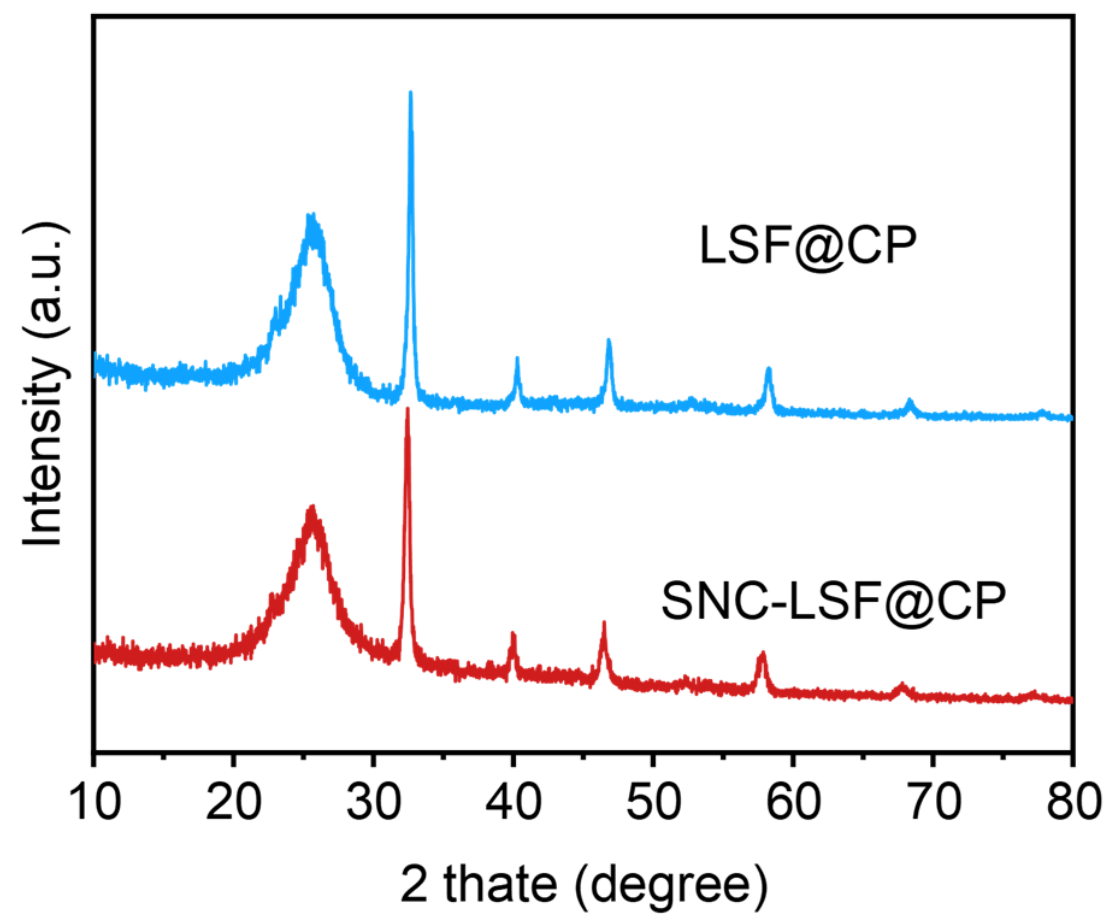

Supplementary Figure 63. XRD pattern of SNC-LSF after stability test and exposure to air 24 h.

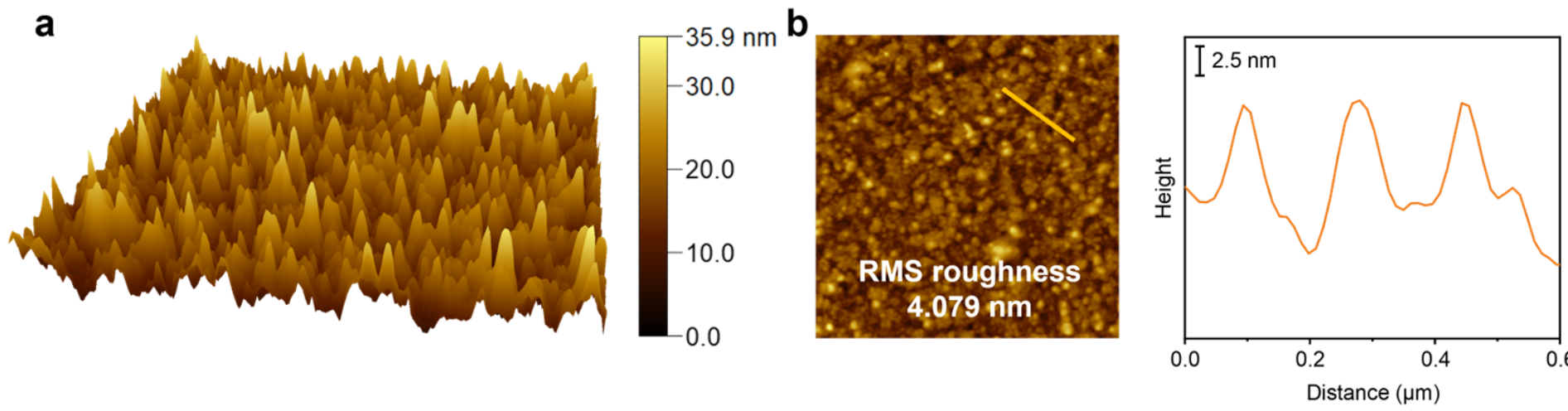

Supplementary Figure 64. (a) AFM maps and (b) Root mean square (RMS) roughness and line scans of SNC-LSF after stability test.

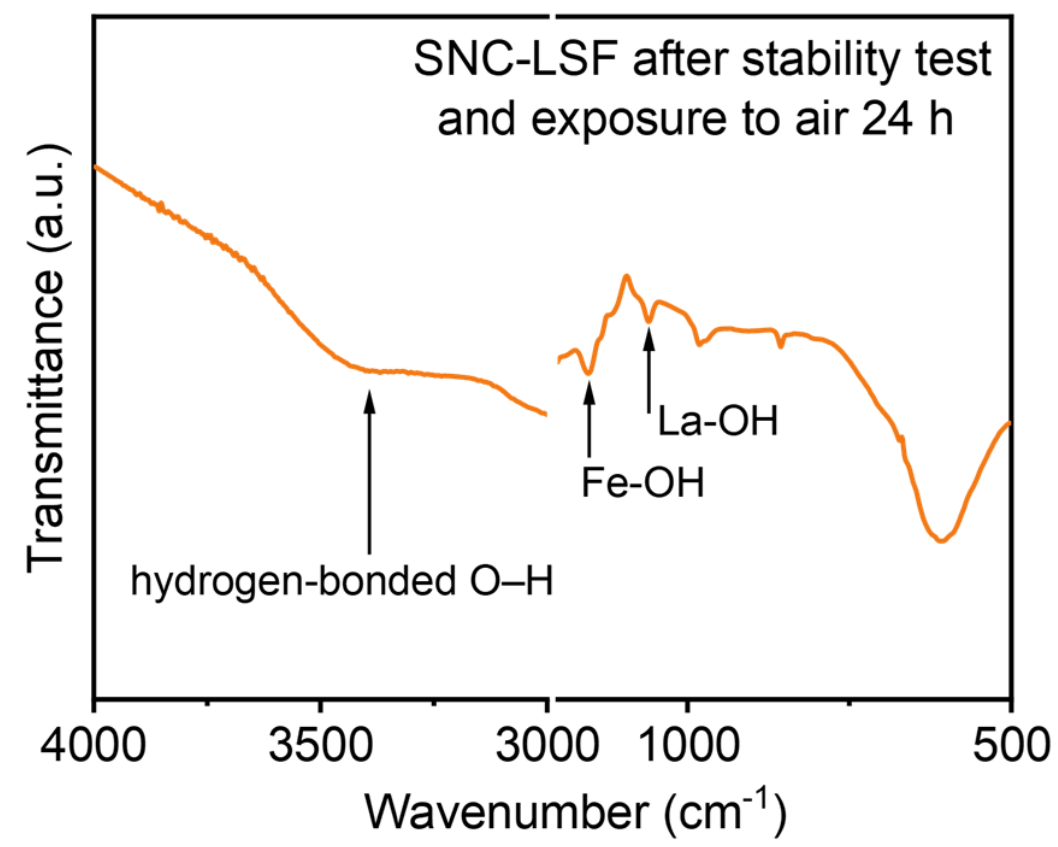

Supplementary Figure 65. FTIR spectra of SNC-LSF after stability test and exposure to air 24 h.

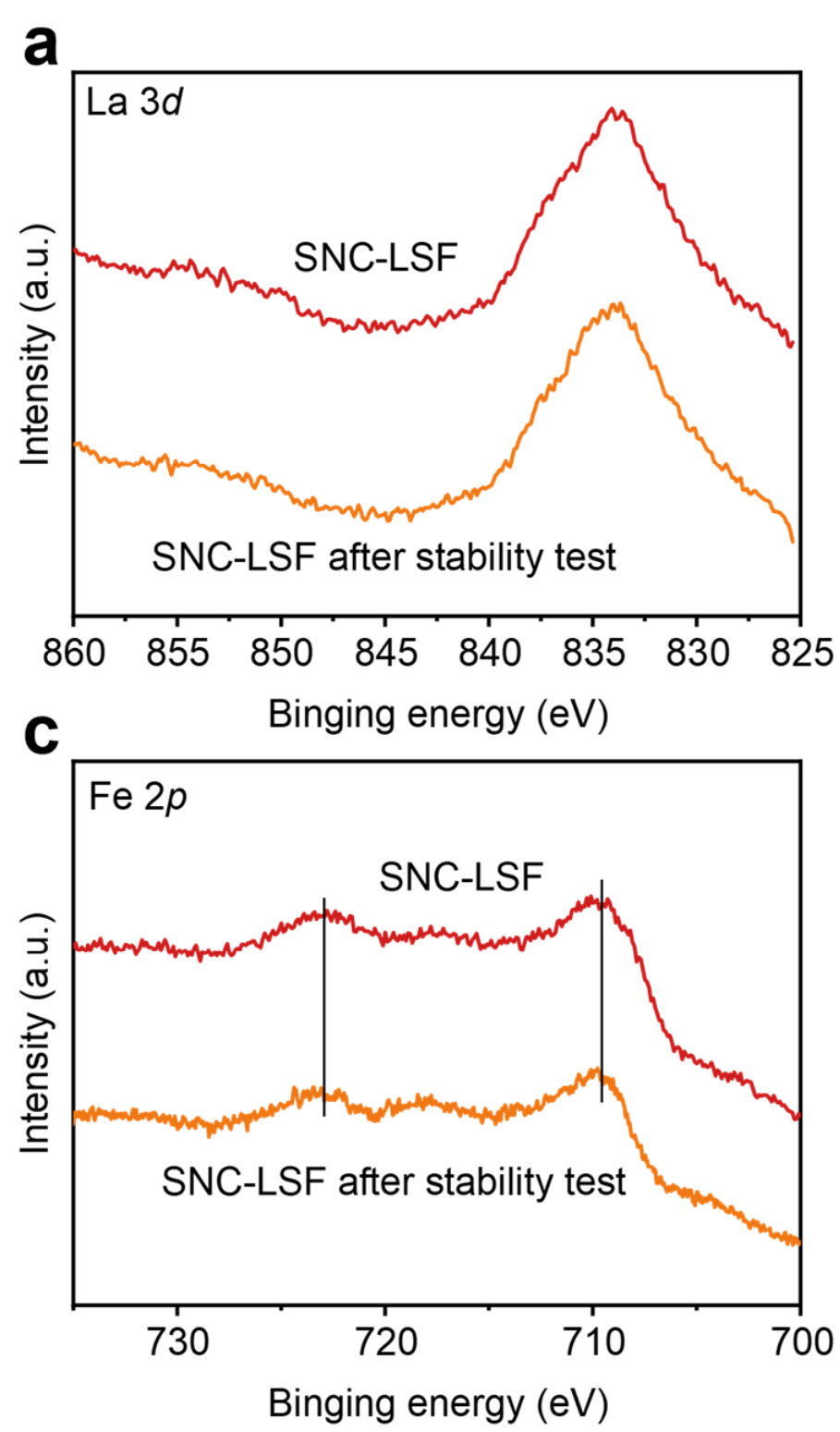

Supplementary Figure 66. XPS spectra of SNC-LSF and SNC-LSF after stability test.

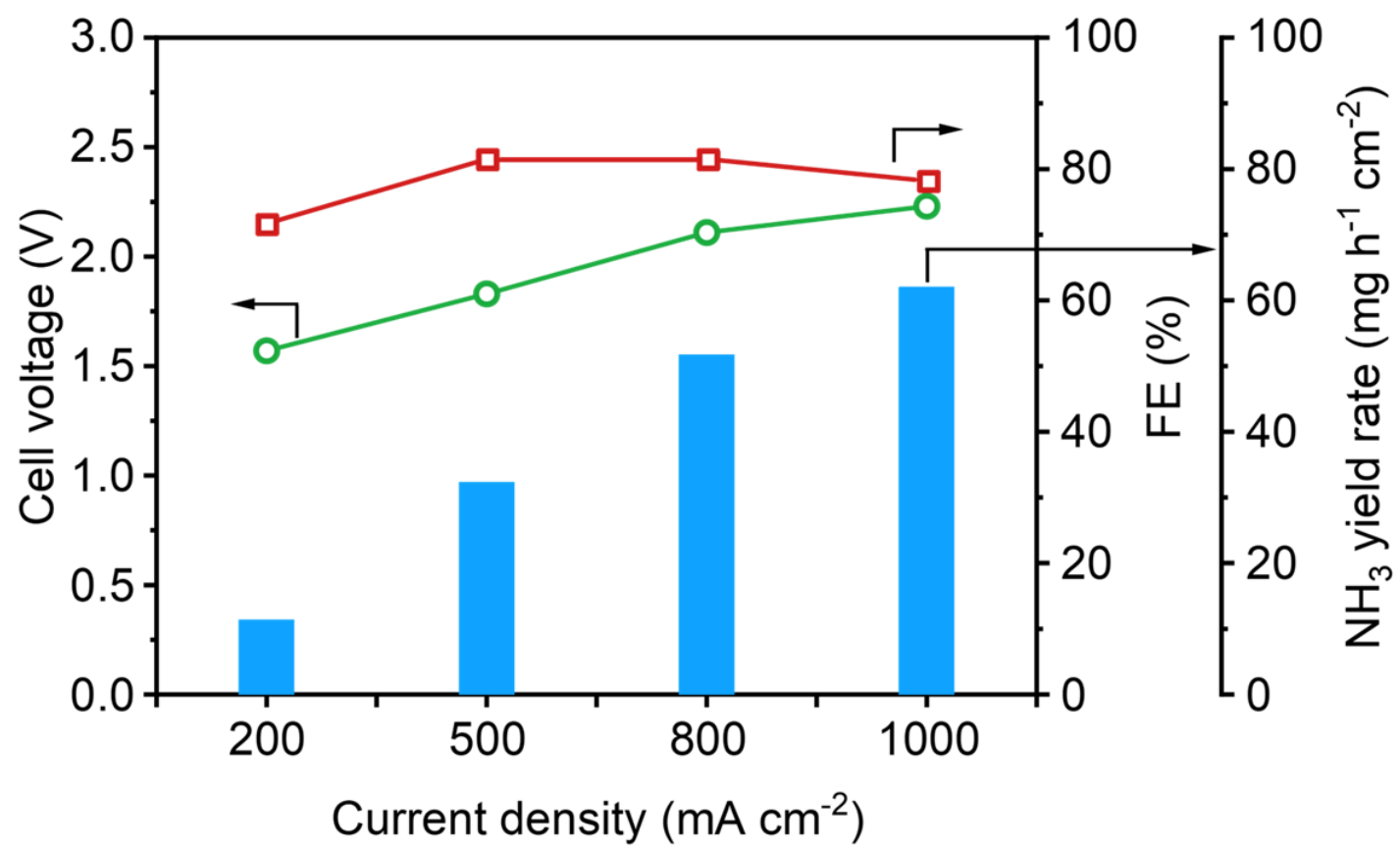

Supplementary Figure 67. NH_3 yield rates, FE_{NH3} and cell voltage of the SNC-LSF/CP over a broad range of current densities in the MEA.

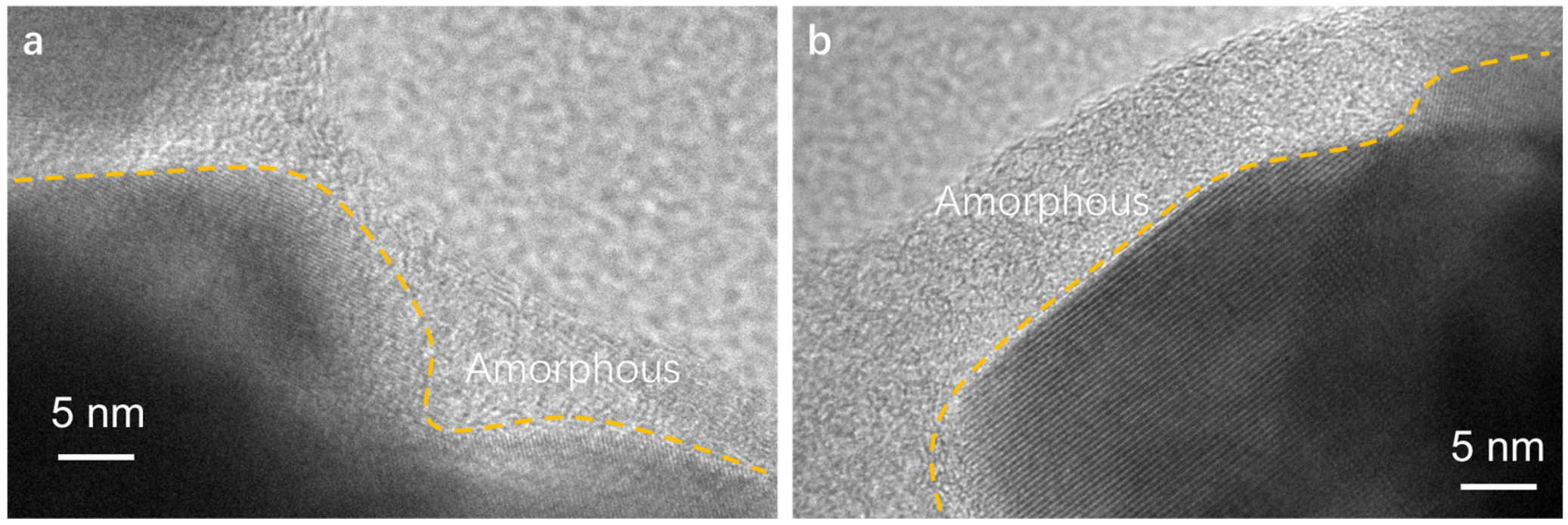

Supplementary Figure 68. Two sets of HRTEM images of SNC-LSF after the stability test in the MEA system.

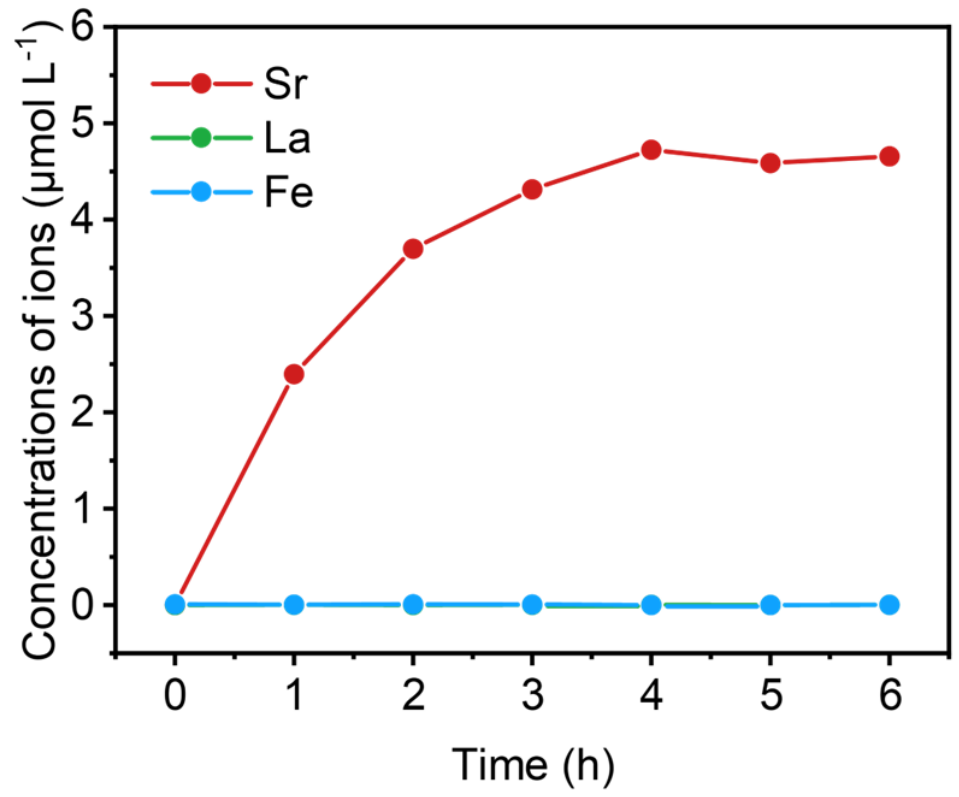

Supplementary Figure 69. Dissolution concentrations of La, Sr and Fe elements from LSF in the MEA at 1 A cm^{-2}.

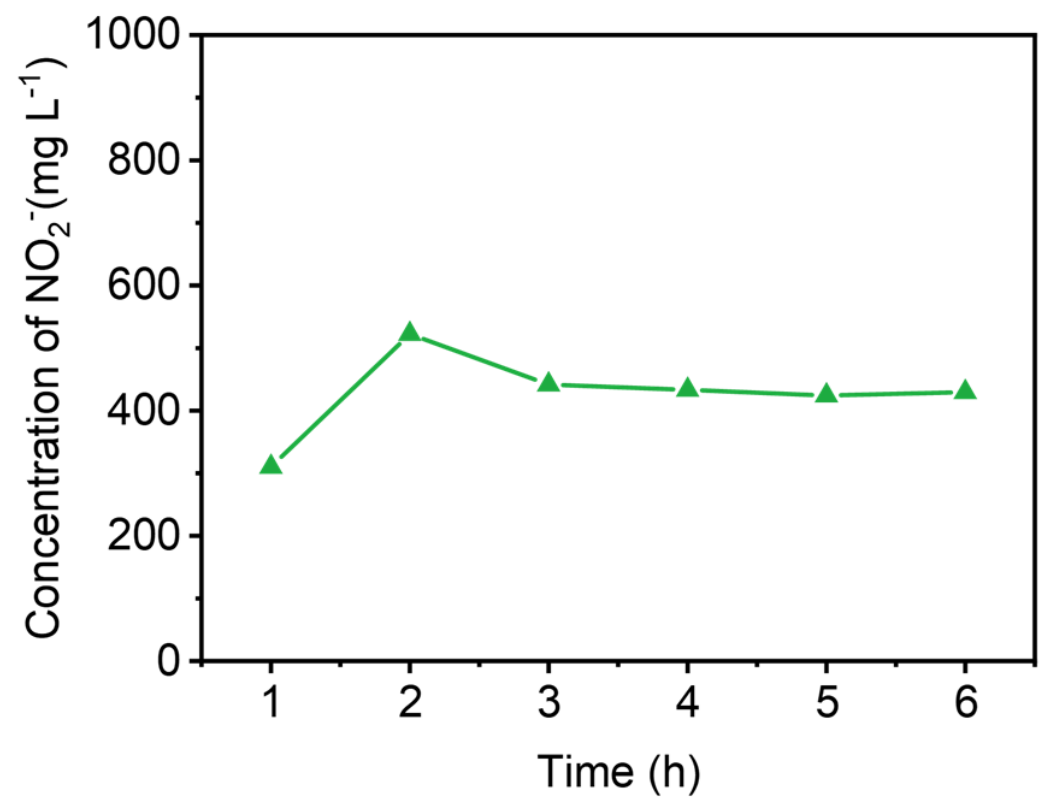

Supplementary Figure 70. Time-dependent concentration of NO_2^- in the MEA at 1 A cm^{-2}.

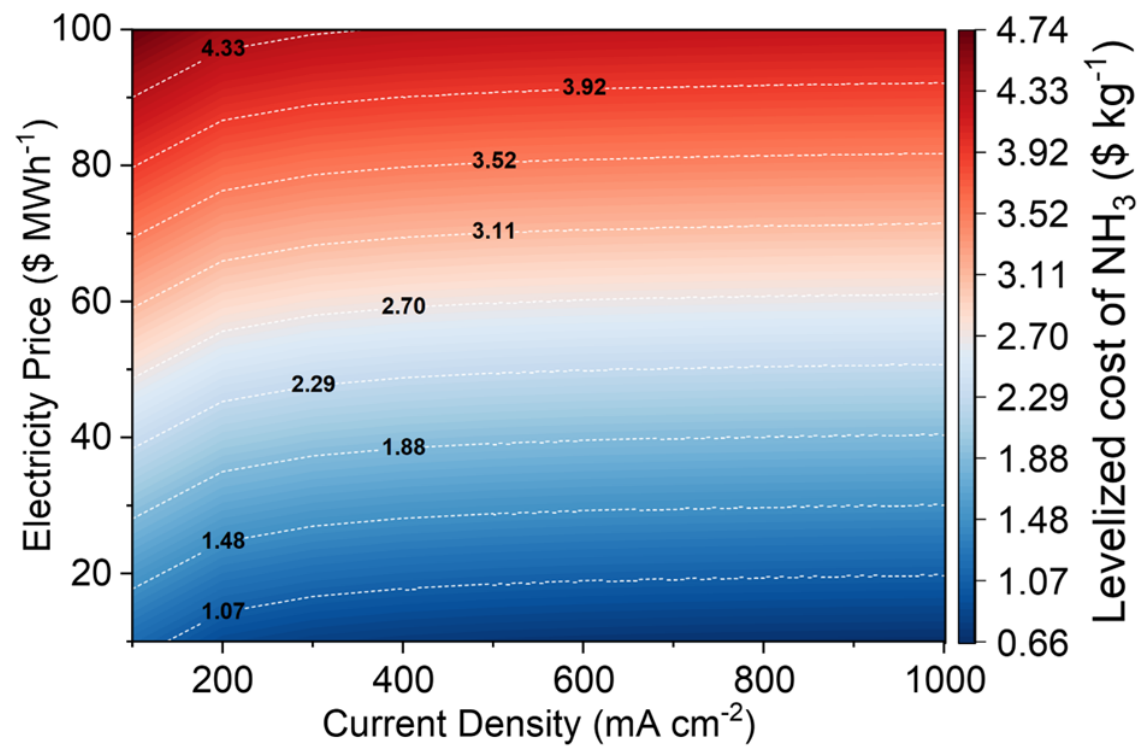

Supplementary Figure 71. Techno-economic landscape of NH_3 production cost as a function of electricity price and current density. Contour plot illustrating the dependence of levelized NH_3 production cost on electricity price and current density, highlighting optimal operational conditions under varying energy input scenarios.

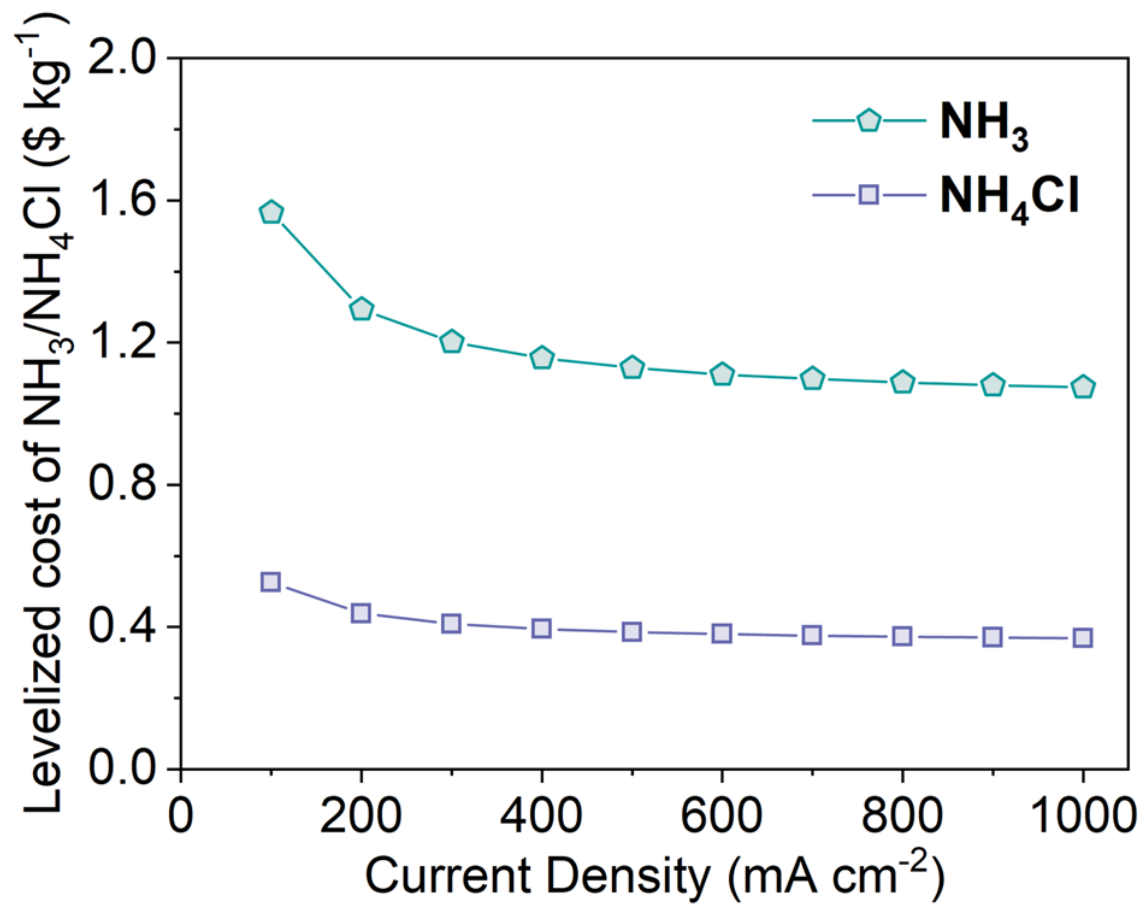

Supplementary Figure 72. Levelized production cost of NH_3 and NH_4Cl as a function of current density. Comparison of cost trajectories for direct ammonia production and downstream conversion to ammonium chloride under increasing current density.

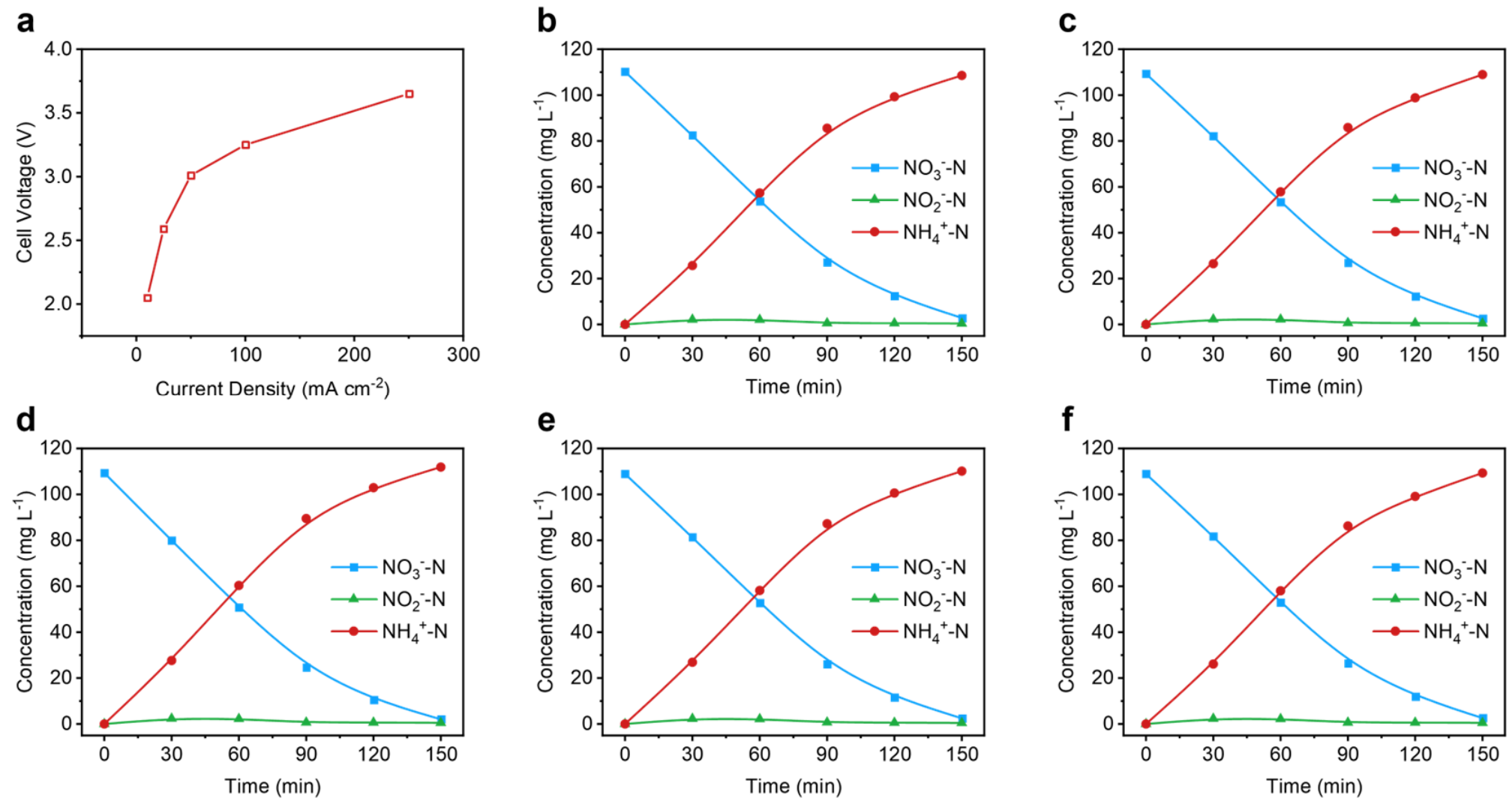

Supplementary Figure 73. (a) Cell voltage of the SNC-LSF/CP over a broad range of current densities and five sets (b-f) of time-dependent product distribution during the treatment of industrial wastewater using the MEA electrolyzer.

Supplementary Notes

Supplementary Note 1. Techno-economic assessment of electrochemical nitrate reduction reaction

The techno-economic analysis (TEA) in this study is developed to evaluate the economic viability of the electrochemical nitrate reduction reaction. Baseline operating conditions are selected based on expethe rimental performance in this study, including a current density of 1000 mA cm^{-2}, a total cell voltage of 2.23 V, an NH_3 Faradaic efficiency of 78.2%. The complete list of input parameters and mathematical formulations adopted in the TEA model are provided in **Supplementary Table 5-7**.

Supplementary Tables

Supplementary Table 1. Structural parameters extracted from the EXAFS fitting.

**Samples**	**Scattering path**	**CN**	**R(Å)**	**σ^2 (10^{-3} Å^2)**	**ΔE_0 (eV)**	**R factor**
LSF	Fe-O	5.31	1.94	5.15	-3.71	0.006
SNC-LSF	Fe-O	5.02	1.94	8.15	-4.15	0.012

Supplementary Table 2. Fitted parameters of the EIS result of SNC-LSF, LSF and LF.

**Catalyst**	**Potential (V vs RHE)**	**Rs (Ω)**	**T ($F\ s^{n+1}$)**	**R_1 (Ω)**	**n_1**	**R_2 (Ω)**	**Cφ (F)**
**SNC-LSF**	-0.3	3.656	0.0007638	58.7	0.86725	9.71	0.0125
	-0.35	3.632	0.00085457	50.13	0.85461	8.07	0.0131
	-0.4	3.643	0.00089204	30.99	0.84831	7.32	0.028
	-0.45	3.663	0.00083341	10.9	0.8679	6.18	0.0426
	-0.5	3.66	0.00071791	4.66	0.88955	5.37	0.054
**LSF**	-0.3	5.079	0.0006496	100.2	0.85411	13.24	0.006254
	-0.35	5.145	0.00052089	82.3	0.88041	13.53	0.0067378
	-0.4	5.051	0.00078737	54.1	0.83664	11.84	0.007125
	-0.45	5.062	0.0007467	17.64	0.85187	11.09	0.00834
	-0.5	5.064	0.00060733	6.222	0.87784	10.21	0.00934
**LF**	-0.3	4.114	0.00051249	597.9	0.739	32.83	0.0032
	-0.35	4.078	0.00059648	486.8	0.7269	31.48	0.0051
	-0.4	4.009	0.00068	276.9	0.71572	31.18	0.0057
	-0.45	4.016	0.00069461	132.2	0.72026	30.67	0.0063
	-0.5	4.071	0.00065362	65.51	0.73907	30.33	0.0069

Supplementary Table 3. Comparison of electrocatalytic NITRR performance for SNC-LSF with some of the state-of-the-art electrocatalysts.

catalyst	Electrolyte	Potential vs. RHE	FE_{NH3}%	NH_3 yield rate ($mg\ cm^{-2}\ h^{-1}$)	References
SNC-LSF	0.1 M KOH with 8 mM KNO_3	-0.2	57.18	0.06	This work
		-0.4	96.19	1.31	
		-0.6	70.33	2.49	
		-0.8	52.62	3.17	
	0.1 M KOH with 16 mM KNO_3	-0.2	50.16	0.05	
		-0.4	92.19	1.32	
		-0.6	84.27	3.19	
		-0.8	69.42	4.63	
	0.1 M KOH with 32 mM KNO_3	-0.2	40.32	0.08	
		-0.4	90.25	1.66	
		-0.6	91.07	3.94	
		-0.8	87.78	6.61	
	0.1 M KOH with 100 mM KNO_3	-0.2	80.65	0.22	
		-0.4	91.1	2.04	
		-0.6	92.29	5.21	
		-0.8	96.7	8.66	
	1 M KOH with 100 mM KNO_3	-0.2	83.62	1.24	
		-0.4	93.77	9.17	
		-0.6	94.95	23.34	
		-0.8	95.35	34.56	
	1 M KOH with 1 M KNO_3	-0.2	89.48	3.00	
		-0.4	95.41	15.25	
		-0.6	96.17	32.24	
		-0.8	97.81	51.37	
$FeNi_{500}$/FF	885.7 ppm NO_3^- with 0.05 M Na_2SO_4	-1.1	65.2	0.213	*Nat. Water.* **1**, 1068-078 (2023).
Fe-CNS SAC	0.02 M Na_2SO_4 with 1.6 mM $NaNO_3$	-0.57	78.4	0.2	*Proc. Natl. Acad. Sci. U.S.A.* **118**, e2105628118 (2021).
RuFe NFs	0.01 M $NaNO_3$ with 0.5 M Na_2SO_4	-0.65	74.4	1.98	*Proc. Natl. Acad. Sci. U.S.A.* **120**, e2306461120 (2023).
Co-Fe@Fe_2O_3	0.1M Na_2SO_4 with 500 ppm $NaNO_3$	-0.75	85.2	1.53	*Proc. Natl. Acad. Sci. U.S.A.* **119**, e2115504119 (2022).
Cu-PTCDA	0.1M PBS with 500 ppm KNO_3	-0.4	77	0.44	*Nat. Energy.* **5**, 605-613 (2020).
1T-MoS_2	620 ppm NO_3^- with 0.5M Na_2SO_4	-0.35	88.12	0.653	*Angew. Chem. Int. Ed.* **63**, e202315109 (2024).
Strained Ru	1 M KOH with 1 M KNO_3	-0.8	100	19.89	*J. Am. Chem. Soc.* **142**, 7036-7046 (2020).
FePc/TiO_2-2	0.1 M HNO_3 with 0.4 M	-0.8	90.6	17.4	*Nat. Commun.* **14**, 8036 (2023).

	KNO_3				
CoRu	1 M KOH with 0.1 M KNO_3		98.8	20.91	*Nat. Catal.* **6**, 402-414 (2023).
Cu-Ni tandem	0.1 M NO_3^- with 0.01M KOH and 0.5M $NaSO_4$	-1	88	9.911	*Angew. Chem. Int. Ed.* **62**, e202217337 (2023).
Fe/Cu-NG	1 M KOH with 0.1 M KNO_3	-0.5	92.51%	4.41	*Nat. Commun.* **14**, 3634 (2023).
Fe-HESA NCs	0.5 M Na_2SO_4 with 100 mM $NaNO_3$	-0.6	93.4	4.07	*Nat. Commun.* **15**, 6932 (2024).
Cu-N-C SAC	0.1 M KOH with 0.1 M KNO_3	-1	84.7	4.5	*J. Am. Chem. Soc.* **144**, 12062-12071 (2022).
Fe-PPy SACs	0.1 M KOH with 0.1 M KNO_3	-0.6	100	2.018	*Energy Environ. Sci.* **14**, 3522-3531 (2021).
FTO-E	PBS with 0.1 M $NaNO_3$	-1	87.5	1.313	*Angew. Chem. Int. Ed.* **62**, e202215782 (2023)
Co_4Fe_6	500 ppm NO_3^--N with 0.1 M Na_2SO_4	-0.69	98.6	2.706	*J. Am. Chem. Soc.* **147**, 8893 (2025).
Ni-P HCs	0.5 M Na_2SO_4 with 0.1 M $NaNO_3$	-0.8	93.7	10.37	*J. Am. Chem. Soc.* **147**, 12228 (2025).
Ni-WS_2	0.1 M KNO_3 with 1 M KOH	−0.7	91.7	23.3	*J. Am. Chem. Soc.* **147**, 27708-27719 (2025).
Co-TATB	0.1 M Na_2SO_4 with 0.1 M KNO_3	-0.8	95.3	1.84	*J. Am. Chem. Soc.* **147**, 18327-18337 (2025).
E-CuBDC	1 M KOH with 0.1 M KNO_3	-0.65	96.6	15.7	*J. Am. Chem. Soc.* **147**, 21432-21442 (2025).
HUST-38	0.1 M NO_3^- with 0.5 M K_2SO_4	-0.6	95.7	2.01	*J. Am. Chem. Soc.* **147**, 29949-29960 (2025).
Ni_6@CuFe-LDH	1 M KOH with 0.1 M KNO_3	-0.5	97	9.35	*J. Am. Chem. Soc.* **147**, 22785-22795 (2025).
$(La_{0.2}Pr_{0.2}Nd_{0.2}Ba_{0.2}Sr_{0.2})CoO_{3-\delta}$	0.1 M KNO_3 with 0.05 M K_2SO_4	-0.7	76	1.18	*J. Am. Chem. Soc.* **147**, 3119-3128 (2025).
RuCu DAs/NGA	0.1 M KOH with 0.1 M KNO_3	-0.4	95.7	3.1	*Nat. Commun.* **16**, 2167 (2025).
Cu@$In(OH)_3$	0.1 M NO_3^- with 0.1 M KOH and 0.1 M NO_3^-	-0.6	97.35	7.726	*Nat. Commun.* **16**, 8940 (2025).
Fe-TiO_2	0.1 M KOH with 0.1 M KNO_3	-0.5	97	8.23	*Nat. Commun.* **16**, 1129 (2025).
PdCuNiCoZn	1 M KOH with 0.1 M KNO_3	-0.8	99	22.35	*Nat. Commun.* **16**, 7915 (2025).
Co-3	0.1 M KOH with 0.1 M KNO_3	-1.2	92	22.79	*Adv. Mater.* **37**, e17303 (2025).

p-CN-Cu^5La^n-m	1 M KOH with 0.5 M KNO_3	-0.45	97.7	6.97	*Adv. Mater.* **37**, 2415632 (2025).
MnFeCoNiCu HEA	0.5 M Na_2SO_4 with 0.1 M NO_3^-	-0.6	94.5	1.02	*Adv. Mater.* **37**, 2415739 (2025).
Ca-Cu_2O-OA	0.5 M Na_2SO_4 with 0.1 M $NaNO_3$	-0.9	97.49	15.02	*Angew. Chem. Int. Ed.* **64**, e202509090 (2025).
Ce/Co_3O_4	1 M KOH with 0.1 M KNO_3	-0.4	97.8	3.423	*Angew. Chem. Int. Ed.* **64**, e202510478 (2025).
CoFe-cMOFs	1 M KOH with 1 M KNO_3	-0.7	94.3	17.3	*Angew. Chem. Int. Ed.* **64**, e202510665 (2025).
Fe_1Mo_1Pd	1 M KOH with 1 M KNO_3	-0.7	94.6	22.78	*Angew. Chem. Int. Ed.* **64**, e202509303 (2025).
Fe_8Zn_1PPc	1 M NaOH with 0.5 M $NaNO_3$	-0.85	99.02	45.56	*Angew. Chem. Int. Ed.* **64**, e202506712 (2025).

Supplementary Table 4. Comparison of electrocatalytic NITRR-OER MEA performance for SNC-LSF with reported electrocatalysts.

Catalyst	C NO_3^- (mM)	*J* (mA cm^{-2})	Cell voltage (V)	Stability(h)	References
SNC-LSF	1000	1000	2.23	240	**This work**
I_1Cu_4	1000	1000	3.58	110	*Proc. Natl. Acad. Sci. U.S.A.* **121**, e2405236121 (2024).
Ru/Cu_2O	1000	626	4.20	72	*J. Am. Chem. Soc.* **146**, 668-676 (2024).
Cu_1Ru_{10}	500	600	2.70	100	*ACS Catal.* **14**, 18223-18236 (2024).
CuCo NW	300	1000	3.20	200	*Adv. Funct. Mater.* **34**, 2405179 (2024).
CoNi array	32	1000	3.50	100	*Nat. Commun.* **14**, 1619 (2023).
Co-Cu SCC	1000	/	4.50	24	*Adv. Sci.* **11**, 2407250 (2024).
Cu_2O@CoO	100	375	2.30	/	*ACS Nano* **18**, 20258-20267 (2024).
Co_6Ni_4	300	300	2.20	240	*Nat Commun* **16**, 6161 (2025).
Ag/N-WO_3	500	480	3.50	/	*Angew. Chem. Int. Ed.* e202507696 (2025).
Ru-$Co(OH)_2$	32	500	2.60	100	*Energy Environ. Sci.* **18**, 5622-5631 (2025).

Supplementary Table 5. Baseline, optimistic, and pessimistic input values for single-variable sensitivity analysis in the TEA of nitrate-to-ammonia electrochemical synthesis.

Sensitivity parameter	Better	Base	Worse
**NH_3 FE (%)**	95	78.2	65
**Voltage (V)**	1.5	2.23	3.0
**Current density (mA cm^{-2})**	1500	1000	500
**Electric price ($ MWh^{-1})**	10	20	30

Supplementary Table 6. Core mathematical equations applied in the TEA analysis.

Description	Unit	Mathematical Formula
Operating Cost		
Total current needed	A	$\frac{NH_3\ production\ \left[\frac{ton}{day}\right] \times Electrons\ transferred \times\ Faraday's\ constant}{Product\ molecular\ weight\ \left[\frac{ton}{day}\right] \times 24 \left[\frac{h}{day}\right] \times 3600 \left[\frac{s}{h}\right] \times\ Faradaic\ efficiency[\%]}$
Electricity cost	$/ton	$\frac{Total\ current\ needed\ [A] \times\ Cell\ voltage\ [V] \times\ Electricity\ price \left[\frac{\$}{kWh}\right]}{NH_3\ production\ \left[\frac{ton}{day}\right]}$
Distillation operating cost	$/ton	$\frac{Reference\ operating\ cost \left[\frac{\$}{day}\right] \times Inlet\ flowrate\ \left[\frac{L}{min}\right]}{Reference\ flowrate\ \left[\frac{L}{min}\right] \times NH_3\ production\ \left[\frac{ton}{day}\right]}$
Other operating cost	$/ton	$10\% \times Electricity\ cost\ \left[\frac{\$}{ton}\right]$
Capital Cost		
Electrolyzer cost	$/ton	$\frac{Capital\ recovery\ factor \times\ Total\ Electrolyzer\ Cost\ [\$]}{Capacity\ factor \times 365\ \left[\frac{day}{year}\right] \times NH_3\ production\ \left[\frac{ton}{day}\right]}$
Catalyst & membrane cost	$/ton	$\frac{Capital\ recovery\ factor \times\ Total\ Electrolyzer\ Cost\ [\$] \times One\ time\ cost}{Capacity\ factor \times 365\ \left[\frac{day}{year}\right] \times NH_3\ production\ \left[\frac{ton}{day}\right]}$
Distillation capital cost	$/ton	$\frac{Capital\ recovery\ factor \times \left(\frac{Aqueous\ solution\ flowrate\ \left[\frac{L}{min}\right]}{Reference\ \ flowrate\ \left[\frac{L}{min}\right]}\right)^{Scaling\ factor}}{Capacity\ factor \times 365\ \left[\frac{day}{year}\right] \times NH_3\ production\ \left[\frac{ton}{day}\right]}$
Installation cost	$/ton	$Lang\ factor \times Total\ capital\ costs\ \left[\frac{\$}{ton}\right]$
Balance of plant cost	$/ton	$BoP\ factor \times Total\ capital\ costs\ \left[\frac{\$}{ton}\right]$
Capital recovery factor		$\frac{Discount\ rate\ \ \times (1+Discount\ rate)^{lifetime}}{(1+Discount\ rate)^{lifetime}-1}$
Chemicals Cost		
H_2O cost	$/ton	$\frac{1\ tonne\ NH_3 \times Molar\ mass\ H_2O\ \left[\frac{g}{mol}\right] \times Molar\ ratio\ H_2O \times\ H_2O\ price \left[\frac{\$}{ton}\right]}{Molar\ mass\ NH_3\ \left[\frac{g}{mol}\right]\ \times NH_3\ production\ \left[\frac{ton}{day}\right]}$
KOH/KNO_3 cost	$/ton	$\frac{Capital\ recovery\ factor \times Electrolyte\ volume[L] \times Molar\ mass\ KOH\ \left[\frac{g}{mol}\right]\ \times KOH\ price \left[\frac{\$}{kg}\right]}{Capacity\ factor \times 365\ \left[\frac{day}{year}\right] \times NH_3\ production\ \left[\frac{ton}{day}\right]}$

Supplementary Table 7. Parameter selection for the TEA analysis.

Parameter	Unit	Value	Reference and note
Ammonia production	ton day^{-1}	100	Basic unit of this analysis
Electrons transferred		8	Based on chemical reactions
Faradaic efficiency	%	78.2	Experiment results
Cell voltage	V	2.23	Experiment results
Electricity price	$ kWh^{-1}	0.02	*Nat. Commun.* **15**, 10351 (2024).
Reference flowrate	L min^{-1}	1000	*Joule* **8**, 1478-1500 (2024)
Life time	year	20	*ACS Energy Lett.* **9**, 2303-2309 (2024).
Discount rate	%	7	*Nat. Sustain.* **4**, 911-919 (2021).
Capacity factor	/	0.9	*Energy Environ. Sci.* **18**, 2804-2816 (2025).
One time cost	%	5	*J. CO_2 Util.* **34**, 490-499 (2019).
Long factor	/	1	*ACS Energy Lett.* **6**, 997-1002 (2021).

Supplementary Table 8. Deter concentration of substances in industrial wastewater (Shaanxi Chemical Industry Research Institute Co., Ltd.)

Parameters	Value
Initial pH	9.36
Electrical conductivity (mS cm^{-1})	37.0
Chemical oxygen demand (mg L^{-1})	290.06
NO_3^--N	113.35 mg L^{-1}
NO_2^--N	0.0
NH_4^+-N	5.3 mg L^{-1}
Na^+	9875.0 mg L^{-1}
K^+	526.1 mg L^{-1}
Mg^{2+}	133.5 mg L^{-1}
Ca^{2+}	5.28 mg L^{-1}
Cl^-	2602.8 mg L^{-1}
SO_4^{2-}	7969.4 mg L^{-1}
