# Supplementary material for: Hydroxyl-rich nanocavities on perovskite enable nearly barrierless intramolecular hydrogen transfer for nitrate electroreduction to ammonia
Source: Nat Commun. 2026 Jun 29;17:8062. doi: 10.1038/s41467-026-74405-1 (PMC13454618; doi:10.1038/s41467-026-74405-1)
Supplement: Supplementary file 2 [file 41467_2026_74405_MOESM2_ESM.pdf]

Description of Additional Supplementary Files

File Name: Supplementary Data 1

Description: Atomic coordinates of the optimized computational models of LSF, SNC-LSF, and LF.

File Name: Supplementary Data 2

Description: Atomic coordinates of the optimized computational models of representative snapshots of the interfacial H_2O for LF, LSF and SNC-LSF.

File Name: Supplementary Data 3

Description: Atomic coordinates of the optimized computational models of hydrogen transfer through the intramolecular hydrogen-bond network with the change of initial, transition and final state structures for SNC-LSF.
