# Supplementary material for: Hydroxyl-rich nanocavities on perovskite enable nearly barrierless intramolecular hydrogen transfer for nitrate electroreduction to ammonia
Source: Nat Commun. 2026 Jun 29;17:8062. doi: 10.1038/s41467-026-74405-1 (PMC13454618; doi:10.1038/s41467-026-74405-1)
Supplement: Supplementary file 3 [file 41467_2026_74405_MOESM6_ESM.pdf]

nature portfolio

Peer Review File

Hydroxyl-rich nanocavities on perovskite enable nearly barrierless intramolecular hydrogen transfer for nitrate electroreduction to ammonia

Corresponding Author: Professor Yinlong Zhu

This file contains all reviewer reports in order by version, followed by all author rebuttals in order by version.

Attachments originally included by the reviewers as part of their assessment can be found at the end of this file.

Version 0:

Reviewer comments:

Reviewer #1

(Remarks to the Author)
Attached.

Reviewer #2

(Remarks to the Author)
This study reports the construction of hydroxyl-rich nanocaves on perovskite surfaces for the efficient electrolytic reduction of nitrate to ammonia. Taking $La_{0.4}Sr_{0.6}FeO_{3-\delta}$ as an example, nanocaves formed by selective Sr dissolution enable rapid *H transfer, thereby achieving efficient and stable ammonia synthesis. This work proposes a novel concept for interface construction in the electrolytic reduction of nitrate to ammonia. However, the manuscript contains some confusing points in its logic and mechanism sections, detailed as follows:
1. This study repeatedly emphasizes the importance of nanocaves in NITRR, yet structural characterization of the catalyst remains insufficient. While AFM can demonstrate significant changes in roughness before and after leaching, close-up views of the nanocaves and quantitative data regarding their distribution, depth, or density remain equally curious.
2. This study appears to construct nanocaves by leaching Sr from the catalyst via electrochemical reduction. When NITRR occurs, this implies that nanocave formation and ammonia synthesis proceed simultaneously. So how should the stability of this catalyst be interpreted? It is necessary to specify whether the NITRR in this study was conducted after Sr leaching or concurrently with leaching.
3. Based on this paper's interpretation of the reaction mechanism, La in the catalyst serves as the water cleavage site, while Fe acts as the NITRR site. However, in DFT calculations, both La and Fe are identified as *NO_3 adsorption sites. Please clarify the functions of each active site on the catalyst.
4. The logical sequence of this manuscript is quite peculiar. I do not think the content of Section 2 does not belong the heading of Section 3: NITRR mechanism investigations. Furthermore, the manuscript places the NITRR performance of this catalyst in Section 4. A good electrocatalyst should first present its performance to the reader, rather than constantly explaining the mechanism. Please reorganize the logical flow of this study.
5. This study highlights a barrierless hydrogen transfer mechanism. How can this phenomenon be demonstrated experimentally? What interface properties are associated with its occurrence?
6. The in-situ Raman spectra in Fig. 2a show Raman peaks at different voltages are compressed too flat to discern any significant differences. The correlation between this and Fig. 2b is unconvincing.
7. In Fig. 2h, the DEMS measurements show that pre-hydroxylation of SNC-LSF in H_2O versus D_2O leads to a nearly one order of magnitude difference in ammonia peak intensity. What causes this difference?
8. This study appears to have replaced metal sites to demonstrate the universality of nanocaves in *H rapid transfer within NITRR. However, the data presentation and discussion for this section are not visible in the main text. This content could provide strong support for the innovative aspects of this research.

Reviewer #3

(Remarks to the Author)
The authors developed a surface nano-concaved $La_{0.4}Sr_{0.6}FeO_{3-δ}$ (SNC-LSF) catalyst via electrochemical reduction-assisted selective Sr^{2+} leaching. The formed nanocavities possess hydroxyl-enriched walls that establish an intramolecular hydrogen-bond network, enabling a nearly barrierless hydrogen transfer pathway. This leads to exceptional nitrate-to-ammonia conversion performance, achieving a Faradaic efficiency of 97.81% and an NH_3 yield rate of 51.37 mg h^{-1} cm^{-2} at –0.8 V vs. RHE. This work explores a timely issue with practical importance, and its results is of interest to Nature Communications readers. However, several key aspects require further clarification and strengthening to fully substantiate the claims and contextualize the significance of the findings within the existing literature.
1. The presentation of Figure 1d is currently not intuitive, which could lead to reader confusion or misinterpretation. To address this, it is recommended to revise the figure into a bar chart for a clearer and more intuitive representation of the data.
2. The author mentioned the following content. Is there any corresponding supporting literature or theoretical data to support this statement? "The slight differences in NITRR activity among SNC- LCF, SNC-LSF, and SNC-LBF may be attributed to variations in the leaching rates and ionic radii of Ca2+, Sr2+ and Ba2+."
3. The authors mention that SNC-LSF can provide abundant active H* species. Given such a high concentration of H*, why does the hydrogen evolution reaction (HER) not occur preferentially, while the more complex nitrate reduction reaction is instead favored?
4. Is there a competitive adsorption relationship between active H* and *NO3?
5. Why does potassium thiocyanate (KSCN) selectively poison only the Fe sites in the catalyst without interacting with other metals? Is this claim supported by relevant literature?
6. The authors are advised to provide a thorough and well-reasoned explanation for the computational results presented in the article to avoid any potential misinterpretation by readers. "We found that only the *+ NO3 - → *NO2 step was an endothermic process with a quite low energy barrier of just 0.10 eV. At the same time, *NH3 desorption becomes energetically barrierless (0 eV), thereby facilitating product release and regeneration of active sites."
7. There is an error in the labeling of Figure S61 that should be corrected.

Version 1:

Reviewer comments:

Reviewer #1

(Remarks to the Author)
I thoroughly reviewed the revised manuscript. The authors diligently conducted the additional experiments requested by the reviewers and clearly addressed their concerns. In sum, I recommend that the revised manuscript be accepted for publication as is.

Reviewer #2

(Remarks to the Author)
The authors have addressed most comments from other reviewers regarding material characterization, structural stability, logical organization, and figure presentation. However, the following critical scientific and methodological concerns still remain fully unaddressed and must be resolved to ensure the reliability and validity of the core conclusions:
1. The isotope-labeling DEMS results still cannot distinguish whether the deuterated ammonia signals (m/z = 17, 18, 19, 20) arise from lattice Fe–OD/La–OD hydroxyl groups or merely *physically adsorbed D species on the catalyst surface. No control experiment or theoretical justification is provided to identify the genuine source of deuterons, leaving this key mechanistic evidence unsubstantiated.
2. The proposed hydrogen tunneling effect and Grotthuss-like hydrogen transfer mechanism still lack rigorous and direct evidence. Although the authors supplemented hydrogen transfer activation energy data, they provided no experimental or computational verification for hydrogen tunneling, and the Grotthuss mechanism is not convincingly validated.
3. The H/D kinetic isotope effect (KIE) analysis remains seriously flawed with no correction. The authors neither excluded the interference caused by different cations (K^+ vs. Na^+) nor separated KIE contributions from nitrate reduction and the competing hydrogen evolution reaction (HER). Using the total KIE to support the intramolecular hydrogen-bond network is scientifically invalid.
4. The techno-economic analysis (TEA) still relies on unrealistic assumptions without modification: the cost calculation ignores wastewater pretreatment costs, the referenced Haber–Bosch ammonia price ($1.15 kg^{-1}) is unreasonably high, and the assumed electricity price ($0.02 kWh^{-1}) is inconsistent with actual market levels, making the claimed economic advantage unconvincing.
5. The significant XRD peak shift with Sr doping (Supplementary Figure 2) still lacks explanation regarding lattice distortion or phase evolution.
6. Basic XRD data for LCF, LBF, LSC, LSN, and LSM are still not provided to support the generality of hydroxyl-rich nanocavity formation.
7. The NITRR activity of carbon black used in electrodes has not been measured to exclude background contributions. To demonstrate the generality of hydroxyl-rich nanocavity formation via electrochemical reduction-assisted selective ion leaching, the authors substituted different A-site and B-site elements in the perovskite lattice. However, basic structural information (such as XRD patterns) for the corresponding perovskites (LCF, LBF, LSC, LSN, and LSM), are suggested to be given.
8. Standard calibration curves for NO_3^- quantification are still missing, undermining the reliability of selectivity calculations. The authors should be offered the standard curves for NO3- quantification, as it is essential for accurately evaluating product

selectivity.
9. The issue of ammonia crossover in MEA tests and its influence on Faradaic efficiency have not been addressed or corrected. For the tests in MEA device, the crossover of ammonia from cathode to anode probably occurred, which may lower the FE of product. Please explain how these issues were addressed or avoided.
10. (One minor comment) Figure 5d is partially overlapped with Figure 5e, please rearrange the layout to ensure these graphs are visibly displayed.

Reviewer #3

(Remarks to the Author)
The authors have resolved my concerns.

Version 2:

Reviewer comments:

Reviewer #2

(Remarks to the Author)
The authors have addressed all of the comments and I agree to take this manuscript for the possibility of publication.

Open Access This Peer Review File is licensed under a Creative Commons Attribution 4.0 International License, which permits use, sharing, adaptation, distribution and reproduction in any medium or format, as long as you give appropriate credit to the original author(s) and the source, provide a link to the Creative Commons license, and indicate if changes were made.
In cases where reviewers are anonymous, credit should be given to 'Anonymous Referee' and the source.
The images or other third party material in this Peer Review File are included in the article's Creative Commons license, unless indicated otherwise in a credit line to the material. If material is not included in the article's Creative Commons license and your intended use is not permitted by statutory regulation or exceeds the permitted use, you will need to obtain permission directly from the copyright holder.
To view a copy of this license, visit https://creativecommons.org/licenses/by/4.0/

Point-to-Point Responses to Reviewers' Comments and Suggestions

First, we sincerely thank all reviewers for their valuable comments and constructive suggestions, which have significantly improved the quality, clarity, and rigor of this manuscript. To address each concern clearly, we have organized the reviewers' comments point-by-point and provided detailed responses as follows.

Reviewer #1

GENERAL COMMENTS: This manuscript reports the rational design of a perovskite oxide with a nano-concave surface, $La_{0.4}Sr_{0.6}FeO_{3-\delta}$ (SNC-LSF), synthesized via electrochemical reduction-assisted selective Sr^{2+} leaching for efficient electrocatalytic nitrate reduction to ammonia. The authors propose a highly innovative hydrogen hopping mechanism, where a hydroxyl-rich nanocavity creates a nearly barrierless intramolecular hydrogen transfer pathway along hydrogen-bond networks. The reported performance (a Faradaic efficiency of 97.81% and an ammonia yield rate of 51.37 mg h^{-1} cm^{-2} at -0.8 V vs. RHE) is truly remarkable and surpasses most state-of-the-art NITRR catalysts. The mechanistic investigations are exceptionally thorough, integrating operando ATRSEIRAS, on-line DEMS, and AIMD/DFT simulations to substantiate the Grotthuss-like hydrogen transport mechanism. Furthermore, the practical demonstration of an industrial-level MEA electrolyzer powered by solar energy provides strong evidence for the technology's scalability and economic viability. However, despite the high quality of the work, there are a few minor points regarding the kinetic competition between NITRR and HER at high overpotentials and the long-term surface integrity that require further clarification to strengthen the manuscript's impact. Therefore, I recommend Revision.

Response: We are pleased that Reviewer #1 **recognized the novelty and significance of this work**. In particular, the reviewer **highlighted the outstanding catalytic performance**, noting that "*the reported performance ... is truly remarkable and surpasses most state-of-the-art NITRR catalysts.*" The **mechanistic depth of the study was also highly appreciated**, with the reviewer stating that "*the mechanistic*

investigations are exceptionally thorough, integrating operando ATR-SEIRAS, on-line DEMS, and AIMD/DFT simulations to substantiate the Grotthuss-like hydrogen transport mechanism." In addition, **the practical relevance of this work was acknowledged**, as the reviewer noted that "*the practical demonstration of an industrial-level MEA electrolyzer powered by solar energy provides strong evidence for the technology's scalability and economic viability.*" At the same time, the reviewer raised constructive comments regarding the kinetic competition between NITRR and HER and the long-term surface integrity. These points have been carefully considered, and additional analyses and discussions have been incorporated in the revised manuscript to further strengthen the clarity and impact of the study.

Comment 1: The authors report a remarkable Faradaic efficiency (FE) of 97.81% at a high negative potential of -0.8 V vs. RHE under 1000 mM nitrate conditions. However, the LSV curve for SNC-LSF in Figure 4a measured without NO_3^- clearly shows a significant current density for the HER at this potential range. Given this high intrinsic HER activity, could the authors provide a more detailed kinetic rationale for suppressing HER pathways to maintain such high selectivity? Specifically, I suggest including a quantitative comparison of reaction rate constants of NITRR versus HER or more detailed kinetic analyses at high overpotentials, as the Tafel data in Figure 2f appear to be collected under relatively low nitrate concentrations and may not fully represent the behavior at 1000 mM.

Response to C1: We thank the reviewer for this insightful comment regarding the potential competition between NITRR and HER at high overpotentials. We would like to clarify that the LSV curve measured in the absence of NO_3^- primarily reflects the intrinsic HER activity of the catalyst. In contrast, under NITRR conditions (*i.e.*, in the presence of high-concentration nitrate), the reaction environment is fundamentally altered. The introduction of NO_3^- leads to preferential adsorption and activation on undercoordinated Fe-O sites, effectively competing with and suppressing proton reduction pathways. To further evaluate the reaction kinetics, we performed a comparative Tafel analysis under nitrate-free and nitrate-containing conditions. As

shown in **Figure R1**, the Tafel slope in pure KOH (without NO_3^-) is 262 mV dec^{-1}, indicating sluggish HER kinetics on SNC-LSF. Notably, upon introducing 1000 mM NO_3^-, the Tafel slope **decreases significantly** to 152 mV dec^{-1}, suggesting that the overall reaction kinetics are markedly accelerated in the presence of nitrate. This pronounced change indicates a shift in the dominant reaction pathway from HER toward NITRR under high nitrate concentrations.

More importantly, the Faradaic efficiency directly reflects electron utilization during electrolysis. The exceptionally high FE (96.19%) observed at -0.4 V vs. RHE demonstrates that the majority of electrons are consumed by NITRR rather than HER, even at relatively negative potentials. This provides strong experimental evidence that nitrate reduction is the dominant reaction pathway under the applied conditions. In addition, previous studies have reported similar behavior, where nitrate effectively suppresses HER via competitive adsorption and pathway modulation (*Proc. Natl. Acad. Sci. U.S.A.* **119**, e2115504119 (2022). *Nat. Energy* **5**, 605-613 (2020). *Proc. Natl. Acad. Sci. U.S.A.* **118**, e2105628118 (2021). *Angew. Chem. Int. Ed.* **63**, e202315109 (2024). *Proc. Natl. Acad. Sci. U.S.A.* **120**, e2305489120 (2023)), further supporting our observations.

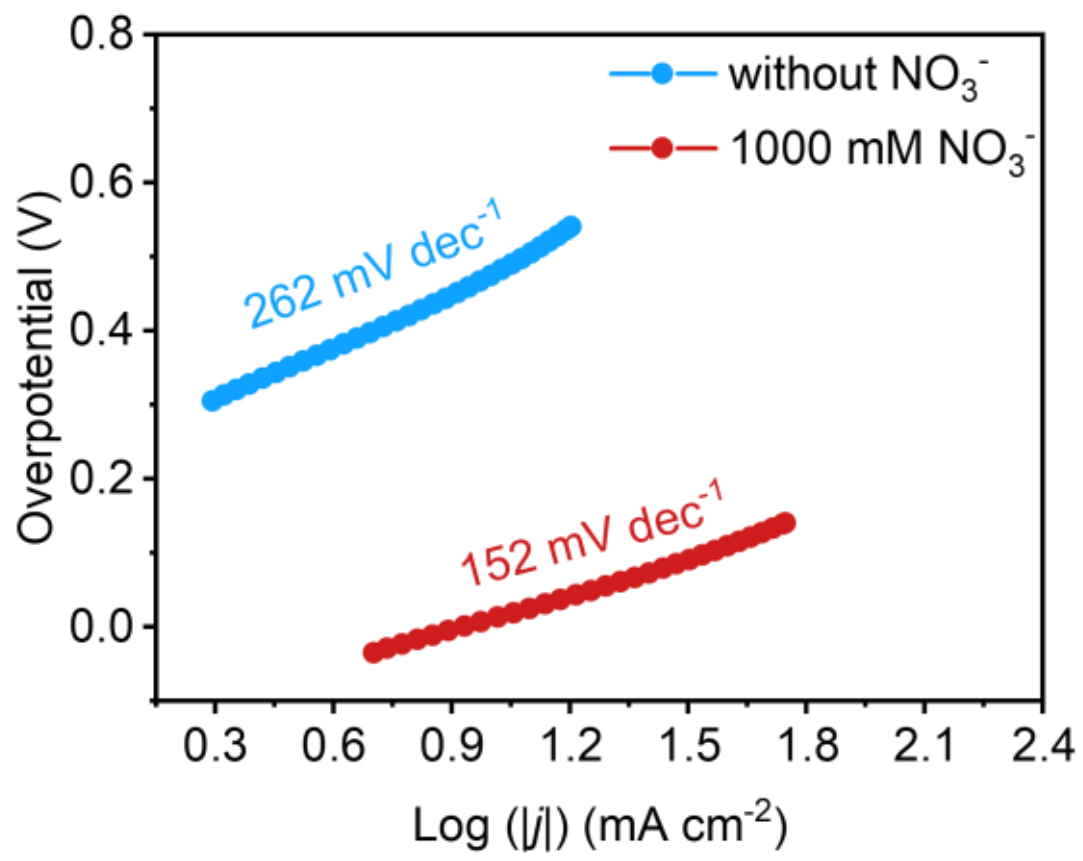

Figure R1. Tafel plots of SNC-LSF.

Comment 2: The amorphous reconstructed surface layer, approximately 10 nm in thickness, is presented as the cornerstone of the catalyst's high activity. Is there any possibility that this metastable amorphous structure, formed through Sr leaching, might

undergo recrystallization or transition back into an oxide form when the potential is removed or upon exposure to air? Furthermore, while the catalyst demonstrated impressive durability over 240 hours in the MEA system, could the authors provide HRTEM images obtained after the stability test to confirm that the 10 nm amorphous layer remains structurally intact and unchanged after such prolonged industrial-level operation?

Response to C2: We thank the reviewer for this important comment regarding the structural stability of the reconstructed amorphous surface layer. Concerning the possibility that the metastable amorphous structure may undergo recrystallization or revert to a crystalline oxide upon removal of the applied potential or exposure to air, additional *ex-situ* characterizations were conducted after electrolysis and subsequent air exposure. As shown in **Figures R2** and **R3**, XRD patterns reveal no emergence of new crystalline phases. Meanwhile, FTIR spectra still exhibit clear hydroxyl-related features. These results indicate that **the reconstructed amorphous layer remains stable under ambient conditions**.

Furthermore, in response to the reviewer's request for direct structural evidence after long-term operation, HRTEM measurements were performed on the catalyst following stability test in the MEA system. As shown in **Figure R4**, the ~10 nm amorphous surface layer is well preserved, with no observable recrystallization or structural collapse. This observation confirms that **the nanocavity-confined amorphous structure remains structurally intact even under prolonged industrial-level operating conditions**.

Collectively, these results demonstrate that the electrochemically reconstructed amorphous layer is not a transient metastable phase but instead represents a robust and stable surface structure under both reaction and post-exposure environments.

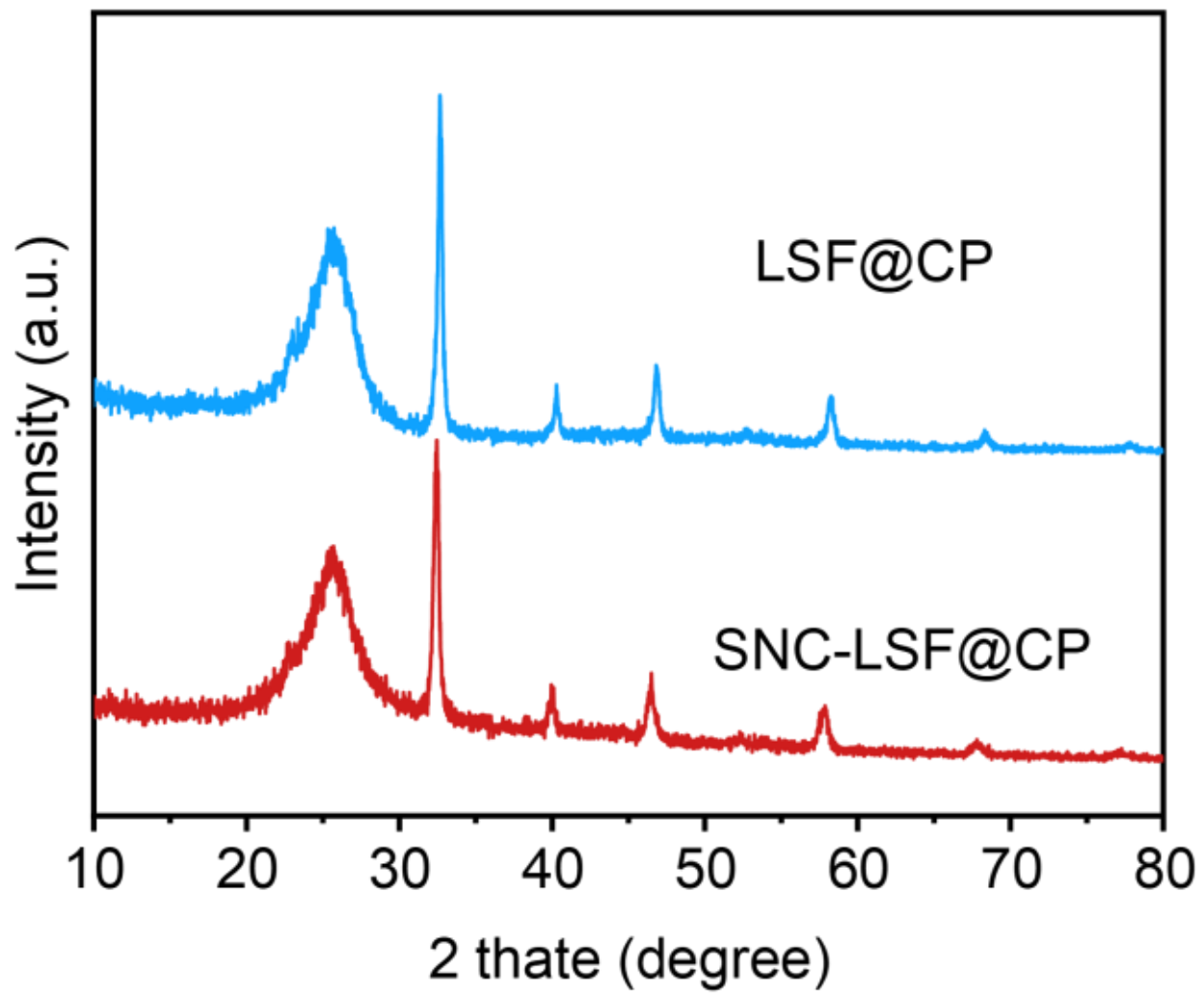

Figure R2 (also **Supplementary Fig. 57**). XRD spectra of SNC-LSF after stability test and exposure to air 24 h.

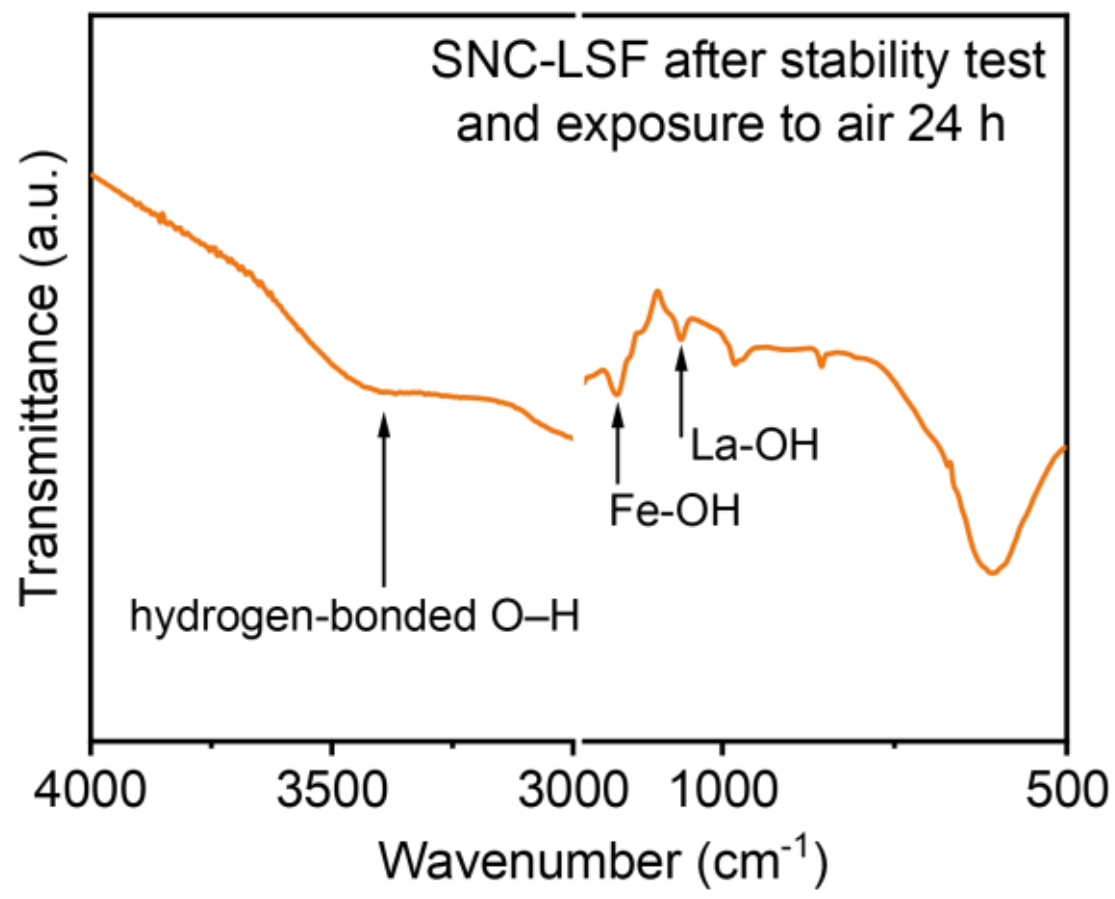

Figure R3 (also **Supplementary Fig. 59**). FTIR spectra of SNC-LSF after stability test and exposure to air 24 h.

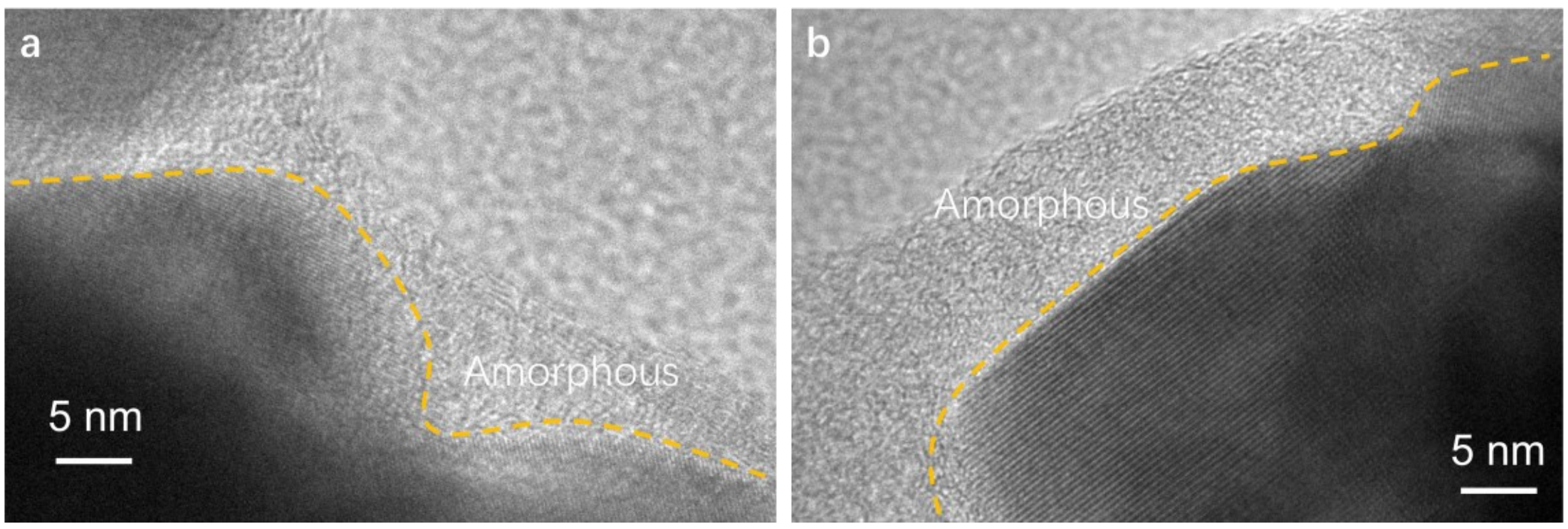

Figure R4 (also **Supplementary Fig. 62**). Two sets of HRTEM images of SNC-LSF after the stability test in the MEA system.

Related data (**Supplementary Fig. 57 and Supplementary Fig. 59, Supplementary Fig. 62**) and discussions (yellow-highlighted in **Line 589-608, Page 23-24** and **Line 636-639, Page 25**) have been added in the revised manuscript as follows:

Line 589-608, Page 23-24: *"As shown in **Supplementary Fig. 56-60**, we further conducted ICP, XRD, AFM, XPS, and FTIR measurements to provide evidences to support the durability of the SNC-LSF electrode after stability tests. Notably, during the cathodic one-hour nitrate reduction electrolysis, the Sr concentration in the electrolyte increases only within the initial stage and becomes nearly constant after ~20 min. Compared to the 1000-s electrocatalyst activation in Figure 1d, Sr dissolution during nitrate reduction electrocatalysis decreases by an order of magnitude, indicating that the formation of SNC-LSF is essentially completed prior to catalysis and the reconstructed structure remains stable thereafter. The post-reaction XRD pattern preserves the characteristic perovskite diffraction peaks with no detectable new crystalline phases upon air exposure. AFM images reveal a surface morphology similar to that of the pristine catalyst, with only a slight increase in roughness (4.079 nm). Moreover, FT-IR spectra confirm the persistence of La-OH/Fe-OH and intramolecular hydrogen-bond features, and XPS analyses show similar elemental compositions/ oxidation states with only a minor enhancement of the hydroxyl-related O 1s component. Collectively, these results demonstrate that the electrochemically induced amorphous nanocavity structure is not a transient metastable phase but rather a stable reconstructed surface under both operating and post-exposure conditions, further substantiating the excellent structural and chemical stability of SNC-LSF during NITRR operation and the durability of the proposed intramolecular hydrogen-bond reaction mechanism."*

Line 636-639, Page 25: *"In addition, HRTEM images after the 240-hour operation (**Supplementary Fig. 62**) confirm that the amorphous surface layer is well-preserved with no observable recrystallization or structural collapse, demonstrating the robustness of the nanocavity-confined amorphous layer under prolonged industrial-level conditions."*

Comment 3: The attempt to validate the catalyst's performance using real industrial wastewater is highly practical and adds significant value to this work. However, the experimental data using real wastewater appear to be insufficient. Please provide additional data, such as LSV curves or long-term stability test results, conducted in the presence of impurities from the wastewater.

Response to C3: We thank the reviewer for recognizing the practical relevance of testing the catalyst in real industrial wastewater and for the valuable suggestion. To strengthen this aspect, additional electrochemical measurements have been conducted under real wastewater conditions. As shown in **Figure R5a**, LSV curves collected in industrial wastewater exhibit comparable current responses to those in model electrolytes, indicating the maintained catalytic activity in the presence of impurities. Furthermore, repeated electrolysis tests of five consecutive cycles were performed in the MEA system (**Figure R5b-f**), which demonstrate stable ammonia production with negligible decay in activity and selectivity. These results confirm the strong tolerance of SNC-LSF toward coexisting ions and impurities, highlighting its robustness under realistic conditions.

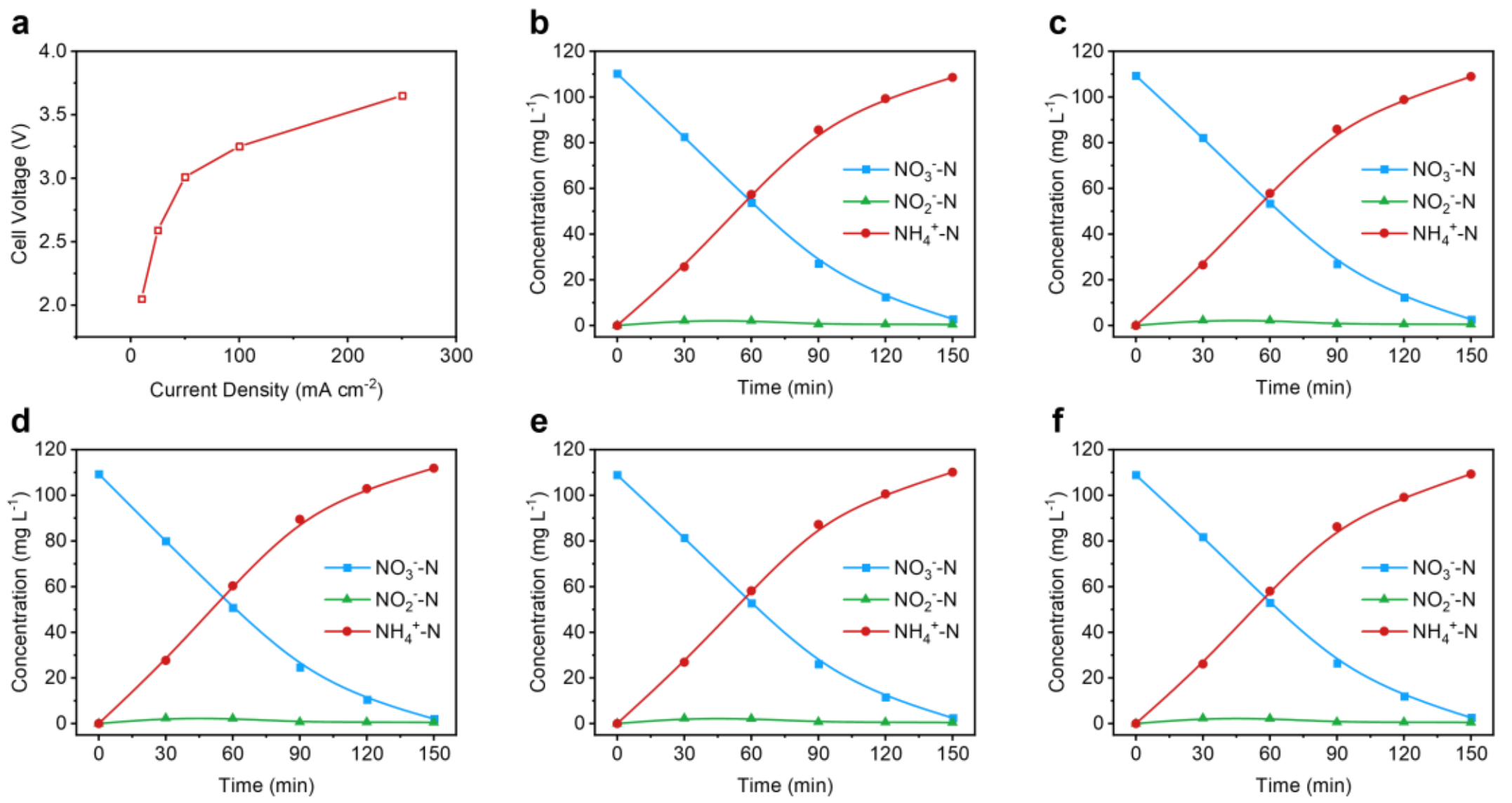

Figure R5 (also **Supplementary Fig. 67**). (a) Cell voltage of the SNC-LSF/CP over a broad range of current densities and five sets (b-f) of time-dependent product distribution during the treatment of industrial wastewater using the MEA electrolyzer.

Related data (**Supplementary Fig. 67**) and discussions (yellow-highlighted in **Line 700-715, Page 28**) have been added in the revised manuscript as follows:

Line 700-715, Page 28: *"Finally, industrial NO_3^--wastewater was employed to evaluate the practical NO_3^--to-NH_3 performance of SNC-LSF. Despite the complex composition of the wastewater including the presence of coexisting ions (e.g., Ca^{2+} and Mg^{2+}), SNC-LSF exhibits efficient nitrate reduction under electrochemical operation (**Supplementary Table 8**). To further assess its robustness under realistic conditions, additional electrochemical measurements were conducted. As shown in **Supplementary Fig. 67**, LSV curves collected in industrial wastewater indicate a maintained catalytic activity in the presence of impurities. Moreover, repeated electrolysis tests (five consecutive cycles) were performed in the MEA system, demonstrating stable ammonia production with negligible decay in activity and selectivity. Notably, the total nitrogen concentration substantially reduces from 113.35 mg N L^{-1} to 2.56 mg N L^{-1}, reaching the regulation levels in WTO (10 mg N L^{-1} for total nitrogen), while no detectable accumulation of the toxic intermediate NO_2^- was observed within the detection limit. Collectively, these results confirm the strong tolerance of SNC-LSF toward coexisting ions and impurities, highlighting its robustness under realistic conditions and demonstrating its strong potential for the treatment and resource utilization of NO_3^--containing wastewater."*

Comment 4: It is noted that the electrolyte was replenished throughout the 240-hour long-term test to compensate for nitrate depletion. Has any analysis been performed on the potential impact of accumulated reaction by-products or the possible re-adsorption of initially leached Sr^{2+} ions back onto the catalyst surface?

Response to C4: We thank the reviewer for this insightful comment regarding the potential impact of by-product accumulation and the re-adsorption of dissolved Sr^{2+} species during long-term operation. To address this concern, ICP analysis was conducted to monitor Sr concentration in the electrolyte during long-term operation. As shown in **Figure R6**, Sr concentration increases during the initial stage (within 4 h) and then stabilizes, indicating that **dissolved Sr^{2+} remains in solution without re-**

adsorption. (*J. Am. Chem. Soc.* **143**, 2741-2750 (2021); *Science* **353**, 1011-1014 (2016); *Nat. Mater.* **16**, 925-931 (2017); *Chem. Mater.* **32**, 4509-4517 (2020); *Small* **20**, 2404894 (2024)). Therefore, re-incorporation into the catalyst can be ruled out.

In addition, the accumulation of reaction by-products was carefully evaluated. The concentration of the primary intermediate NO_2^- was monitored during the first 6 hours of electrolysis, and **no noticeable accumulation was observed**, indicating that intermediate species are continuously consumed during the reaction (**Figure R7**). Combined with the periodic replenishment of the electrolyte to maintain nitrate concentration, these results confirm that **neither by-product accumulation nor Sr^{2+} re-adsorption** has a measurable impact on the catalytic performance or stability under the tested conditions.

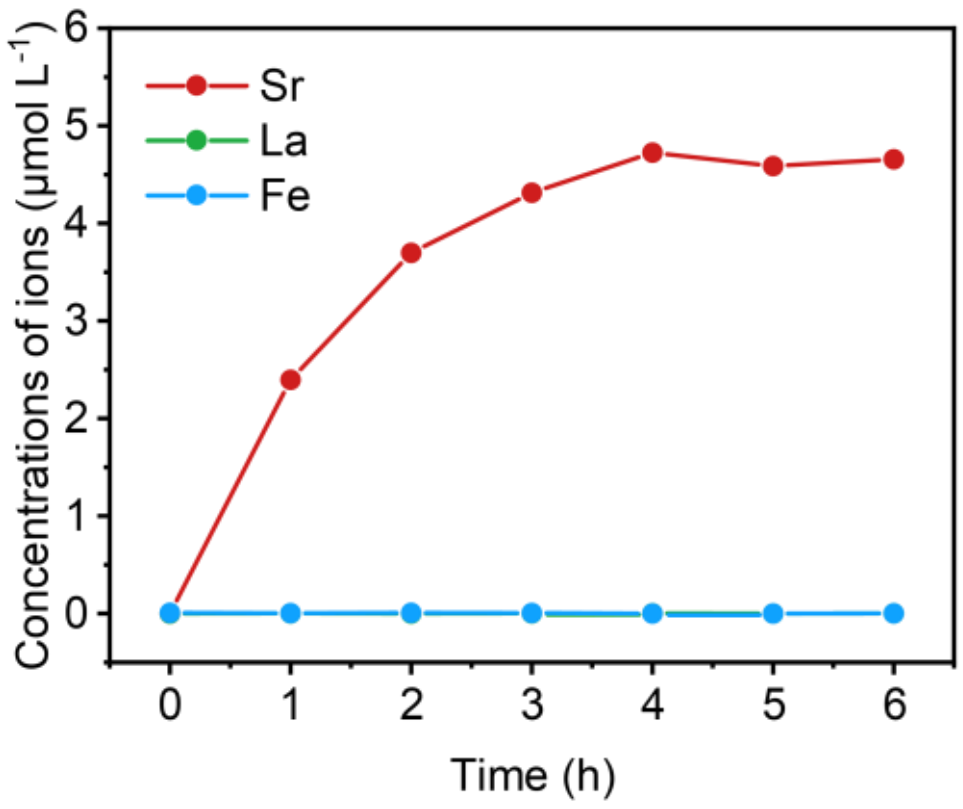

Figure R6 (also **Supplementary Fig. 63**). Dissolution concentrations of La, Sr and Fe elements from LSF in the MEA at 1 A cm^{-2}.

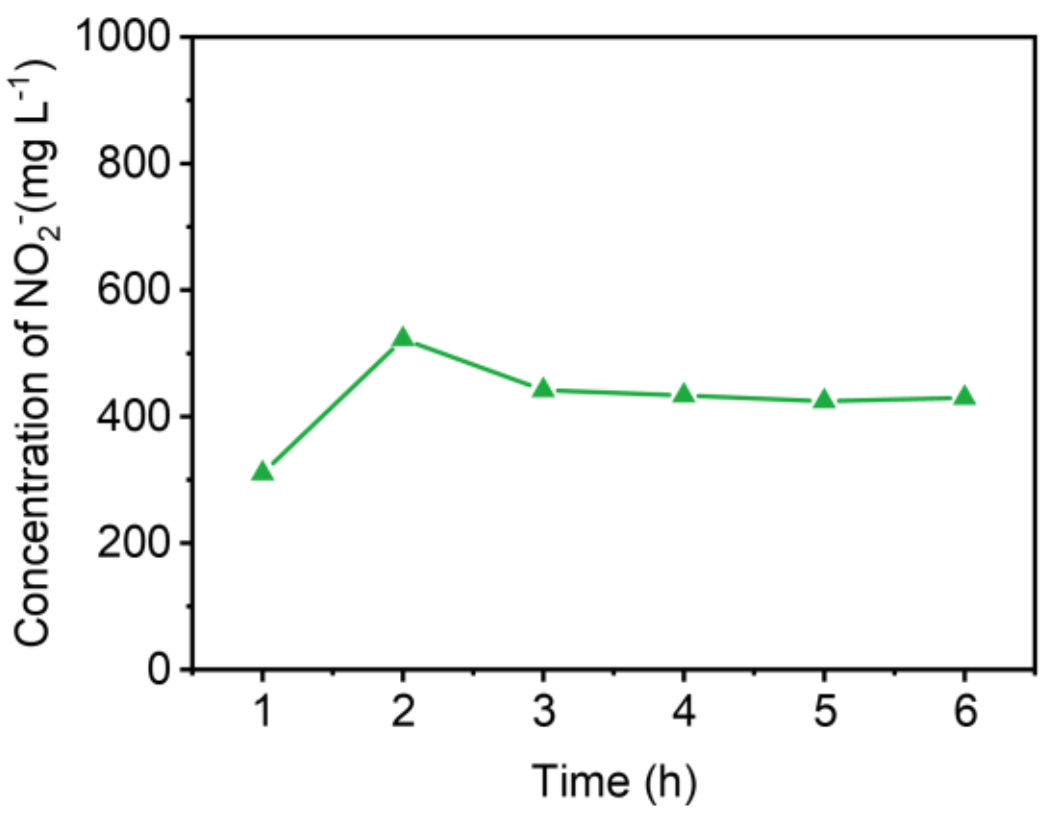

Figure R7 (also **Supplementary Fig. 64**). Time-dependent concentration of NO_2^- in the MEA at 1 A cm^{-2}.

Related data (**Supplementary Fig. 63** and **Supplementary Fig. 64**) and discussions (yellow-highlighted in **Line 639-651, Page 26**) have been added in the revised manuscript as follows:

Line 639-651, Page 26: *"To further assess the potential impact of by-product accumulation and possible re-adsorption of leached Sr^{2+} ions, additional analyses were conducted. ICP monitoring of the electrolyte during long-term operation (**Supplementary Fig. 63**) shows that the Sr concentration increases initially (within 4 h) and then stabilizes, indicating that the dissolved Sr^{2+} remains in solution without re-adsorption onto the catalyst surface, consistent with its high solubility in alkaline media and thermodynamic instability for re-deposition under cathodic conditions. Furthermore, the concentration of the primary intermediate NO_2^- was monitored during the first 6 hours of electrolysis (**Supplementary Fig. 64**), and no noticeable accumulation was observed, confirming that intermediate species are continuously consumed. The absence of performance decay over 240 h further corroborates that neither Sr^{2+} re-adsorption nor by-product accumulation adversely affects the catalytic performance or stability."*

Reviewer #2

GENERAL COMMENTS: This study reports the construction of hydroxyl-rich nanocaves on perovskite surfaces for the efficient electrolytic reduction of nitrate to ammonia. Taking $La_{0.4}Sr_{0.6}FeO_{3-\delta}$ as an example, nanocaves formed by selective Sr dissolution enable rapid *H transfer, thereby achieving efficient and stable ammonia synthesis. This work proposes a novel concept for interface construction in the electrolytic reduction of nitrate to ammonia. However, the manuscript contains some confusing points in its logic and mechanism sections, detailed as follows.

Response: We are pleased that Reviewer #2 **recognized the conceptual advance of this work**. The reviewer acknowledged that "*this work proposes a novel concept for*

interface construction in the electrolytic reduction of nitrate to ammonia," **highlighting the significance of constructing hydroxyl-rich nanocavities on perovskite surfaces**. It was further recognized that nanocavities formed via selective Sr dissolution enable rapid hydrogen transfer and contribute to efficient and stable ammonia synthesis. At the same time, the reviewer pointed out several concerns regarding the logical flow and mechanistic presentation. These comments have been carefully considered, and the manuscript has been revised to improve structural clarity and enhance the coherence of the mechanistic discussion, with clearer distinctions made between experimental observations and interpretation.

Comment 1: This study repeatedly emphasizes the importance of nanocaves in NITRR, yet structural characterization of the catalyst remains insufficient. While AFM can demonstrate significant changes in roughness before and after leaching, close-up views of the nanocaves and quantitative data regarding their distribution, depth, or density remain equally curious.

Response to C1: We thank the reviewer for this valuable comment regarding the structural characterization of nanocavities. **According to reviewer's suggestions**, we conducted additional high-resolution AFM measurements to provide more detailed morphological information. As shown in **Figure R8**, AFM images with a scan size of 150 × 150 nm have been included, offering clearer visualization of the nanocave features.

Furthermore, a quantitative analysis of nanocavity distribution has been performed. As shown in **Figure R9**, statistical evaluation indicates that, within a 0.5 × 0.5 μm region, approximately three nanocavities with sizes larger than 5 nm were observed along each edge on average. These results provide direct evidence of the density and uniform distribution of the nanocavities, thereby strengthening the structural characterization.

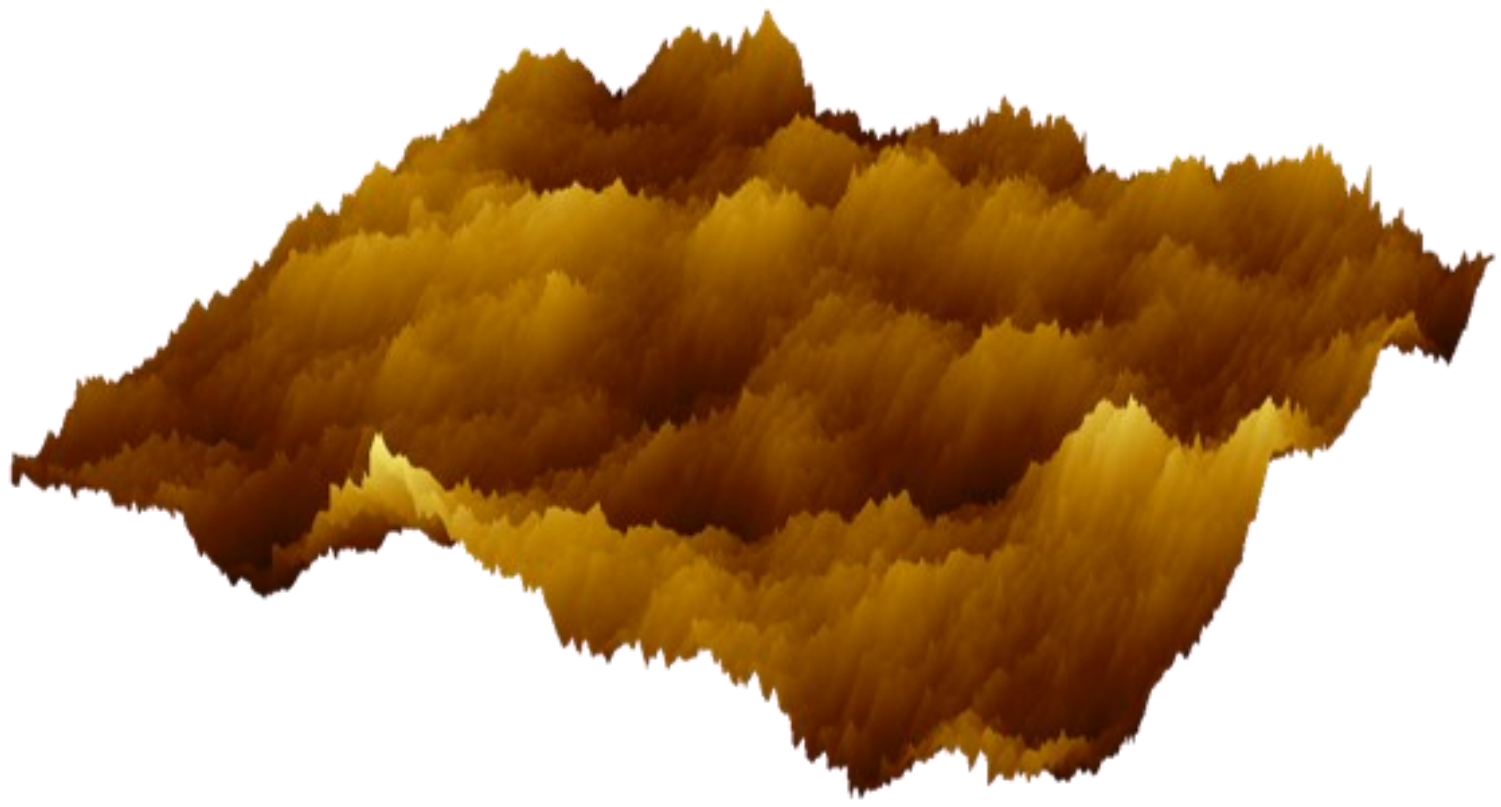

Figure R8 (also **Supplementary Fig. 10**). AFM image of SNC-LSF with a scan size of 150 × 150 nm.

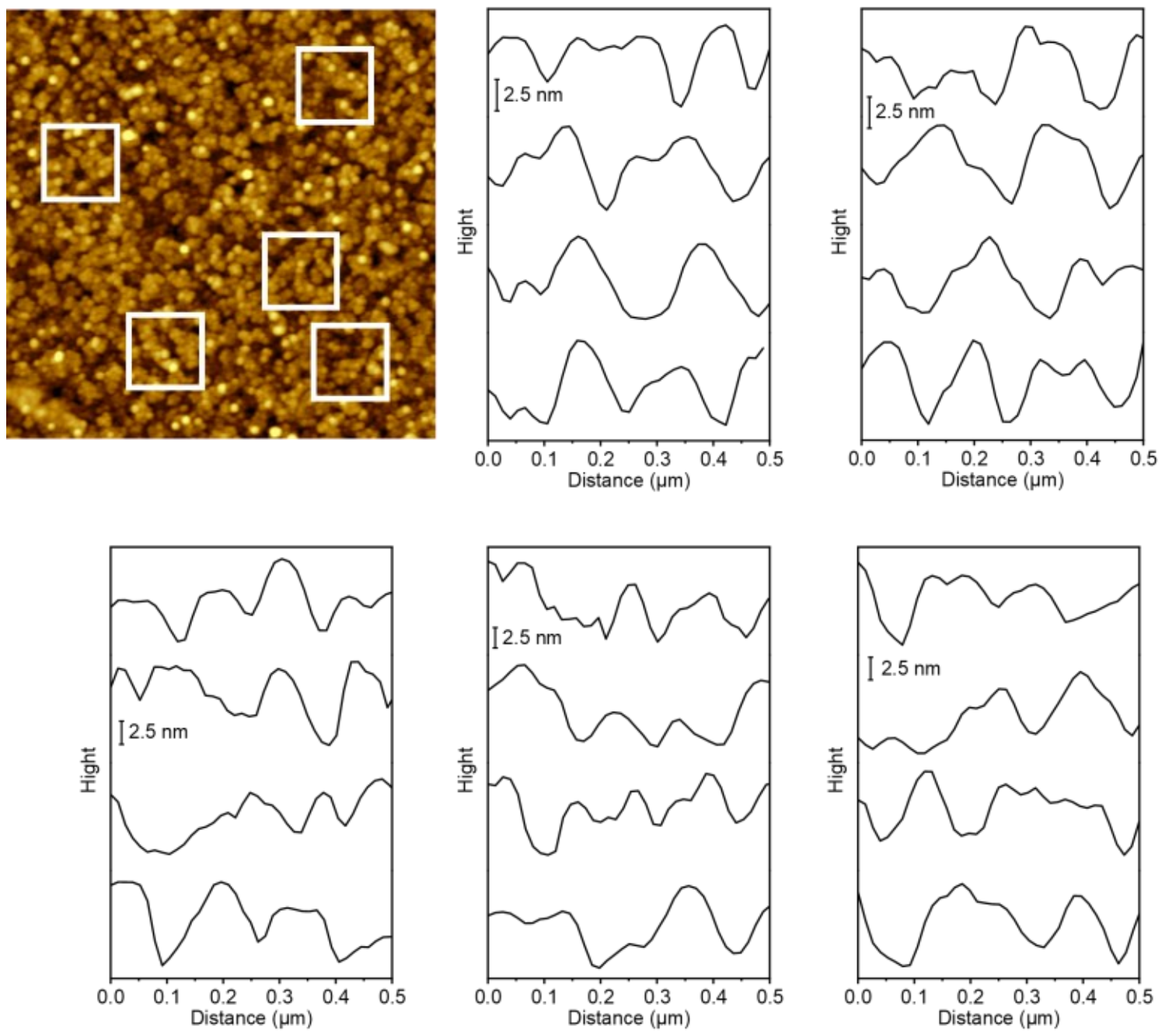

Figure R9 (also **Supplementary Fig. 11**). Quantitative statistics of the nanopore density in SNF-LSF.

Related data (**Supplementary Fig. 10** and **Supplementary Fig. 11**) and discussions (yellow-highlighted in **Line 207-211, Page 8-9**) have been added in the revised manuscript as follows:

Line 207-211, Page 8-9: *"High-resolution AFM images provide close-up views of the nanocavities (**Supplementary Fig. 10**). Quantitative AFM analysis further reveals that, within a 0.5 × 0.5 μm region, approximately three nanocavities with sizes larger than 5 nm were observed along each edge on average (**Supplementary Fig. 11**)."*

Comment 2: This study appears to construct nanocaves by leaching Sr from the catalyst via electrochemical reduction. When NITRR occurs, this implies that nanocave formation and ammonia synthesis proceed simultaneously. So how should the stability of this catalyst be interpreted? It is necessary to specify whether the NITRR in this study was conducted after Sr leaching or concurrently with leaching.

Response to C2: We thank the reviewer for this important comment regarding the relationship between Sr leaching and the NITRR process. We would like to clarify that the surface nano-concaved perovskite oxide catalyst (SNC-LSF) was **obtained prior to the NITRR measurements** via 1000-s cathodic polarization of the parent LSF, during which Sr was selectively leached to form the nanocavity structure. The as-prepared SNC-LSF **was then used for subsequent NITRR tests**. To further verify the structural stability during operation, the ionic composition of the electrolyte was analyzed during a 1-hour NITRR test using the pre-activated SNC-LSF (**Figure R10**). Compared to the 1000-s electrocatalyst activation in Figure 1d, Sr dissolution during nitrate reduction electrocatalysis decreases by an order of magnitude. Moreover, once Sr leaching reaches a certain extent, the catalyst structure tends to stabilize, as supported by the stability characterizations presented in the Supporting Information. These results demonstrate that the NITRR process is primarily conducted on **a pre-formed nanocavity structure, rather than** being dominated by continuous structural reconstruction, and thus the catalytic stability can be reliably interpreted based on the structural integrity of SNC-LSF during operation.

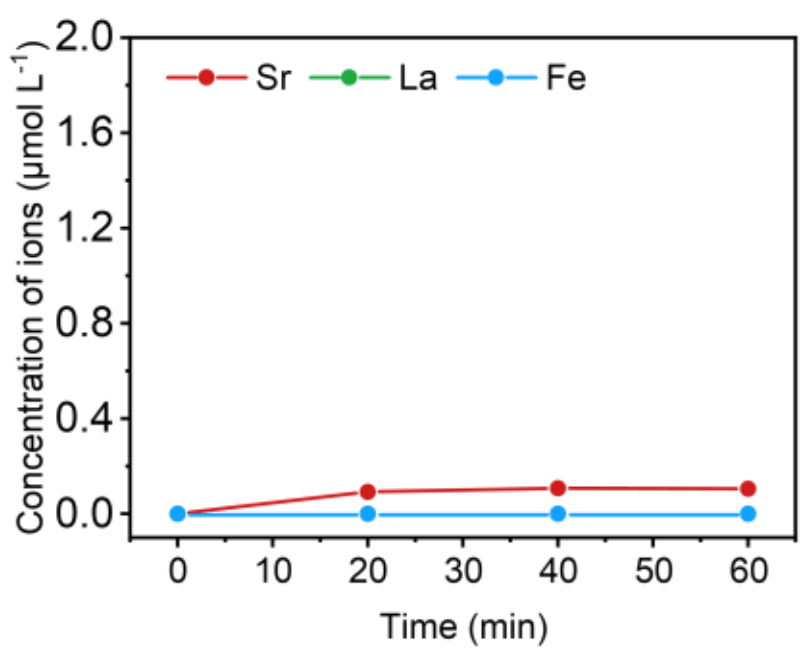

Figure R10 (also **Supplementary Fig. 56**). Dissolution concentrations of La, Sr and Fe elements from SNC-LSF in 0.1 M KOH with 8 mM KNO_3 at -0.4 V vs. RHE.

Related data (**Supplementary Fig. 56**) and discussions (yellow-highlighted in **Line 589-597, Page 23-24**) have been added in the revised manuscript as follows:

Line 589-597, Page 23-24: *"As shown in **Supplementary Fig. 56-60**, we further conducted ICP, XRD, AFM, XPS, and FTIR measurements to provide evidences to support the durability of the SNC-LSF electrode after stability tests. Notably, during the cathodic one-hour nitrate reduction electrolysis, the Sr concentration in the electrolyte increases only within the initial stage and becomes nearly constant after ~20 min. Compared to the 1000-s electrocatalyst activation in Figure 1d, Sr dissolution during nitrate reduction electrocatalysis decreases by an order of magnitude. indicating that the formation of SNC-LSF is essentially completed prior to catalysis and that the reconstructed structure remains stable thereafter."*

Comment 3: Based on this paper's interpretation of the reaction mechanism, La in the catalyst serves as the water cleavage site, while Fe acts as the NITRR site. However, in DFT calculations, both La and Fe are identified as $*NO_3$ adsorption sites. Please clarify the functions of each active site on the catalyst.

Response to C3: We thank the reviewer for this important question. Regarding the roles of La and Fe, we **calculated the interaction between the nitrate ion and the catalyst surface during nitrate adsorption**, as shown in **Figure R11**. After adsorption, Fe loses 0.177 electrons, while La loses 0.011 electrons. The electron interaction between nitrate and the catalyst mainly occurs between Fe and O. Therefore, the interaction between La and nitrate is primarily electrostatic, and Fe serves as the active site for nitrate reduction. Due to the alternating arrangement of Fe and La on the LSF surface, NO_3^- adsorbed on Fe is inevitably attracted by the adjacent La, which has a larger ionic radius and tends to form multi-coordination structures with O atoms, resulting in a bidentate coordination configuration. Moreover, since the concentration of nitrate in the solution is low, NO_3^- adsorbed on the surface does not significantly hinder the adsorption and dissociation of water on the La sites.

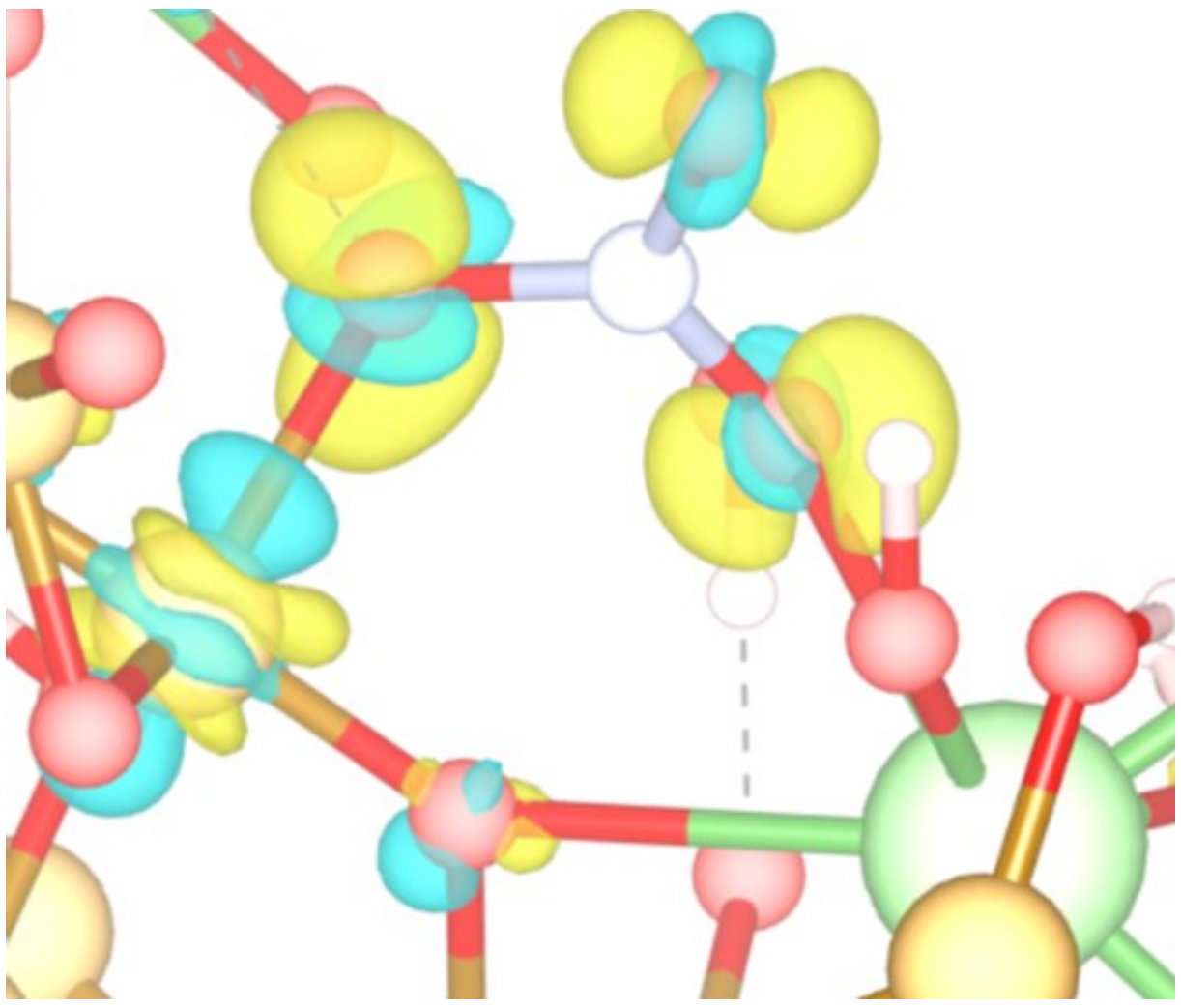

Figure R11. The charge density difference diagrams of $*NO_3$ absorbed on SNC-LSF.

Comment 4: The logical sequence of this manuscript is quite peculiar. I do not think the content of Section 2 does not belong the heading of Section 3: NITRR mechanism investigations. Furthermore, the manuscript places the NITRR performance of this catalyst in Section 4. A good electrocatalyst should first present its performance to the reader, rather than constantly explaining the mechanism. Please reorganize the logical flow of this study.

Response to C4: We thank the reviewer for this constructive suggestion. **As suggested by the reviewer**, we have revised the titles of Sections 2 and 3 into "**Interfacial nitrate adsorption and hydrogen transfer behaviors**" and "**Insights into the NITRR mechanism on SNC-LSF**" to better distinguish the content of them. In particular, the descriptions of Figures 2 and 3 have been **updated** to explicitly reflect their roles in elucidating the reaction mechanism, thereby improving the overall logical flow. We would also like to **emphasize that** the primary objective of this work is to uncover the mechanistic origin of the enhanced activity, specifically the nearly barrier-free hydrogen transfer enabled by intramolecular hydrogen bonding within hydroxyl-rich nanocavities. Therefore, the manuscript is intentionally structured with a mechanism-oriented emphasis. Meanwhile, we have **improved the transition from Section 3 to Section 4** of Electrocatalytic NITRR performance to better guide the reader.

Related discussions (yellow-highlighted in **Line 262, Page 10** and **Line 382, Page**

15, Line 503-506, Page 20) have been added in the revised manuscript as follows:

Line 262, Page 10: *"Nitrate adsorption and hydrogen transfer behaviors on SNC-LSF"*

Line 382, Page 15: *"Insights into the NITRR mechanism on SNC-LSF"*

Line 503-506, Page 20: *"The aforementioned experimental and theoretical results have fully validated the potential of the SNC-LSF electrode for selective ammonia production via nitrate reduction. Therefore, we systematically evaluated its performance toward NITRR, including activity, selectivity, and stability."*

Comment 5: This study highlights a barrierless hydrogen transfer mechanism. How can this phenomenon be demonstrated experimentally? What interface properties are associated with its occurrence?

Response to C5: We thank the reviewer for this insightful question. **In Section 2 of original manuscript**, **we have demonstrated** that adjacent hydroxyls over hydroxyl-rich nanocavity on SNC-LSF readily establish intramolecular hydrogen bonds, and the formed interfacial hydrogen bond networks are desired for favorable hydrogen transport during NITRR. Besides, in the revised manuscript, we further conducted **temperature-dependent EIS measurements to probe hydrogen transfer kinetics**. As shown in **Figure R12**, the activation energy (E_a) is as low as **0.22** eV, significantly below the ~0.4 eV threshold distinguishing Grotthuss-type hydrogen transport. This result **provides strong experimental evidence** for rapid hydrogen hopping along hydrogen-bond networks, consistent with the proposed mechanism.

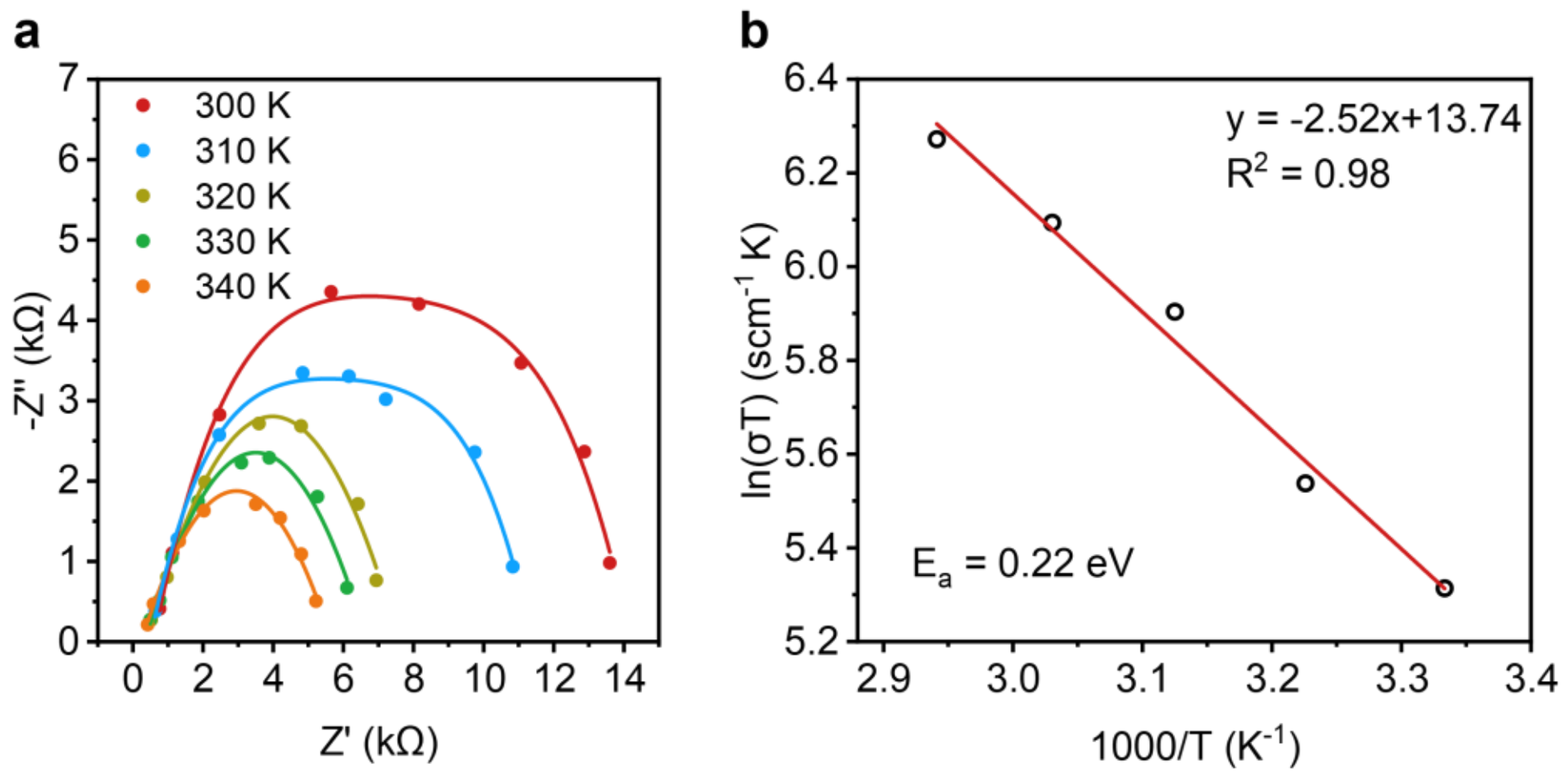

Figure R12 (also **Supplementary Fig. 27**). Temperature-dependent EIS measurements.

(a) Nyquist plots of SNC-LSF measured under 100% RH at different temperatures. (b) The fitted Arrhenius plots based on temperature-dependent EIS measurements on SNC-LSF.

Related data (**Supplementary Figure 27**) and discussions (yellow-highlighted in **Line 332-338, Page 13, Line 432-433, Page 17** and **Line 844-849, Page 33**) have been added in the revised manuscript as follows:

Line 332-338, Page 13: *"In addition, temperature-dependent EIS measurement was performed to investigate the hydrogen transfer capacity of the intramolecular hydrogen bonds on SNC-LSF (**Supplementary Fig. 27**). Fitted Arrhenius plots indicate that the hydrogen transfer activation energy (E_a) of 0.22 eV on SNC-LSF is below the empirical threshold of 0.4 eV, suggesting that hydrogen transport on SNC-LSF is predominantly governed by the Grotthuss-like mechanism[57]."*

Line 432-433, Page 17: *"in line with the experimentally observed low activation energy from temperature-dependent EIS measurements,"*

Line 844-849, Page 33: "***Temperature-dependent electrochemical impedance spectroscopy (EIS) measurements***

Temperature-dependent EIS measurements of SNC-LSF were conducted using a Solartron 1260 coupled with Solartron 1287 via the quasi-four-probe method. A 0.4 g sample was pressed into uniform pellets with a diameter of 12 mm and a thickness of 1-2 mm. The pellets were placed in an anhydrous chamber or under humidified conditions for 24 hours, after which conductivity measurements were performed under various conditions."

Comment 6: The in-situ Raman spectra in Fig. 2a show Raman peaks at different voltages are compressed too flat to discern any significant differences. The correlation between this and Fig. 2b is unconvincing.

Response to C6: We thank the reviewer for this helpful comment. The Raman spectra have been **reprocessed with optimized scaling and baseline correction**, allowing clearer visualization of potential-dependent changes and improved correlation with Fig.

2b. **As suggested by the reviewer**, the revised figure has been **updated** in the revised manuscript (Fig. 2a).

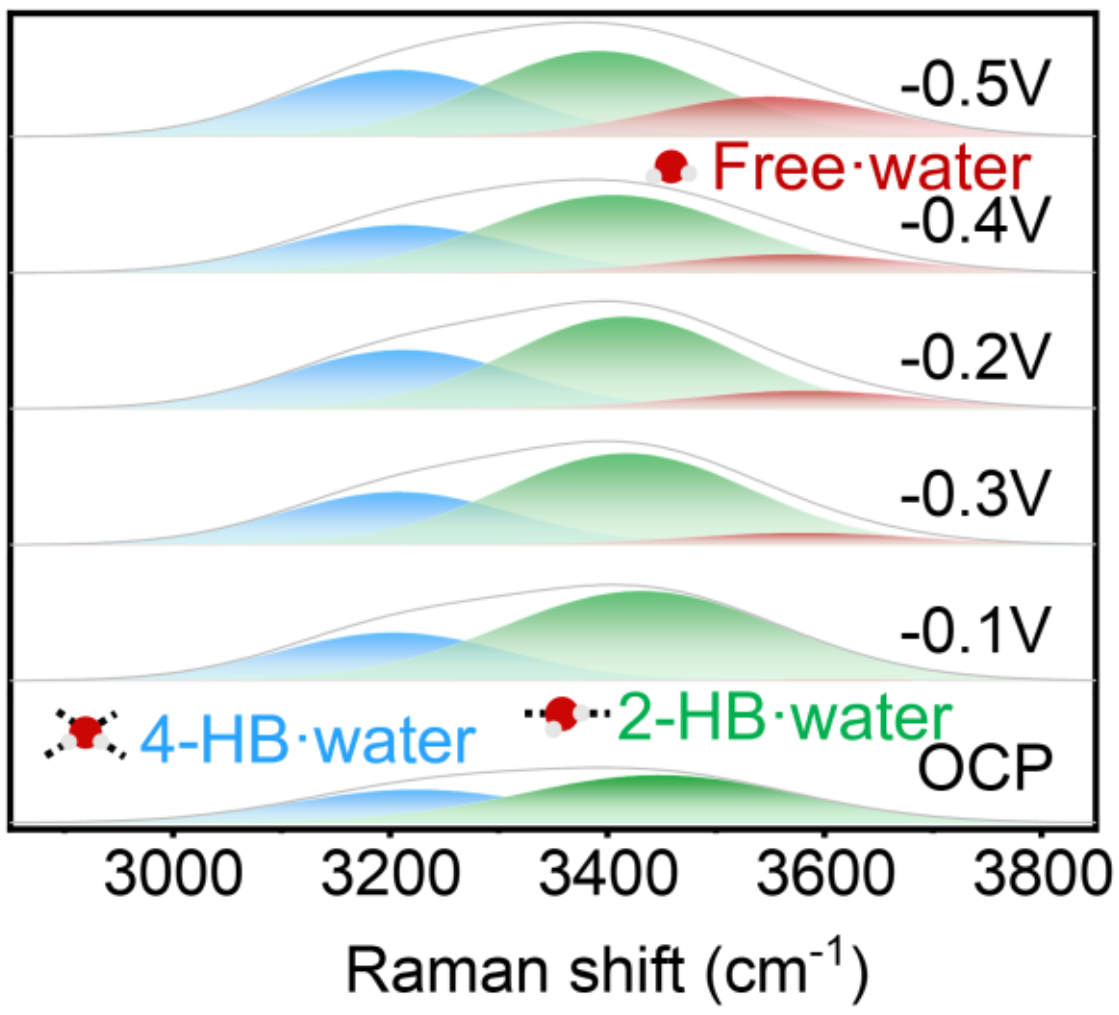

Figure R13 (also revised **Fig. 2a**). *In-situ* Raman spectra of SNC-LSF.

Comment 7: In Fig. 2h, the DEMS measurements show that pre-hydroxylation of SNC-LSF in H_2O versus D_2O leads to a nearly one order of magnitude difference in ammonia peak intensity. What causes this difference?

Response to C7: We thank the reviewer for this insightful question. The observed difference in ammonia signal intensity between H_2O and D_2O originates from the **kinetic isotope effect**. Specifically, the transfer of deuterium (D) is intrinsically slower than that of hydrogen due to its larger mass (*Angew. Chem. Int. Ed.* **64**, e202417631 (2025). *Nat. Commun.* **15**, 9499 (2024). *Nat. Commun.* **13**, 1189 (2022).), resulting in reduced proton transfer kinetics during the hydrogenation steps of NITRR. As a consequence, the rate of hydrogenation intermediates toward NH_3 formation is significantly suppressed in the D_2O system, leading to a markedly lower ammonia signal in DEMS measurements. This pronounced isotope-dependent behavior provides strong experimental evidence that hydrogen transfer is a rate-influencing step in the reaction pathway, further supporting the vital role of the proposed hydrogen hopping mechanism within the hydroxyl-rich nanocavity in the NITRR.

Comment 8: This study appears to have replaced metal sites to demonstrate the

universality of nanocaves in *H rapid transfer within NITRR. However, the data presentation and discussion for this section are not visible in the main text. This content could provide strong support for the innovative aspects of this research.

Response to C8: We thank the reviewer for highlighting the importance of clearly presenting the universality of the proposed nanocavity strategy. In response, **we have revised the presentation of the relevant data in Fig. 4c** by adjusting the layout and incorporating additional structural characterization results, which more clearly demonstrate the formation of nanocavity features across different compositions. Furthermore, **the corresponding discussion has been expanded in the main text** to explicitly emphasize the consistent enhancement in catalytic behavior and hydrogen transfer characteristics across these systems. These results provide stronger support for the general applicability of the nanocavity-confined hydrogen transfer mechanism and further highlight the broader significance of this design strategy.

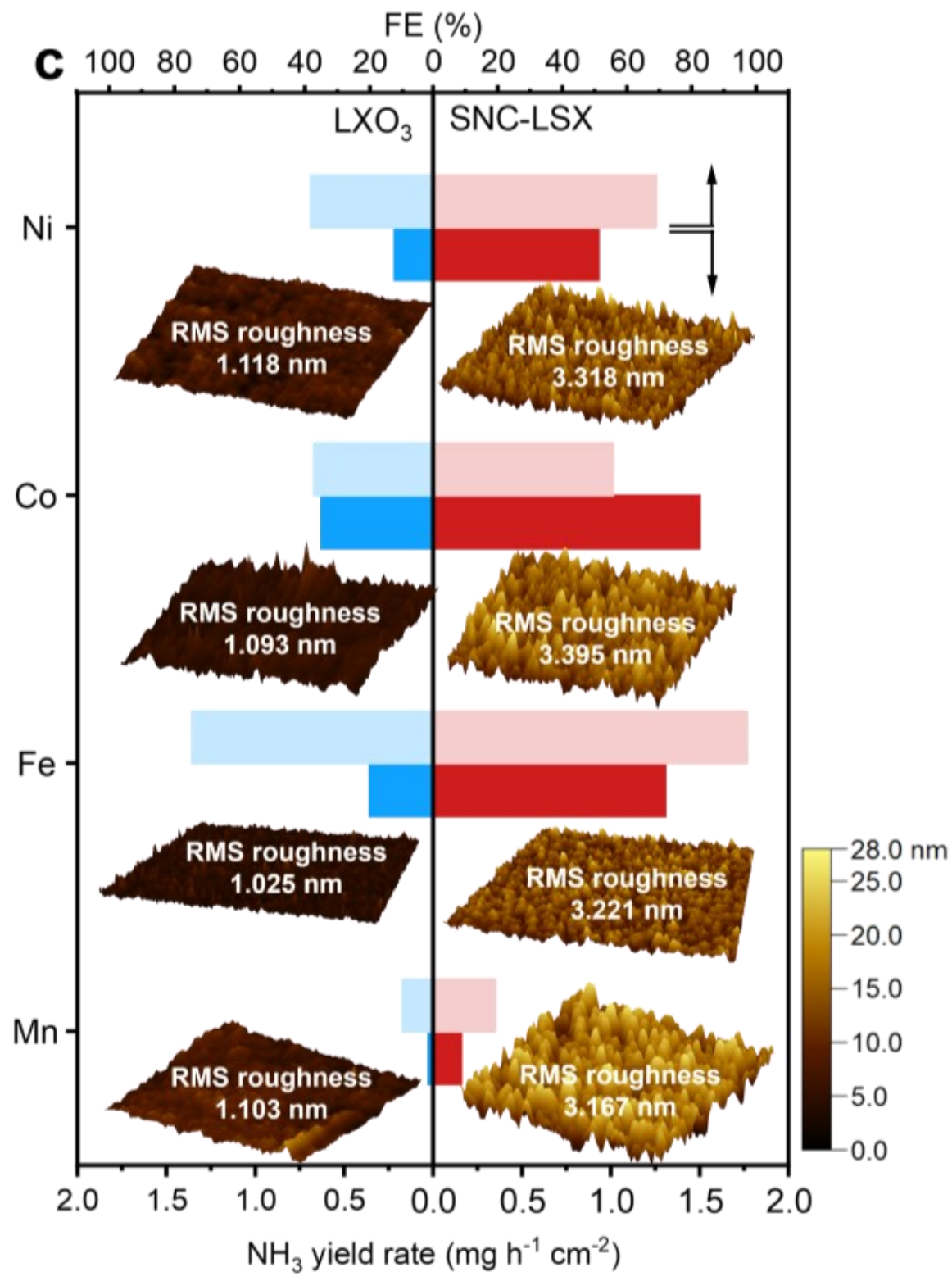

Figure R14 (also revised **Fig. 4c**). NH_3 yield rate and FE_{NH3} of LXO_3 and SNC-LSX (X= Mn, Fe, Co or Ni) in 0.1 M KOH with 8 mM KNO_3 at -0.4 V vs. RHE (inset are AFM maps and root mean square roughness of LXO_3 and SNC-LSX).

Related data **(Fig. 4c**) and discussions (yellow-highlighted in **Line 530-534, Page 21**) have been added in the revised manuscript as follows:

Line 530-534, Page 21: *"Notably, AFM analyses reveal that the surface roughness of all these samples increases significantly after Sr leaching, with RMS roughness increasing from 1.025-1.118 nm for the pristine samples to 3.167-3.395 nm after activation, consistent with the formation of nanocavity features."*

Reviewer #3

GENERAL COMMENTS: The authors developed a surface nano-concaved $La_{0.4}Sr_{0.6}FeO_{3-\delta}$ (SNC-LSF) catalyst via electrochemical reduction-assisted selective Sr^{2+} leaching. The formed nanocavities possess hydroxyl-enriched walls that establish an intramolecular hydrogen-bond network, enabling a nearly barrierless hydrogen transfer pathway. This leads to exceptional nitrate-to-ammonia conversion performance, achieving a Faradaic efficiency of 97.81% and an NH_3 yield rate of 51.37 mg h^{-1} cm^{-2} at -0.8 V vs. RHE. This work explores a timely issue with practical importance, and its results is of interest to Nature Communications readers. However, several key aspects require further clarification and strengthening to fully substantiate the claims and contextualize the significance of the findings within the existing literature.

Response: We are pleased that Reviewer #3 **recognized both the significance and timeliness of this work**. The reviewer noted that "*this work explores a timely issue with practical importance, and its results is of interest to Nature Communications readers,*" **highlighting the broader impact of the study**. **The mechanistic innovation was also appreciated**, as it was recognized that the hydroxyl-enriched nanocavities enable "*a nearly barrierless hydrogen transfer pathway*" through an intramolecular hydrogen-bond network. In addition, **the reviewer acknowledged the excellent catalytic performance**, noting the "*exceptional nitrate-to-ammonia conversion performance*" achieved in this system. At the same time, the reviewer raised several points requiring further clarification and strengthening. In response, additional analyses, expanded

discussions, and clarifications have been incorporated into the revised manuscript to further substantiate the conclusions and better position this work within the existing literature.

Comment 1: The presentation of Figure 1d is currently not intuitive, which could lead to reader confusion or misinterpretation. To address this, it is recommended to revise the figure into a bar chart for a clearer and more intuitive representation of the data.

Response to C1: We thank the reviewer for this helpful suggestion. **According to reviewer's recommendation**, **Fig. 1d has been revised** into a bar chart to provide a more intuitive and clear comparison of the data. This modification improves readability and reduces the possibility of misinterpretation.

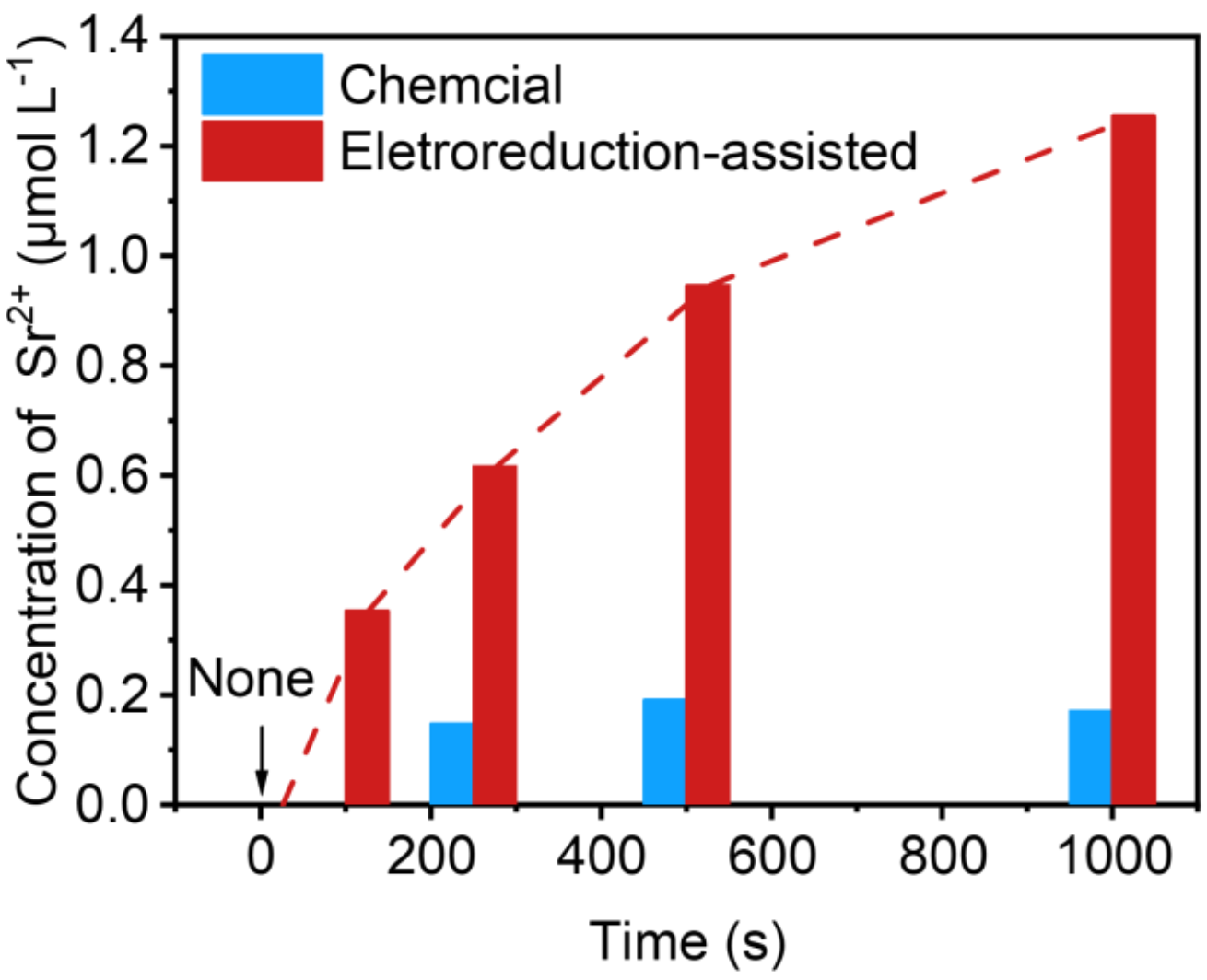

Figure R15 (also revised **Fig. 1d**). Dissolution concentrations of Sr elements from LSF in 0.1 M KOH.

Comment 2: The author mentioned the following content. Is there any corresponding supporting literature or theoretical data to support this statement? "The slight differences in NITRR activity among SNC- LCF, SNC-LSF, and SNC-LBF may be attributed to variations in the leaching rates and ionic radii of Ca^{2+}, Sr^{2+} and Ba^{2+}."

Response to C2: We thank the reviewer for this constructive comment. In the revised manuscript, we **have added relevant references** (*i.e.*, **ref. 38** and **39**) to support the

statement (*ACS Nano* **19**, 39480-39490 (2025). *Sci. Adv.* **5**, eaav6262 (2019). *Energy Environ. Sci.* **13**, 4178-4188 (2020). *Chem* **5**, 3243-3259 (2019). *Adv. Energy Mater.* **13**, 2204417 (2023)). Previous studies have shown that the ionic radii and bonding strength of A-site cations (Ca^{2+}, Sr^{2+}, and Ba^{2+}) significantly influence their leaching behavior and the extent of surface reconstruction under electrochemical conditions. Larger ionic radii and weaker metal-oxygen bonding generally facilitate faster leaching and more pronounced structural reconstruction. These differences in leaching kinetics and structural evolution can, in turn, affect the formation of active sites and local reaction environments, thereby leading to variations in NITRR performance among SNC-LCF, SNC-LSF, and SNC-LBF.

Comment 3: The authors mention that SNC-LSF can provide abundant active H* species. Given such a high concentration of H*, why does the hydrogen evolution reaction (HER) not occur preferentially, while the more complex nitrate reduction reaction is instead favored?

Response to C3: We thank the reviewer for this important question. Although abundant active hydrogen species (H*) can be generated on SNC-LSF, their utilization pathway is **fundamentally different from** that in conventional HER-dominated systems. Specifically, the hydroxyl-rich nanocavities establish an intramolecular hydrogen-bond network that enables directional and confined hydrogen transfer toward adsorbed nitrate intermediates. This unique environment effectively channels H* into the multi-step hydrogenation of NO_3^- species, thereby suppressing their recombination into H_2. At the same time, this behavior is further supported by theoretical calculations, which show that the adsorption energies of hydrogen at representative surface sites (0.322, -0.593, -0.854, -0.387, and 1.014 eV; **Figure R16**) deviate significantly from the near-thermoneutral value ($\Delta G_{H^*} \approx 0$ eV) required for efficient HER catalysts, indicating intrinsically unfavorable HER kinetics. In particular, sites with moderately negative adsorption energies can stabilize H* for participation in nitrate reduction, whereas sites with positive adsorption energies are not conducive to H* adsorption. Therefore, **the combined effects of confined hydrogen transfer within nanocavities and non-**

optimal H* adsorption energies ensure that H* is preferentially consumed in NITRR rather than HER, resulting in the observed high selectivity toward ammonia.

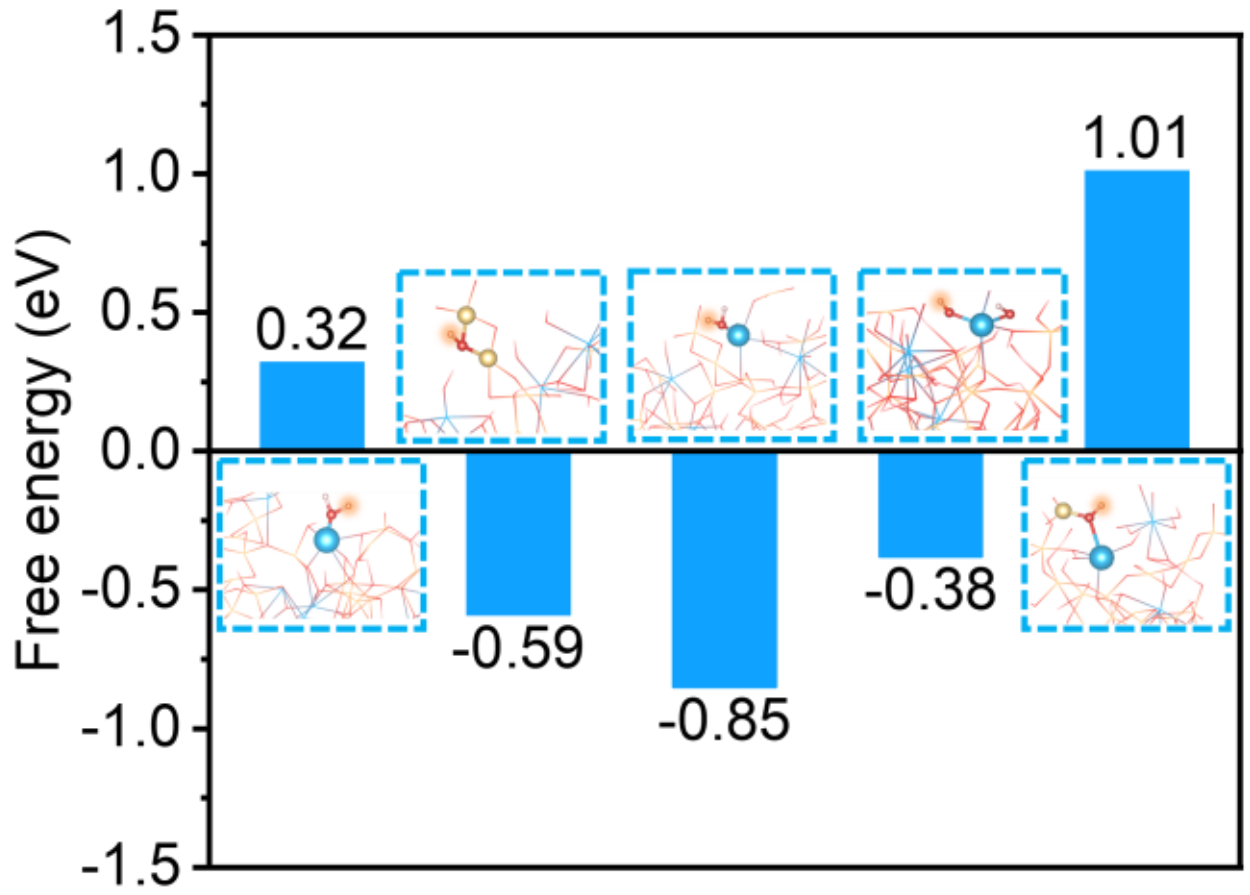

Figure R16 (also **Supplementary Figure 35**). Gibbs free energy diagram for hydrogen adsorption at different catalytic sites on SNC-LSF.

Related data **(Supplementary Figure 35**) and discussions (yellow-highlighted in **Line 415-419, Page 16**) have been added in the revised manuscript as follows:

Line 415-419, Page 16: *"This preference originates from the confined hydrogen-bond network within the hydroxyl-rich nanocavities, which enables directional hydrogen transfer toward nitrate intermediates, together with the non-ideal hydrogen adsorption energies that deviate from thermoneutral conditions ($\Delta G_{H*} \approx 0$ eV) for HER, thereby disfavoring H_2 evolution (**Supplementary Fig. 35**)."*

Comment 4: Is there a competitive adsorption relationship between active H* and $*NO_3$?

Response to C4: We appreciate the reviewer's insightful question. As shown in **Figure R17**, it is observed in our calculations that the hydrogen adsorption sites including non-metal O and OH sites are distinct from those metal sites of nitrate adsorption, which implies that **no competitive adsorption** between H* and $*NO_3$ occurs.

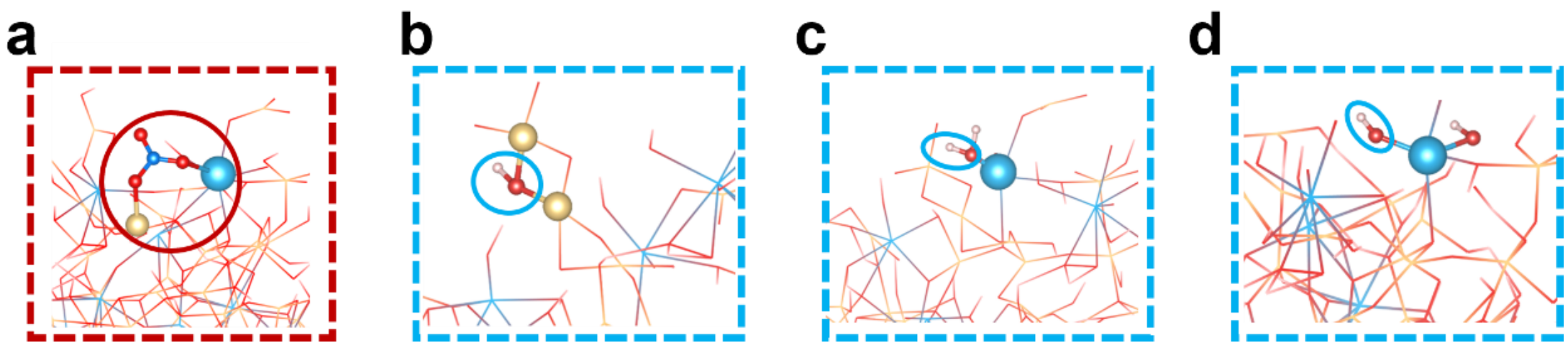

Figure R17. (a) *NO_3 adsorption site and (b-d) H* adsorption sites on SNC-LSF.

Comment 5: Why does potassium thiocyanate (KSCN) selectively poison only the Fe sites in the catalyst without interacting with other metals? Is this claim supported by relevant literature?

Response to C5: We thank the reviewer for this valuable comment. **KSCN is widely used as a selective poisoning agent for transition metal sites** due to the strong coordination interaction between SCN^- and metal centers, particularly Fe sites (*Nat. Sustain.* **8**, 1513-1523 (2025). *Angew. Chem. Int. Ed.* **57**, 11445 (2018). *Angew. Chem. Int. Ed.* **63**, e202410123 (2024)). The preferential adsorption of SCN^- on Fe can effectively block the active sites involved in nitrate adsorption and reduction. We have added relevant literature references (*i.e.*, **ref. 64**) to support this point in the revised manuscript (These studies demonstrate that SCN^- can selectively coordinate with transition metal centers and inhibit their catalytic activity, thereby serving as an effective probe for identifying active sites.

Comment 6: The authors are advised to provide a thorough and well-reasoned explanation for the computational results presented in the article to avoid any potential misinterpretation by readers. "We found that only the *+ NO_3^- → *NO_2 step was an endothermic process with a quite low energy barrier of just 0.10 eV. At the same time, *NH_3 desorption becomes energetically barrierless (0 eV), thereby facilitating product release and regeneration of active sites."

Response to C6: We thank the reviewer for this important suggestion. In the revised manuscript, we have provided a more detailed and rigorous explanation of the computational results.

Related discussions (yellow-highlighted in **Line 486-492, Page 19-20**) have been added in the revised manuscript as follows:

Line 486-492, Page 19-20: *"The calculated free energy profile indicates that the initial step (*+ NO_3^- → $^{*}NO_2$) is the only mildly endothermic process, with a low energy barrier of approximately 0.10 eV, suggesting that nitrate adsorption and activation is favorable. Subsequent hydrogenation steps are energetically downhill, reflecting the facile hydrogenation of reaction intermediates within the hydroxyl-rich nanocavity. Notably, the final $^{*}NH_3$ desorption step is predicted to be thermodynamically barrierless (0 eV), which facilitates efficient product release and rapid regeneration of active sites."*

Comment 7: There is an error in the labeling of Figure S61 that should be corrected.

Response to C7: We thank the reviewer for pointing out this error. As suggested by the reviewer, the labeling mistake in **Figure S67** has been corrected in the revised manuscript.

Point-to-Point Responses to Reviewers' Comments and Suggestions

First of all, we thank the reviewers for their valuable comments and suggestions, which are very helpful to improve the quality and clarity of this paper. To address the specific concern/point clearly, we have separated the referees' comments into question areas and answered them in turn as follows.

Reviewer #1

GENERAL COMMENTS: I thoroughly reviewed the revised manuscript. The authors diligently conducted the additional experiments requested by the reviewers and clearly addressed their concerns. In sum, I recommend that the revised manuscript be accepted for publication as is.

Response: We thank the reviewer for this constructive review process and recommendation for publication of our work.

Reviewer #2

GENERAL COMMENTS: The authors have addressed most comments from other reviewers regarding material characterization, structural stability, logical organization, and figure presentation. However, the following critical scientific and methodological concerns still remain fully unaddressed and must be resolved to ensure the reliability and validity of the core conclusions.

Response: We sincerely thank the reviewer for **acknowledging that we have adequately addressed the concerns raised by the other two reviewers regarding material characterization, structural stability, logical organization, and figure presentation**. We are pleased that these aspects have now been resolved to the reviewer's satisfaction.

Regarding the remaining critical scientific and methodological concerns pointed out by the reviewer, we greatly appreciate the constructive feedback. In the following,

we provide a point-by-point response to each of these specific issues, along with detailed explanations and additional data where necessary. We believe that with these revisions, the core conclusions of our work are further strengthened and fully substantiated.

Comment 1: The isotope-labeling DEMS results still cannot distinguish whether the deuterated ammonia signals (m/z = 17, 18, 19, 20) arise from lattice Fe–OD/La–OD hydroxyl groups or merely *physically adsorbed D species on the catalyst surface. No control experiment or theoretical justification is provided to identify the genuine source of deuterons, leaving this key mechanistic evidence unsubstantiated.

Response to C1: We thank the reviewer for this critical comment regarding the origin of deuterium species in the isotope-labeling DEMS experiments. First, we would like to clarify that physically adsorbed species typically correspond to weak, reversible interactions that can be readily removed by standard cleaning procedures and do not exhibit stable participation in electrochemical reactions. In our experiments, the catalyst electrodes were **thoroughly rinsed with ultrapure water and subjected to repeated sonication prior to measurements to eliminate any physically adsorbed species**. These procedures are widely adopted to remove weakly bound adsorbates (*Angew. Chem. Int. Ed.* **2026**, e7288776. *J. Am. Chem. Soc.* **147**, 14869-14877 (2025). *Nat. Commun.* **15**, 10289 (2024). *Nat. Commun.* **15**, 9499 (2024). *Angew. Chem. Int. Ed.* **64**, e18519 (2025).). To further ensure the reliability of the results, we **have now added a detailed description of the cleaning protocol** in the **revised Experimental Section (Line 874-879, Page 34)**. Under such conditions, residual deuterium species originating from simple physical adsorption are negligible. More importantly, the observed deuterated ammonia signals are only generated under electrochemical reduction, which is inconsistent with the behavior of physically adsorbed species. Instead, these results are more reasonably attributed to the participation of chemically bound hydroxyl species (e.g., Fe–OD/La–OD) involved in interfacial hydrogen transfer. Therefore, we conclude that the detected deuterium signals originate from structurally associated hydroxyl species rather than physically adsorbed D species.

Related discussions (yellow-highlighted in **Line 874-879, Page 34**) have been added in the revised manuscript as follows:

Line 874-879, Page 34: *"Prior to isotope-labeling DEMS measurements, the catalyst-coated electrodes were thoroughly cleaned to remove any physically adsorbed species. Specifically, the electrodes were rinsed with ultrapure water and subjected to repeated sonication, followed by drying under an inert atmosphere. This procedure ensures the effective removal of weakly bound adsorbates, minimizing interference from physically adsorbed deuterium species during subsequent measurements."*

Comment 2: The proposed hydrogen tunneling effect and Grotthuss-like hydrogen transfer mechanism still lack rigorous and direct evidence. Although the authors supplemented hydrogen transfer activation energy data, they provided no experimental or computational verification for hydrogen tunneling, and the Grotthuss mechanism is not convincingly validated.

Response to C2: We thank the reviewer for this important comment. We fully agree with the reviewer that the term "hydrogen tunneling" is not rigorously supported in the current context. In the revised manuscript, we have removed all related descriptions to avoid potential overinterpretation. Instead, we focus on the experimentally and theoretically supported hydrogen-bond-network-mediated hydrogen transfer mechanism. Specifically, temperature-dependent EIS measurements yield an activation energy of 0.22 eV, which is significantly lower than the commonly accepted threshold (~0.4 eV) for Grotthuss-type hydrogen transport, as widely reported in the literature (*Nat. Energy.* **4**, 123-130 (2019). *Chem. Soc. Rev.* **46**, 464-480 (2017). *Angew. Chem. Int. Ed.* **57**, 5345 (2018). *Angew. Chem. Int. Ed.* **60**,21838-21845 (2021). *Sci. Adv.* **11**, eadu1602 (2025)). In addition, AIMD simulations reveal a continuous hydrogen-bond network that facilitates rapid hydrogen hopping with an activation energy of 0.10 eV, further supporting a Grotthuss-like mechanism. Taken together, **both experimental and computational results consistently support that hydrogen transfer in this system proceeds via a hydrogen-bond-mediated Grotthuss-type mechanism**.

Related discussions (yellow-highlighted in **Line 354-359, Page 14**) have been

added in the revised manuscript as follows:

Line 354-359, Page 14: *"Such interesting phenomenon affirms the hydrogen migration along the intramolecular hydrogen-bond network on SNC-LSF, where free* H_2O *dissociates to generate* $^*H$ *and form H-O on the lattice oxygen of surface hydroxyls as well as hydrogen existed initially in surface hydroxyls dissociates and transfers stepwise along the hydrogen-bonded network to the* NO_3^-*-adsorbed active sites for* NO_3^- *hydrogenation."*

Comment 3: The H/D kinetic isotope effect (KIE) analysis remains seriously flawed with no correction. The authors neither excluded the interference caused by different cations (K^+ vs. Na^+) nor separated KIE contributions from nitrate reduction and the competing hydrogen evolution reaction (HER). Using the total KIE to support the intramolecular hydrogen-bond network is scientifically invalid.

Response to C3: We thank the reviewer for this important and insightful comment regarding the validity of the KIE analysis. We agree that rigorous control of experimental variables and careful interpretation are essential for drawing reliable mechanistic conclusions. **As suggested by the reviewer**, control experiments were conducted in **consistent electrolyte systems** (*i.e.*, KNO_3+KOH/KOD) to eliminate cation-related interference in the revised manuscript. More importantly, **the KIE analysis was re-evaluated based on product-specific metrics (NH_3 yield rate and Faradaic efficiency)**, thereby excluding contributions from competing HER and enabling a more direct assessment of the nitrate reduction pathway.

As shown in **Figure R1**, a clear isotope effect on both activity and selectivity was observed. Specifically, when switching to deutero conditions, the NH_3 yield rate and Faradaic efficiency decrease, while NO_2^- production and its corresponding Faradaic efficiency increase. Furthermore, when using pre-deuterated catalysts in deutero electrolyte (SNC-LSF(D)), this trend became more pronounced, with a further suppression of NH_3 formation and enhanced accumulation of NO_2^-. This behavior is fully consistent with prior KIE studies (*J. Am. Chem. Soc.* **147**, 14316-14325(2025)), where slower deuteron transfer impedes the multi-step proton-coupled reduction

pathway toward NH_3, resulting in a shift toward the partial $2e^-/2H^+$ reduction product (NO_2^-). These observations indicate that proton transfer plays a critical role in the rate-determining steps of the NITRR, particularly in the downstream hydrogenation processes from NO_2^- to NH_3. The isotope-dependent suppression of NH_3 formation, together with the accumulation of NO_2^-, supports that hydrogen transfer kinetics directly govern the reaction pathway and product distribution.

Accordingly, the revised KIE results provide consistent evidence that hydrogen transfer is intimately involved in the NITRR mechanism and supports the proposed hydrogen-bond-mediated transfer pathway.

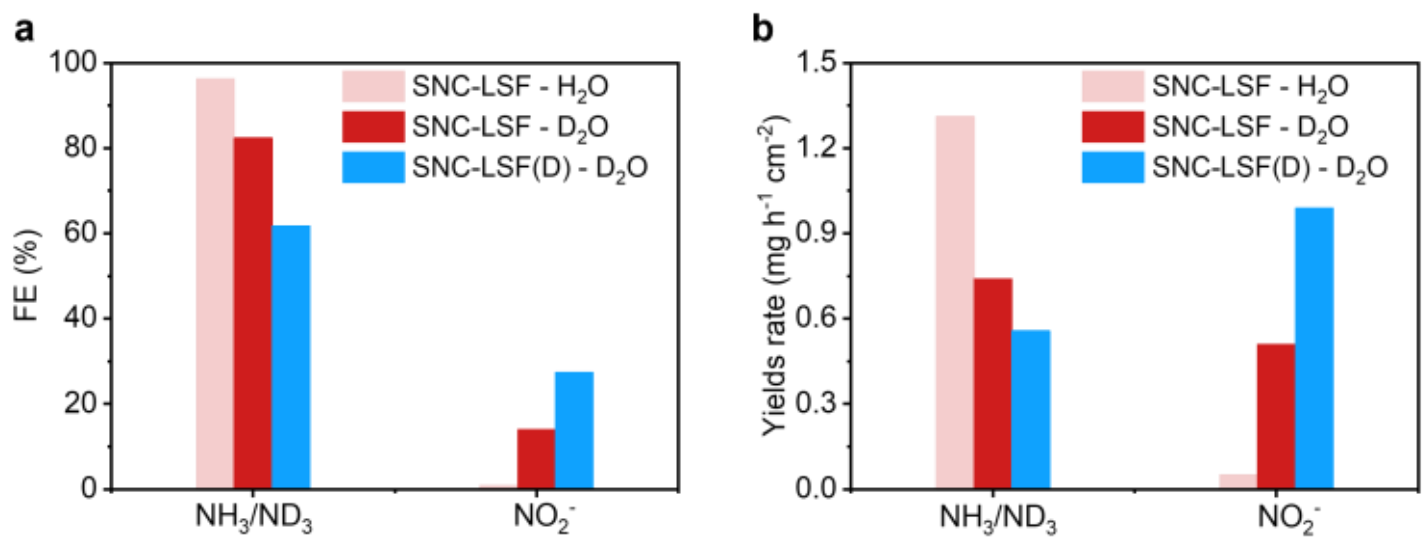

Figure R1 (also **Supplementary Figure 30**). **Kinetic isotope effect.** NITRR performance of SNC-LSF and SNC-LSF(D) recorded in both H_2O and D_2O electrolytes with the addition of 8 mM KNO_3 containing 0.1 M KOH or KOD. (a) Faradic efficiency (b) yields rate of NH_3/ND_3 and NO_2^- of SNC-LSF and SNC-LSF(D) at -0.4 V vs. RHE.

Related data **(Supplementary Fig. 30**) and discussions (yellow-highlighted in **Line 362-368, Page 14-15**) have been added in the revised manuscript as follows:

Line 362-367, **Page 14-15**: *"Additionally, the H/D kinetic isotope effect (KIE) was re-evaluated using NH_3 yield rate and Faradaic efficiency. Upon deuteration, the NH_3 yield and Faradaic efficiency decrease while NO_2^- production increase, with this trend becoming more pronounced for pre-deuterated SNC-LSF(D). These results indicate that hydrogen transfer is involved in the rate-determining steps and support a hydrogen-bond-mediated hydrogen transfer pathway during NITRR."*

Comment 4: The techno-economic analysis (TEA) still relies on unrealistic assumptions without modification: the cost calculation ignores wastewater pretreatment

costs, the referenced Haber–Bosch ammonia price (\$1.15 kg^{-1}) is unreasonably high, and the assumed electricity price (\$0.02 kWh^{-1}) is inconsistent with actual market levels, making the claimed economic advantage unconvincing.

Response to C4: We thank the reviewer for this important and constructive comment. As is common in most techno-economic analyses of ammonia synthesis, our TEA is intended as **a simplified, process-level, and trend-oriented analysis**, with the main purpose of identifying the dominant cost drivers of the electrochemical conversion and NH_3 recovery steps, and of illustrating the possible economic feasibility range of this route, **rather than** providing a single definitive plant-wide cost for all real wastewater-treatment cases.

First, regarding the reviewer's concern that the cost calculation ignores wastewater pretreatment costs, we would like to clarify further that, in nitrate-remediation-related systems, **water-treatment economics are usually not represented by a single and standalone "pretreatment cost" item.** This is because real nitrate-containing water sources may vary substantially in nitrate concentration, impurity composition, salinity level, discharge requirements, and specific treatment train. As a result, TEA studies related to nitrate removal generally adopt a system-level distributed cost-accounting framework to evaluate treatment-related economics. In other words, these costs are often distributed across different categories, including annualized capital cost, operating cost, maintenance cost, concentration or separation cost, as well as waste-treatment or disposal cost, rather than being simplified into one unified and independent pretreatment cost item. Following this widely adopted treatment logic, in the present work, we also **adopted an approach consistent with the relevant literature** (*Nat. Commun.* **16**, 5581 (2025). *Nat. Chem. Eng.* **2**, 379–390 (2025). *Energy Environ. Sci.* **17**, 5691–5705 (2024)) and incorporated the economics associated with wastewater pretreatment into both Capital Cost and Operating Cost, as shown in **Table S6**. Therefore, our intention **was not** to imply that water-treatment-related costs do not exist, **but rather** to evaluate the electrochemical conversion stage and NH_3 recovery stage under a defined process boundary, while recognizing that upstream water-treatment complexity cannot be reduced to a single universally applicable pretreatment term.

Second, regarding the concern that the referenced Haber-Bosch ammonia price of US$1.15 kg^{-1} is unreasonably high, we respectfully note that this value was not arbitrarily selected by us, but **adopted from published literature as an external benchmark**. More importantly, a recent *Nature Catalysis* study (*Nat. Catal.* **7**, 1032–1043 (2024)) in the same nitrate-to-ammonia field also explicitly **used the same benchmark value of US$1.15** per kilogram NH_3 as the current NH_3 market price from the Haber-Bosch process, and cited the same *Farmdoc Daily* source by Schnitkey et al. [Farmdoc Daily https://farmdocdaily.illinois.edu/2023/02/fertilizer-prices-and-company-profits-going-into-spring-2023.] as its literature basis. This indicates that our use of this benchmark **is consistent with recent precedent in the NO_3RR literature rather than being an unrealistic number introduced without support.**

Since ammonia is still predominantly produced via the Haber-Bosch process, its market price largely reflects the economics of Haber-Bosch production. At the same time, this price is not fixed, but continuously fluctuates with time and market conditions. It can be affected by multiple factors, including energy price, feedstock cost, regional variation, supply-demand relationship, and accounting boundary. For this reason, the purpose of our TEA is not to draw an absolute conclusion from one single price point, but to evaluate the cost response under different operating and economic scenarios through sensitivity analysis and contour-map analysis. As shown by our results (**Figure R2**), under different combinations of electricity price, current density, and cell voltage, the resulting NH_3 production cost can be either higher or lower than the literature benchmark, which is itself consistent with the inherently fluctuating nature of ammonia market prices. Therefore, our point is not to treat US$1.15 kg^{-1} as a uniquely fixed value, but to show that, under reasonable operating conditions and parameter combinations, this route may have the potential to approach or even fall below the literature-based benchmark price. The *Nature Catalysis* study mentioned above followed the same logic by using US$1.15 kg^{-1} as a literature benchmark while also presenting sensitivity analysis and contour plots to show how the cost varies with key parameters.

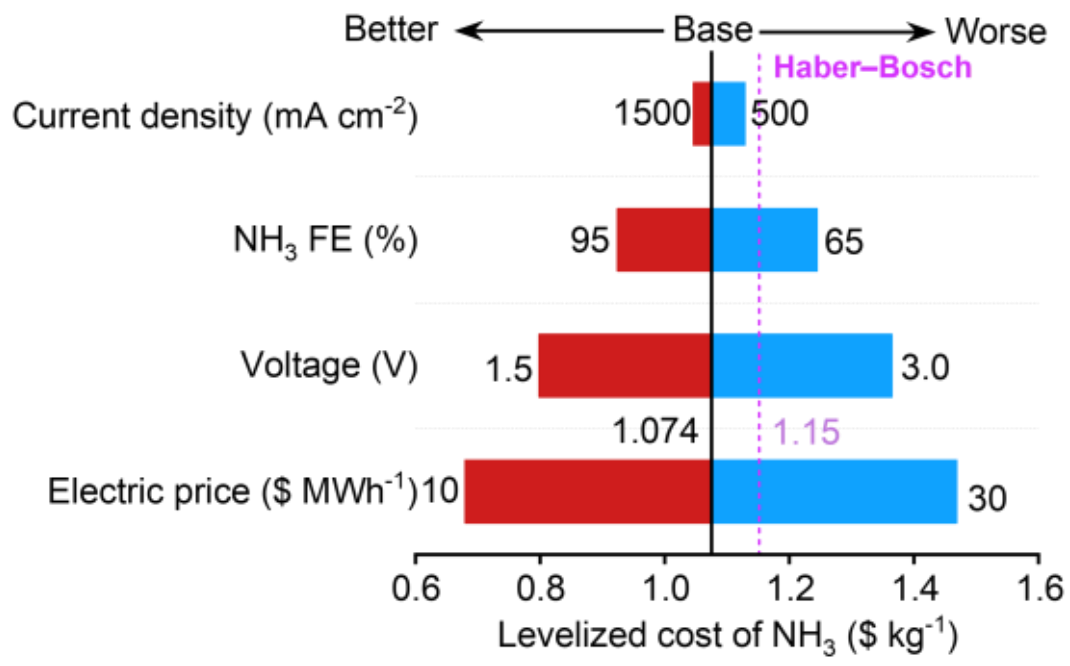

Figure R2 (also **Figure 5e)**. Sensitivity analysis of levelized NH_3 cost for operational and economic parameters.

Third, regarding the reviewer's concern that the assumed electricity price of US$0.02 kWh^{-1} is inconsistent with actual market levels, we would like to **clarify further that this value was used as a scenario assumption to evaluate the possible economics under favorable renewable-electricity conditions, rather than** being treated as a universally applicable fixed market price. The purpose of introducing this value was to examine cost sensitivity to key parameters, reveal cost trends, and evaluate the possible competitiveness range of this route under different operating conditions, rather than to define one single and fixed real-world outcome based on a single price point. **This type of treatment is common in recent electrochemical TEA studies as well**. For example, in the techno-economic analysis of n-propanol synthesis (*Nat. Catal.* **8**, 239-247(2025)), the authors explicitly used US$0.02 kWh^{-1} as the electricity price assumption for a renewable wind energy scenario and further conducted sensitivity analysis over a range of US$0.01 to 0.03 kWh^{-1}.

More importantly, our manuscript **does not rely on a single-point electricity-price assumption**. As shown in **Table S5** and **Figure R3**, we have already provided sensitivity analysis and contour-map analysis under different electricity prices as well as different performance conditions, including current density and cell voltage. In particular, **Table S5** sets the electricity price at US$10, 20, and 30 per MWh, corresponding to US$0.01, 0.02, and 0.03 kWh^{-1}, showing that the present study itself already considers the cost variation over different electricity-price conditions. Moreover, the future price of green electricity is inherently uncertain and difficult to constrain to any single fixed value in advance. Therefore, **the central message of this**

TEA is not that the system must correspond to one fixed production cost in real-world practice, but rather that the cost is highly sensitive to electrochemical performance and electricity price. The main purpose of the model is to identify the dominant cost drivers, reveal cost trends, and illustrate the possible competitiveness range.

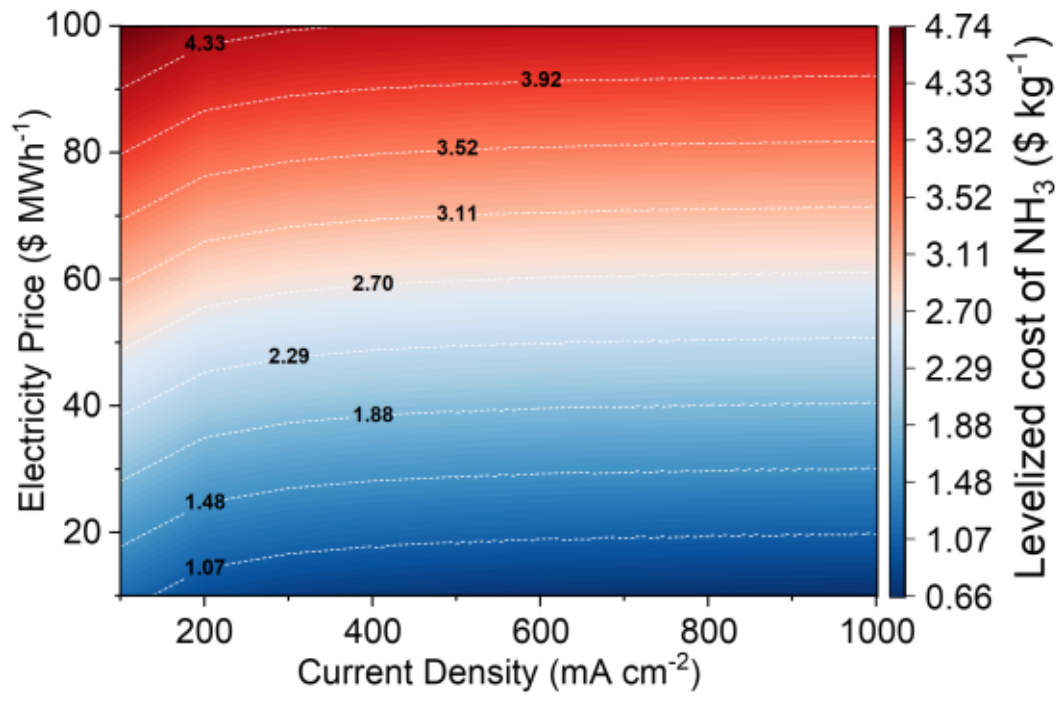

Figure R3 (also **Supplementary Figure 71).** Techno-economic landscape of NH_3 production cost as a function of electricity price and current density. Contour plot illustrating the dependence of levelized NH_3 production cost on electricity price and current density, highlighting optimal operational conditions under varying energy input scenarios.

Overall, we want to emphasize that the TEA presented in this work is not intended to provide one universally fixed cost conclusion for all real scenarios. Rather, under clearly defined parameter assumptions, it serves as a process-level evaluation to identify the dominant cost drivers, reveal cost trends, and illustrate the possible competitiveness range of this electrochemical nitrate-to-ammonia route.

Comment 5: The significant XRD peak shift with Sr doping (Supplementary Figure 2) still lacks explanation regarding lattice distortion or phase evolution.

Response to C5: We thank the reviewer for this insightful comment. We would like to clarify that Figure S2 corresponds to the pristine samples. The observed XRD peak shift with increasing Sr content can be understood from **the combined effects of A-site substitution and charge compensation**. Considering that the ionic radius of Sr^{2+} (1.44 Å) is larger than that of La^{3+} (1.36 Å), such a lattice reduction likely comes from the oxidation of Fe^{3+} (0.645 Å) to Fe^{4+} (0.585 Å) due to charge compensation when Sr^{2+} substitutes for La^{3+}. Similar behavior has been reported in perovskite systems with

aliovalent substitution (*ACS Appl. Mater. Interfaces* **10**, 11715-11721 (2018), *Phys. Rev. B* **107**, 024104 (2023). *J. Am. Chem. Soc.* **147**, 30401−30411(2025), *J. Am. Chem. Soc.* **143**, 2741−2750(2021). *Appl. Catal. B* **339**, 123456 (2024).). We have added this explanation in the revised manuscript to clarify the origin of the peak shift.

Related discussions (yellow-highlighted in **Line 2-5, Page 3**) have been added in **the caption of Supplementary Figure 2** in revised supplementary information as follows:

Line 2-5, Page 3: *"The positions of the characteristic diffraction peaks shift systematically toward higher angles with increasing strontium content (x), indicating a reduction in lattice size, this may originate from the B-site charge compensation phenomenon upon substitution of* La^{3+} *by* Sr^{2+}*."*

Comment 6: Basic XRD data for LCF, LBF, LSC, LSN, and LSM are still not provided to support the generality of hydroxyl-rich nanocavity formation.

Response to C6: We thank the reviewer for these helpful comments. As suggested by the reviewer, to support the generality of the proposed nanocavity formation strategy, , the XRD patterns of LCF, LBF, LSC, LSN, and LSM **have now been included** in the revised Supporting Information (**Figures R4** and **R5**), covering both pristine and post-activation states. Notably, the XRD peak intensities of SNC-LCF, SNC-LBF, and SNC-LSX (X = Co, Ni, Mn) are **consistently lower** than those of their corresponding pristine counterparts (LCF, LBF, and LSX), indicating a reduced crystallinity and the formation of partially amorphous surface structures after activation.

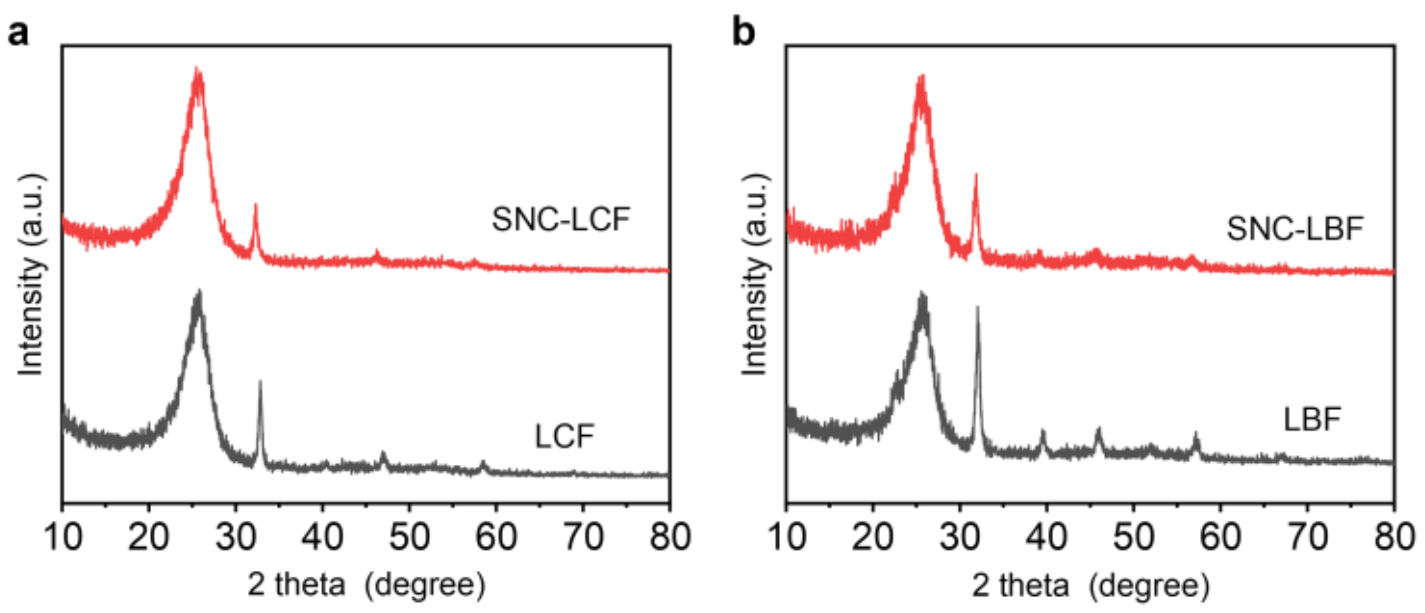

Figure R4 (also **Supplementary Figure 18)**. XRD patterns of (a) LCF and SNC-LCF, (b) LBF and SNC-LBF.

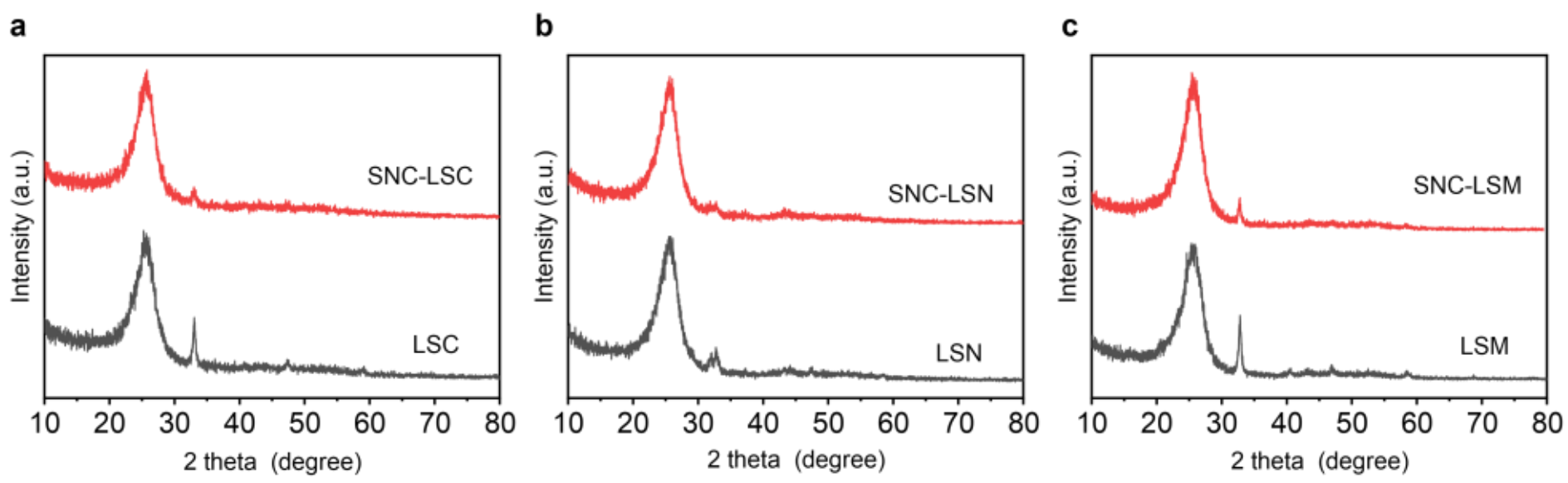

Figure R5 (also **Supplementary Figure 48**). XRD patterns of (a) LSC and SNC-LSC, (b) LSN and SNC-LSN, and (c) LSM and SNC-LSM.

Related date (**Supplementary Figure 18**) and discussions (yellow-highlighted in **Line 218-222, Page 9**) have been added in the revised manuscript as follows:

Line 218-222, Page 9: *"The surface amorphization was also confirmed by the decrease in peak intensity of powder X-ray diffraction (XRD) pattern in for SNC-LSF compared to LSF (**Supplementary Fig. 17**), with similar peak attenuation also observed for SNC-LCF and SNC-LBF relative to their corresponding pristine phases* (**Supplementary Fig. 18**)*."*

Comment 7: The NITRR activity of carbon black used in electrodes has not been measured to exclude background contributions. To demonstrate the generality of hydroxyl-rich nanocavity formation via electrochemical reduction-assisted selective ion leaching, the authors substituted different A-site and B-site elements in the perovskite lattice. However, basic structural information (such as XRD patterns) for the corresponding perovskites (LCF, LBF, LSC, LSN, and LSM), are suggested to be given.

Response to C7: We thank the reviewer for these helpful comments. **As suggested by the reviewer**, to exclude possible background contributions from carbon black, control experiments using carbon black alone were first performed under identical NITRR conditions. As shown in **Figure R6**, **negligible ammonia production** confirms that the observed catalytic performance of SNC-LSF mainly originates from the perovskite catalysts rather than the conductive additive. These data have been included in the revised manuscript. In addition, we have now added the XRD patterns of LCF, LBF, LSC, LSN, and LSM in the revised Supporting Information (**Figure R7 and R8**).

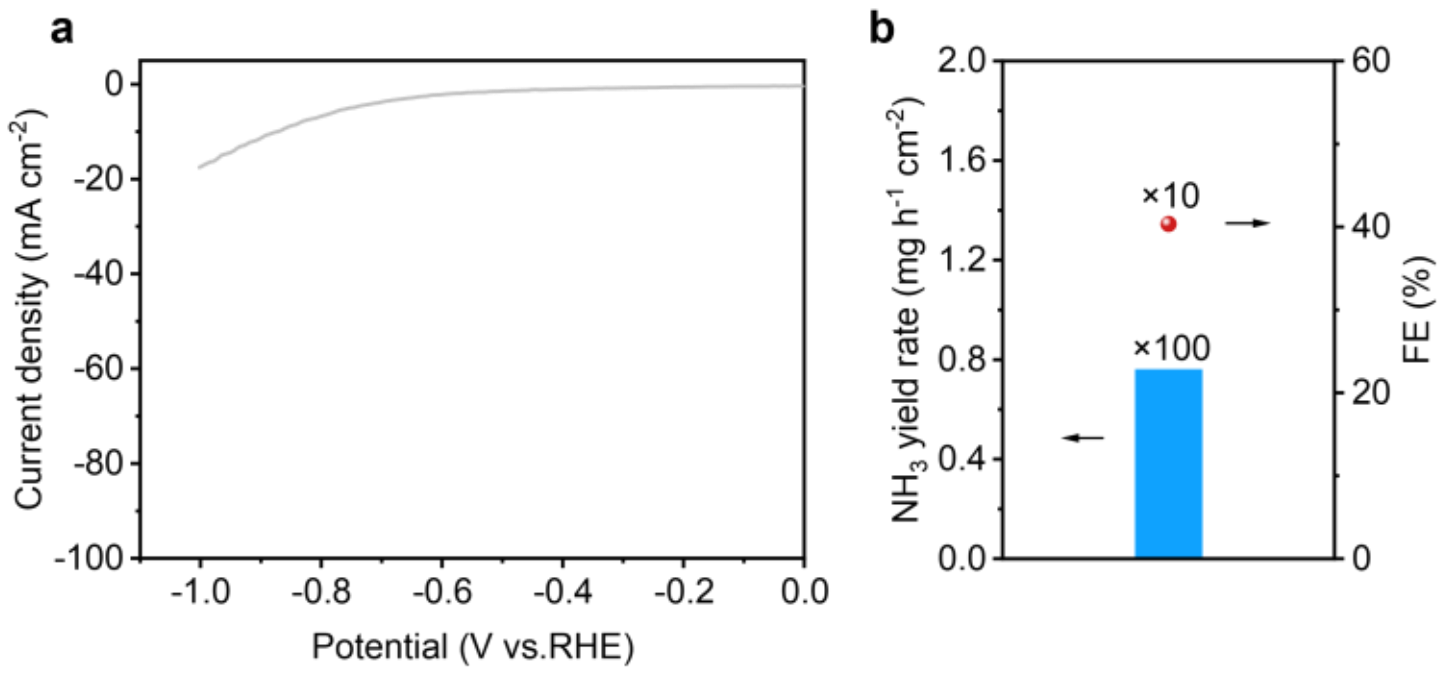

Figure R6 (also **Supplementary Figure 43)**. NITRR performance of carbon black. (a) LSV polarization curves of carbon black, (b) NH_3 yield rate and FE_{NH3} of carbon black in 0.1 M KOH with 8 mM KNO_3 at -0.4 V vs. RHE.

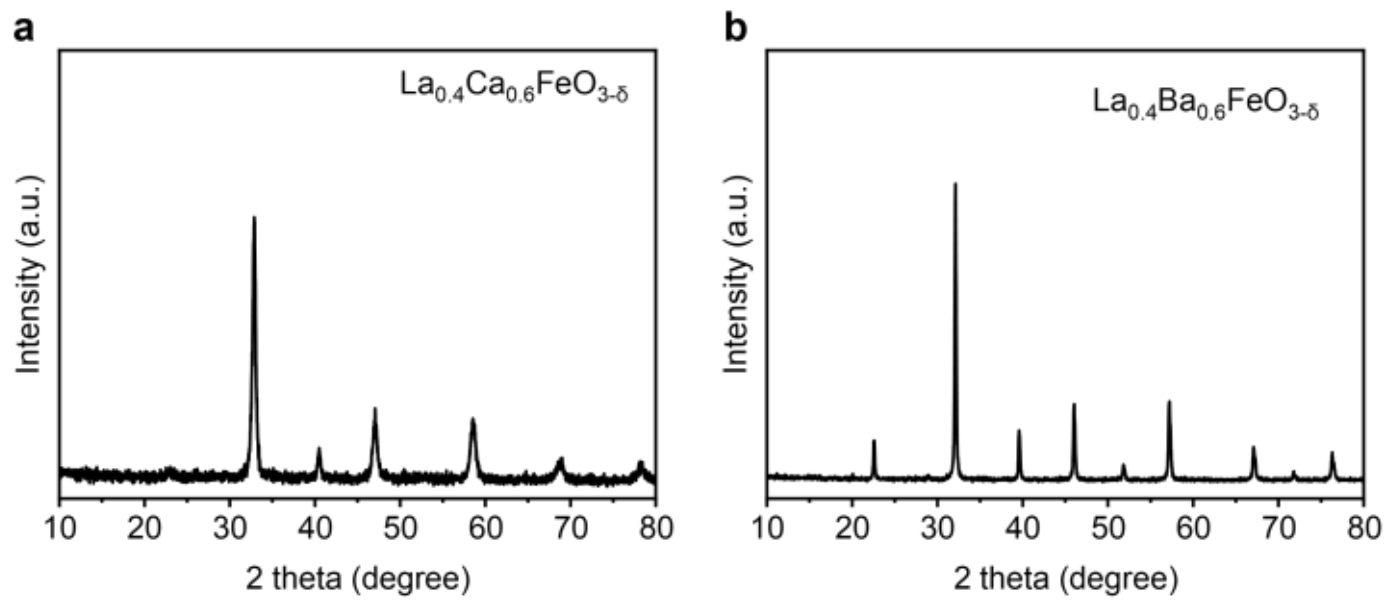

Figure R7 (also **Supplementary Figure 6)**. XRD patterns of (a) $La_{0.4}Ca_{0.6}FeO_{3-\delta}$, (b) $La_{0.4}Ba_{0.6}FeO_{3-\delta}$.

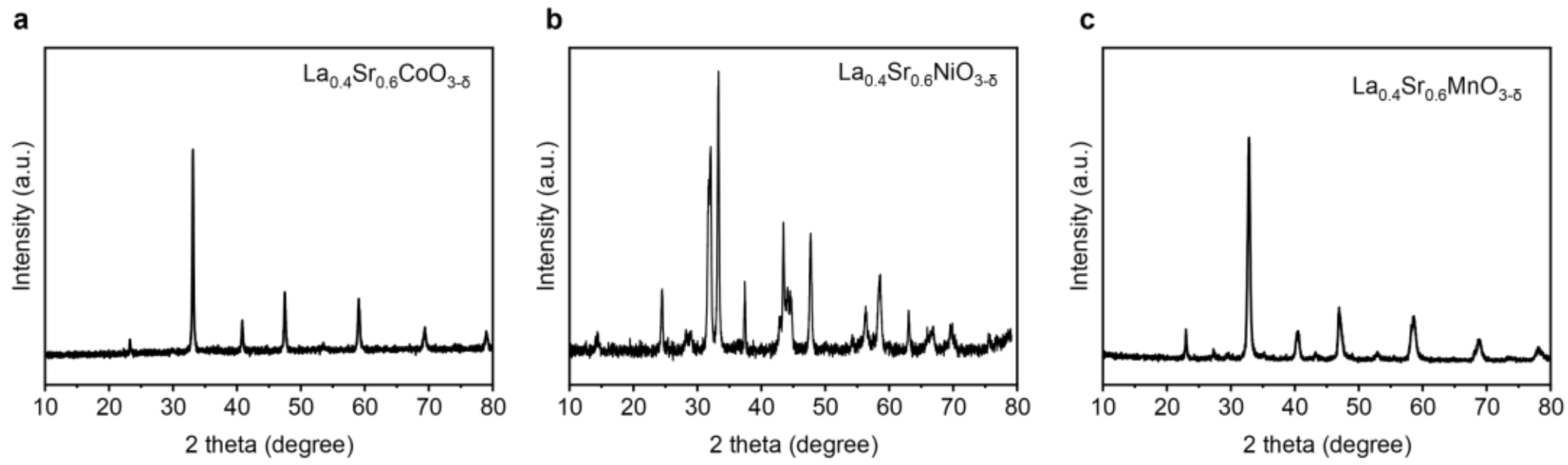

Figure R8 (also **Supplementary Figure 47)**. XRD patterns of (a) $La_{0.4}Sr_{0.6}CoO_{3-\delta}$, (b) $La_{0.4}Sr_{0.6}NiO_{3-\delta}$, and (c) $La_{0.4}Sr_{0.6}MnO_{3-\delta}$.

Related data **(Supplementary Fig. 43)** and discussions (yellow-highlighted in **Line 520-522, Page 21**) have been added in the revised manuscript as follows:

Line 520-522, Page 21: *"To rule out the potential background contributions of carbon black, its NITRR activity was measured, which exhibits a negligible NITRR activity (**Supplementary Figure 43**)."*

Comment 8: Standard calibration curves for NO_3^- quantification are still missing, undermining the reliability of selectivity calculations. The authors should be offered the standard curves for NO3- quantification, as it is essential for accurately evaluating product selectivity.

Response to C8: We thank the reviewer for this important comment. We agree that standard calibration curves are essential for reliable quantification. **As suggested by the reviewer**, we have added the standard calibration curves for NO_3^- quantification in the Supporting Information in the revised manuscript. These curves were used for all selectivity and conversion calculations to ensure accuracy and reproducibility.

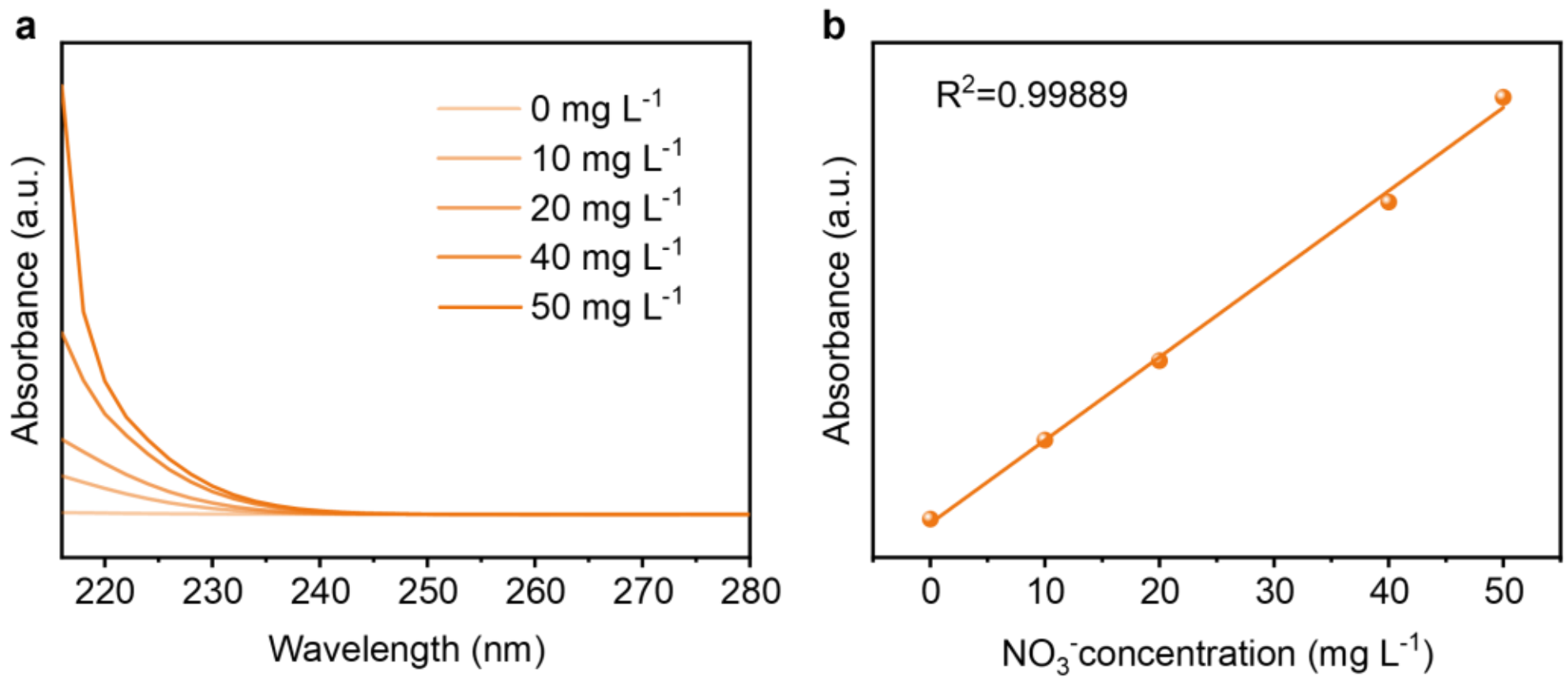

Figure R9 (also **Supplementary Figure 56)**. (a) UV-vis curves and (b) corresponding calibration curve with known NO_3^- concentrations.

Comment 9: The issue of ammonia crossover in MEA tests and its influence on Faradaic efficiency have not been addressed or corrected. For the tests in MEA device, the crossover of ammonia from cathode to anode probably occurred, which may lower the FE of product. Please explain how these issues were addressed or avoided.

Response to C9: We thank the reviewer for raising this question. We **agree with** the reviewer that ammonia crossover from the cathode to the anode can occur in MEA devices, which may lead to an underestimation of the apparent Faradaic efficiency if not properly accounted for. In our study, this effect was **explicitly considered** in the FE calculation. Specifically, ammonia produced at the cathode and subsequently transported to the anode compartment was quantitatively analyzed and included in the

total ammonia amount used for Faradaic efficiency determination. Therefore, the reported FE values represent the **overall ammonia production of the MEA system**, rather than the catholyte-side yield alone.

Comment 10: (One minor comment) Figure 5d is partially overlapped with Figure 5e, please rearrange the layout to ensure these graphs are visibly displayed.

Response to C10: We thank the reviewer for this helpful comment. **As suggested by the reviewer**, we have carefully revised the layout of Figure 5d and Figure 5e to eliminate overlap and ensure clear visualization. The updated figures are presented in the revised manuscript.

Reviewer #3

GENERAL COMMENTS: The authors have resolved my concerns.

Response: We thank the reviewer for this constructive review process and strong support on the publication of this work.

This manuscript reports the rational design of a perovskite oxide with a nano-concave surface, $La_{0.4}Sr_{0.6}FeO_{3-\delta}$ (SNC-LSF), synthesized via electrochemical reduction-assisted selective Sr^{2+} leaching for efficient electrocatalytic nitrate reduction to ammonia. The authors propose a highly innovative hydrogen hopping mechanism, where a hydroxyl-rich nanocavity creates a nearly barrierless intramolecular hydrogen transfer pathway along hydrogen-bond networks. The reported performance (a Faradaic efficiency of 97.81% and an ammonia yield rate of 51.37 mg $h^{-1}cm^{-2}$ at -0.8 V vs. RHE) is truly remarkable and surpasses most state-of-the-art NITRR catalysts.

The mechanistic investigations are exceptionally thorough, integrating operando ATR-SEIRAS, on-line DEMS, and AIMD/DFT simulations to substantiate the Grotthuss-like hydrogen transport mechanism. Furthermore, the practical demonstration of an industrial-level MEA electrolyzer powered by solar energy provides strong evidence for the technology's scalability and economic viability.

However, despite the high quality of the work, there are a few minor points regarding the kinetic competition between NITRR and HER at high overpotentials and the long-term surface integrity that require further clarification to strengthen the manuscript's impact. Therefore, I recommend Revision.

1. The authors report a remarkable Faradaic efficiency (FE) of 97.81% at a high negative potential of -0.8 V vs. RHE under 1000 mM nitrate conditions. However, the LSV curve for SNC-LSF in Figure 4a measured without NO_3^- clearly shows a significant current density for the HER at this potential range. Given this high intrinsic HER activity, could the authors provide a more detailed kinetic rationale for suppressing HER pathways to maintain such high selectivity? Specifically, I suggest including a quantitative comparison of reaction rate constants of NITRR versus HER or more detailed kinetic analyses at high overpotentials, as the Tafel data in Figure 2f appear to be collected under relatively low nitrate concentrations and may not fully represent the behavior at 1000 mM.

2. The amorphous reconstructed surface layer, approximately 10 nm in thickness, is presented as the cornerstone of the catalyst's high activity. Is there any possibility that

this metastable amorphous structure, formed through Sr leaching, might undergo recrystallization or transition back into an oxide form when the potential is removed or upon exposure to air? Furthermore, while the catalyst demonstrated impressive durability over 240 hours in the MEA system, could the authors provide HRTEM images obtained after the stability test to confirm that the 10 nm amorphous layer remains structurally intact and unchanged after such prolonged industrial-level operation?

3. The attempt to validate the catalyst's performance using real industrial wastewater is highly practical and adds significant value to this work. However, the experimental data using real wastewater appear to be insufficient. Please provide additional data, such as LSV curves or long-term stability test results, conducted in the presence of impurities from the wastewater.

4. It is noted that the electrolyte was replenished throughout the 240-hour long-term test to compensate for nitrate depletion. Has any analysis been performed on the potential impact of accumulated reaction by-products or the possible re-adsorption of initially leached Sr^{2+} ions back onto the catalyst surface?
