# Supplementary material for: Hydroxyl-rich nanocavities on perovskite enable nearly barrierless intramolecular hydrogen transfer for nitrate electroreduction to ammonia
Source: Nat Commun. 2026 Jun 29;17:8062. doi: 10.1038/s41467-026-74405-1 (PMC13454618; doi:10.1038/s41467-026-74405-1)
Supplement: Supplementary file 4 [file 41467_2026_74405_MOESM3_ESM.docx]

LSF

_cell_length_a	10.9835

_cell_length_b	19.0240

_cell_length_c	22.8470

_cell_angle_alpha	90.0000

_cell_angle_beta	90.0000

_cell_angle_gamma	90.0000

loop_

_atom_site_label

_atom_site_type_symbol

_atom_site_fract_x

_atom_site_fract_y

_atom_site_fract_z

_atom_site_U_iso_or_equiv

_atom_site_adp_type

_atom_site_occupancy

La1	La	-0.01052	0.66356	0.76624	0.00127 Uiso	1.00

La2	La	0.48948	0.16356	0.76624	0.00127 Uiso	1.00

La3	La	-0.01052	0.33023	0.56998	0.00127 Uiso	1.00

La4	La	0.48948	0.83023	0.56998	0.00127 Uiso	1.00

Fe5	Fe	-0.01052	0.66356	0.91344	0.00155 Uiso	1.00

Fe6	Fe	0.48948	0.16356	0.91344	0.00155 Uiso	1.00

Fe7	Fe	-0.01052	0.33023	0.71717	0.00155 Uiso	1.00

Fe8	Fe	0.48948	0.83023	0.71717	0.00155 Uiso	1.00

O9	O	0.11448	0.70523	0.86437	0.01204 Uiso	1.00

O10	O	0.61448	0.20523	0.86437	0.01204 Uiso	1.00

O11	O	0.11448	0.37189	0.66811	0.01204 Uiso	1.00

O12	O	0.61448	0.87189	0.66811	0.01204 Uiso	1.00

O13	O	-0.01052	0.58023	0.86437	0.01204 Uiso	1.00

O14	O	0.48948	0.08023	0.86437	0.01204 Uiso	1.00

O15	O	-0.01052	0.24689	0.66811	0.01204 Uiso	1.00

O16	O	0.48948	0.74689	0.66811	0.01204 Uiso	1.00

O17	O	0.36448	0.20523	0.86437	0.01204 Uiso	1.00

O18	O	0.86448	0.70523	0.86437	0.01204 Uiso	1.00

O19	O	0.36448	0.87189	0.66811	0.01204 Uiso	1.00

O20	O	0.86448	0.37189	0.66811	0.01204 Uiso	1.00

La21	La	0.23948	0.08023	0.86437	0.00127 Uiso	1.00

La22	La	0.73948	0.58023	0.86437	0.00127 Uiso	1.00

La23	La	0.23948	0.74689	0.66811	0.00127 Uiso	1.00

La24	La	0.73948	0.24689	0.66811	0.00127 Uiso	1.00

Fe25	Fe	0.23948	0.74689	0.81530	0.00155 Uiso	1.00

Fe26	Fe	0.73948	0.24689	0.81530	0.00155 Uiso	1.00

Fe27	Fe	0.23948	0.41356	0.61904	0.00155 Uiso	1.00

Fe28	Fe	0.73948	0.91356	0.61904	0.00155 Uiso	1.00

O29	O	0.36448	0.78856	0.76624	0.01204 Uiso	1.00

O30	O	0.86448	0.28856	0.76624	0.01204 Uiso	1.00

O31	O	0.36448	0.45523	0.56998	0.01204 Uiso	1.00

O32	O	0.86448	0.95523	0.56998	0.01204 Uiso	1.00

O33	O	0.23948	0.66356	0.76624	0.01204 Uiso	1.00

O34	O	0.73948	0.16356	0.76624	0.01204 Uiso	1.00

O35	O	0.23948	0.33023	0.56998	0.01204 Uiso	1.00

O36	O	0.73948	0.83023	0.56998	0.01204 Uiso	1.00

O37	O	0.11448	0.78856	0.76624	0.01204 Uiso	1.00

O38	O	0.61448	0.28856	0.76624	0.01204 Uiso	1.00

O39	O	0.11448	0.45523	0.56998	0.01204 Uiso	1.00

O40	O	0.61448	0.95523	0.56998	0.01204 Uiso	1.00

Sr41	Sr	-0.01052	0.83023	0.86437	0.00127 Uiso	1.00

Sr42	Sr	0.48948	0.33023	0.86437	0.00127 Uiso	1.00

Sr43	Sr	-0.01052	0.49689	0.66811	0.00127 Uiso	1.00

Sr44	Sr	0.48948 -0.00311	0.66811	0.00127 Uiso	1.00

Fe45	Fe	-0.01052	0.49689	0.81530	0.00155 Uiso	1.00

Fe46	Fe	0.48948 -0.00311	0.81530	0.00155 Uiso	1.00

Fe47	Fe	-0.01052	0.16356	0.61904	0.00155 Uiso	1.00

Fe48	Fe	0.48948	0.66356	0.61904	0.00155 Uiso	1.00

O49	O	0.11448	0.53856	0.76624	0.01204 Uiso	1.00

O50	O	0.61448	0.03856	0.76624	0.01204 Uiso	1.00

O51	O	0.11448	0.20523	0.56998	0.01204 Uiso	1.00

O52	O	0.61448	0.70523	0.56998	0.01204 Uiso	1.00

O53	O	-0.01052	0.41356	0.76624	0.01204 Uiso	1.00

O54	O	0.48948	0.91356	0.76624	0.01204 Uiso	1.00

O55	O	-0.01052	0.08023	0.56998	0.01204 Uiso	1.00

O56	O	0.48948	0.58023	0.56998	0.01204 Uiso	1.00

O57	O	0.36448	0.03856	0.76624	0.01204 Uiso	1.00

O58	O	0.86448	0.53856	0.76624	0.01204 Uiso	1.00

O59	O	0.36448	0.70523	0.56998	0.01204 Uiso	1.00

O60	O	0.86448	0.20523	0.56998	0.01204 Uiso	1.00

Sr61	Sr	0.23948	0.91356	0.76624	0.00127 Uiso	1.00

Sr62	Sr	0.73948	0.41356	0.76624	0.00127 Uiso	1.00

Sr63	Sr	0.23948	0.58023	0.56998	0.00127 Uiso	1.00

Sr64	Sr	0.73948	0.08023	0.56998	0.00127 Uiso	1.00

Fe65	Fe	0.23948	0.91356	0.91344	0.00155 Uiso	1.00

Fe66	Fe	0.73948	0.41356	0.91344	0.00155 Uiso	1.00

Fe67	Fe	0.23948	0.58023	0.71717	0.00155 Uiso	1.00

Fe68	Fe	0.73948	0.08023	0.71717	0.00155 Uiso	1.00

O69	O	0.36448	0.95523	0.86437	0.01204 Uiso	1.00

O70	O	0.86448	0.45523	0.86437	0.01204 Uiso	1.00

O71	O	0.36448	0.62189	0.66811	0.01204 Uiso	1.00

O72	O	0.86448	0.12189	0.66811	0.01204 Uiso	1.00

O73	O	0.23948	0.83023	0.86437	0.01204 Uiso	1.00

O74	O	0.73948	0.33023	0.86437	0.01204 Uiso	1.00

O75	O	0.23948	0.49689	0.66811	0.01204 Uiso	1.00

O76	O	0.73948 -0.00311	0.66811	0.01204 Uiso	1.00

O77	O	0.11448	0.95523	0.86437	0.01204 Uiso	1.00

O78	O	0.61448	0.45523	0.86437	0.01204 Uiso	1.00

O79	O	0.11448	0.62189	0.66811	0.01204 Uiso	1.00

O80	O	0.61448	0.12189	0.66811	0.01204 Uiso	1.00

Sr81	Sr	0.23948	0.58023	0.86437	0.00127 Uiso	1.00

Sr82	Sr	0.73948	0.08023	0.86437	0.00127 Uiso	1.00

Sr83	Sr	0.23948	0.24689	0.66811	0.00127 Uiso	1.00

Sr84	Sr	0.73948	0.74689	0.66811	0.00127 Uiso	1.00

Fe85	Fe	0.23948	0.24689	0.81530	0.00155 Uiso	1.00

Fe86	Fe	0.73948	0.74689	0.81530	0.00155 Uiso	1.00

Fe87	Fe	0.23948	0.91356	0.61904	0.00155 Uiso	1.00

Fe88	Fe	0.73948	0.41356	0.61904	0.00155 Uiso	1.00

O89	O	0.36448	0.28856	0.76624	0.01204 Uiso	1.00

O90	O	0.86448	0.78856	0.76624	0.01204 Uiso	1.00

O91	O	0.36448	0.95523	0.56998	0.01204 Uiso	1.00

O92	O	0.86448	0.45523	0.56998	0.01204 Uiso	1.00

O93	O	0.23948	0.16356	0.76624	0.01204 Uiso	1.00

O94	O	0.73948	0.66356	0.76624	0.01204 Uiso	1.00

O95	O	0.23948	0.83023	0.56998	0.01204 Uiso	1.00

O96	O	0.73948	0.33023	0.56998	0.01204 Uiso	1.00

O97	O	0.11448	0.28856	0.76624	0.01204 Uiso	1.00

O98	O	0.61448	0.78856	0.76624	0.01204 Uiso	1.00

O99	O	0.11448	0.95523	0.56998	0.01204 Uiso	1.00

O100	O	0.61448	0.45523	0.56998	0.01204 Uiso	1.00

La101 La	-0.01052	0.16356	0.76624	0.00127 Uiso	1.00

La102 La	0.48948	0.66356	0.76624	0.00127 Uiso	1.00

La103 La	-0.01052	0.83023	0.56998	0.00127 Uiso	1.00

La104 La	0.48948	0.33023	0.56998	0.00127 Uiso	1.00

Fe105 Fe	-0.01052	0.16356	0.91344	0.00155 Uiso	1.00

Fe106 Fe	0.48948	0.66356	0.91344	0.00155 Uiso	1.00

Fe107 Fe	-0.01052	0.83023	0.71717	0.00155 Uiso	1.00

Fe108 Fe	0.48948	0.33023	0.71717	0.00155 Uiso	1.00

O109	O	0.11448	0.20523	0.86437	0.01204 Uiso	1.00

O110	O	0.61448	0.70523	0.86437	0.01204 Uiso	1.00

O111	O	0.11448	0.87189	0.66811	0.01204 Uiso	1.00

O112	O	0.61448	0.37189	0.66811	0.01204 Uiso	1.00

O113	O	-0.01052	0.08023	0.86437	0.01204 Uiso	1.00

O114	O	0.48948	0.58023	0.86437	0.01204 Uiso	1.00

O115	O	-0.01052	0.74689	0.66811	0.01204 Uiso	1.00

O116	O	0.48948	0.24689	0.66811	0.01204 Uiso	1.00

O117	O	0.36448	0.70523	0.86437	0.01204 Uiso	1.00

O118	O	0.86448	0.20523	0.86437	0.01204 Uiso	1.00

O119	O	0.36448	0.37189	0.66811	0.01204 Uiso	1.00

O120	O	0.86448	0.87189	0.66811	0.01204 Uiso	1.00

Sr121 Sr	0.23948	0.41356	0.76624	0.00127 Uiso	1.00

Sr122 Sr	0.73948	0.91356	0.76624	0.00127 Uiso	1.00

Sr123 Sr	0.23948	0.08023	0.56998	0.00127 Uiso	1.00

Sr124 Sr	0.73948	0.58023	0.56998	0.00127 Uiso	1.00

Fe125 Fe	0.23948	0.41356	0.91344	0.00155 Uiso	1.00

Fe126 Fe	0.73948	0.91356	0.91344	0.00155 Uiso	1.00

Fe127 Fe	0.23948	0.08023	0.71717	0.00155 Uiso	1.00

Fe128 Fe	0.73948	0.58023	0.71717	0.00155 Uiso	1.00

O129	O	0.36448	0.45523	0.86437	0.01204 Uiso	1.00

O130	O	0.86448	0.95523	0.86437	0.01204 Uiso	1.00

O131	O	0.36448	0.12189	0.66811	0.01204 Uiso	1.00

O132	O	0.86448	0.62189	0.66811	0.01204 Uiso	1.00

O133	O	0.23948	0.33023	0.86437	0.01204 Uiso	1.00

O134	O	0.73948	0.83023	0.86437	0.01204 Uiso	1.00

O135	O	0.23948 -0.00311	0.66811	0.01204 Uiso	1.00

O136	O	0.73948	0.49689	0.66811	0.01204 Uiso	1.00

O137	O	0.11448	0.45523	0.86437	0.01204 Uiso	1.00

O138	O	0.61448	0.95523	0.86437	0.01204 Uiso	1.00

O139	O	0.11448	0.12189	0.66811	0.01204 Uiso	1.00

O140	O	0.61448	0.62189	0.66811	0.01204 Uiso	1.00

Sr141 Sr	-0.01052	0.33023	0.86437	0.00127 Uiso	1.00

Sr142 Sr	0.48948	0.83023	0.86437	0.00127 Uiso	1.00

Sr143 Sr	-0.01052 -0.00311	0.66811	0.00127 Uiso	1.00

Sr144 Sr	0.48948	0.49689	0.66811	0.00127 Uiso	1.00

Fe145 Fe	-0.01052 -0.00311	0.81530	0.00155 Uiso	1.00

Fe146 Fe	0.48948	0.49689	0.81530	0.00155 Uiso	1.00

Fe147 Fe	-0.01052	0.66356	0.61904	0.00155 Uiso	1.00

Fe148 Fe	0.48948	0.16356	0.61904	0.00155 Uiso	1.00

O149	O	0.11448	0.03856	0.76624	0.01204 Uiso	1.00

O150	O	0.61448	0.53856	0.76624	0.01204 Uiso	1.00

O151	O	0.11448	0.70523	0.56998	0.01204 Uiso	1.00

O152	O	0.61448	0.20523	0.56998	0.01204 Uiso	1.00

O153	O	-0.01052	0.91356	0.76624	0.01204 Uiso	1.00

O154	O	0.48948	0.41356	0.76624	0.01204 Uiso	1.00

O155	O	-0.01052	0.58023	0.56998	0.01204 Uiso	1.00

O156	O	0.48948	0.08023	0.56998	0.01204 Uiso	1.00

O157	O	0.36448	0.53856	0.76624	0.01204 Uiso	1.00

O158	O	0.86448	0.03856	0.76624	0.01204 Uiso	1.00

O159	O	0.36448	0.20523	0.56998	0.01204 Uiso	1.00

O160	O	0.86448	0.70523	0.56998	0.01204 Uiso	1.00

loop_

_geom_bond_atom_site_label_1

_geom_bond_atom_site_label_2

_geom_bond_distance

_geom_bond_site_symmetry_2

_ccdc_geom_bond_type

Fe5	O9	1.942	. S

Fe5	O13	1.942	. S

Fe5	O18	1.942	1_455 S

Fe6	O10	1.942	. S

Fe6	O14	1.942	. S

Fe6	O17	1.942	. S

Fe7	O11	1.942	. S

Fe7	O30	1.942	1_455 S

Fe7	O15	1.942	. S

Fe7	O53	1.942	. S

Fe7	O20	1.942	1_455 S

Fe7	O97	1.942	. S

Fe8	O12	1.942	. S

Fe8	O29	1.942	. S

Fe8	O16	1.942	. S

Fe8	O54	1.942	. S

Fe8	O19	1.942	. S

Fe8	O98	1.942	. S

O9	Fe25	1.942	. S

O10	Fe26	1.942	. S

O11	Fe27	1.942	. S

O12	Fe28	1.942	. S

O13	Fe45	1.942	. S

O14	Fe46	1.942	. S

O15	Fe47	1.942	. S

O16	Fe48	1.942	. S

O17	Fe85	1.942	. S

O18	Fe5	1.942	1_655 S

O18	Fe86	1.942	. S

O19	Fe87	1.942	. S

O20	Fe7	1.942	1_655 S

O20	Fe88	1.942	. S

Fe25	O29	1.942	. S

Fe25	O33	1.942	. S

Fe25	O73	1.942	. S

Fe25	O37	1.942	. S

Fe25	O117	1.942	. S

Fe26	O30	1.942	. S

Fe26	O34	1.942	. S

Fe26	O74	1.942	. S

Fe26	O38	1.942	. S

Fe26	O118	1.942	. S

Fe27	O31	1.942	. S

Fe27	O35	1.942	. S

Fe27	O75	1.942	. S

Fe27	O39	1.942	. S

Fe27	O119	1.942	. S

Fe28	O32	1.942	. S

Fe28	O36	1.942	. S

Fe28	O76	1.942	1_565 S

Fe28	O40	1.942	. S

Fe28	O120	1.942	. S

O30	Fe7	1.942	1_655 S

O33	Fe67	1.942	. S

O34	Fe68	1.942	. S

O37	Fe107	1.942	. S

O38	Fe108	1.942	. S

Fe45	O49	1.942	. S

Fe45	O70	1.942	1_455 S

Fe45	O53	1.942	. S

Fe45	O58	1.942	1_455 S

Fe45	O137	1.942	. S

Fe46	O50	1.942	. S

Fe46	O69	1.942	1_545 S

Fe46	O54	1.942	1_545 S

Fe46	O57	1.942	. S

Fe46	O138	1.942	1_545 S

Fe47	O51	1.942	. S

Fe47	O72	1.942	1_455 S

Fe47	O55	1.942	. S

Fe47	O60	1.942	1_455 S

Fe47	O139	1.942	. S

Fe48	O52	1.942	. S

Fe48	O71	1.942	. S

Fe48	O56	1.942	. S

Fe48	O59	1.942	. S

Fe48	O140	1.942	. S

O49	Fe67	1.942	. S

O50	Fe68	1.942	. S

O54	Fe46	1.942	1_565 S

O57	Fe127	1.942	. S

O58	Fe45	1.942	1_655 S

O58	Fe128	1.942	. S

O60	Fe47	1.942	1_655 S

Fe65	O69	1.942	. S

Fe65	O73	1.942	. S

Fe65	O77	1.942	. S

Fe66	O70	1.942	. S

Fe66	O74	1.942	. S

Fe66	O78	1.942	. S

Fe67	O71	1.942	. S

Fe67	O75	1.942	. S

Fe67	O79	1.942	. S

Fe67	O157	1.942	. S

Fe68	O72	1.942	. S

Fe68	O76	1.942	. S

Fe68	O80	1.942	. S

Fe68	O158	1.942	. S

O69	Fe46	1.942	1_565 S

O70	Fe45	1.942	1_655 S

O72	Fe47	1.942	1_655 S

O76	Fe28	1.942	1_545 S

O77	Fe145	1.942	1_565 S

O78	Fe146	1.942	. S

O79	Fe147	1.942	. S

O80	Fe148	1.942	. S

Fe85	O89	1.942	. S

Fe85	O109	1.942	. S

Fe85	O93	1.942	. S

Fe85	O133	1.942	. S

Fe85	O97	1.942	. S

Fe86	O90	1.942	. S

Fe86	O110	1.942	. S

Fe86	O94	1.942	. S

Fe86	O134	1.942	. S

Fe86	O98	1.942	. S

Fe87	O91	1.942	. S

Fe87	O111	1.942	. S

Fe87	O95	1.942	. S

Fe87	O135	1.942	1_565 S

Fe87	O99	1.942	. S

Fe88	O92	1.942	. S

Fe88	O112	1.942	. S

Fe88	O96	1.942	. S

Fe88	O136	1.942	. S

Fe88	O100	1.942	. S

O89	Fe108	1.942	. S

O90	Fe107	1.942	1_655 S

O93	Fe127	1.942	. S

O94	Fe128	1.942	. S

Fe105 O109	1.942	. S

Fe105 O113	1.942	. S

Fe105 O118	1.942	1_455 S

Fe106 O110	1.942	. S

Fe106 O114	1.942	. S

Fe106 O117	1.942	. S

Fe107 O111	1.942	. S

Fe107 O90	1.942	1_455 S

Fe107 O115	1.942	. S

Fe107 O153	1.942	. S

Fe107 O120	1.942	1_455 S

Fe108 O112	1.942	. S

Fe108 O116	1.942	. S

Fe108 O154	1.942	. S

Fe108 O119	1.942	. S

O113	Fe145	1.942	. S

O114	Fe146	1.942	. S

O115	Fe147	1.942	. S

O116	Fe148	1.942	. S

O118	Fe105	1.942	1_655 S

O120	Fe107	1.942	1_655 S

Fe125 O129	1.942	. S

Fe125 O133	1.942	. S

Fe125 O137	1.942	. S

Fe126 O130	1.942	. S

Fe126 O134	1.942	. S

Fe126 O138	1.942	. S

Fe127 O131	1.942	. S

Fe127 O149	1.942	. S

Fe127 O135	1.942	. S

Fe127 O139	1.942	. S

Fe128 O132	1.942	. S

Fe128 O150	1.942	. S

Fe128 O136	1.942	. S

Fe128 O140	1.942	. S

O129	Fe146	1.942	. S

O130	Fe145	1.942	1_665 S

O131	Fe148	1.942	. S

O132	Fe147	1.942	1_655 S

O135	Fe87	1.942	1_545 S

O138	Fe46	1.942	1_565 S

Fe145 O149	1.942	. S

Fe145 O130	1.942	1_445 S

Fe145 O153	1.942	1_545 S

Fe145 O158	1.942	1_455 S

Fe145 O77	1.942	1_545 S

Fe146 O150	1.942	. S

Fe146 O154	1.942	. S

Fe146 O157	1.942	. S

Fe147 O151	1.942	. S

Fe147 O132	1.942	1_455 S

Fe147 O155	1.942	. S

Fe147 O160	1.942	1_455 S

Fe148 O152	1.942	. S

Fe148 O156	1.942	. S

Fe148 O159	1.942	. S

O153	Fe145	1.942	1_565 S

O158	Fe145	1.942	1_655 S

O160	Fe147	1.942	1_655 S

SNC-LSF

_cell_angle_alpha 90.000

_cell_angle_beta	90.000

_cell_angle_gamma 90.000

_cell_length_a	10.983500

_cell_length_b	19.024000

_cell_length_c	28.847000

loop_

_symmetry_equiv_pos_as_xyz

x,y,z

loop_

_atom_site_label

_atom_site_fract_x

_atom_site_fract_y

_atom_site_fract_z

Fe	0.48042000	0.07342000	0.02659000

Fe	0.98042000	0.57342000	0.02659000

Fe	0.23042000	0.82342000	0.02659000

Fe	0.73042000	0.32342000	0.02659000

Fe	0.48042000	0.57342000	0.02659000

Fe	0.98042000	0.07342000	0.02659000

Fe	0.23042000	0.32342000	0.02659000

Fe	0.73042000	0.82342000	0.02659000

La	0.23042000	0.99008000	0.06546000

La	0.73042000	0.49008000	0.06546000

O	0.10542000	0.61508000	0.06546000

O	0.60542000	0.11508000	0.06546000

O	0.48042000	0.99008000	0.06546000

O	0.98042000	0.49008000	0.06546000

O	0.35542000	0.11508000	0.06546000

O	0.85542000	0.61508000	0.06546000

O	0.35542000	0.86508000	0.06546000

O	0.85542000	0.36508000	0.06546000

O	0.23042000	0.74008000	0.06546000

O	0.73042000	0.24008000	0.06546000

O	0.10542000	0.86508000	0.06546000

O	0.60542000	0.36508000	0.06546000

O	0.10542000	0.11508000	0.06546000

O	0.60542000	0.61508000	0.06546000

O	0.48042000	0.49008000	0.06546000

O	0.98042000	0.99008000	0.06546000

O	0.35542000	0.61508000	0.06546000

O	0.85542000	0.11508000	0.06546000

O	0.35542000	0.36508000	0.06546000

O	0.85542000	0.86508000	0.06546000

O	0.23042000	0.24008000	0.06546000

O	0.73042000	0.74008000	0.06546000

O	0.10542000	0.36508000	0.06546000

O	0.60542000	0.86508000	0.06546000

Sr	0.48042000	0.24008000	0.06546000

Sr	0.98042000	0.74008000	0.06546000

Sr	0.23042000	0.49008000	0.06546000

Sr	0.73042000	0.99008000	0.06546000

Sr	0.48042000	0.74008000	0.06546000

Sr	0.98042000	0.24008000	0.06546000

H	0.37937000	0.09595000	0.09339000

Fe	0.98042000	0.90675000	0.10431000

Fe	0.23042000	0.15675000	0.10431000

Fe	0.73042000	0.65675000	0.10431000

Fe	0.48042000	0.90675000	0.10431000

Fe	0.98042000	0.40675000	0.10431000

Fe	0.23042000	0.65675000	0.10431000

Fe	0.73042000	0.15675000	0.10431000

Fe	0.48042000	0.40675000	0.10431000

H	0.57463000	0.74104000	0.12840000

O	0.10542000	0.19842000	0.14318000

O	0.60542000	0.69842000	0.14318000

O	0.23042000	0.57342000	0.14318000

O	0.73042000	0.07342000	0.14318000

O	0.10542000	0.69842000	0.14318000

O	0.60542000	0.19842000	0.14318000

O	0.10542000	0.44842000	0.14318000

O	0.60542000	0.94842000	0.14318000

O	0.48042000	0.82342000	0.14318000

O	0.98042000	0.32342000	0.14318000

O	0.35542000	0.94842000	0.14318000

O	0.85542000	0.44842000	0.14318000

La	0.48042000	0.57342000	0.14318000

La	0.98042000	0.07342000	0.14318000

O	0.10542000	0.94842000	0.14318000

O	0.60542000	0.44842000	0.14318000

O	0.48042000	0.32342000	0.14318000

O	0.98042000	0.82342000	0.14318000

O	0.35542000	0.44842000	0.14318000

O	0.85542000	0.94842000	0.14318000

La	0.48042000	0.07342000	0.14318000

La	0.98042000	0.57342000	0.14318000

Sr	0.23042000	0.82342000	0.14318000

Sr	0.73042000	0.32342000	0.14318000

O	0.35542000	0.69842000	0.14318000

O	0.85542000	0.19842000	0.14318000

Sr	0.23042000	0.32342000	0.14318000

Sr	0.73042000	0.82342000	0.14318000

O	0.35542000	0.19842000	0.14318000

O	0.85542000	0.69842000	0.14318000

O	0.23058355	0.07342238	0.14164814

O	0.72945515	0.57379730	0.14287297

La	0.98649574	0.74849563	0.16021175

O	0.88172342	0.51153460	0.15832014

O	0.04463545	0.64956557	0.16390332

La	0.51593661	0.23924080	0.17102204

Fe	0.97919748	0.18464391	0.17275312

H	0.21273282	0.05261545	0.17287758

H	0.68113282	0.57574749	0.17253427

O	0.18551073	0.43507738	0.17716777

H	0.71023337	0.08803282	0.17517639

La	0.41015630	0.74621139	0.18164502

La	0.41378261	0.39823533	0.18097899

La	0.05936011	0.90208708	0.18472267

H	0.36314179	0.21097321	0.17847925

O	0.57485327	0.99606959	0.18528356

O	0.90009560	0.84354756	0.17651874

La	0.53489773	0.89529179	0.19089166

Fe	0.32174848	0.62463738	0.18444934

O	0.54243146	0.81745678	0.19206459

Fe	0.32942887	0.51832021	0.18582208

O	0.55243728	0.33798926	0.19385947

Fe	0.83253526	0.01317147	0.18818332

O	0.99908242	0.50696773	0.19593460

O	0.82157190	0.72094182	0.19251709

La	0.91484017	0.40159281	0.19897972

O	0.39019029	0.07444872	0.19918135

Fe	0.65670418	0.44673937	0.19438499

Fe	0.39759175	0.98777344	0.19611604

H	0.85901448	0.61219508	0.19927996

O	0.21690216	0.85759470	0.21235838

O	0.71622505	0.89413774	0.20785174

O	0.43306303	0.66855639	0.21473509

O	0.79230609	0.31487028	0.21465109

O	0.05643704	0.11471200	0.21471135

O	0.30775257	0.33545994	0.20790350

O	0.24005612	0.22763811	0.21411610

O	0.42942106	0.84299055	0.22752480

Fe	0.05615805	0.65573773	0.22123885

O	1.00398985	0.82340127	0.22898021

La	0.81113269	0.80738563	0.23129384

O	0.35832677	0.11884842	0.22586623

O	0.94526898	0.98002967	0.23002879

O	0.60584631	0.10177215	0.22798942

O	0.28592682	0.57090268	0.23029928

O	0.45937532	0.24925959	0.23326225

O	0.01988704	0.35770746	0.22769806

Fe	0.08882492	0.98955694	0.23045780

O	0.80750785	0.07828850	0.22935489

O	0.77826170	0.45658427	0.23637541

Fe	0.55843787	0.18053223	0.23335835

O	0.65425755	0.58738100	0.22981743

Fe	0.56733901	0.70043021	0.23496458

O	0.88684849	0.61309445	0.23191351

O	0.47589952	0.48657616	0.23087334

O	0.24104848	0.98702859	0.23174390

La	0.14936595	0.30246538	0.22476865

Fe	0.11356119	0.18335457	0.23585095

H	0.50741826	0.02658027	0.23838716

O	0.68562308	0.74339701	0.23999236

H	0.22873554	0.88220310	0.24034050

H	0.51786881	0.52676764	0.24315150

Fe	0.92062641	0.29404944	0.25012504

O	0.47213771	0.98238802	0.25232406

O	0.10514134	0.49786759	0.25142290

O	0.11446035	0.71508617	0.25563570

La	0.36971343	0.41637190	0.26516340

Fe	0.67075666	0.91120684	0.27047520

O	0.30541149	0.31540967	0.27087689

O	0.84467569	0.42163784	0.26603206

Fe	0.24513794	0.75276529	0.27029225

O	0.87314037	0.22751584	0.27207503

O	0.18223317	0.38352489	0.28132250

Fe	0.75883323	0.13025812	0.27361818

H	0.89181943	0.67068728	0.27540784

Fe	0.73171233	0.56560897	0.27980187

H	0.75061319	0.02329345	0.28025496

O	0.51634834	0.35937915	0.28225867

Fe	0.20363936	0.53488688	0.28467521

O	0.37809917	0.71946210	0.28858124

O	0.88887394	0.71844114	0.28830679

H	0.43932172	0.60845107	0.28709199

O	0.57403304	0.84740692	0.29059905

O	0.09408920	0.22782119	0.29177029

H	0.99571737	0.90630150	0.29315326

H	0.04715051	0.06436438	0.29233466

H	0.96759248	0.56248470	0.29412147

O	0.68198943	-0.00154971	0.29662894

Fe	0.49532508	0.77178225	0.29918508

O	0.06206486	0.93868539	0.30296336

O	0.78289426	0.85899104	0.30290800

O	0.48617673	0.57027899	0.30507128

H	0.45955243	0.06666784	0.30414951

H	0.03005616	0.19790147	0.30554551

O	0.25207320	0.82686948	0.30591860

O	0.97523248	0.07577703	0.31270846

H	0.90997907	0.70165059	0.31905307

H	0.73178334	0.82624014	0.32240203

Fe	0.60471730	0.19684203	0.32294885

O	0.98754986	0.56671630	0.32491765

Fe	0.59146826	0.33747457	0.32626974

O	0.70311053	0.12694131	0.33099217

O	0.26916901	0.51531946	0.33311771

H	0.46004241	0.58452090	0.33564381

O	0.70861197	0.56328510	0.33610943

H	0.53165469	0.89840265	0.33542299

Fe	0.22299169	0.89450578	0.33300606

O	0.45174080	0.06508106	0.33772782

H	0.53714871	0.07823014	0.34161817

H	0.97251383	0.06606094	0.34522037

H	0.49969654	0.45196891	0.34533896

H	0.05895099	0.56836799	0.34805328

H	0.19648908	0.66703930	0.34821051

O	0.47377661	0.20726786	0.35207780

H	0.46678160	0.95727601	0.35484749

Fe	0.81338614	0.05723005	0.35571828

Fe	0.83886844	0.58993415	0.36138468

O	0.48892269	0.90909732	0.36450393

O	0.69055258	0.32667733	0.36703545

O	0.12676514	0.68314232	0.36636364

O	0.48764842	0.41194472	0.36696388

O	0.21197605	0.96441585	0.36098080

O	0.23833225	0.21585875	0.36333677

Fe	0.85219953	0.89858562	0.37021259

O	0.84267202	0.98665382	0.37512523

H	0.60232912	0.60882204	0.37748564

H	0.39499020	0.09861512	0.38080540

O	0.46535044	0.74919122	0.38108632

Fe	0.36880841	0.19726776	0.38897312

O	0.26125027	0.26754438	0.39377076

O	0.79208629	0.82336443	0.38969713

O	0.89573708	0.11934003	0.38644132

H	0.17368287	0.69455589	0.39419151

H	0.38752199	0.85429443	0.39435492

H	0.48702345	0.79378485	0.39581649

O	0.15958695	0.01445028	0.39214074

H	0.03513665	0.83929697	0.39874431

Fe	0.14157735	0.28471577	0.40933084

La	0.52939442	0.28495497	0.40663330

O	0.54052276	0.60683234	0.40208073

O	0.02139863	0.88899262	0.40531348

H	0.75753173	0.15789958	0.40512241

O	0.91438025	0.60184485	0.40747772

O	0.32540559	0.86272808	0.41119544

O	0.37200150	0.12104372	0.41217148

H	0.84483051	0.82139363	0.41737743

Fe	0.44970248	0.67594103	0.42190268

H	0.98868417	0.28510639	0.41578673

La	0.23542358	0.93598332	0.43628760

O	0.73006633	0.19013507	0.43018449

O	0.43260879	0.99669972	0.43111232

H	0.67827593	0.97145022	0.43432359

O	0.17732320	0.34001993	0.43863048

Fe	0.89867866	0.14165038	0.44517912

H	0.83480869	0.27140616	0.44492067

O	0.91303031	0.29599204	0.44173813

O	0.35376943	0.66039064	0.45932073

H	0.62433071	0.19240313	0.46297904

O	0.98075135	0.27233237	0.47234565

O	0.64846388	0.96247098	0.46620293

Fe	0.48491834	0.94797293	0.47429004

Fe	0.08018166	0.23860015	0.47250893

O	0.56445186	0.21510435	0.48381808

H	0.27053252	0.04376154	0.48965656

O	0.21565872	0.00644763	0.49418667

O	0.88667505	0.10574763	0.50046142

O	0.20642768	0.92615694	0.50107295

O	0.14977570	0.77988818	0.50037754

O	0.18643369	0.20966803	0.50865190

O	0.45437148	0.90045219	0.51635307

Fe	0.18338758	0.85201498	0.52460461

O	0.87982575	0.07797734	0.53578221

O	0.06907876	0.22592503	0.57643885

Fe	0.95029583	0.17868612	0.58515775

O	0.83058777	0.22465164	0.58693271

O	0.14386727	0.87842400	0.58711863

O	0.92908205	0.10067003	0.60703056

H	0.15525017	0.84479431	0.61344644

O	0.47466792	0.07202654	0.97163273

LF

_cell_length_a	11.130000

_cell_length_b	15.706000

_cell_length_c	31.545300

_cell_angle_alpha	90.000000

_cell_angle_beta	90.000000

_cell_angle_gamma	90.000000

_cell_volume	5514.363952

_space_group_name_H-M_alt	'P 1'

_space_group_IT_number	1

loop_

_space_group_symop_operation_xyz

'x, y, z'

loop_

_atom_site_label

_atom_site_occupancy

_atom_site_fract_x

_atom_site_fract_y

_atom_site_fract_z

_atom_site_adp_type

_atom_site_U_iso_or_equiv

_atom_site_type_symbol

O1	1.0	0.102520	0.021950	0.075530	Uiso ? O

O2	1.0	0.602520	0.021950	0.075530	Uiso ? O

O3	1.0	0.102520	0.311750	0.075530	Uiso ? O

O4	1.0	0.602520	0.311750	0.075530	Uiso ? O

O5	1.0	0.102520	0.521950	0.075530	Uiso ? O

O6	1.0	0.602520	0.521950	0.075530	Uiso ? O

O7	1.0	0.102520	0.811750	0.075530	Uiso ? O

O8	1.0	0.602520	0.811750	0.075530	Uiso ? O

O9	1.0	0.352520	0.021950	0.085920	Uiso ? O

O10	1.0	0.852520	0.021950	0.085920	Uiso ? O

O11	1.0	0.352520	0.811750	0.085920	Uiso ? O

O12	1.0	0.852520	0.811750	0.085920	Uiso ? O

O13	1.0	0.352520	0.521950	0.085920	Uiso ? O

O14	1.0	0.852520	0.521950	0.085920	Uiso ? O

O15	1.0	0.352520	0.311750	0.085920	Uiso ? O

O16	1.0	0.852520	0.311750	0.085920	Uiso ? O

O17	1.0	0.487030	0.166850	0.111290	Uiso ? O

O18	1.0	0.987030	0.166850	0.111290	Uiso ? O

O19	1.0	0.487030	0.666850	0.111290	Uiso ? O

O20	1.0	0.987030	0.666850	0.111290	Uiso ? O

La21	1.0	0.228030	0.916850	0.124430	Uiso ? La

La22	1.0	0.728030	0.916850	0.124430	Uiso ? La

La23	1.0	0.228030	0.416850	0.124430	Uiso ? La

La24	1.0	0.728030	0.416850	0.124430	Uiso ? La

Fe25	1.0	0.492210	0.041850	0.124750	Uiso ? Fe

Fe26	1.0	0.992210	0.041850	0.124750	Uiso ? Fe

Fe27	1.0	0.492210	0.291850	0.124750	Uiso ? Fe

Fe28	1.0	0.992210	0.291850	0.124750	Uiso ? Fe

Fe29	1.0	0.492210	0.541850	0.124750	Uiso ? Fe

Fe30	1.0	0.992210	0.541850	0.124750	Uiso ? Fe

Fe31	1.0	0.492210	0.791850	0.124750	Uiso ? Fe

Fe32	1.0	0.992210	0.791850	0.124750	Uiso ? Fe

La33	1.0	0.256380	0.666850	0.125060	Uiso ? La

La34	1.0	0.756380	0.666850	0.125060	Uiso ? La

La35	1.0	0.256380	0.166850	0.125060	Uiso ? La

La36	1.0	0.756380	0.166850	0.125060	Uiso ? La

O37	1.0	0.497380	0.416850	0.138200	Uiso ? O

O38	1.0	0.997380	0.416850	0.138200	Uiso ? O

O39	1.0	0.497380	0.916850	0.138200	Uiso ? O

O40	1.0	0.997380	0.916850	0.138200	Uiso ? O

O41	1.0	0.131890	0.771950	0.163570	Uiso ? O

O42	1.0	0.631890	0.771950	0.163570	Uiso ? O

O43	1.0	0.131890	0.271950	0.163570	Uiso ? O

O44	1.0	0.631890	0.271950	0.163570	Uiso ? O

O45	1.0	0.131890	0.061750	0.163570	Uiso ? O

O46	1.0	0.631890	0.061750	0.163570	Uiso ? O

O47	1.0	0.131890	0.561750	0.163570	Uiso ? O

O48	1.0	0.631890	0.561750	0.163570	Uiso ? O

O49	1.0	0.381890	0.271950	0.173960	Uiso ? O

O50	1.0	0.881890	0.271950	0.173960	Uiso ? O

O51	1.0	0.381890	0.771950	0.173960	Uiso ? O

O52	1.0	0.881890	0.771950	0.173960	Uiso ? O

O53	1.0	0.381890	0.061750	0.173960	Uiso ? O

O54	1.0	0.881890	0.061750	0.173960	Uiso ? O

O55	1.0	0.381890	0.561750	0.173960	Uiso ? O

O56	1.0	0.881890	0.561750	0.173960	Uiso ? O

O57	1.0	0.247380	0.416850	0.199330	Uiso ? O

O58	1.0	0.747380	0.416850	0.199330	Uiso ? O

O59	1.0	0.247380	0.916850	0.199330	Uiso ? O

O60	1.0	0.747380	0.916850	0.199330	Uiso ? O

La61	1.0	0.007520	0.666150	0.208970	Uiso ? La

La62	1.0	0.509330	0.665360	0.209920	Uiso ? La

La63	1.0	0.009430	0.166710	0.210290	Uiso ? La

La64	1.0	0.507430	0.167870	0.210850	Uiso ? La

Fe65	1.0	0.244100	0.042640	0.213180	Uiso ? Fe

Fe66	1.0	0.740860	0.042420	0.213240	Uiso ? Fe

Fe67	1.0	0.243640	0.541930	0.213080	Uiso ? Fe

Fe68	1.0	0.740710	0.538550	0.213180	Uiso ? Fe

Fe69	1.0	0.243840	0.291980	0.213420	Uiso ? Fe

Fe70	1.0	0.740770	0.291400	0.212980	Uiso ? Fe

Fe71	1.0	0.244120	0.791270	0.213110	Uiso ? Fe

Fe72	1.0	0.740730	0.791030	0.211040	Uiso ? Fe

La73	1.0	0.475740	0.917730	0.214270	Uiso ? La

La74	1.0	0.977640	0.916290	0.213830	Uiso ? La

La75	1.0	0.475610	0.418660	0.212830	Uiso ? La

La76	1.0	0.975750	0.417150	0.214300	Uiso ? La

O77	1.0	0.237150	0.166870	0.224100	Uiso ? O

O78	1.0	0.733760	0.167280	0.224020	Uiso ? O

O79	1.0	0.236970	0.667380	0.223830	Uiso ? O

O80	1.0	0.734870	0.661350	0.221310	Uiso ? O

O81	1.0	0.102120	0.021140	0.250260	Uiso ? O

O82	1.0	0.599640	0.025880	0.252210	Uiso ? O

O83	1.0	0.103520	0.312250	0.252330	Uiso ? O

O84	1.0	0.592870	0.312430	0.250230	Uiso ? O

O85	1.0	0.104050	0.521500	0.250330	Uiso ? O

O86	1.0	0.606950	0.525400	0.250550	Uiso ? O

O87	1.0	0.104360	0.812140	0.252170	Uiso ? O

O88	1.0	0.599520	0.807560	0.250160	Uiso ? O

O89	1.0	0.355500	0.812690	0.263070	Uiso ? O

O90	1.0	0.845260	0.807260	0.260540	Uiso ? O

O91	1.0	0.349360	0.312000	0.263060	Uiso ? O

O92	1.0	0.844980	0.312170	0.263100	Uiso ? O

O93	1.0	0.348510	0.025880	0.265420	Uiso ? O

O94	1.0	0.845310	0.026000	0.263150	Uiso ? O

O95	1.0	0.354660	0.525980	0.260510	Uiso ? O

O96	1.0	0.845010	0.521410	0.260540	Uiso ? O

O97	1.0	0.488100	0.175830	0.286810	Uiso ? O

O98	1.0	0.985420	0.166410	0.286230	Uiso ? O

O99	1.0	0.489130	0.671840	0.286450	Uiso ? O

O100	1.0	0.985630	0.666820	0.283930	Uiso ? O

La101	1.0	0.223610	0.420200	0.297160	Uiso ? La

La102	1.0	0.718020	0.413450	0.296780	Uiso ? La

La103	1.0	0.218290	0.917740	0.296790	Uiso ? La

La104	1.0	0.724050	0.914640	0.295450	Uiso ? La

Fe105	1.0	0.495680	0.035560	0.304740	Uiso ? Fe

Fe106	1.0	0.991300	0.041700	0.302290	Uiso ? Fe

Fe107	1.0	0.489520	0.295970	0.299710	Uiso ? Fe

Fe108	1.0	0.992860	0.297450	0.304660	Uiso ? Fe

Fe109	1.0	0.495630	0.792590	0.299330	Uiso ? Fe

Fe110	1.0	0.985750	0.791870	0.302110	Uiso ? Fe

Fe111	1.0	0.489270	0.537080	0.302020	Uiso ? Fe

Fe112	1.0	0.992420	0.541870	0.302280	Uiso ? Fe

La113	1.0	0.264470	0.169180	0.299950	Uiso ? La

La114	1.0	0.762190	0.167520	0.299190	Uiso ? La

La115	1.0	0.260590	0.669610	0.298100	Uiso ? La

La116	1.0	0.758010	0.665120	0.295890	Uiso ? La

O117	1.0	0.495830	0.415540	0.307410	Uiso ? O

O118	1.0	0.999630	0.420510	0.309830	Uiso ? O

O119	1.0	0.503130	0.914680	0.309790	Uiso ? O

O120	1.0	0.992920	0.917120	0.307440	Uiso ? O

O121	1.0	0.134740	0.271170	0.341290	Uiso ? O

O122	1.0	0.636000	0.275710	0.335810	Uiso ? O

O123	1.0	0.139830	0.067330	0.338640	Uiso ? O

O124	1.0	0.634760	0.068230	0.340790	Uiso ? O

O125	1.0	0.139330	0.568180	0.338380	Uiso ? O

O126	1.0	0.629620	0.572370	0.341280	Uiso ? O

O127	1.0	0.133360	0.770740	0.340940	Uiso ? O

O128	1.0	0.629570	0.770990	0.335500	Uiso ? O

O129	1.0	0.385480	0.765300	0.346680	Uiso ? O

O130	1.0	0.874190	0.760740	0.346740	Uiso ? O

O131	1.0	0.376750	0.572620	0.346570	Uiso ? O

O132	1.0	0.887720	0.578250	0.349310	Uiso ? O

O133	1.0	0.378660	0.077980	0.353720	Uiso ? O

O134	1.0	0.882230	0.073370	0.354050	Uiso ? O

O135	1.0	0.386180	0.270470	0.348800	Uiso ? O

O136	1.0	0.880630	0.261070	0.349660	Uiso ? O

O137	1.0	0.252260	0.421790	0.375130	Uiso ? O

O138	1.0	0.748080	0.416400	0.382730	Uiso ? O

O139	1.0	0.258660	0.917190	0.375020	Uiso ? O

O140	1.0	0.748940	0.916490	0.370360	Uiso ? O

La141	1.0	0.019250	0.172730	0.392430	Uiso ? La

La142	1.0	0.516380	0.168340	0.390340	Uiso ? La

La143	1.0	0.016280	0.673610	0.390820	Uiso ? La

La144	1.0	0.511610	0.667240	0.383850	Uiso ? La

Fe145	1.0	0.258370	0.307940	0.382340	Uiso ? Fe

Fe146	1.0	0.747650	0.302520	0.380380	Uiso ? Fe

Fe147	1.0	0.244950	0.030950	0.382990	Uiso ? Fe

Fe148	1.0	0.748150	0.031080	0.382700	Uiso ? Fe

Fe149	1.0	0.245980	0.536370	0.383250	Uiso ? Fe

Fe150	1.0	0.756230	0.525820	0.372390	Uiso ? Fe

Fe151	1.0	0.258390	0.803030	0.380240	Uiso ? Fe

Fe152	1.0	0.741220	0.801060	0.377980	Uiso ? Fe

O153	1.0	0.266250	0.307670	0.417170	Uiso ? O

H154	1.0	0.327160	0.346260	0.437350	Uiso ? H

H155	1.0	0.159800	0.311100	0.430970	Uiso ? H
