# Supplementary material for: Hydroxyl-rich nanocavities on perovskite enable nearly barrierless intramolecular hydrogen transfer for nitrate electroreduction to ammonia
Source: Nat Commun. 2026 Jun 29;17:8062. doi: 10.1038/s41467-026-74405-1 (PMC13454618; doi:10.1038/s41467-026-74405-1)
Supplement: Supplementary file 5 [file 41467_2026_74405_MOESM4_ESM.docx]

LF

_cell_angle_alpha 90.000

_cell_angle_beta	90.000

_cell_angle_gamma 90.000

_cell_length_a	11.130000

_cell_length_b	7.853000

_cell_length_c	33.545300

loop_

_symmetry_equiv_pos_as_xyz

x,y,z

loop_

_atom_site_label

_atom_site_fract_x

_atom_site_fract_y

_atom_site_fract_z

O	0.10252000	0.04390000	0.07102684

O	0.60252000	0.04390000	0.07102684

O	0.10252000	0.62350000	0.07102684

O	0.60252000	0.62350000	0.07102684

O	0.35252000	0.04390000	0.08079737

O	0.85252000	0.04390000	0.08079737

O	0.35252000	0.62350000	0.08079737

O	0.85252000	0.62350000	0.08079737

O	0.48703000	0.33370000	0.10465479

O	0.98703000	0.33370000	0.10465479

La	0.22803000	0.83370000	0.11701138

La	0.72803000	0.83370000	0.11701138

Fe	0.49221000	0.08370000	0.11731230

Fe	0.99221000	0.08370000	0.11731230

Fe	0.49221000	0.58370000	0.11731230

Fe	0.99221000	0.58370000	0.11731230

La	0.25638000	0.33370000	0.11760381

La	0.75638000	0.33370000	0.11760381

O	0.49738000	0.83370000	0.12996040

O	0.99738000	0.83370000	0.12996040

O	0.13189000	0.54390000	0.15381781

O	0.63189000	0.54390000	0.15381781

O	0.13189000	0.12350000	0.15381781

O	0.63189000	0.12350000	0.15381781

O	0.38189000	0.54390000	0.16358835

O	0.88189000	0.54390000	0.16358835

O	0.38189000	0.12350000	0.16358835

O	0.88189000	0.12350000	0.16358835

O	0.24738000	0.83370000	0.18744577

O	0.74738000	0.83370000	0.18744577

La	0.00638000	0.33370000	0.19980235

La	0.50638000	0.33370000	0.19980235

Fe	0.24221000	0.08370000	0.20010327

Fe	0.74221000	0.08370000	0.20010327

Fe	0.24221000	0.58370000	0.20010327

Fe	0.74221000	0.58370000	0.20010327

La	0.47803000	0.83370000	0.20039479

La	0.97803000	0.83370000	0.20039479

O	0.23703000	0.33370000	0.21275137

O	0.73703000	0.33370000	0.21275137

O	0.59765763	0.04149914	0.23641306

O	0.11109486	0.05124162	0.23511614

O	0.60227789	0.61048060	0.23805885

O	0.10405913	0.62424193	0.23210971

O	0.35920721	0.05280667	0.24380154

O	0.86373886	0.62167959	0.24272664

O	0.36046550	0.63399179	0.24465874

O	0.85441325	0.04287668	0.24709742

O	0.49238612	0.34404853	0.26874601

O	0.98813022	0.33336264	0.26994344

La	0.23409561	0.83559632	0.27872673

La	0.72500612	0.82876757	0.27514451

La	0.77686025	0.35750088	0.27862615

La	0.27275953	0.33996420	0.28030557

Fe	0.49043949	0.07009245	0.28085387

Fe	0.49177485	0.60672683	0.28300203

Fe	1.00073711	0.07382066	0.28680519

Fe	1.00048516	0.60699170	0.28059471

O	0.49954764	0.83745041	0.29137658

O	1.01337309	0.84468040	0.29255406

O	0.14420758	0.12672177	0.31419491

O	0.63517596	0.13522610	0.31454647

O	0.62826984	0.55570693	0.31726376

O	0.14566210	0.56179731	0.31080118

O	0.88565730	0.57720002	0.32288637

O	0.38108225	0.53480317	0.32620010

O	0.38113466	0.13168942	0.32614214

O	0.88467837	0.12462058	0.32885342

O	0.27997601	0.82554797	0.35740345

Fe	0.74142603	0.04987234	0.35022012

Fe	0.25008630	0.04752228	0.35555657

Fe	0.75161541	0.63192429	0.34993794

Fe	0.24167261	0.60832636	0.35539961

O	0.77848646	0.85128071	0.35983656

La	0.01678259	0.35205814	0.35523912

La	0.51785579	0.33183825	0.35677955

O	0.11553611	0.08986617	0.39016095

O	0.69377519	0.48021684	0.38672010

O	0.60757343	0.09902208	0.39085464

O	0.18082550	0.47791701	0.39075664

H	0.77571051	0.43399690	0.40211791

O	0.45956839	0.39257982	0.42966308

H	0.37227231	0.36812096	0.43654731

O	-0.12105972	0.35303593	0.41318137

H	0.15296669	1.08232983	0.41691584

H	0.64595109	0.07250220	0.41689369

H	0.95070760	1.00799880	0.42703223

H	0.43180968	1.02242566	0.39810605

H	0.51511195	0.36768900	0.45253531

H	-0.14070762	0.24304891	0.42600374

O	0.90975981	1.04882320	0.45054613

O	0.35353215	1.07279437	0.40552103

H	0.62317159	0.60313583	0.42639922

H	0.55562532	-0.11520261	0.47205760

H	0.45132258	-0.01512145	0.48901850

H	1.02840553	0.11593507	0.48364102

H	0.86025069	0.95314194	0.46011677

O	0.53977533	-0.02749791	0.49244639

O	0.60094049	0.68746480	0.44776568

H	0.32413130	0.00824318	0.43057038

O	0.10595276	0.13406431	0.49588070

H	0.03975618	0.56583649	0.50759798

H	0.52754875	0.64647815	0.46251538

H	0.82856240	0.68990367	0.48870115

H	1.09769951	0.13792226	0.52480086

H	0.19017654	0.27867909	0.47536275

H	0.61180657	0.16097382	0.49230760

O	0.10800955	0.62060477	0.52249137

O	0.79618149	0.80543279	0.48826367

H	0.90912678	0.42963114	0.45698448

O	0.62679378	0.28438191	0.49143838

O	0.90924245	0.50170632	0.48167834

H	0.68917341	0.31664986	0.51105708

H	0.07126783	0.71762669	0.53642797

H	0.19972206	0.43377295	0.44178259

O	0.23861358	0.38082180	0.46630573

H	0.22790130	0.01462288	0.48516429

O	0.30094105	0.95961364	0.47284868

H	0.72005538	0.78650929	0.47328979

H	0.86659078	0.44062744	0.50523708

H	0.30519583	0.83356628	0.47897264

H	0.89769701	0.88286919	0.52606362

H	0.81355995	0.46255060	0.56683247

H	0.90310250	0.80544546	0.56847057

H	0.33806091	0.68178875	0.52098171

O	0.80498316	0.35299631	0.55340122

O	0.36930908	0.64265455	0.49597755

O	0.94896640	0.88384177	0.55078676

O	0.75504602	-0.01146800	0.62196923

H	0.67769723	0.06286682	0.62092941

H	0.84942537	0.27983843	0.57068991

H	0.81837398	0.05908855	0.61088922

H	0.30153398	0.56583479	0.48858383

O	0.32460682	-0.08598238	0.56422543

H	0.48800905	0.26696871	0.53375406

H	0.36993980	0.01922533	0.55714093

H	0.77422462	0.77742714	0.61033780

H	0.85680556	0.62438290	0.62568902

O	0.81525260	0.67066435	0.60107818

H	1.17446295	1.05521129	0.58178230

O	0.43716918	0.23221078	0.55552883

H	0.36955646	0.32195388	0.55418230

H	0.33975389	-0.13695092	0.59012072

O	0.25394884	0.45460601	0.57075598

H	0.19025186	0.51362209	0.55641225

O	1.14368024	1.16895500	0.57516583

H	1.29941012	0.55071177	0.58403290

H	1.19899624	1.26159305	0.58087407

O	0.37916320	0.69950014	0.62980002

H	0.65210248	0.55505262	0.61346262

H	0.51498179	0.19886045	0.59951905

H	0.21372772	0.64626277	0.66747985

H	0.99864321	0.20584742	0.60101673

O	0.13723110	0.62712348	0.68225398

H	0.45902606	0.63849520	0.62800237

O	0.91550003	0.23095890	0.61141089

H	0.40297264	0.80877653	0.64371279

O	0.60072440	0.51226541	0.63420839

H	0.59535373	0.38504353	0.63019647

H	0.90557475	0.31088245	0.63320174

O	0.56175486	0.17459002	0.62379834

H	0.16791600	0.59533800	0.71027432

H	0.50390392	0.10761763	0.64214126

H	0.88335602	-0.05578938	0.69788265

H	0.31783879	0.06930801	0.67804684

O	0.39671392	0.00937080	0.67002367

H	0.77522652	-0.04906728	0.66739154

H	1.12877974	0.08985386	0.69326167

H	-0.00041097	0.51212885	0.66695349

O	-0.08912731	0.48600172	0.66757817

H	0.63856722	0.61057609	0.68228673

H	1.08008069	0.82364633	0.69707631

O	1.19262603	0.17267039	0.68606075

O	0.79428618	-0.06344565	0.69690265

H	0.42152705	-0.04452673	0.69564512

O	1.04244546	0.94150522	0.70354484

H	0.69545060	0.79097936	0.70053319

H	0.31849804	0.20323646	0.77516655

H	0.88222241	0.49089039	0.69619234

O	0.63693272	0.69592985	0.70482370

O	0.23992194	0.58867150	0.75558151

H	0.21526267	0.19750512	0.71429306

H	0.24875454	0.46754156	0.75842987

H	1.03514979	0.93596071	0.73365211

O	0.24438703	0.24968594	0.76504513

H	0.43984310	0.06474765	0.82602069

H	0.76817644	0.08494434	0.73949416

H	0.16706694	0.62753714	0.77062557

H	0.18443043	0.24388252	0.78756102

H	0.74782104	0.59219864	0.72864884

H	0.48972958	0.77525301	0.73200210

O	0.80632654	0.51446290	0.74196548

O	0.44259265	0.09836097	0.79811272

O	0.75361116	0.15668430	0.76398644

H	0.42568350	0.98530913	0.78447355

H	0.36300090	0.75634919	0.74574295

H	0.75903437	0.40881449	0.74607187

H	0.66839826	0.13904370	0.77006837

O	-0.04717015	0.96748318	0.78442319

O	0.42424579	0.84626244	0.74220104

H	0.87094254	1.00119700	0.78512573

H	0.02723254	0.14827378	0.81558606

H	-0.03652792	0.85549400	0.79810127

O	0.07147631	0.24783544	0.82475094

H	-0.07238843	0.59959656	0.77682158

O	-0.01289285	0.63526413	0.79679344

H	0.07271443	0.21270035	0.85366521

H	-0.00250656	0.53851299	0.81440216

LSF

_cell_angle_alpha 90.000

_cell_angle_beta	90.000

_cell_angle_gamma 90.000

_cell_length_a	10.983500

_cell_length_b	19.024000

_cell_length_c	35.481400

loop_

_symmetry_equiv_pos_as_xyz

x,y,z

loop_

_atom_site_label

_atom_site_fract_x

_atom_site_fract_y

_atom_site_fract_z

H	0.58961616	0.37265607	0.11720798

O	0.65415924	0.35323209	0.10206649

H	0.80031861	0.57888661	0.10468301

H	0.66466863	0.30504548	0.11175157

H	0.13635556	0.88465193	0.14795368

O	0.81539774	0.58433895	0.13233734

H	0.76604168	0.41233224	0.13797960

O	0.86767539	0.80783645	0.12259174

H	0.82209362	0.48602331	0.14554834

H	0.84482499	0.81545587	0.09736427

H	0.92608624	0.62680539	0.15419616

H	0.16504116	0.22690358	0.14212044

H	0.95428026	0.81253989	0.12534466

H	0.96411356	0.69523816	0.17778848

H	0.73561103	0.59050248	0.14630126

O	0.81030951	0.43951659	0.15675061

H	0.25742593	0.00688858	0.12754653

O	0.08205745	0.84446884	0.14974565

H	0.72613496	0.77244712	0.15580088

O	0.67185432	0.20859607	0.14754836

H	0.66087282	0.79276500	0.19563949

H	0.37413633	0.25220757	0.15547594

O	0.19786554	0.97865289	0.11242992

H	0.58162720	0.22203882	0.15124068

O	0.99856508	0.65440308	0.16404343

O	0.23437334	0.20089636	0.15290847

H	0.45908375	0.35936459	0.16530761

H	0.67452518	0.15675613	0.14480189

O	0.45620048	0.26487310	0.16505648

H	0.32939216	0.10606676	0.16009766

O	0.64815043	0.77036924	0.16997196

H	0.94250248	0.36951974	0.16535759

H	0.12600659	0.81156921	0.16375573

O	0.46316269	0.41173539	0.16660355

O	0.37360592	0.06097158	0.15645275

H	0.84800127	0.91301472	0.14831054

H	0.66059868	0.67997039	0.17752025

H	0.46064810	0.06336471	0.15687525

H	0.37883521	0.43224771	0.16210808

H	0.14683416	1.01338371	0.09806290

O	0.97406354	0.32282861	0.17010408

H	0.18448408	0.55696723	0.15148490

H	0.20701744	0.19371525	0.18022783

O	0.65414847	0.62448683	0.18116286

O	0.63309739	0.06043726	0.16633305

H	0.56084866	0.61757224	0.18882175

H	0.67311490	0.01893591	0.15562198

H	0.22414170	0.50869841	0.18684263

H	0.72918953	0.44037675	0.19542516

H	0.44977220	0.23866493	0.19007358

O	0.25760572	0.53932730	0.16755991

H	0.35512435	0.59698596	0.18988228

H	0.74283144	0.22280844	0.19203072

O	0.79123574	0.93793390	0.16554776

H	0.12809788	0.38764997	0.18798236

H	0.05822538	0.63769439	0.22687917

H	0.58822230	0.43181168	0.20059363

H	0.90620939	0.29808151	0.18157261

O	0.41601186	0.62527019	0.20651984

O	0.16564175	0.42411294	0.20338421

H	0.65771790	0.05947822	0.19363493

O	0.66446671	0.43002745	0.21496785

H	0.39002227	0.67189275	0.20844322

H	0.75030812	0.90575563	0.18235931

H	0.93782004	0.90186396	0.21673479

O	0.78862679	0.24953619	0.21290307

O	0.13044509	0.77436140	0.25687866

H	0.26071526	0.77689266	0.22281414

O	1.02685370	0.64159641	0.25175525

O	0.32778974	0.77198467	0.20456763

H	0.14890260	0.00985898	0.20596578

O	0.97231463	0.90626150	0.24134490

H	0.08533220	0.72714084	0.25548062

H	0.93912188	0.65084054	0.24912790

H	0.22155896	0.39796427	0.22052438

H	0.74293468	0.63153697	0.21792889

H	0.73182126	0.28162654	0.22360085

O	0.19542731	0.16836567	0.23028921

O	0.45565007	0.19457243	0.23118222

H	0.18570345	0.76843225	0.27946214

H	0.58413584	0.13453546	0.23482616

H	0.74067656	0.62720691	0.33973529

H	0.19081089	0.11739649	0.23677267

H	1.01074883	0.86420004	0.25073453

H	0.36745039	0.18969779	0.23867262

H	0.43933511	0.57568800	0.31605214

O	0.68456110	0.82648766	0.23767806

H	0.62961057	0.84763907	0.25725743

H	0.71447504	0.41032627	0.25849891

H	0.35551772	0.81863653	0.19719670

O	0.15520756	0.01229353	0.23362105

H	0.89116642	0.02597117	0.30973403

H	0.11917227	0.18767551	0.24045904

H	0.49150014	0.23559323	0.24455941

H	0.08709249	0.98949224	0.24687555

O	0.64343645	0.10063018	0.24459377

H	0.55899660	0.02736489	0.32856824

O	0.79353909	0.65997752	0.23508462

H	0.08187186	0.55688376	0.28425471

O	0.56176885	0.90974955	0.28535906

H	0.62245658	0.08085007	0.27085861

H	0.74281509	0.70395409	0.24025545

H	0.03493175	0.02037574	0.32088665

H	0.48720503	0.93292749	0.27682576

H	0.76048612	0.84654675	0.23960299

O	0.97037414	0.04989905	0.30992165

O	0.25885999	0.76436950	0.32242290

O	0.69820828	0.60403566	0.31967984

H	0.88884936	0.20360440	0.24712470

H	1.00298406	0.29559706	0.27411061

H	0.83791670	0.37657921	0.27338494

O	0.36387692	0.58531932	0.30260926

O	0.98283305	0.34597453	0.27254875

H	0.37786978	0.60392520	0.27786513

H	0.74102728	0.61863750	0.29819506

O	0.56310414	0.27666829	0.28057558

O	0.75392826	0.38887235	0.28142290

O	0.13256650	0.53367896	0.30530216

H	0.64244465	0.29745551	0.28488175

O	0.64897061	0.02760553	0.32386461

H	0.26100696	0.81288323	0.33440509

H	0.22319954	0.54148539	0.30228776

O	0.96346631	0.19576808	0.26117657

O	0.29001400	0.33979401	0.25687115

H	0.61289576	0.94975818	0.29697536

H	0.34403268	0.75309702	0.31856403

H	0.25253607	0.35821517	0.27926000

H	0.37684651	0.34824865	0.25875195

H	0.57044274	0.23356842	0.29841799

H	1.02740993	0.37023272	0.25307632

H	0.66876752	0.11116706	0.32794654

H	0.95038958	0.15607845	0.27863426

O	0.66838760	0.16564095	0.32763827

H	0.74649286	0.42055806	0.32540205

H	0.74774079	0.17452635	0.31486544

H	0.07726779	0.60302460	0.34321222

O	0.74292095	0.40706847	0.35348292

H	0.69353051	0.22994667	0.36468898

O	0.81432945	0.71417292	0.38028515

H	0.72383601	0.31065693	0.36854104

H	0.89360821	0.72472468	0.37205257

H	0.90878050	0.44381923	0.35237603

H	0.09850009	0.10097240	0.35690746

H	0.81682267	0.68165451	0.40116889

H	1.03094276	0.48172880	0.33438895

H	0.69817951	0.44405758	0.36656590

O	0.70191798	0.26860551	0.38260915

H	0.05403241	0.77049482	0.33758136

O	0.04105346	0.62504132	0.36525605

H	0.52817186	0.66831584	0.36163057

O	0.78390771	-0.03040091	0.36483821

H	0.57081546	0.69014077	0.40374994

H	0.31314009	0.08053512	0.35860532

O	-0.02552846	0.78141431	0.32690067

H	0.71818764	-0.01168645	0.34734147

O	0.98874347	0.46280813	0.35669987

O	0.15577937	0.06628985	0.36687341

H	0.36119457	0.19168406	0.37078139

H	0.73898219	0.92272653	0.37338576

O	0.40380430	0.09144977	0.35601977

H	0.19912747	0.93279759	0.35566387

O	0.52509879	0.65611293	0.38865619

H	0.82373487	0.22856940	0.39932108

H	0.11387802	0.64822940	0.37405843

O	0.33316397	0.23411367	0.38383803

O	0.24410927	0.88989745	0.36092895

H	0.73549405	0.79412562	0.38736737

H	0.56305302	0.53794208	0.39569724

H	0.32807686	0.90357534	0.36986413

H	0.25330247	0.22655988	0.39758668

H	-0.01424466	0.77066148	0.29980386

O	0.89766611	0.19459586	0.39665482

H	0.07064748	0.38921087	0.38997130

H	0.13975884	0.06385033	0.39307478

O	0.57840647	0.49284130	0.40801706

O	0.45084139	0.91396610	0.39658862

H	0.50566853	0.46951371	0.41621997

H	0.51497661	0.88244008	0.38713250

H	0.16938898	0.33107654	0.39603594

H	0.56663946	0.31410255	0.40577773

H	0.42638867	0.07539977	0.38123747

O	0.14607951	0.38031312	0.40214019

H	0.25190868	0.45161388	0.40599267

H	0.77370906	0.02801718	0.39668570

O	0.69012267	0.83321952	0.39787566

H	0.04133942	0.20642474	0.41324059

H	0.98167782	0.50229582	0.40629801

H	0.42922659	0.29217584	0.40823623

H	0.85276852	0.14884908	0.40058952

O	0.29590356	0.47288940	0.42918620

O	0.12792863	0.21409017	0.41939610

H	0.40480116	0.89023112	0.41827926

O	0.49298148	0.31948378	0.42145018

H	0.28205339	0.52738296	0.42703752

H	0.67123056	0.82258860	0.42318035

H	0.86067638	0.83885857	0.42093339

O	0.76516666	0.06582947	0.41996549

O	0.05472122	0.61208498	0.46339156

H	0.23444270	0.66034845	0.43414830

O	0.93393305	0.50028778	0.43122249

O	0.28844170	0.62605049	0.42289376

H	0.19462567	0.82748446	0.41921877

O	0.91540289	0.82304358	0.44124322

H	0.02343793	0.56254172	0.45858518

H	0.85889820	0.52723369	0.42878107

H	0.14263486	0.21754252	0.44718146

H	0.63082164	0.53385026	0.45649543

H	0.70266616	0.09807339	0.41205550

O	0.13726217	0.04378423	0.44681301

O	0.36761672	0.00416423	0.47213842

H	0.13741954	0.60382292	0.47567606

H	0.72399730	1.01973280	0.46229390

H	0.53566295	0.37348484	0.46636409

H	0.09401793	1.00003961	0.45517995

H	0.97173719	0.78502745	0.43300494

H	0.29361797	0.02350524	0.45859431

H	0.28368735	0.42174939	0.46181936

O	0.77563420	0.65945053	0.46044120

H	0.70140339	0.69056689	0.45978833

H	0.53128316	0.25441438	0.45724535

H	0.36477880	0.64760742	0.41365705

O	0.22765879	0.84690192	0.44366546

H	0.87817227	0.95591326	0.48429033

H	0.10701511	0.07815375	0.46551478

H	0.43276470	0.03933062	0.47214549

H	0.25981414	0.81275066	0.46064805

O	0.72541759	0.97773887	0.48108534

H	0.44447662	0.93987039	0.48004661

O	0.67505036	0.54358021	0.48061901

H	-0.15312242	0.42844075	0.45488256

O	0.57625530	0.74987040	0.46835928

H	0.58850565	0.92891640	0.48139195

H	0.72325183	0.58859336	0.47587205

H	0.95111609	0.89579340	0.46688071

H	0.48696687	0.74577616	0.46595163

H	0.83649425	0.67240284	0.48090673

O	0.96240559	0.93729878	0.48413885

O	0.33220776	0.75741938	0.48741529

O	0.56226098	0.40160555	0.48794594

H	0.87255303	0.09835348	0.46146490

H	0.85647042	0.20666717	0.47394178

O	0.29108361	0.39225551	0.48822416

H	0.65711071	0.24470762	0.47619714

O	0.82741569	0.25547557	0.48207755

H	0.25401115	0.34388780	0.48437142

H	0.65214930	0.40557827	0.49019747

O	0.54350839	0.09936148	0.49327662

O	0.57194165	0.23437535	0.47971615

O	0.84341087	0.40539767	0.48080993

H	0.55487775	0.14845620	0.48534145

O	0.51064153	0.90964048	0.49194668

H	0.36673548	0.77374528	0.51399885

H	-0.16713490	0.35312749	0.47684474

O	0.91724218	0.12445861	0.47948218

O	0.19489463	0.25384770	0.49012755

H	0.59915875	0.75442393	0.49464473

H	0.31944210	0.70797162	0.49639528

H	0.26085312	0.22849580	0.50228413

O	0.93236801	0.67619642	0.51180066

H	0.14601050	0.27792021	0.51127787

H	0.35258080	-0.00020070	0.51796034

H	0.56983538	0.09089131	0.51941919

H	0.85051754	0.25716287	0.50910351

H	0.59632303	0.54013290	0.52038022

H	0.16859405	0.90389299	0.50791315

O	0.27626660	0.61385713	0.51749883

H	0.98679024	0.63548593	0.50778266

H	0.89196273	0.10549531	0.50525728

H	0.69027783	0.98921627	0.50621585

H	0.52867334	0.27514067	0.52514584

O	0.05375527	0.54354264	0.53516042

O	0.54287581	0.52365234	0.54302301

O	0.09566289	0.03062418	0.54149883

O	0.35894811	0.00671944	0.54564679

H	0.38998337	0.66162160	0.54172195

O	0.36097420	0.19677312	0.53725332

O	0.47888931	0.66654473	0.55024179

La	0.42045826	0.41388389	0.54264523

H	0.50201889	0.61749813	0.55977568

H	0.34672755	0.14695100	0.54336200

O	0.17952320	0.90595077	0.53548125

H	0.71205268	0.77995914	0.55799998

O	0.86156728	0.80349175	0.54705195

O	0.63454955	0.78514806	0.54549205

O	0.53320578	0.29536160	0.54982376

O	0.08888150	0.73961109	0.56203706

O	0.73326607	0.41835097	0.55604023

H	0.08992788	0.10896170	0.55541638

O	0.73359787	0.94638772	0.55839177

Fe	0.19367365	0.57041217	0.54854473

La	0.93283479	0.44259530	0.55192803

Fe	0.94443929	0.72897205	0.55185246

O	0.06076778	0.32432850	0.55481270

La	0.53500576	0.89653394	0.56229792

O	0.39547500	0.80147895	0.55525719

O	0.86286756	0.25408646	0.55783265

H	0.15789865	0.32224329	0.56252308

La	0.95197099	0.92526137	0.55575630

O	0.61504936	0.06311297	0.56520362

O	0.26394000	0.49342000	0.56463000

O	0.10265000	0.15920000	0.56539000

Fe	0.21243000	0.99399000	0.56575000

O	0.79492000	0.54604000	0.56587000

O	0.87198000	0.04431000	0.57263000

O	0.29650000	0.32953000	0.57587000

Fe	0.65985000	0.48814000	0.57969000

Fe	0.41333000	0.24166000	0.57984000

Fe	0.99097000	0.25345000	0.58212000

Fe	0.51349000	0.74337000	0.58245000

O	0.88058000	0.67515000	0.58632000

H	0.90775000	0.08072000	0.58962000

O	0.17407000	0.61675000	0.58984000

Fe	0.71095000	0.01811000	0.59145000

La	0.23099000	0.81975000	0.59569000

La	0.70131000	0.31475000	0.59776000

Fe	0.75147000	0.61737000	0.59902000

O	0.13209000	0.94461000	0.59981000

O	0.23403000	0.06346000	0.60015000

H	0.31206000	0.34255000	0.60227000

O	0.55937000	0.43041000	0.60292000

O	0.63062000	0.69072000	0.60627000

O	0.02764000	0.44631000	0.60783000

Fe	0.18712000	0.15235000	0.60889000

O	0.13856000	0.24328000	0.61088000

O	0.34804000	0.18054000	0.61203000

O	0.63866000	0.55274000	0.61489000

O	0.37736000	0.71597000	0.61658000

O	0.37992000	0.92390000	0.61686000

O	0.48459000	0.29276000	0.61727000

O	0.61876000	0.95844000	0.61742000

O	0.00826000	0.82378000	0.61825000

O	0.91588000	0.18409000	0.61969000

O	0.54043000	0.82512000	0.62210000

O	0.88266000	0.93414000	0.62787000

O	0.91412000	0.31806000	0.62800000

Sr	0.20445000	0.49005000	0.62978000

O	0.80840000	0.42821000	0.63030000

O	0.67231000	0.20680000	0.63100000

O	0.73684000	0.07435000	0.63168000

Fe	0.27801000	0.65881000	0.64479000

O	0.31517000	0.39331000	0.64569000

Fe	0.95020000	0.40617000	0.64674000

O	0.16554000	0.73913000	0.64814000

O	0.84242000	0.61255000	0.64936000

Fe	0.00689000	0.89382000	0.64940000

O	0.36625000	0.58086000	0.64951000

La	0.94886000	0.71195000	0.64992000

Fe	0.78819000	0.15856000	0.65132000

Fe	0.49769000	0.90019000	0.65177000

Fe	0.49910000	0.39417000	0.65563000

Sr	0.26492000	0.00834000	0.65856000

O	0.13000000	0.12736000	0.65900000

O	0.15140000	0.60369000	0.66401000

O	0.65329000	0.34915000	0.66717000

La	0.55125000	0.74399000	0.67000000

O	0.17659000	0.87401000	0.67023000

La	0.50511000	0.21470000	0.67054000

La	0.01356000	0.24862000	0.67290000

O	0.10704000	0.35853000	0.67567000

Sr	0.75070000	0.50836000	0.67570000

Sr	0.75277000	0.98264000	0.67709000

O	0.00125000	0.48342000	0.67743000

O	0.03474000	0.97701000	0.67969000

O	0.76763000	0.73983000	0.68271000

O	0.88832000	0.10327000	0.68564000

Fe	0.24759000	0.79259000	0.68616000

O	0.63697000	0.86325000	0.68617000

Fe	0.26906000	0.34025000	0.68639000

O	0.39685000	0.83418000	0.68821000

O	0.46407000	0.98008000	0.68967000

O	0.33583000	0.70514000	0.68982000

O	0.92928000	0.85445000	0.69081000

Fe	0.46167000	0.56542000	0.69099000

O	0.79826000	0.23054000	0.69099000

O	0.50065000	0.46942000	0.69109000

Fe	0.04304000	0.07221000	0.69198000

O	0.42934000	0.32752000	0.69447000

O	0.90405000	0.36443000	0.69526000

O	0.38384000	0.11627000	0.69532000

Fe	0.04705000	0.56983000	0.69638000

O	0.60480000	0.61475000	0.69890000

O	0.23980000	0.24741000	0.69935000

O	0.62027000	0.11599000	0.70205000

Fe	0.75212000	0.32295000	0.70544000

Fe	0.77359000	0.82365000	0.71118000

O	0.97442000	0.65116000	0.71266000

Fe	0.49736000	0.06378000	0.71565000

O	0.25495000	0.41724000	0.72907000

O	0.15478000	0.06200000	0.73080000

O	0.15957000	0.78061000	0.73095000

O	0.60682000	0.27634000	0.73456000

Fe	0.01141000	0.73861000	0.73637000

Fe	0.26888000	0.50340000	0.73670000

O	0.38105000	0.57076000	0.73879000

La	0.74595000	0.65682000	0.73972000

Sr	0.06071000	0.90862000	0.74105000

O	0.13413000	0.55496000	0.74257000

Sr	0.02697000	0.42241000	0.74393000

O	0.99611000	0.19376000	0.74423000

O	0.59317000	0.76833000	0.74480000

Sr	0.48059000	0.88992000	0.74616000

O	0.63069000	0.00489000	0.74733000

Sr	0.80350000	0.13319000	0.74942000

O	0.83078000	0.51963000	0.75102000

Sr	0.28124000	0.67490000	0.75245000

La	0.26186000	0.17265000	0.75305000

Sr	0.45649000	0.38268000	0.75450000

O	0.86722000	0.75551000	0.75516000

O	0.76919000	0.37810000	0.75603000

O	0.76657000	0.87812000	0.75963000

O	0.39617000	0.06676000	0.75979000

Fe	0.26851000	0.01372000	0.76111000

Fe	0.75885000	0.96789000	0.76474000

O	0.27488000	0.92033000	0.76588000

O	0.88196000	0.01261000	0.76771000

Fe	0.54568000	0.22803000	0.76901000

Fe	0.73077000	0.45751000	0.76905000

O	0.40699000	0.25375000	0.78038000

Fe	0.04713000	0.23839000	0.78287000

O	0.59326000	0.47958000	0.78413000

Fe	0.53882000	0.73032000	0.78496000

O	0.03978000	0.32358000	0.79083000

O	0.62669000	0.17305000	0.79385000

O	0.63166000	0.66562000	0.79659000

O	0.41061000	0.75094000	0.80230000

O	0.13051000	0.18414000	0.80976000

SNC-LSF

_cell_angle_alpha 90.000

_cell_angle_beta	90.000

_cell_angle_gamma 90.000

_cell_length_a	10.981700

_cell_length_b	19.024000

_cell_length_c	35.047500

loop_

_symmetry_equiv_pos_as_xyz

x,y,z

loop_

_atom_site_label

_atom_site_fract_x

_atom_site_fract_y

_atom_site_fract_z

O	0.45999000	0.15523000	0.04337000

Fe	0.96574000	0.65663000	0.08861000

Fe	0.21574000	0.90663000	0.08861000

Fe	0.71574000	0.40663000	0.08861000

Fe	0.46574000	0.65663000	0.08861000

Fe	0.96574000	0.15663000	0.08861000

Fe	0.21574000	0.40663000	0.08861000

Fe	0.71574000	0.90663000	0.08861000

Fe	0.46574000	0.15663000	0.08861000

La	0.71574000	0.57329000	0.12061000

O	0.09074000	0.69829000	0.12061000

O	0.59074000	0.19829000	0.12061000

O	0.46574000	0.07329000	0.12061000

O	0.96574000	0.57329000	0.12061000

O	0.34074000	0.19829000	0.12061000

O	0.84074000	0.69829000	0.12061000

O	0.34074000	0.94829000	0.12061000

O	0.84074000	0.44829000	0.12061000

O	0.21574000	0.82329000	0.12061000

O	0.71574000	0.32329000	0.12061000

O	0.09074000	0.94829000	0.12061000

O	0.59074000	0.44829000	0.12061000

O	0.09074000	0.19829000	0.12061000

O	0.59074000	0.69829000	0.12061000

O	0.46574000	0.57329000	0.12061000

O	0.96574000	0.07329000	0.12061000

O	0.34074000	0.69829000	0.12061000

O	0.84074000	0.19829000	0.12061000

O	0.34074000	0.44829000	0.12061000

O	0.84074000	0.94829000	0.12061000

O	0.21574000	0.32329000	0.12061000

O	0.71574000	0.82329000	0.12061000

O	0.09074000	0.44829000	0.12061000

O	0.59074000	0.94829000	0.12061000

Sr	0.46574000	0.32329000	0.12061000

Sr	0.96574000	0.82329000	0.12061000

Sr	0.21574000	0.57329000	0.12061000

Sr	0.71574000	0.07329000	0.12061000

Sr	0.46574000	0.82329000	0.12061000

Sr	0.96574000	0.32329000	0.12061000

La	0.21574000	0.07329000	0.12061000

Fe	0.21574000	0.23996000	0.15259000

Fe	0.71574000	0.73996000	0.15259000

Fe	0.46574000	0.98996000	0.15259000

Fe	0.96574000	0.48996000	0.15259000

Fe	0.21574000	0.73996000	0.15259000

Fe	0.71574000	0.23996000	0.15259000

Fe	0.46574000	0.48996000	0.15259000

Fe	0.96574000	0.98996000	0.15259000

H	0.55995000	0.82425000	0.17242000

O	0.21590000	0.15663000	0.18333000

O	0.21574000	0.65663000	0.18459000

O	0.71574000	0.15663000	0.18459000

O	0.09074000	0.78163000	0.18459000

O	0.59074000	0.28163000	0.18459000

O	0.09074000	0.53163000	0.18459000

O	0.59074000	0.03163000	0.18459000

O	0.46574000	0.90663000	0.18459000

O	0.96574000	0.40663000	0.18459000

O	0.34074000	0.03163000	0.18459000

O	0.84074000	0.53163000	0.18459000

La	0.46574000	0.65663000	0.18459000

La	0.96574000	0.15663000	0.18459000

O	0.09074000	0.03163000	0.18459000

O	0.59074000	0.53163000	0.18459000

O	0.46574000	0.40663000	0.18459000

O	0.96574000	0.90663000	0.18459000

O	0.34074000	0.53163000	0.18459000

O	0.84074000	0.03163000	0.18459000

La	0.46574000	0.15663000	0.18459000

La	0.96574000	0.65663000	0.18459000

Sr	0.21574000	0.90663000	0.18459000

Sr	0.71574000	0.40663000	0.18459000

O	0.34074000	0.78163000	0.18459000

O	0.84074000	0.28163000	0.18459000

Sr	0.21574000	0.40663000	0.18459000

Sr	0.71574000	0.90663000	0.18459000

O	0.34074000	0.28163000	0.18459000

O	0.84074000	0.78163000	0.18459000

O	0.09074000	0.28163000	0.18459000

O	0.59074000	0.78163000	0.18459000

O	0.86704000	0.59474000	0.19705000

La	0.97181000	0.83170000	0.19861000

O	0.02995000	0.73277000	0.20165000

La	0.50125000	0.32245000	0.20751000

Fe	0.96452000	0.26785000	0.20893000

O	0.88541000	0.92675000	0.21203000

O	0.17083000	0.51828000	0.21257000

La	0.39910000	0.48144000	0.21571000

La	0.39547000	0.82942000	0.21625000

Fe	0.30707000	0.70784000	0.21856000

La	0.04468000	0.98529000	0.21879000

O	0.56017000	0.07928000	0.21925000

Fe	0.31475000	0.60153000	0.21969000

Fe	0.81785000	0.09638000	0.22164000

La	0.52022000	0.97850000	0.22387000

O	0.52775000	0.90066000	0.22483000

O	0.80689000	0.80415000	0.22520000

O	0.53776000	0.42120000	0.22631000

Fe	0.64202000	0.52995000	0.22674000

O	0.94621523	0.67760929	0.27631241

Fe	0.31437291	0.04484473	0.31198102

La	0.88590729	0.46957730	0.29668788

O	0.36481243	0.27051683	0.28254356

O	0.67026141	0.96494029	0.27968819

O	0.30217861	0.39557395	0.24852947

O	0.17591727	0.86475010	0.24601347

O	0.07579868	0.34996613	0.29804345

O	0.74805005	0.33649277	0.28209590

O	-0.04855611	0.23416472	0.25276066

O	0.46251782	0.72630812	0.24271109

Fe	0.06303073	0.68579364	0.31046546

La	0.27228337	0.37642789	0.31013761

O	0.32239094	0.12945776	0.32772204

O	0.24689456	1.01329086	0.35372250

O	-0.29790081	0.31348753	0.36186769

O	0.50623335	0.14891541	0.28734880

O	0.74703369	0.73693709	0.34105415

O	0.76123703	0.12186084	0.26497661

O	0.71307642	0.68652243	0.26774958

O	0.97114836	0.09614344	0.24499765

O	0.25326250	0.64638040	0.25865294

Fe	0.07328732	0.09996358	0.28555966

O	0.53218353	0.58995190	0.25185235

La	0.72827416	0.83390850	0.40030829

O	0.19144569	0.04156867	0.27562624

O	0.95411548	0.65655078	0.35099731

O	0.47233619	0.23572072	0.35545465

Fe	0.40995955	0.20769436	0.31185452

Fe	0.54671543	0.67497707	0.27731104

Fe	0.02442989	0.26435462	0.29244751

O	0.75573196	0.47914069	0.24337867

O	0.51465146	0.72444774	0.31641471

H	0.73985590	0.72412887	0.25193944

Fe	0.77149256	0.28251712	0.31662175

O	0.05766966	0.54244788	0.29068626

O	0.43438301	-0.01087247	0.30080655

O	0.11932242	0.76300939	0.30289228

La	0.39169259	0.50291456	0.40194667

O	0.94463919	0.48529055	0.36257853

Fe	0.18976719	0.84372700	0.29427765

Fe	0.57748570	0.96037262	0.31950279

O	0.45373056	0.37983277	0.35343828

O	0.70124983	0.19845162	0.32861196

Fe	0.64950163	0.13046689	0.29972564

H	0.91882817	0.71548863	0.38767583

Fe	0.82935150	0.67066197	0.31013146

O	0.22859550	0.46346991	0.36474919

O	0.70338037	0.44838346	0.34903033

Fe	0.17693845	0.60091327	0.29421073

H	0.44829030	0.63590164	0.35814633

O	0.89276047	0.75021855	0.40757066

O	0.34551169	0.84320683	0.30336006

O	0.56438727	0.87903062	0.34322414

O	-0.08393084	0.26504750	0.33001667

H	-0.32076587	0.04661633	0.36087765

H	0.65937857	0.73140088	0.33635182

H	0.99811372	0.47042747	0.38395743

O	0.61897644	0.05187789	0.33966905

Fe	0.45620162	0.80595303	0.33156509

O	0.81341101	0.93253956	0.37370900

O	0.00167048	0.04819345	0.32203330

H	0.40313061	0.15519379	0.41641758

O	0.44502902	0.63091612	0.38600733

H	-0.09813558	0.22092737	0.36974647

O	0.10062520	0.90162692	0.31860163

O	1.11073262	0.18670167	0.29765383

H	0.20733244	0.50764175	0.35080358

H	0.93025206	0.74005582	0.43192047

H	0.83776661	0.95714569	0.35028686

Fe	0.61645385	0.24058263	0.37493163

O	0.98711955	0.58278704	0.41631953

Fe	0.59919241	0.38746566	0.37265328

O	0.66675727	0.18353621	0.41123675

Fe	0.09622731	0.98105559	0.34137978

O	0.27149519	0.57899540	0.32860137

H	0.49402635	0.91321611	0.43813437

H	0.69566362	0.49224984	0.36498764

O	0.80265911	0.57841517	0.32039782

O	0.41140845	0.10322420	0.40977400

H	0.45767882	0.09737331	0.38561699

H	1.03555358	0.16228607	0.38946512

H	0.71158473	0.64747504	0.49167941

H	-0.04031980	0.61971709	0.43442047

H	0.12857971	0.57357979	0.42498785

O	0.55192682	0.30320603	0.40880555

H	0.58800055	-0.05613923	0.46239310

Fe	0.80113120	0.14430546	0.42236872

O	0.04717448	-0.02752678	0.39086225

Fe	0.89362155	0.57563100	0.37175668

O	0.24455265	0.28372189	0.35560510

O	0.58146394	0.92367303	0.43701631

O	0.21643735	0.57880155	0.43432500

O	0.57419790	0.44716019	0.41151026

O	0.62995727	0.66374562	0.48183984

Fe	0.90601370	0.98162350	0.41388336

O	0.82596899	0.05676526	0.40951634

H	0.62294262	0.71284775	0.45314286

H	0.57184693	0.23429536	0.53493555

O	0.40780824	0.77998531	0.37626395

O	0.92478018	0.19819039	0.39551290

Fe	0.38851439	0.30701303	0.38746212

O	0.85571285	0.90163002	0.44479424

H	0.57822434	0.62320175	0.47405973

O	0.11846147	0.13023913	0.38705870

O	0.33721764	0.36917349	0.42243531

H	0.23325244	0.63118814	0.43198131

H	0.28992066	0.88182318	0.44429821

H	0.34338128	0.87069464	0.40608834

H	-0.00727255	1.03030923	0.47614864

O	0.59880557	0.74780435	0.43053345

H	0.83121507	0.33062966	0.41401232

O	0.02152333	1.01049617	0.45304500

La	0.64493342	0.36822764	0.47203094

O	0.75477233	0.58136285	0.39641965

Fe	0.23402526	0.30094287	0.43997848

O	0.32907867	0.90964599	0.42348867

O	0.34197604	0.24237549	0.42684486

H	0.76623246	0.55566160	0.42153298

H	1.16599911	0.28677011	0.37098572

H	0.91011376	0.88637721	0.46549098

Fe	0.45439663	0.71601297	0.40712039

O	0.82376523	0.32721418	0.44240718

O	0.25014090	0.08372666	0.48849951

H	0.32805978	0.01998278	0.57652021

La	0.20280309	0.03361944	0.42118592

O	0.08284018	0.28767866	0.42278127

O	0.77182081	0.47919449	0.46586349

H	0.85087583	0.47711081	0.48204468

Fe	0.94924254	0.25739529	0.44677069

O	0.35465074	0.68863926	0.44571529

H	0.70189228	0.25503059	0.52743747

O	0.27349927	-0.00619628	0.55688044

O	0.67427135	0.48515027	0.49302174

Fe	0.12745224	0.28095437	0.51540121

Fe	0.32215224	0.02390933	0.51277687

H	0.34341874	0.72976322	0.46186459

O	0.63022777	0.24711543	0.51590860

H	0.20851477	0.15969919	0.51853046

O	0.19799253	0.20304448	0.53317566

O	-0.00568312	0.75032085	0.48063054

O	0.87892159	0.17670084	0.46182638

O	0.21363565	-0.17071271	0.47497664

O	0.23364028	0.32659455	0.48782083

H	0.17819613	0.15339144	0.37018021

H	0.25712594	0.62071480	0.49427897

O	0.45123760	0.99309374	0.49720804

H	0.26570025	0.55240216	0.52256686

Fe	0.10110888	0.80142346	0.49797328

O	0.30705858	0.59382226	0.51225200

O	0.79227945	0.35057230	0.53124618

O	0.47852800	0.54286941	0.46380469

H	0.54836347	0.51552883	0.47440539

H	0.42142696	0.56477485	0.48311732

H	0.34127695	0.71676882	0.57421209

H	0.29931291	0.65220716	0.55902743

O	-0.01979027	0.28774943	0.49473007

O	0.22449140	0.48039886	0.54546158

Fe	0.93702035	0.35997151	0.52532434

O	0.96713431	0.43840449	0.51196634

O	0.12602177	0.81566431	0.54657770

O	0.26234904	0.69267911	0.57371695

H	0.21092194	0.49237021	0.57193202

H	0.18255928	0.70123459	0.61444520

H	1.03201780	0.55227727	0.58028512

H	0.14242110	0.46982057	0.53528730

H	0.94646807	0.51827342	0.55263201

O	1.06073649	0.33120648	0.55412970

O	0.87986257	0.62219665	0.50463129

O	0.47946228	0.39672877	0.51766491

H	0.17325287	0.77349125	0.55587984

O	0.95239897	0.56130643	0.56865998

H	0.51561718	0.71886203	0.54273985

H	0.91948340	0.66548717	0.49466108

H	0.39167798	0.38394392	0.51333055

O	0.14623922	0.70534660	0.64016287

O	0.29771447	0.77025102	0.70242023

H	0.90185593	0.62050182	0.53221818

H	0.49319566	0.90557553	0.55465720

H	0.59022512	1.02319737	0.62006257

H	0.18854231	0.74214123	0.65239601

H	0.28817351	0.34985264	0.57567759

H	-0.08882983	0.39232641	0.63907273

O	0.62715535	0.97797364	0.61645496

H	0.60152967	0.60321255	0.67527126

H	0.49398976	0.40766977	0.54520782

H	0.36327794	0.74894907	0.71853698

O	1.04334731	0.94208353	0.59204718

H	1.12141662	0.95018008	0.57869342

H	0.16550032	0.18328297	0.57857539

O	0.48238218	0.73380256	0.56738820

O	0.13789795	0.50889133	0.61996433

H	0.33114860	0.80515493	0.68247360

H	0.44093713	0.27640070	0.58901970

H	0.43438637	0.13075472	0.59292121

H	0.55988047	0.91595303	0.58948716

H	0.57102579	0.46124087	0.60373263

O	0.50203985	0.88082637	0.57846992

O	0.48473741	0.23213220	0.58298446

H	0.71725409	0.98293436	0.61965387

O	0.60464058	0.45590048	0.57755060

H	1.01289868	0.90276045	0.57728621

O	0.35213142	0.35309614	0.59496745

H	0.69470836	0.39929068	0.64097741

H	0.58455932	0.49707710	0.56233484

H	0.18543616	0.53707426	0.63738119

H	0.51833912	0.78099332	0.57412633

H	0.22771668	0.63691203	0.65692475

O	-0.02146709	0.40214167	0.65597923

O	0.52349635	0.60534313	0.68967699

H	0.98040128	0.71926663	0.65303518

H	0.10989040	0.46443327	0.63330596

O	0.17175986	0.16827574	0.60640200

H	0.52662653	0.57185242	0.71130739

H	0.94301462	0.41903704	0.68037230

H	0.91379689	0.98621453	0.61490183

H	0.20236167	0.21103818	0.61752740

H	0.30154749	0.11909645	0.61027118

O	0.38425069	0.09747932	0.60865622

H	0.74464884	0.41479358	0.59922759

H	0.29718037	0.34462370	0.61736028

H	0.02892978	0.15677468	0.63070008

O	0.26197983	0.59791262	0.67261851

H	0.55932031	0.23162682	0.59810550

O	0.89824676	0.72201537	0.66440108

O	0.75568270	0.38343919	0.62305863

O	0.67854627	0.83355336	0.68008062

H	0.75476947	0.29336987	0.62838821

H	0.50804980	0.37450042	0.68603695

H	0.34826086	0.60461203	0.67645831

H	-0.07462711	0.10332948	0.63927718

O	0.57649202	0.40408313	0.67761474

H	0.91495561	0.72105913	0.69214416

H	0.36581014	0.87270926	0.62009431

H	0.61549711	0.84744983	0.66225261

H	0.74029475	0.80443256	0.66761161

O	-0.05202673	0.15228208	0.64604368

O	0.85096560	1.01977978	0.62207369

H	0.83680180	0.64724367	0.66131164

O	0.07232162	0.94652864	0.70108955

H	0.81285466	0.21674198	0.63011526

H	0.42181203	0.09449258	0.65815963

H	0.84382124	1.00786033	0.65033526

O	0.73790189	0.24139091	0.62835423

H	0.13096571	0.33851376	0.65949769

H	0.12039314	0.93385964	0.67923073

H	0.23988245	0.32040214	0.68528397

H	0.58154773	0.43622532	0.70037461

H	0.37190714	0.30929514	0.72885616

O	0.35753035	0.86705785	0.64747502

H	0.37435760	-0.08662364	0.66048740

O	0.20428404	0.31190814	0.66035760

H	-0.01368815	0.94765155	0.69409638

O	0.46727721	0.09391738	0.68344228

O	0.77684405	0.60688046	0.66458139

H	0.52173681	0.13436064	0.68136829

O	0.09848806	0.58885615	0.73198610

H	0.14231063	0.59165320	0.70764144

H	0.56360148	0.76051168	0.75996216

H	0.78151492	0.57941732	0.64235413

H	0.68772360	1.10917393	0.70922729

O	0.40775092	0.30954880	0.70340996

H	-0.04680070	0.17470342	0.69254143

H	0.47534378	0.98073013	0.70373290

O	0.40391729	0.95158851	0.70807340

H	0.65028508	0.22744259	0.66786233

O	0.62632612	0.80222604	0.75885492

H	0.40321084	0.67207089	0.76428201

O	0.30120955	0.62224617	0.78229237

H	0.79434927	1.01240041	0.70658999

H	0.84352801	0.53952743	0.69803627

O	0.46179177	0.70252120	0.74884665

H	0.63822321	0.80718126	0.73072765

H	0.23126108	0.61711135	0.76585568

H	0.05651463	0.63548620	0.73758611

O	0.82672651	0.97209551	0.69234395

H	0.45988525	0.26675958	0.70373060

H	0.38884562	0.11679654	0.73135927

H	0.00269883	0.28724069	0.80105891

O	-0.11152903	0.50428806	0.71442312

H	0.00713188	0.14536921	0.72903091

H	0.11856049	0.41226503	0.76586188

O	0.61119372	0.21398890	0.69445477

H	0.49025871	0.48412193	0.75742891

H	-0.02874578	0.52939891	0.71590173

O	-0.05427835	0.18110392	0.72055691

H	0.42865847	0.91865819	0.72798349

H	0.32210235	0.57392828	0.79062978

H	0.49230894	0.67057521	0.72852805

O	0.74555179	1.09158868	0.72889814

O	0.55721071	0.50066954	0.74132413

O	0.05564237	0.28047647	0.77888334

H	0.81293121	0.12486838	0.72703486

H	0.90079821	0.71970258	0.75689219

O	0.35466524	0.13783210	0.75608034

H	0.00565598	0.25618783	0.75835546

H	0.76939943	0.93004355	0.69112406

H	0.67480265	0.22596662	0.71245963

H	0.72937390	0.75207123	0.78910455

H	0.20343059	0.27512596	0.77370036

H	0.41912205	0.13679061	0.77510898

O	0.17915338	0.43849878	0.75104730

H	0.15715264	0.48673174	0.74915788

O	0.96515406	0.70955925	0.73782110

H	0.62383128	0.51050326	0.75923552

H	0.82459315	0.53147544	0.75728687

H	0.31506650	0.22309115	0.76544246

O	0.38759607	0.46451007	0.79105303

H	0.81676866	0.59445500	0.78478710

O	0.78067257	0.71687436	0.80209991

H	0.42156432	0.42005100	0.80157906

H	1.02048787	0.75084578	0.73897176

O	0.29400222	0.27213240	0.77308362

O	0.71797284	0.91274854	0.80007068

H	0.69853784	0.06178304	0.78326704

H	0.31064740	0.45005632	0.77908908

H	0.79987524	0.89805225	0.79686360

O	0.79294284	0.54390224	0.78396164

H	0.12050531	0.86738942	0.73651658

H	0.67852278	0.87584853	0.78546689

O	0.67634295	0.24458895	0.78438395

H	0.82399935	0.74319881	0.82227069

H	0.18057185	1.09441619	0.75861903

H	0.69352206	0.99289885	0.80919589

O	0.68482877	0.04478749	0.80943302

H	0.04090149	0.83829789	0.79247143

H	0.59341933	0.17644430	0.80347719

H	0.55115593	0.10417795	0.81616658

H	0.62267863	0.28130438	0.79441362

H	0.42554676	0.31392848	0.79515093

O	0.96315489	0.85967778	0.80241254

H	0.78463579	0.47803689	0.82747827

O	0.51719776	0.15326313	0.81362442

H	0.45705596	0.30454649	0.83590725

H	0.75645916	0.26469204	0.79009691

O	0.12439307	0.81764199	0.74498154

H	0.10424643	1.03808029	0.73496236

O	0.09644027	1.08231634	0.75006710

O	0.48743424	0.33262971	0.81446553

H	0.20118461	0.79972974	0.73138055

H	0.81388366	0.06795802	0.85567538

H	0.98864063	0.88038597	0.82688995

H	0.14543651	0.32347674	0.88452988

O	0.77682759	0.43891040	0.84709503

H	0.40968889	0.19381401	0.84563460

O	0.16073619	0.28536968	0.90159630

H	0.69041179	0.43179609	0.84446697

H	0.02676682	1.00522737	0.80668550

H	0.23564650	0.26404088	0.89303842

O	0.86268067	0.05770043	0.87877729

H	0.91520679	0.01913233	0.87135335

H	0.31736388	0.24181470	0.82975409

O	0.35870783	0.23255771	0.85440859

O	0.06288861	1.00005426	0.83210427

O	0.89558046	0.31133771	0.83133901

H	0.14036015	1.02439805	0.83198236

H	0.87966678	0.16122370	0.88256303

H	-0.00872099	0.21184432	0.89271948

H	0.65452264	1.06250744	1.03068558

H	0.86333658	0.35569950	0.84046225

H	0.90027693	0.28212197	0.85431461

H	0.79617121	1.04482409	1.03639580

O	0.73252679	1.07673403	1.04238989

O	0.90665875	0.20914501	0.88514983
