# Supplementary material for: Hydroxyl-rich nanocavities on perovskite enable nearly barrierless intramolecular hydrogen transfer for nitrate electroreduction to ammonia
Source: Nat Commun. 2026 Jun 29;17:8062. doi: 10.1038/s41467-026-74405-1 (PMC13454618; doi:10.1038/s41467-026-74405-1)
Supplement: Supplementary file 6 [file 41467_2026_74405_MOESM5_ESM.docx]

Initial State

O	5.0523001600	2.9530955200	2.0001393900

Fe	10.6072052900	12.4917291200	3.5853705100

Fe	2.3695802900	17.2477291200	3.5853705100

Fe	7.8613302900	7.7357291200	3.5853705100

Fe	5.1154552900	12.4917291200	3.5853705100

Fe	10.6072052900	2.9797291200	3.5853705100

Fe	2.3695802900	7.7357291200	3.5853705100

Fe	7.8613302900	17.2477291200	3.5853705100

Fe	5.1154552900	2.9797291200	3.5853705100

La	7.8613302900	10.9062689600	4.7067623400

O	0.9966427900	13.2842689600	4.7067623400

O	6.4883927900	3.7722689600	4.7067623400

O	5.1154552900	1.3942689600	4.7067623400

O	10.6072052900	10.9062689600	4.7067623400

O	3.7425177900	3.7722689600	4.7067623400

O	9.2342677900	13.2842689600	4.7067623400

O	3.7425177900	18.0402689600	4.7067623400

O	9.2342677900	8.5282689600	4.7067623400

O	2.3695802900	15.6622689600	4.7067623400

O	7.8613302900	6.1502689600	4.7067623400

O	0.9966427900	18.0402689600	4.7067623400

O	6.4883927900	8.5282689600	4.7067623400

O	0.9966427900	3.7722689600	4.7067623400

O	6.4883927900	13.2842689600	4.7067623400

O	5.1154552900	10.9062689600	4.7067623400

O	10.6072052900	1.3942689600	4.7067623400

O	3.7425177900	13.2842689600	4.7067623400

O	9.2342677900	3.7722689600	4.7067623400

O	3.7425177900	8.5282689600	4.7067623400

O	9.2342677900	18.0402689600	4.7067623400

O	2.3695802900	6.1502689600	4.7067623400

O	7.8613302900	15.6622689600	4.7067623400

O	0.9966427900	8.5282689600	4.7067623400

O	6.4883927900	18.0402689600	4.7067623400

Sr	5.1154552900	6.1502689600	4.7067623400

Sr	10.6072052900	15.6622689600	4.7067623400

Sr	2.3695802900	10.9062689600	4.7067623400

Sr	7.8613302900	1.3942689600	4.7067623400

Sr	5.1154552900	15.6622689600	4.7067623400

Sr	10.6072052900	6.1502689600	4.7067623400

La	2.3695802900	1.3942689600	4.7067623400

H	4.0055726200	3.4083398400	5.5124485100

Fe	2.3695802900	4.5649990400	5.8274838700

Fe	7.8613302900	14.0769990400	5.8274838700

Fe	5.1154552900	18.8329990400	5.8274838700

Fe	10.6072052900	9.3209990400	5.8274838700

Fe	2.3695802900	14.0769990400	5.8274838700

Fe	7.8613302900	4.5649990400	5.8274838700

Fe	5.1154552900	9.3209990400	5.8274838700

Fe	10.6072052900	18.8329990400	5.8274838700

H	6.1502108200	15.6805320000	6.5222373800

O	0.8937274000	5.2374974400	6.7937040200

O	6.4350129800	14.8023841600	6.8774900200

O	2.3713376500	2.9797291200	6.9043015400

O	7.8506762900	12.4987680000	6.9398268100

O	6.4883927900	5.3577291200	6.9485405500

O	0.9966427900	10.1137291200	6.9485405500

O	6.4883927900	0.6017291200	6.9485405500

O	5.1154552900	17.2477291200	6.9485405500

O	10.6072052900	7.7357291200	6.9485405500

O	3.7425177900	0.6017291200	6.9485405500

O	9.2342677900	10.1137291200	6.9485405500

La	5.1154552900	12.4917291200	6.9485405500

La	10.6072052900	2.9797291200	6.9485405500

O	0.9966427900	0.6017291200	6.9485405500

O	6.4883927900	10.1137291200	6.9485405500

O	5.1154552900	7.7357291200	6.9485405500

O	10.6072052900	17.2477291200	6.9485405500

O	3.7425177900	10.1137291200	6.9485405500

O	9.2342677900	0.6017291200	6.9485405500

La	5.1154552900	2.9797291200	6.9485405500

La	10.6072052900	12.4917291200	6.9485405500

Sr	2.3695802900	17.2477291200	6.9485405500

Sr	7.8613302900	7.7357291200	6.9485405500

O	3.7425177900	14.8697291200	6.9485405500

O	9.2342677900	5.3577291200	6.9485405500

Sr	2.3695802900	7.7357291200	6.9485405500

Sr	7.8613302900	17.2477291200	6.9485405500

O	3.7425177900	5.3577291200	6.9485405500

O	9.2342677900	14.8697291200	6.9485405500

O	7.8613302900	2.9797291200	6.9485405500

O	0.9966427900	14.8697291200	6.9485405500

O	2.4450369400	12.3616049600	7.1137665400

H	8.1754583900	13.0875608000	7.7435021200

Fe	10.5895218500	6.1038504000	7.8641539600

H	2.5653062600	3.3647748800	7.8725325600

H	7.8269519400	3.2513918400	7.9331936200

Fe	7.8798924100	10.7810910400	8.0427857100

O	9.1029051300	14.0459899200	8.2415261000

O	10.0182700200	17.5970097600	8.2840893900

Fe	2.0490817600	10.5316864000	8.3551399200

Fe	8.5451630000	1.1806294400	8.6983273800

La	4.7660701600	15.6556105600	8.8648939400

La	5.2362737900	6.4953643200	8.8933811800

La	10.8969500200	15.4689851200	8.8963974800

La	5.3354548000	18.9610305600	8.9423122100

Fe	2.6017714800	12.8533753600	8.9493502300

O	4.8802985600	2.1430536000	8.9868863600

O	3.1007518900	3.9583236800	9.0147033100

O	0.4881067400	11.1630929600	9.0193953300

O	0.8069577500	13.4155345600	9.1001650300

O	6.8354713900	16.6496145600	9.1078733400

O	1.6993671200	8.9568796800	9.1785887300

O	4.4958760600	13.3234584000	9.1799293000

O	6.9200443400	8.1626276800	9.1842861800

O	9.1132296200	11.4183950400	9.2617044400

O	3.1784052300	17.9961332800	9.2751102000

O	9.7547758600	6.8115432000	9.2891862500

O	0.3292853300	4.1320128000	9.3428092900

O	7.9736915000	2.8515073600	9.3485067400

O	2.3607934900	6.4147025600	9.4192221200

O	10.2518890600	1.5162128000	9.5237870500

La	10.2556234500	9.1992454400	9.5948375800

H	2.7726747400	17.1326339200	9.6886779000

O	2.0364507400	15.9341219200	9.7000727900

O	6.8283321200	12.0471382400	9.7493389600

La	0.8351853400	18.8269113600	9.7516849700

H	8.8702746000	13.9331776000	9.8602716200

O	3.0048659300	11.1824974400	9.9413764700

Fe	1.9500105900	4.8349496000	10.0238219000

O	7.7220271718	0.0766598623	9.8020575588

O	4.3895435563	7.9133750692	10.3261242568

La	5.4553494444	9.7931417096	10.1418157377

Fe	3.6488580690	2.6654040379	10.3611085432

O	1.8850443194	1.9004431523	10.4735239707

Fe	0.2138835444	2.7718837443	10.5111167968

Fe	11.2850991809	12.4157218126	10.6897270471

Fe	5.7803785664	13.2048322852	10.7060546419

O	9.3103846040	13.7381180704	10.7487383215

O	10.1556076752	17.1162334319	10.6376367908

O	5.8341858879	4.5225915066	10.3483739701

Fe	7.5730613700	4.0275960801	10.6723154118

Fe	10.0925419113	6.3683472390	11.0884420578

La	7.6718701830	17.0593981882	11.2343849063

Fe	8.3128303903	11.8529577014	10.9452556214

O	7.7599204148	9.9877774256	11.0137091551

La	2.3634766370	7.7982942128	11.4470588705

O	9.0435549594	4.9629566253	11.3752923838

O	4.0315456764	15.3464493008	11.3366660804

H	4.1827286952	0.6753011737	12.0943969752

O	6.5587265141	14.7228509103	11.6020065362

Fe	2.1798432579	15.0812728665	11.4012576865

H	8.5980803807	1.9108714888	11.7620459614

O	4.8007723627	1.2104577845	11.5167694499

O	11.0061014044	7.8836237646	11.3979051737

O	3.3495008234	4.3120897779	11.4136817551

O	0.9930992499	5.5268842786	11.6097532792

O	-0.8014992865	10.9885806311	11.5473283031

O	1.9154799216	13.1374116845	11.4766930536

Fe	2.9414362884	11.6499158677	11.9222278234

H	10.3733809796	16.9447676674	11.5857462460

O	5.7417512313	17.1828394010	12.7844832161

Fe	6.5200618350	1.1855348962	12.3096342417

O	4.9515419288	11.7725776664	11.7644752591

Fe	4.9548577736	4.8317669837	12.0203505226

O	7.8538486572	2.4794824346	11.9990018651

O	6.2389146504	5.9510432962	12.6646891261

O	1.6027976201	18.5371104860	11.9925378393

H	5.4987822592	11.7296779009	12.5960165306

O	10.9737672568	2.6416221477	12.4988068946

Fe	5.1840505377	15.4174504532	12.7208576536

H	0.9348576752	4.8262934251	12.3002650692

H	-0.8324831929	10.7499555471	12.4972629112

O	8.5036447671	8.9374894027	11.6005056205

O	5.9127630444	8.9721739445	13.1889705094

O	1.4261388578	15.7650622777	12.8747319630

O	7.9110973692	12.0993701180	12.9653721035

H	0.9179143245	2.4894720594	12.8193846596

O	10.1766216580	13.6023842313	13.4452977441

H	1.9213871882	0.3361652469	12.3503932681

H	-0.2186562593	14.3333218453	13.7363266457

O	7.8830904150	19.0188482329	12.7547704359

O	2.9204067756	9.9242920610	12.2730344868

Fe	1.3722165326	17.4005059361	13.5675733318

H	8.0005266733	11.4296446666	13.7002200494

Fe	7.1378346125	7.8453844755	12.5438740565

Fe	7.4907210458	5.7013336064	14.2244438078

O	9.2516201306	17.0254212998	13.1820814910

H	5.7135165835	17.5234511554	13.7686279870

Fe	9.9213510593	3.2294802103	13.9892525759

O	3.2402169578	1.2597699548	14.5053758967

O	2.7217156867	2.5223154769	13.9914389429

O	8.5407903174	4.3439367920	14.2759646366

O	8.3825923451	7.2990784906	13.8811808912

H	3.9347663318	3.6373271761	13.9524203382

O	3.3749032951	7.2092778229	13.7522815417

Fe	9.3905874290	18.6579876080	13.7121335193

Fe	8.4128631074	13.7302166429	13.7085776311

H	8.6255651675	16.0047682714	14.2156941398

La	4.0232255984	9.8232326329	14.4099030454

O	4.5290623664	14.8078315168	14.2940229441

O	4.6121269727	4.3752404816	13.8230182007

Fe	4.5822972240	6.0088272394	14.9474567565

O	9.2647871018	1.4088048387	14.4568718803

O	3.1466271320	5.5330364667	16.1197740078

O	2.7358821366	17.1754065616	14.8275672397

O	5.2818986254	11.9239093323	15.0622220266

O	6.2672329360	6.3307119113	15.4913125400

H	3.1892468598	16.3005792999	14.6540224830

O	11.1301446865	3.8963296367	15.1857077220

O	0.0862493173	18.3593969942	14.5390212292

Fe	4.2354117926	13.0301130103	14.3853432295

O	8.2341755205	15.1734134029	14.6382269653

O	7.6916048056	10.8598207688	15.5165760640

H	8.3059937973	11.2500456550	16.2595741237

H	4.0134501231	18.2760628007	15.3747126731

H	0.4460688872	18.9973755674	15.2990531898

Fe	2.4483133876	7.1518220206	15.3983694154

H	8.2988065690	1.6366493531	14.5335407763

H	8.6966673878	6.7705914900	18.6313545300

H	6.7531754294	10.9290666689	15.8968687637

O	3.0015098247	11.9025121510	13.8962630603

O	4.6867042503	2.2876739469	16.5390566189

La	2.3550119534	2.9521974943	16.3576414225

O	4.9036737819	18.7148593948	15.5372090768

O	2.2546586565	8.9468337918	15.8461596648

Fe	4.9597862396	20.4029077100	15.1650336528

O	6.2037901524	1.6733227851	14.1034356327

Fe	10.9587183610	5.3173674661	16.3822401784

O	8.2078343952	7.6774768102	18.5909428159

La	8.0006225571	8.4855741963	16.0118914322

O	0.5797919483	6.8690404465	15.5730300678

O	1.1465638934	4.6296013725	17.7570233042

O	1.0891458092	19.8444838559	16.4098256799

O	9.9634744458	9.5984548582	15.5439845276

O	9.1732217435	5.9718372119	16.7621711227

Fe	0.3812625728	8.7148596338	16.1157737203

O	5.7717826093	9.4172871768	16.2365701916

H	1.2377513357	4.8906559035	18.6850302076

H	5.3942177862	9.2504395350	17.1034036374

H	8.4780266935	5.3564305128	16.5808027406

O	2.4632386831	1.3162393168	18.4538989315

Fe	1.8598793881	19.0066625085	17.6588507198

O	8.5251493918	10.1414232615	18.0284059430

O	-0.1384282892	8.5385827045	18.0437969744

Fe	9.4423475157	9.0493344361	18.8127803564

O	9.5886670428	9.5403301783	20.3220266040

O	2.4534190030	17.3821946578	17.4375109040

H	2.7343813250	17.1649924335	16.4394680306

Transition State

O	5.0523001600	2.9530955200	2.0001393900

Fe	10.6072052900	12.4917291200	3.5853705100

Fe	2.3695802900	17.2477291200	3.5853705100

Fe	7.8613302900	7.7357291200	3.5853705100

Fe	5.1154552900	12.4917291200	3.5853705100

Fe	10.6072052900	2.9797291200	3.5853705100

Fe	2.3695802900	7.7357291200	3.5853705100

Fe	7.8613302900	17.2477291200	3.5853705100

Fe	5.1154552900	2.9797291200	3.5853705100

La	7.8613302900	10.9062689600	4.7067623400

O	0.9966427900	13.2842689600	4.7067623400

O	6.4883927900	3.7722689600	4.7067623400

O	5.1154552900	1.3942689600	4.7067623400

O	10.6072052900	10.9062689600	4.7067623400

O	3.7425177900	3.7722689600	4.7067623400

O	9.2342677900	13.2842689600	4.7067623400

O	3.7425177900	18.0402689600	4.7067623400

O	9.2342677900	8.5282689600	4.7067623400

O	2.3695802900	15.6622689600	4.7067623400

O	7.8613302900	6.1502689600	4.7067623400

O	0.9966427900	18.0402689600	4.7067623400

O	6.4883927900	8.5282689600	4.7067623400

O	0.9966427900	3.7722689600	4.7067623400

O	6.4883927900	13.2842689600	4.7067623400

O	5.1154552900	10.9062689600	4.7067623400

O	10.6072052900	1.3942689600	4.7067623400

O	3.7425177900	13.2842689600	4.7067623400

O	9.2342677900	3.7722689600	4.7067623400

O	3.7425177900	8.5282689600	4.7067623400

O	9.2342677900	18.0402689600	4.7067623400

O	2.3695802900	6.1502689600	4.7067623400

O	7.8613302900	15.6622689600	4.7067623400

O	0.9966427900	8.5282689600	4.7067623400

O	6.4883927900	18.0402689600	4.7067623400

Sr	5.1154552900	6.1502689600	4.7067623400

Sr	10.6072052900	15.6622689600	4.7067623400

Sr	2.3695802900	10.9062689600	4.7067623400

Sr	7.8613302900	1.3942689600	4.7067623400

Sr	5.1154552900	15.6622689600	4.7067623400

Sr	10.6072052900	6.1502689600	4.7067623400

La	2.3695802900	1.3942689600	4.7067623400

H	4.0055726200	3.4083398400	5.5124485100

Fe	2.3695802900	4.5649990400	5.8274838700

Fe	7.8613302900	14.0769990400	5.8274838700

Fe	5.1154552900	18.8329990400	5.8274838700

Fe	10.6072052900	9.3209990400	5.8274838700

Fe	2.3695802900	14.0769990400	5.8274838700

Fe	7.8613302900	4.5649990400	5.8274838700

Fe	5.1154552900	9.3209990400	5.8274838700

Fe	10.6072052900	18.8329990400	5.8274838700

H	6.1502108200	15.6805320000	6.5222373800

O	0.8937274000	5.2374974400	6.7937040200

O	6.4350129800	14.8023841600	6.8774900200

O	2.3713376500	2.9797291200	6.9043015400

O	7.8506762900	12.4987680000	6.9398268100

O	6.4883927900	5.3577291200	6.9485405500

O	0.9966427900	10.1137291200	6.9485405500

O	6.4883927900	0.6017291200	6.9485405500

O	5.1154552900	17.2477291200	6.9485405500

O	10.6072052900	7.7357291200	6.9485405500

O	3.7425177900	0.6017291200	6.9485405500

O	9.2342677900	10.1137291200	6.9485405500

La	5.1154552900	12.4917291200	6.9485405500

La	10.6072052900	2.9797291200	6.9485405500

O	0.9966427900	0.6017291200	6.9485405500

O	6.4883927900	10.1137291200	6.9485405500

O	5.1154552900	7.7357291200	6.9485405500

O	10.6072052900	17.2477291200	6.9485405500

O	3.7425177900	10.1137291200	6.9485405500

O	9.2342677900	0.6017291200	6.9485405500

La	5.1154552900	2.9797291200	6.9485405500

La	10.6072052900	12.4917291200	6.9485405500

Sr	2.3695802900	17.2477291200	6.9485405500

Sr	7.8613302900	7.7357291200	6.9485405500

O	3.7425177900	14.8697291200	6.9485405500

O	9.2342677900	5.3577291200	6.9485405500

Sr	2.3695802900	7.7357291200	6.9485405500

Sr	7.8613302900	17.2477291200	6.9485405500

O	3.7425177900	5.3577291200	6.9485405500

O	9.2342677900	14.8697291200	6.9485405500

O	7.8613302900	2.9797291200	6.9485405500

O	0.9966427900	14.8697291200	6.9485405500

O	2.4450369400	12.3616049600	7.1137665400

H	8.1754583900	13.0875608000	7.7435021200

Fe	10.5895218500	6.1038504000	7.8641539600

H	2.5653062600	3.3647748800	7.8725325600

H	7.8269519400	3.2513918400	7.9331936200

Fe	7.8798924100	10.7810910400	8.0427857100

O	9.1029051300	14.0459899200	8.2415261000

O	10.0182700200	17.5970097600	8.2840893900

Fe	2.0490817600	10.5316864000	8.3551399200

Fe	8.5451630000	1.1806294400	8.6983273800

La	4.7660701600	15.6556105600	8.8648939400

La	5.2362737900	6.4953643200	8.8933811800

La	10.8969500200	15.4689851200	8.8963974800

La	5.3354548000	18.9610305600	8.9423122100

Fe	2.6017714800	12.8533753600	8.9493502300

O	4.8802985600	2.1430536000	8.9868863600

O	3.1007518900	3.9583236800	9.0147033100

O	0.4881067400	11.1630929600	9.0193953300

O	0.8069577500	13.4155345600	9.1001650300

O	6.8354713900	16.6496145600	9.1078733400

O	1.6993671200	8.9568796800	9.1785887300

O	4.4958760600	13.3234584000	9.1799293000

O	6.9200443400	8.1626276800	9.1842861800

O	9.1132296200	11.4183950400	9.2617044400

O	3.1784052300	17.9961332800	9.2751102000

O	9.7547758600	6.8115432000	9.2891862500

O	0.3292853300	4.1320128000	9.3428092900

O	7.9736915000	2.8515073600	9.3485067400

O	2.3607934900	6.4147025600	9.4192221200

O	10.2518890600	1.5162128000	9.5237870500

La	10.2556234500	9.1992454400	9.5948375800

H	2.7726747400	17.1326339200	9.6886779000

O	2.0364507400	15.9341219200	9.7000727900

O	6.8283321200	12.0471382400	9.7493389600

La	0.8351853400	18.8269113600	9.7516849700

H	8.8702746000	13.9331776000	9.8602716200

O	3.0048659300	11.1824974400	9.9413764700

Fe	1.9500105900	4.8349496000	10.0238219000

O	7.6007391467	0.0650422678	9.9013650525

O	4.4420365682	7.7039834350	10.7156424727

La	5.4565763494	9.6035352461	10.4367201219

Fe	3.5902511105	2.6346378731	10.3792305880

O	1.8020017200	1.9536516921	10.7755630619

Fe	0.0805838156	2.7089064963	10.6891759264

Fe	11.1525565263	12.3555417529	10.5855438715

Fe	5.5944078746	13.0371092385	10.7629400956

O	9.3613352142	13.7166833860	10.7035714518

O	10.2395666213	17.1875710917	10.6952537216

O	5.8358106732	4.5793331650	10.5085000187

Fe	7.5600126618	3.9318892633	10.7344704785

Fe	10.1782969275	6.2661998773	10.9395960642

La	7.8480690302	17.1157150430	11.0837344687

Fe	8.3056990581	11.7400934418	11.0318887569

O	7.9720623571	9.8606412429	10.9937545453

La	2.3841755044	7.6361396768	11.6639209677

O	9.0189653997	5.0210526959	11.3276061511

O	4.0103215768	15.0850244464	11.2847528225

H	3.8401937357	0.2331596293	11.5029624315

O	6.4705102561	14.3509252623	11.9181285798

Fe	2.1472957512	15.0412529344	11.4415885912

H	7.8724487135	1.5679510871	11.1028058658

O	4.5445518692	0.8798873018	11.2655861126

O	11.0216025201	7.7724062495	11.4030364619

O	3.4507271361	4.2637451033	11.5097132580

O	0.8753657896	5.4209506961	11.6179670368

O	-0.4064952312	10.9519144994	11.6357629141

O	1.5451825278	13.2099623846	11.6806083757

Fe	2.6483628269	11.7144296926	12.2000984070

H	10.5694419762	16.8012140756	11.5529451997

O	5.6894490437	17.3155136102	12.3930450004

Fe	6.2195070558	0.9591790363	12.4092716192

O	4.7351272329	12.0113561932	12.1469982378

Fe	5.0032606693	4.7697331827	12.1155791380

O	7.5812200900	2.2013382035	11.7928132416

O	6.3648629256	5.9051930669	12.6971820247

O	1.6794135786	18.5319598124	12.0685029480

H	5.1944449264	12.2249783496	12.9983275759

O	10.9239158818	3.1291417126	12.6626204036

Fe	5.0498781985	15.5214093827	12.7470441146

H	0.5199839188	4.6637124925	12.1549749730

H	-0.2683868616	10.8577320145	12.6039569503

O	8.5308278035	8.7426568510	11.6619803395

O	5.8169338237	9.2118914999	12.7373063252

O	1.0451673552	15.8326179619	12.6097884577

O	7.7472887178	12.2388902795	12.7727965754

H	0.8352292853	2.8408141074	12.9874432363

O	8.8372216523	13.5382682444	14.7037181190

H	1.9277783211	0.3494453241	12.4299159610

H	-1.3316668897	14.0347357434	14.9111676851

O	7.5868363143	18.7556137002	13.0159679943

O	2.8180254204	9.7870133783	12.4427628958

Fe	0.9334757159	17.4207109183	13.5779970369

H	7.8072524940	11.5824152088	13.5212089044

Fe	6.9794240070	7.8171471678	12.5946355880

Fe	7.3990297780	5.7115755209	14.6222318713

O	9.9179635881	17.4937870993	13.3468717629

H	5.9639800260	17.6289789863	13.2251468199

Fe	9.8370315449	3.3132432538	14.2807070895

O	2.6751162317	1.5728991636	14.7747763267

O	2.1949849597	2.9068620458	14.2138195013

O	8.4149112092	4.3653607928	14.0881303470

O	8.1758315945	7.3807761132	14.0009470230

H	3.6085996660	3.8346684930	13.9779786540

O	3.2147464568	7.2710132402	14.2894010756

Fe	9.1950563871	19.0017397254	13.8373049395

Fe	7.8317974808	14.0770275969	13.2097835502

H	8.5582593616	16.1524011872	13.8510521201

La	4.3283737503	9.8220047643	14.4007295375

O	4.2792934776	14.7634534091	14.3157571304

O	4.5334007665	4.2919029286	13.9097656736

Fe	4.3368734842	5.8837231001	15.0885616573

O	8.7439843197	1.6329402526	14.7583410754

O	2.9518929010	5.5050619393	16.4937097211

O	1.9584672760	17.3385432149	15.1569560139

O	5.2499189120	12.0001239732	14.7633761633

O	5.9930957610	6.1209492342	15.6070345778

H	2.5042008885	16.5081024181	15.1511838852

O	10.9478629564	4.2357439443	15.4299619088

O	0.1865402923	19.4334854862	14.1038976154

Fe	3.9382739097	13.0949066061	14.5903555723

O	7.7806545440	15.8588349752	13.2834332715

O	7.9004449497	10.8528927840	14.8131346087

H	8.3538512604	11.6724969560	15.2496940085

H	3.2781814726	18.4159341806	15.2703798154

H	0.3260018531	19.7191934470	15.0896459007

Fe	2.1766820900	7.1472649534	15.8797496751

H	7.8174956122	1.9546417437	14.8363468048

H	8.1097199720	8.1424337642	19.0132452467

H	6.9849983215	10.4372133623	15.5251366039

O	2.7038796981	11.8124981367	14.1373695309

O	4.6583149185	2.2400200932	16.4780588298

La	1.8706829023	3.4823420931	16.6816033601

O	4.2310965150	18.7861462564	15.1532298918

O	2.6420538885	8.9435264738	16.4916005390

Fe	4.3989611382	20.5455109237	15.0673887019

O	5.6898041820	1.7722365388	13.9006685119

Fe	10.5112463794	5.6727100686	16.5200259716

O	8.7407897777	8.4807706836	18.3101247625

La	8.9213762907	8.7464944724	15.8013755784

O	0.1810337793	7.4178770493	16.0538172583

O	0.4255305812	4.9771790937	18.0761815974

O	0.3137747981	20.5260441195	16.8927668406

O	10.9094255695	10.1055729606	15.4799879372

O	8.6664544635	5.9771209243	16.5280152716

Fe	0.9027070704	9.3610656300	16.6068613617

O	6.3713800862	9.5326682346	16.2147161172

H	-0.0403463518	4.9754522642	18.9283638688

H	6.3860320296	9.8034807546	17.1682294930

H	8.0646983395	5.9891158139	17.3747752732

O	2.4530540090	1.8703361067	18.3628402260

Fe	1.3721184137	19.7281961030	17.9332264985

O	8.6673310242	11.1398038749	17.6255587979

O	0.1894415143	10.0032246571	18.2031469246

Fe	9.5212669112	10.2133143316	18.7029926866

O	9.1700364019	10.4642054434	20.2649123674

O	1.4723533026	18.0069345270	17.9187175311

H	1.8144833267	17.6355294298	17.0915283221

Final State

O	5.0523001600	2.9530955200	2.0001393900

Fe	10.6072052900	12.4917291200	3.5853705100

Fe	2.3695802900	17.2477291200	3.5853705100

Fe	7.8613302900	7.7357291200	3.5853705100

Fe	5.1154552900	12.4917291200	3.5853705100

Fe	10.6072052900	2.9797291200	3.5853705100

Fe	2.3695802900	7.7357291200	3.5853705100

Fe	7.8613302900	17.2477291200	3.5853705100

Fe	5.1154552900	2.9797291200	3.5853705100

La	7.8613302900	10.9062689600	4.7067623400

O	0.9966427900	13.2842689600	4.7067623400

O	6.4883927900	3.7722689600	4.7067623400

O	5.1154552900	1.3942689600	4.7067623400

O	10.6072052900	10.9062689600	4.7067623400

O	3.7425177900	3.7722689600	4.7067623400

O	9.2342677900	13.2842689600	4.7067623400

O	3.7425177900	18.0402689600	4.7067623400

O	9.2342677900	8.5282689600	4.7067623400

O	2.3695802900	15.6622689600	4.7067623400

O	7.8613302900	6.1502689600	4.7067623400

O	0.9966427900	18.0402689600	4.7067623400

O	6.4883927900	8.5282689600	4.7067623400

O	0.9966427900	3.7722689600	4.7067623400

O	6.4883927900	13.2842689600	4.7067623400

O	5.1154552900	10.9062689600	4.7067623400

O	10.6072052900	1.3942689600	4.7067623400

O	3.7425177900	13.2842689600	4.7067623400

O	9.2342677900	3.7722689600	4.7067623400

O	3.7425177900	8.5282689600	4.7067623400

O	9.2342677900	18.0402689600	4.7067623400

O	2.3695802900	6.1502689600	4.7067623400

O	7.8613302900	15.6622689600	4.7067623400

O	0.9966427900	8.5282689600	4.7067623400

O	6.4883927900	18.0402689600	4.7067623400

Sr	5.1154552900	6.1502689600	4.7067623400

Sr	10.6072052900	15.6622689600	4.7067623400

Sr	2.3695802900	10.9062689600	4.7067623400

Sr	7.8613302900	1.3942689600	4.7067623400

Sr	5.1154552900	15.6622689600	4.7067623400

Sr	10.6072052900	6.1502689600	4.7067623400

La	2.3695802900	1.3942689600	4.7067623400

H	4.0055726200	3.4083398400	5.5124485100

Fe	2.3695802900	4.5649990400	5.8274838700

Fe	7.8613302900	14.0769990400	5.8274838700

Fe	5.1154552900	18.8329990400	5.8274838700

Fe	10.6072052900	9.3209990400	5.8274838700

Fe	2.3695802900	14.0769990400	5.8274838700

Fe	7.8613302900	4.5649990400	5.8274838700

Fe	5.1154552900	9.3209990400	5.8274838700

Fe	10.6072052900	18.8329990400	5.8274838700

H	6.1502108200	15.6805320000	6.5222373800

O	0.8937274000	5.2374974400	6.7937040200

O	6.4350129800	14.8023841600	6.8774900200

O	2.3713376500	2.9797291200	6.9043015400

O	7.8506762900	12.4987680000	6.9398268100

O	6.4883927900	5.3577291200	6.9485405500

O	0.9966427900	10.1137291200	6.9485405500

O	6.4883927900	0.6017291200	6.9485405500

O	5.1154552900	17.2477291200	6.9485405500

O	10.6072052900	7.7357291200	6.9485405500

O	3.7425177900	0.6017291200	6.9485405500

O	9.2342677900	10.1137291200	6.9485405500

La	5.1154552900	12.4917291200	6.9485405500

La	10.6072052900	2.9797291200	6.9485405500

O	0.9966427900	0.6017291200	6.9485405500

O	6.4883927900	10.1137291200	6.9485405500

O	5.1154552900	7.7357291200	6.9485405500

O	10.6072052900	17.2477291200	6.9485405500

O	3.7425177900	10.1137291200	6.9485405500

O	9.2342677900	0.6017291200	6.9485405500

La	5.1154552900	2.9797291200	6.9485405500

La	10.6072052900	12.4917291200	6.9485405500

Sr	2.3695802900	17.2477291200	6.9485405500

Sr	7.8613302900	7.7357291200	6.9485405500

O	3.7425177900	14.8697291200	6.9485405500

O	9.2342677900	5.3577291200	6.9485405500

Sr	2.3695802900	7.7357291200	6.9485405500

Sr	7.8613302900	17.2477291200	6.9485405500

O	3.7425177900	5.3577291200	6.9485405500

O	9.2342677900	14.8697291200	6.9485405500

O	7.8613302900	2.9797291200	6.9485405500

O	0.9966427900	14.8697291200	6.9485405500

O	2.4450369400	12.3616049600	7.1137665400

H	8.1754583900	13.0875608000	7.7435021200

Fe	10.5895218500	6.1038504000	7.8641539600

H	2.5653062600	3.3647748800	7.8725325600

H	7.8269519400	3.2513918400	7.9331936200

Fe	7.8798924100	10.7810910400	8.0427857100

O	9.1029051300	14.0459899200	8.2415261000

O	10.0182700200	17.5970097600	8.2840893900

Fe	2.0490817600	10.5316864000	8.3551399200

Fe	8.5451630000	1.1806294400	8.6983273800

La	4.7660701600	15.6556105600	8.8648939400

La	5.2362737900	6.4953643200	8.8933811800

La	10.8969500200	15.4689851200	8.8963974800

La	5.3354548000	18.9610305600	8.9423122100

Fe	2.6017714800	12.8533753600	8.9493502300

O	4.8802985600	2.1430536000	8.9868863600

O	3.1007518900	3.9583236800	9.0147033100

O	0.4881067400	11.1630929600	9.0193953300

O	0.8069577500	13.4155345600	9.1001650300

O	6.8354713900	16.6496145600	9.1078733400

O	1.6993671200	8.9568796800	9.1785887300

O	4.4958760600	13.3234584000	9.1799293000

O	6.9200443400	8.1626276800	9.1842861800

O	9.1132296200	11.4183950400	9.2617044400

O	3.1784052300	17.9961332800	9.2751102000

O	9.7547758600	6.8115432000	9.2891862500

O	0.3292853300	4.1320128000	9.3428092900

O	7.9736915000	2.8515073600	9.3485067400

O	2.3607934900	6.4147025600	9.4192221200

O	10.2518890600	1.5162128000	9.5237870500

La	10.2556234500	9.1992454400	9.5948375800

H	2.7726747400	17.1326339200	9.6886779000

O	2.0364507400	15.9341219200	9.7000727900

O	6.8283321200	12.0471382400	9.7493389600

La	0.8351853400	18.8269113600	9.7516849700

H	8.8702746000	13.9331776000	9.8602716200

O	3.0048659300	11.1824974400	9.9413764700

Fe	1.9500105900	4.8349496000	10.0238219000

O	7.7015736648	0.0428502591	9.8364596787

O	4.4886789886	7.7190365237	10.6588463351

La	5.6651610902	9.6434472879	10.4061348750

Fe	3.6001844631	2.6880710200	10.4416951961

O	1.8412936361	2.0035969422	10.7552665072

Fe	0.1158317298	2.8204223766	10.7282163147

Fe	11.0714033180	12.4265119339	10.5864328437

Fe	5.6496720069	13.2007602860	10.6853581970

O	9.3784124611	13.7531797587	10.6939011633

O	10.2705617994	17.0177079223	10.8434423974

O	6.1646039802	4.7468856822	10.4243032319

Fe	7.7155072882	3.8715758595	10.7728881736

Fe	10.1564002340	6.2566058159	11.0019306284

La	7.8845865582	17.1158313417	11.1012328077

Fe	8.2937883924	11.8110163948	10.9097018426

O	7.8898315818	9.8076789134	10.9861924422

La	2.2999962437	7.7591106668	11.4978086860

O	9.1313344982	4.8435682530	11.4352064502

O	4.1356142649	14.9195367437	11.2557191688

H	4.3421654692	0.5248472721	12.3242046505

O	6.6766251903	14.3924709828	11.6545502467

Fe	2.1877738530	14.9844586335	11.5111920343

H	8.6113370191	2.0018212468	12.1589398015

O	4.9896153416	0.9186800833	11.6546505546

O	10.9880259475	7.9666130392	11.4889057414

O	3.6815288179	4.2658755183	11.4408697742

O	0.9689572473	5.4941304398	11.6258174429

O	-0.7059076790	11.0313738618	11.5218325987

O	1.7065788938	13.1672496494	11.5048241426

Fe	2.7982847959	11.5867673078	11.8031123523

H	10.8385034409	16.5660724324	11.5195613334

O	5.7408182808	17.1242401533	12.6412559906

Fe	6.5517077069	1.1196657717	12.7991423039

O	4.8462006671	11.8989018149	11.7762051741

Fe	5.2903119842	4.9413004441	12.0186129306

O	7.7372002667	2.4538158182	12.1598740000

O	6.4800346586	6.0546848252	12.7494818447

O	1.4088477562	18.6062485574	12.0755371645

H	5.2438842328	11.8724335567	12.7583624380

O	10.4818719544	2.6139352303	12.5871214479

Fe	5.2147433692	15.2598814083	12.7738246715

H	0.8899653495	4.8878539537	12.3915928285

H	-0.5073781218	10.8343396160	12.4747392559

O	8.5838280729	8.7598179549	11.5680534710

O	6.0053773949	9.0533567633	12.7307455426

O	1.0590229425	15.9048723367	12.5643557821

O	7.5879263777	12.2334193296	12.6051458463

H	-0.1297120709	1.8218599617	13.1493211825

O	9.4923997308	13.5178590007	13.8903802290

H	1.6229548042	0.5767003068	12.2553213703

H	-0.8952861807	14.2924896029	13.8543352786

O	7.8358814166	18.7730041112	12.9976331610

O	2.9362601661	9.9368123339	12.4222896284

Fe	0.8301033304	17.4911695738	13.5312939072

H	7.6664696821	11.5346373717	13.9933148461

Fe	7.2590629367	7.7360205387	12.5083931132

Fe	7.3190938085	6.0133759548	14.8526318138

O	9.9915982034	17.4390719777	13.9685142373

H	5.7561401534	17.5294383055	13.5744755158

Fe	9.4910445002	3.5663869921	14.0991194195

O	3.2275452584	1.3443313876	14.4568404580

O	2.7456193326	2.6392417975	14.2453073382

O	8.0483944690	4.3891193091	14.6283135304

O	8.2993903771	7.5079675409	14.0631944263

H	4.0997054415	3.5236718686	13.7797432579

O	3.2110231973	7.2743785377	13.7540255374

Fe	9.3615799342	19.0814287344	13.9592989423

Fe	8.1119112913	13.9088217322	12.7894708382

H	8.8096589915	15.8891770299	13.8056643849

La	4.4593264219	9.4405757253	14.2848076598

O	4.6057548558	14.5676432079	14.3054617066

O	4.8715464616	4.1586363018	13.6106934332

Fe	4.1449489123	5.6635389469	14.6456865128

O	8.7398894986	1.7646647315	14.7517249993

O	2.6492309200	5.5460434331	15.7727943961

O	1.9364440518	17.2576168754	15.2208184477

O	5.4353000560	11.7180311287	14.2925820202

O	5.6293206373	6.2743102464	15.4306701833

H	2.3875145028	16.4156728118	15.1247925833

O	10.8224574302	4.2755858349	15.2058894831

O	0.1391915961	19.7162617302	14.1303707873

Fe	4.3042170012	12.9146579833	14.8044953078

O	8.0930180123	15.6246846615	13.1686903169

O	8.2248182595	11.0530885430	14.7400546734

H	8.8673362477	11.7769550981	15.0406865399

H	3.2850627628	18.2275473541	15.6560712481

H	0.2834642512	20.2053319337	15.0370742994

Fe	2.0021657532	7.3438771597	15.2165939037

H	7.7972780849	1.8143143453	15.0486769674

H	12.0024984501	8.4545749539	17.3877403907

H	6.8476595759	10.4324887228	15.8752380707

O	2.9430591093	11.7267470041	15.2169338234

O	4.2875446970	2.0893616895	16.8658466944

La	1.9831586134	3.2854950625	16.6081193813

O	4.2801002347	18.5410811102	15.8709126288

O	2.4973607787	8.9879738202	15.9348299807

Fe	4.5868721996	20.2143867848	15.5002671056

O	6.0486544715	1.2775361814	14.5980398536

Fe	10.5727924372	5.7941586056	16.2978027256

O	11.0602469778	8.5053103429	17.5720095942

La	9.5339839731	8.9758494172	15.4123541710

O	0.1872624082	7.1997063495	15.3402814429

O	0.4115469331	4.9044959070	17.9416571512

O	0.4643523630	20.7085531098	16.2943621645

O	11.2694266622	10.1678723562	14.4370921105

O	8.6965116624	6.3537975028	16.5927345681

Fe	1.5491782525	10.6749371176	15.5844898398

O	6.2382891546	9.7754927528	16.2104535378

H	0.9167396145	5.5888395024	18.4163502843

H	6.4574371634	9.5328680143	17.1053594462

H	8.3429723456	5.8410425385	17.2927811580

O	1.6102075910	1.6648188076	18.6113300310

Fe	0.7202693586	19.6998978130	17.7089575216

O	9.1776498253	10.3569930447	17.4137045086

O	0.7113102933	11.0906972583	17.1791098965

Fe	10.6278242787	10.2311678674	18.1666071644

O	10.7504693450	10.6100874632	19.7473406829

O	0.6389802094	18.0270865833	17.5191668204

H	1.2385193622	17.5284198191	16.8361042214
